# Dynamic carbon flux network of a diverse marine microbial community

**DOI:** 10.1038/s43705-021-00055-7

**Published:** 2021-09-25

**Authors:** Marvin M. Mayerhofer, Falk Eigemann, Carsten Lackner, Jutta Hoffmann, Ferdi L. Hellweger

**Affiliations:** Water Quality Engineering, Technical University of Berlin, Berlin, Germany

**Keywords:** Microbiology, Ecology, Ecology, Biogeochemistry

## Abstract

The functioning of microbial ecosystems has important consequences from global climate to human health, but quantitative mechanistic understanding remains elusive. The components of microbial ecosystems can now be observed at high resolution, but interactions still have to be inferred e.g., a time-series may show a bloom of bacteria X followed by virus Y suggesting they interact. Existing inference approaches are mostly empirical, like correlation networks, which are not mechanistically constrained and do not provide quantitative mass fluxes, and thus have limited utility. We developed an inference method, where a mechanistic model with hundreds of species and thousands of parameters is calibrated to time series data. The large scale, nonlinearity and feedbacks pose a challenging optimization problem, which is overcome using a novel procedure that mimics natural speciation or diversification e.g., stepwise increase of bacteria species. The method allows for curation using species-level information from e.g., physiological experiments or genome sequences. The product is a mass-balancing, mechanistically-constrained, quantitative representation of the ecosystem. We apply the method to characterize phytoplankton—heterotrophic bacteria interactions via dissolved organic matter in a marine system. The resulting model predicts quantitative fluxes for each interaction and time point (e.g., 0.16 µmolC/L/d of chrysolaminarin to *Polaribacter* on April 16, 2009). At the system level, the flux network shows a strong correlation between the abundance of bacteria species and their carbon flux during blooms, with copiotrophs being relatively more important than oligotrophs. However, oligotrophs, like SAR11, are unexpectedly high carbon processors for weeks into blooms, due to their higher biomass. The fraction of exudates (vs. grazing/death products) in the DOM pool decreases during blooms, and they are preferentially consumed by oligotrophs. In addition, functional similarity of phytoplankton i.e., what they produce, decouples their association with heterotrophs. The methodology is applicable to other microbial ecosystems, like human microbiome or wastewater treatment plants.

## Introduction

Microbes are members and affect the functioning of many ecosystems, from the human gut to the global ocean, with important implications for health and climate. Components of these complex, diverse and dynamic systems, e.g., microbes and substrates, can be observed at high resolution using modern technologies [1–4]. However, a critical step towards a quantitative understanding is to also characterize interactions, i.e., how mass moves through these ecological networks. What species or functional groups (e.g., oligotrophs, copiotrophs) process carbon and how does this change over time? Is the association between producers and consumers conserved/static or decoupled/dynamic? For mass fluxes, observations are still limited to few samples, and bulk ecological compartments or select types [5–7]. Consequently, interactions have to be inferred from observations of components, like time series data.

Past approaches to infer interactions from microbial time series data have been mostly empirical, including principal component analysis (PCA), non-metric multidimensional scaling (NMDS), empirical dynamic modeling (EDM) and various regression and correlation analyses [1–3, 8–11]. Those methods may consider time lags and local interactions (i.e. considering only a subset of the time series) [2, 10], and interactions inferred from those methods can be depicted using association or interaction networks. Past examples include phage—cyanobacteria genotypes [2], DOM species—bacteria genotypes [1], ciliate morphotypes—phytoplankton genera [12], and lake bacteria - phytoplankton—environmental factor [10] interactions. These empirical methods can point to possible interactions, but results can be difficult to interpret mechanistically (e.g., virus-virus interaction) and are not quantitative (e.g., do not provide carbon flux between species). These shortcomings limit the utility of empirical methods to develop a quantitative mechanistic understanding of microbial ecosystems.

Mechanistic models describe the time evolution of components using differential mass balance equations that include specific interaction terms, like exudation of dissolved organic matter (DOM) by phytoplankton and assimilation by heterotrophic bacteria (hereafter bacteria). Parameters, like half-saturation constants, can be calibrated to observations using numerical optimization routines, but past applications have been limited to few components [13, 14]. One concern with larger models is parameter uncertainty, which may lead to getting the “right answer for the wrong reason”, although there are counterarguments [15, 16].

Here we propose that, in the context of flux inference (vs. prediction), even a model that gets “the right answer for the wrong reason” may be useful. On the one hand, if the model overestimates the gain and loss of some species it may still match the concentration data, but it would overestimate the flux, which would be a problem. On the other hand, if the model overestimates temperature limitation and underestimates light limitation, it may still get the right growth rate and flux. This would be a problem for prediction (i.e., climate change), but not for flux inference. Furthermore, we propose that a mechanistic model, because it is mass-balancing and mechanistically constrained, is less likely to produce the “right answer for the wrong reason” than an empirical correlation analysis.

We developed a method to generate a dynamic carbon flux network based on mechanisms, and informed by general and species-level literature data (FluxNet), and applied it to a marine time series of phytoplankton, organic matter and bacteria. The result is a mass-balancing, mechanistically-constrained, quantitative representation of the ecosystem. Analysis of this network provides insights into carbon processing of individual members and groups (i.e., oligotrophs) and associations between phytoplankton producers and bacteria consumers.

## Results and discussion

### Overview of the FluxNet method

The FluxNet approach is based on a mechanistic model, which includes multiple species/types of phytoplankton, bacteria, dissolved and particulate organic matter (DOM, POM), inorganic nutrients, micronutrients and inhibitors (see Table 1). For phytoplankton—bacteria carbon flux, which is the focus here, phytoplankton produce organic carbon by exudation and death. For exudation, living phytoplankton produce total DOM at constant and photosynthesis-proportional rates (*ke*, *ef*), with a composition defined by an exudation fraction (*Fe*) for each DOM species. These parameters vary by phytoplankton type. For example, for green algae (*gre*), the constant exudation rate is *ke*_*gre*_ and the fraction of glucose-containing HMW DOM (*gl2*) is *Fe*_*gre,gl2*_. For one phytoplankton type the total DOM production varies in time with the photosynthesis rate, but the composition is constant. Phytoplankton die by a general death function and inhibition. The death function is time-variable (a bell-shaped function with a maximum at a specific time of year) and does not differentiate between various death mechanisms like zooplankton grazing or viral lysis, but presumably it represents mostly grazing in this case. Upon death, the phytoplankton biomass is converted to POM and DOM, where e.g., the content of chrysolaminarin (*chr*) for the diatom *Rhizosolenia styliformis* (*rst*) is defined by a composition fraction (*Fx*_*rst,chr*_). POM dissolves to DOM at a first-order rate. Bacteria consume DOM using Monod-level kinetics, where e.g. the affinity for *Polaribacter* (*pol*) for chrysolaminarin is defined by a half-saturation constant (*Ksh*_*pol,chr*_).

**Table 1:** Model components.

*Phytoplankton*	*Bacteria*
Chlorophyll a (BBE) (chl) [S]	Total bacteria by DAPI (dap) [S]
Diatoms (dia) [S]	EUB338-I-III, Eubacteria (eub) [S]
Diatoms—Pennales (dip)	ALF968, Alphaproteobacteria (alf) [S]
Diatoms—Centrales (dic) [S]	SAR11–486, SAR11, Alphaproteobacteria (s48)
Greenalgae (gre)	SAR11–441, SAR11, Alphaproteobacteria (s44)
Dinoflagellates (dif) [S]	ROS537, *Roseobacter*, Alphaproteobacteria (ros) [S]
Silicoflagellates (sif)	NAC11-7-1030, Nac11, *Roseobacter* (nac)
Coccolithophorids (coc)	RCA1000, RCA/DC5, *Roseobacter* (rca)
*Mediopyxis helysia* (mhe)	GAM42a, Gammaproteobacteria (gam) [S]
*Chaetoceros debilis* (cde)	REI731, *Reinekea*, Gammaproteobacteria (rei)
*Chaetoceros minimus* (cmi)	Bal731, *Balneatrix*, Gammaproteobacteria (bal)
*Rhizosolenia styliformis* (rst)	OM182–707, OM182, Gammaproteobacteria (om1)
*Thalassiosira nordenskioeldii* (tno)	NOR5–730, NOR5, Gammaproteobacteria (nor)
Dinophyceae (din)	PSA184, *Pseudoalteromonas*, Gammaproteobacteria (psa)
*Phaeocystis* (pha)	ALT1413, *Alteromonas*, Gammaproteobacteria (alt)
*Chattonella* (cha)	GV841, *Vibrio*, Gammaproteobacteria (gv8)
Cryptic: (dix), (dxf), (ph1)	SAR92–627, SAR92, Gammaproteobacteria (s92)
	SAR86–1245, SAR86, Gammaproteobacteria (s86)
***POM and DOM***	Glac227, *Glaciecola*, Gammaproteobacteria (gla)
POC (poc) [S]	CF319a, Bacteroidetes (cf3) [S]
DOC (doc) [S]	POL740,* Polaribacter*, Bacteroidetes (pol)
Arabinose (ara) [S]	FORM181A, *Formosa*, Bacteroidetes (foa) [S]
Fucose (fuc) [S]	FORM181B, *Formosa* Hel1_33_131, Bacteroidetes (fob)
Galactose (gal) [S]	ULV995, *Ulvibacter*, Bacteroidetes (ulv)
Glucose (glc) [S]	VIS6–814, VIS6, Bacteroidetes (vis)
Mannose/xylose (max) [S]	NS3a-840, NS3a marine group, Bacteroidetes (ns3)
Rhamnose (rha) [S]	NS5/DE2–471, NS5/DE2, Bacteroidetes (nde)
Galacturonic acid (gau) [S]	NS5/VIS1–575, VIS1, NS5 (nvi)
Gluconic acid (glu) [S]	NS9–664, NS9 marine group, Bacteroidetes (ns9)
Glucuronic acid (gca) [S]	CYT-734, *Marinoscillum*, Bacteroidetes (cyt)
Muramic acid (mur) [S]	PLA46, Planctomycetes (pa4) [S]
Galactosamine (gan) [S]	PirD1039, *Pirellula*, Planctomycetes (pir)
Glucosamine (gln) [S]	uPlaB440, Planctomycetes group B (upl)
Chitin (chi)	PlaA1228, Planctomycetes group A (pa1)
Chrysolaminarin (chr)	ARCH915, Archaea (arc) [S]
Glycogen (gly)	EURY806, Euryarcheota (eur)
Xylan (xyl)	CREN554, Crenarcheota (cre)
Cellulose (cel)	BET42a, Betaproteobacteria (bet)
Mannan (man)	SAR324–1412, SAR 324, Deltaproteobacteria (s32)
Starch (sta)	HGC69a, Actinobacteria (hgc)
Pectin (pec)	Cryptic: (eux), (alx), (rox), (gax), (cfx), (fox), (px4), (arx)
Glucoromannan (glo)	
FCSP (fcs)	***Nutrients and misc***.
Rhamnan (rhm)	Nitrate+nitrite (nox)
POM Chrysolaminarin (lam) [S]	Ammonium (nh4)
Biogenic silica (bsi)	Phosphate (po4)
Cryptic: (d01+), (ar2), (fu2), (ga2), (gl2), (ma2), (rh2), (ga3), (gl3), (gc2), (mu2), (ga4), (gl4), (m01+), (i01+), (p01+), (pra), (puc), (pal), (plc), (pax), (xph), (pau), (plu), (pca), (pur), (pan), (pln), (phi), (phr), (ply), (pyl), (pel), (xpa), (pta), (xpe), (plo), (pcs), (phm)	Silicate (sil) Light extinction coefficient (kex) [S] Inorganic suspended solids (nkx)

[S] = summary parameter.

The novel aspect is the upscaling to hundreds of state variables and thousands of parameters, which is accompanied by several conceptual and practical modeling challenges. To balance mass and account for the action of unobserved components, cryptic or hypothetical species are included [17], like DOM types *d01*-*d15*, which may represent e.g., threonine [18]. To simulate a diverse community with a smaller number of drivers (“paradox of the plankton”) and control chaos, interaction via micronutrients and inhibitors, as well as dormancy is included [19–22]. Parameters are optimized/calibrated to minimize the discrepancy between the model and observations. Which parameters are optimized and the corresponding ranges is based on available information (complete model equations and parameters are in Table S1–S25). For example, the constant DOM production rate (*ke*) is optimized for all phytoplankton, with a range adopted from a previous modeling study [23]. For *rst (Rhizosolenia styliformis)*, the exudation fractions for most DOM components, like the cryptic species *d01* (*Fe*_*rst,d01*_), are optimized. Others, like glucose-containing HMW DOM (*Fe*_*rst,gl2*_), are fixed based on literature (Table S14). The optimization is challenging because of the many components, nonlinear interactions, and resulting local optima in the objective function. We developed an optimization routine customized for microbial ecosystems with a number of key features.

**Fig. 1:**
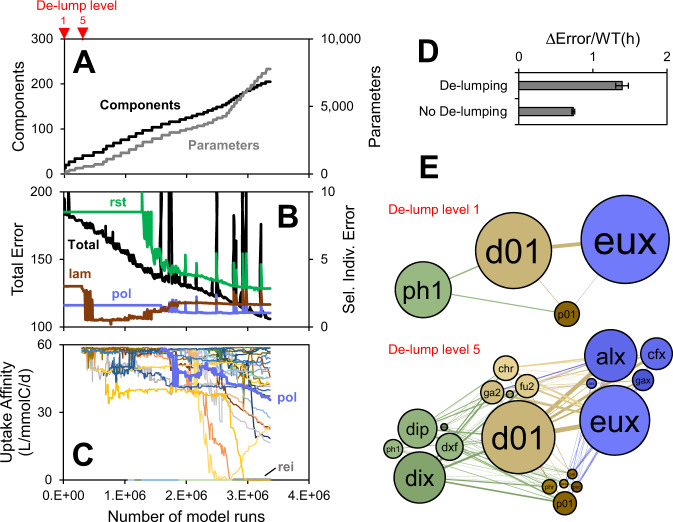
FluxNet inference method illustration. **A** Number components and optimized parameters. **B** Error for entire model (Total) and selected individual observations (*rst* = *R. styliformis*, *pol* = *Polaribacter*, *lam* = particulate chrysolaminarin). Best of 128 replicate runs. **C** Diversification of chrysolaminarin uptake affinity (max. heterotrophy rate/half-saturation constant). **D** Method performance with and without de-lumping. **E** Network corresponding to different de-lump levels. See Table 1 for component names and abbreviations.

First, the method mimics natural speciation, where a coarse-grained model is gradually de-lumped to a finer resolution, a strategy also used in manual model development [13, 24, 25]. This is illustrated in Fig. 1, which shows how the model starts with just one component in each ecological compartment (Fig. 1E). This model is optimized until a threshold is reached, and then all species are de-lumped/split into two, followed by another round of optimization and so on. During the course of the optimization, with time or model runs, the number of components and parameters increase, and the total error generally decreases, although there can be a transient increase when new species are introduced (Fig. 1A, B). This way the optimization routine works with a smaller model on average and computational effort can be directed to a smaller set of parameters corresponding to newly introduced species, and the performance increases (Fig. 1C).

At each de-lumping level, the new species generally inherits the parameter values (i.e., the genome [26]) from the old species. Subsequent optimization then diversifies the population. This is illustrated in Fig. 1C, which shows the uptake affinity of all bacteria species for *chr*. However, different parameter values can also be specified for the new species, and then they are adopted and overwrite those inherited from the old species. This is used, for example, to assign species-specific cell sizes or prevent species from taking up a substrate. In Fig. 1C, those species that are not capable of assimilating *chr*, like *rei (Reinekea)*, have an affinity equal to 0. The method thus allows for natural and automated expansion of the model to very large scale, yet provides a way to constrain/curate it based on available information.

Second, the routine includes multi-parameter optimization (Nelder-Mead simplex method) on selected subsets of dependent parameters, like those involved in the production and consumption of chrysolaminarin (*chr*) or directly affecting the photosynthesis of the diatom *R. styliformis* (*rst*). Dependence between parameters, like max. photosynthesis rate and nutrient half-saturation constant, are explicitly considered. Also, Monte Carlo scans are performed on selected parameter sets at various points in the process.

### Application to Helgoland time series

The FluxNet method is applied to a four-year time series at Helgoland [27], including near-daily observations of 15 phytoplankton and 38 heterotrophic bacteria types (e.g., species, strains) and various bulk and auxiliary parameters (e.g., Chlorophyll *a*, DAPI, temperature, nitrate+nitrite, ammonium, phosphate, light extinction) (Tables S19 and S20). Data from more focused studies characterizing DOM and POM are also included [28, 29] (Table S21).

In addition to the time-series data, the model is informed by literature information. Model parameters, incl. general properties like phytoplankton exudation fraction or bacteria growth efficiency, are constrained based on past models and data. Also, constraints are implemented for parameters controlling composition, exudation and utilization for the specific components included in the model. Those were based on a literature meta-analysis, where we searched primarily for studies with strains from Helgoland, but included strains from other locations if necessary. These constraints include, for example, for the phytoplankton storage polysaccharide chrysolaminarin, the typical content (~30% for diatoms, none for dinoflagellates) and ability of bacteria to assimilate it (yes for *Polaribacter*, no for *Roseobacters* and *Reinekea*) (Tables S4 and S11). Imposing constraints from the literature generally results in a worse agreement with the observations, but also increased realism of the model. Removing the constraints of phytoplankton composition (Table S4) significantly improves the agreement with observations, but also predicts substantial glycogen content of diatoms (e.g., *Fx*_*mhe,gly+ply*_ = 0.19). Removing uptake constraints by bacteria (Table S11) reduces the error, but not significantly, suggesting that there is enough flexibility of the model to reproduce the observations even with this constraint. However, that model also includes features that disagree with literature, like substantial uptake of *chr* by *s11* (*Ksh*_*s11,chr*_ = 25 L/mmolC/d).

### Carbon fluxes through and within in the ecosystem

**Fig. 2:**
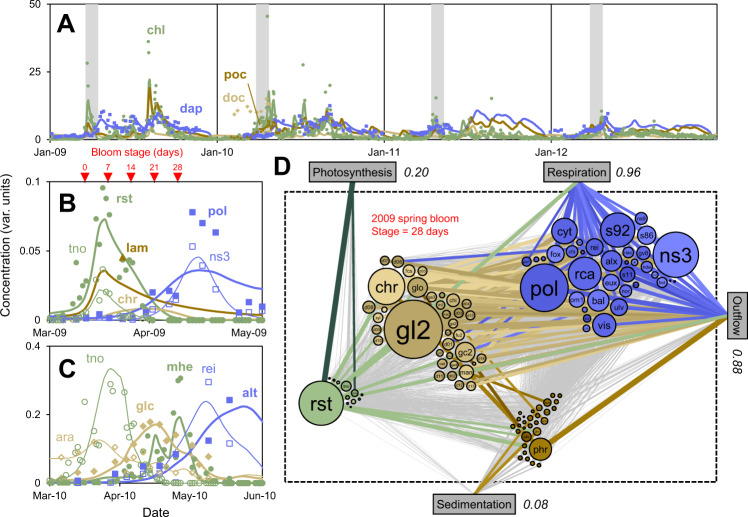
FluxNet model results and comparison to data. **A** All model types lumped. Phytoplankton (*chl*, μgChl*a*/L), POM (*poc*, incl. microbes, μmolC/L ×0.1*), DOM (*doc*, μmolC/L ×0.1*), bacteria (*dap*, 1e6/mL ×3*). Gray shading are spring blooms, defined as the first time of the year the phytoplankton exceeds 3 µgChl*a*/L plus 28 days. **B** Selected types for 2009 spring bloom. *Rhizosolenia styliformis* (*rst*, centric diatom, 1e6/L ×1.2*), *Thalassiosira nordenskioeldii* (*tno*, centric diatom, 1e6/L ×0.05*), particulate chrysolaminarin (*lam* = *phr* + phytoplankton content, μmolC/L ×0.002*), dissolved chrysolaminarin (*chr*, μmolC/L ×0.002*, no data available), *Polaribacter* (*pol*, DAPI × CARD-FISH, 1e6/mL ×0.1*), NS3a marine group (*ns3*, 1e6/mL ×0.2*). **C** Selected types for 2010 spring bloom. *Mediopyxis helysia* (*mhe*, centric diatom, 1e6/L), *Thalassiosira nordenskioeldii* (*tno*, centric diatom, 1e6/L), glucose-containing HMW DOM (*glc*, μmolC/L ×0.01*), arabinose-containing HMW DOM (*ara*, μmolC/L ×3*), *Reinekea* (*rei*, 1e6/mL ×5*), *Alteromonas* (*alt*, 1e6/mL ×1.5*). Lines are model and symbols are data [27–29]. *Individual concentration series scaled to illustrate dynamics. See Fig. S1 for all model-data comparisons. Upside-down triangles mark various bloom stages for networks in (**D**) and Fig. 4A. **D** Inferred carbon flux network. Nodes are components. Size indicates in/outflux (μmolC/L/d), color varied randomly within each ecological compartment. Lines are fluxes. Thickness is proportional to log flux (μmolC/L/d), colored based on the source node, lines below a threshold distance are colored gray to highlight most important fluxes. Italic numbers are total fluxes (μmolC/L/d). Flux cut off is 0.01%. See Table 1 for component names and abbreviations. See Movie S1.

The final model includes 210 components and their behavior and interaction are described by a total of 8200 calibrated parameters of 50 different parameter types (e.g., the composition of each of the 53 microbes is described by 76 fractions *Fx*, or 4000 total parameters) (Fig. 1), and it constitutes a mass-balancing, mechanistically-constrained, quantitative representation of the ecosystem. It reproduces many of the observed patterns of summary parameters like Chlorophyll *a* (*chl*), total bacteria (*dap*), particulate chrysolaminarin (*lam*), various high-molecular weight (HMW) DOM compounds, as well as absolute concentrations of individual phytoplankton and bacteria species (Fig. 2A–C). Only subset of the hundreds of model components is shown in Fig. 2B, C, which were selected based on (a) importance (e.g., *rst* is the dominant OM producer in 2009), (b) availability of data (e.g., chrysolaminarin, [29]) and (c) illustration of co-blooming (panel B) and succession (panel C). All model-data comparisons are presented in the SI (Fig. S1). The model under-predicts total DOM (*doc*), probably because a large fraction of observed DOM is more refractory allochthonous material, which is not considered in the model.

It is important to understand that the model was calibrated to these observations, so this is not a prediction *per se*. The main information produced by this analysis (emergent property) are the mass fluxes. Predicted ecosystem-level fluxes can be compared to independent estimates, which were not used as input here. For the period 2009–2012, the gross primary production rate in the model is 28 (±1.2 standard deviation) mmolC/m^2^/d. Uncertainty of fluxes and parameters are based on top 5% of 128 replicate runs, as in [23]. This flux compares well to a regional estimate of 29 (26–33) mmolC/m^2^/d for the Transitional East Region of the North Sea for the same period [30]. At the end of March, the bacterial production rate in the model is 0.32 (±0.041), 0.14 (±0.017), 0.20 (±0.025) and 0.45 (±0.057) μmolC/L/d for the 4 years, respectively. This is consistent with measurements of 0.20 μmolC/L/d in 1992 ~30 km from Helgoland [31].

These comparisons provide confidence in other aggregate fluxes predicted by the model. The C, N and P fluxes to the sediment bed, via settling of phytoplankton and POM, are 5.8 (±0.91) mmolC/m^2^/d, 0.87 (±0.14) mmolN/m^2^/d and 0.054 (±0.0085) mmolP/m^2^/d, which constitute 20%, 16% and 18% of the input via photosynthesis (C) or external input (N, P) (see Fig. S2). External “new” input of N is 0.66 μmolN/L/d, which is 6.0 time higher than the 0.11 (±0.023) μmolN/L/d released or “recycled” by bacteria.

The resulting flux network includes quantitative carbon fluxes between all components at each time point, like 28 days into the 2009 spring bloom (Fig. 2D, Dataset S1 list all fluxes). The dominant source of organic matter is *rst* at 0.36 (±0.19) μmolC/L/d, 30% of which is dissolved and particulate chrysolaminarin (*chr* + *phr*). These instantaneous fluxes exhibit a higher uncertainty than the integrated fluxes discussed in the previous paragraph, which can be explained by small timing differences (Table S26). The DOM is consumed by a diverse consortium of bacteria, mostly *Polaribacter* (*pol*) at 0.46 (±0.22) μmolC/L/d, 35% of which is *chr*. *chr* has a through-flux of 0.25 (±0.049) μmolC/L/d and a turnover time of 8.8 (±2.0) days. In the model, phytoplankton and bacteria interact via DOM, but the carbon flux can be traced and used to quantify phytoplankton – bacteria associations. Here, the carbon flux via all DOM types from *rst* to *pol* is 0.27 (±0.20) μmolC/L/d, 58% of carbon to *pol*, making this the second-strongest (after *ns3*) microbial linkage in the system at this time. This *who produces/consumes how much of what when* information is the main output of the FluxNet method, and it is critical for moving our understanding of microbial ecosystem functioning beyond bulk parameters like respiration and photosynthesis rates towards a higher resolution.

Whereas the 2009 spring bloom illustrates co-blooming of phytoplankton and bacteria, the 2010 bloom shows succession of phytoplankton, DOM and bacteria. Several factors control this pattern in the model. *Reinekea* (*rei*) is negative for chrysolaminarin (*chr*) based on literature (Table S11), but is predicted to have a relatively high affinity for other glucose-containing DOM (*gl2*) (*kh*_*rei*_ / *Ksh*_*rei,gl2*_ = 63 (±22) L/mmolC/d). A substantial fraction of *gl2* is produced relatively early by phytoplankton exudation, and it is the primary substrate for *rei* at bloom stage 14 days. *Alteromonas* (*alt*) is predicted to have a low affinity for *gl2* (*kh*_*alt*_ / *Ksh*_*alt,gl2*_ = 0.015 (±0.0097) L/mmolC/d), but it is positive for *chr* based on literature and predicted to have a high affinity (*kh*_*alt*_ / *Ksh*_*alt,chr*_ = 52 (±4.7) L/mmolC/d). *Chr* is a death (i.e. grazing) product of phytoplankton and produced relatively later in the bloom, and it is the primary substrate for *alt* at this time. The substrate spectra of bacteria emerge in the analysis, within literature constraints, and can be considered a prediction testable with modern experimental techniques [6].

### Oligotrophic and copiotrophic carbon processing

**Fig. 3:**
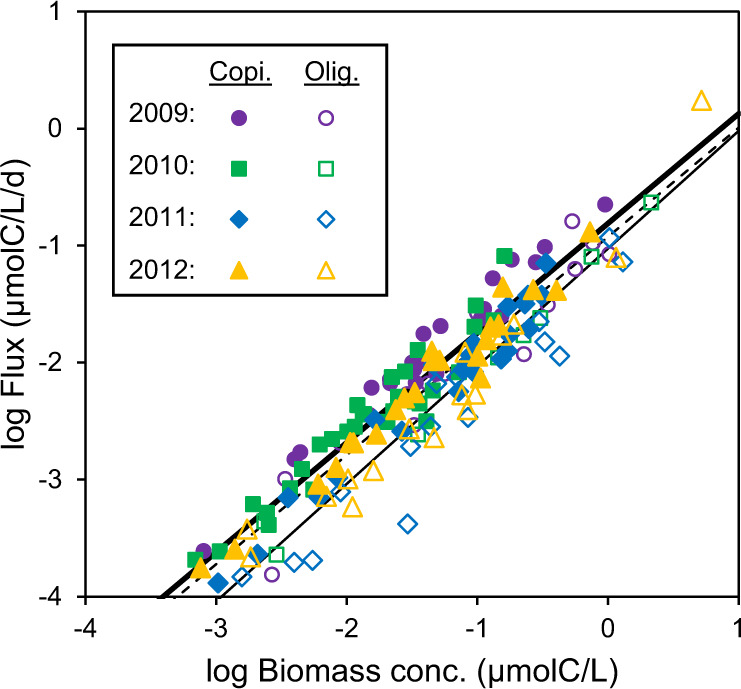
Correlation between spring bloom abundance and importance. Concentration and carbon flux for all model bacteria types during spring bloom periods (see Fig. 2 caption for definition). Lines: All(dashed)/Olig.(thick)/Copi.(thin), log Flux = –0.93/–1.03/–0.81 + 0.93/1.00/0.94 × log Conc., *R*^2^ = 0.88/0.92/0.92.

The network includes concentrations and fluxes for each bacteria type, and a natural question is to what extend they are correlated. There is increasing awareness that high abundance may not necessarily mean high importance and *vice versa*, including the over-proportional role of rare species in biogeochemical cycles [32]. In the model, there is a strong correlation between concentration and carbon flux of bacteria, but for the same concentration there is also about an order of magnitude variation in flux (Fig. 3). The spread reflects differences in growth rates during the bloom periods. Some species, like the oligotroph SAR11 (*s11*), have consistently lower flux and others, like the copiotroph *Polaribacter* (*pol*), have consistently higher flux. There are also some, like the cryptic alphaproteobacteria (*alx*), that go in different directions in different years.

**Fig. 4:**
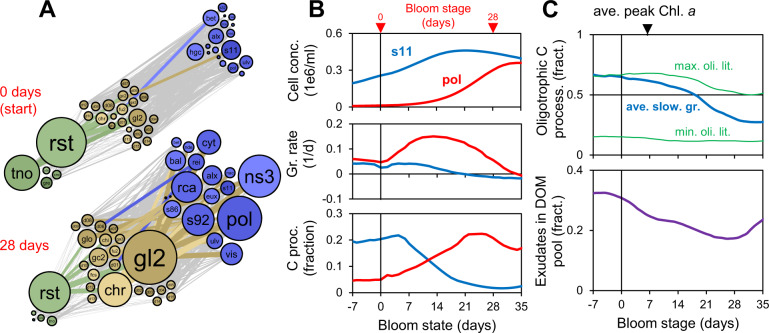
Carbon processing during the course of blooms. **A** Phytoplankton—DOM—bacteria carbon flux network for the start and +28 days of 2009 spring bloom. See Fig. 2 legend. Flux cut off is 0.3%. **B** Cell concentrations, growth rate and relative carbon processing for *s11* and *pol* for 2009 spring bloom. **C** Fraction of DOM processed by oligotrophic bacteria and exudate fraction in DOM pool for all blooms. Oligotrophs are defined based on literature as shown in Table S23 or based on below-average growth rates (*kg*). For the later, the oligotrophic fraction or weight given for type *i*, is based on *fOLI*_*i*_ = *kg*_*AVE*_^*n*^ / (*kg*_*AVE*_^*n*^ + *kg*_*i*_^*n*^), *n* = 5. *kg* is the net growth rate calculated from biomass change, plus dilution rate.

It is important to realize that, in dynamic systems, microbial interactions and the corresponding networks are not static [3, 33]. The dynamics of the entire Helgoland flux network over the four-year period is illustrated in an animation, which shows the production of DOM and POM during and after phytoplankton blooms and later blooming of bacteria (Movie S1). These features are also evident in the phytoplankton – DOM – bacteria interactions at two selected time points during the 2009 spring bloom (Fig. 4A, B). At the onset of the bloom, the oligotroph SAR11 (*s11*) consumes the most DOM, primarily the cryptic species *d08*, which comes mostly from grazing death of green algae (*gre*) and exudation by *rst*. After 28 days the copiotroph *pol* dominates, which consumes primarily *chr*, a death product of mostly *rst*. SAR11 continues to be a major carbon processor in the early parts of the bloom, which was unexpected, because it is an inferior competitor at this time (growth rate *s11* = 0.051 vs. *pol* = 0.15 1/d, bloom average), but can be explained by the higher biomass concentration (*s11* = 0.68 vs. *pol* = 0.13 μmolC/L, bloom start). The flux is proportional to concentration and growth rate, and neither measure alone is a good proxy for the importance of a species [4]. Across all four years, oligotrophic bacteria, defined based on below-average growth rates (literature classifications are often ambiguous), dominate carbon processing for the first 18 days, generally past the phytoplankton peak (Fig. 4C).

The use of *d08* by *s11* and *chr* by *pol* in 2009 suggests are more general pattern, i.e., use of exudation products earlier by oligotrophs and death (i.e., grazing) products later by copiotrophs. Across all years, the fraction of DOM produced by exudation decreases during the course of the bloom (Fig. 4C), a common feature of phytoplankton blooms [33]. This is reflected in the diet of these bacterial groups, i.e., for oligotrophs (vs. copiotrophs), exudates make up a higher portion of the diet (27 vs. 18%), and they have a higher affinity for exudates (39 vs. 35 L/mmolC/d), which is also consistent with experimental evidence from another system [7].

**Fig. 5:**
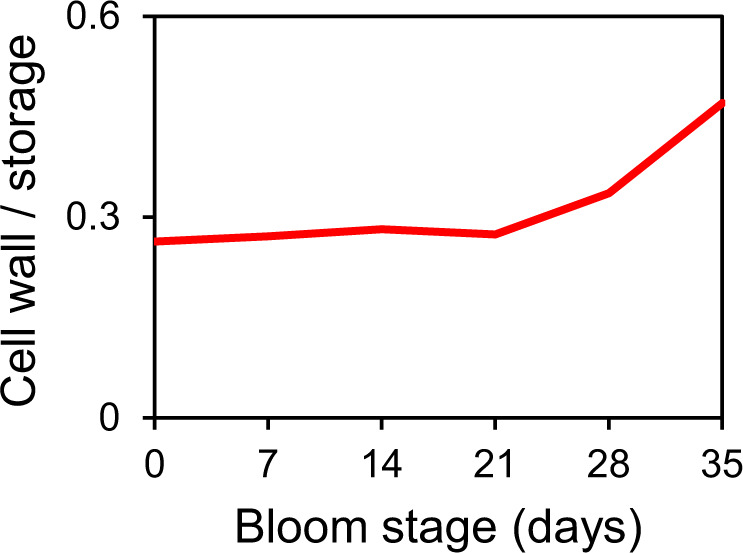
Consumption of cell wall vs. storage compounds during the course of blooms. Total consumption (all bacteria) in µmolC/L/d of cell wall compounds divided by storage compounds. Cell wall compounds = *man* (mannan) + *glo* (glucoromannan) + *fcs* (FCSP). Storage compounds = *chr* (chrysolaminarin) + *gly* (glycogen) + *sta* (starch). Averages for all four years.

After the model was developed, while this paper was in peer review, metaproteomic data for the Helgoland Island spring bloom in 2016 were published that suggest that algal storage compounds (e.g., chrysolaminarin) are used throughout the bloom, whereas cell wall-related compounds (e.g., fucose-containing) are used at later bloom stages [34]. Our model also predicts an increase in the consumption of cell-wall vs. storage compounds at later bloom stages (Fig. 5), which validates our outcomes, although a direct comparison is not possible because of the different time.

### Phytoplankton functional similarity decouples them from bacteria

**Fig. 6:**
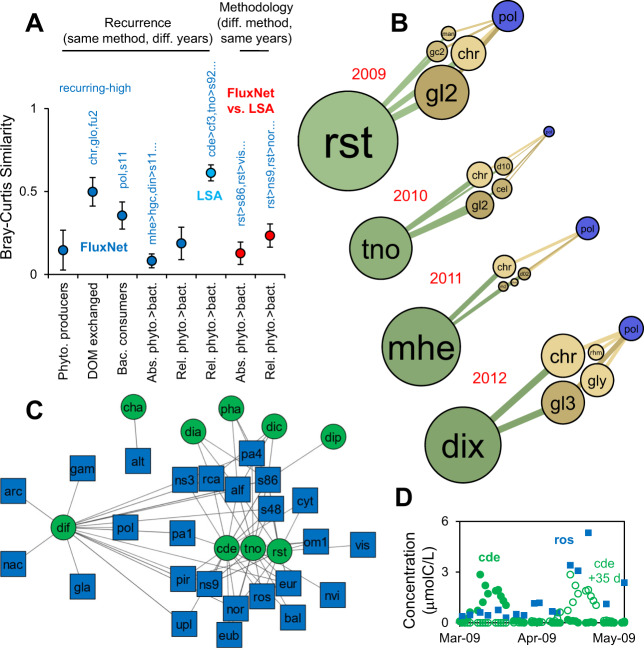
Recurring patterns and comparison of FluxNet and LSA methods. **A** Similarity of networks for spring blooms. Error bars are 95% confidence limits. Bray-Curtis similarity was calculated as 1 – Bray-Curtis dissimilarity. Text on top of symbols lists components that recur in the top quartile every year, listed in order of average rank. **B** Carbon flux networks for top recurring bacterial consumer, top four DOM sources and top coupled phytoplankton. **(C&D)** LSA network (showing top 15% of significant local similarity scores) and sample time series.

An important question is to what extent the patterns recur from year to year [27]. We compare networks of phytoplankton producers, DOM exchanged and bacteria consumers, as well as phytoplankton – bacteria interactions quantified in absolute (μmolC/L/d moving between phytoplankton X to bacteria Y) and relative (% of carbon for bacteria Y supplied by phytoplankton X) terms (Fig. 6A). All networks show significant similarity so there is recurrence from year to year. The recurrence is higher for DOM than phytoplankton, suggesting that different phytoplankton produce similar DOM, which is expected considering similar composition (e.g., *chr* in diatoms). There are no phytoplankton producers that recur in the top quartile every year, but *chr* and others are in the top quartile of DOM exchanged (produced and consumed) every year. The recurrence is lower for bacteria consumers suggesting factors beyond DOM shape the bacteria community.

An important question is how specific interactions are and how tightly networks are interconnected [35, 36], which depends on the mechanisms of interaction and will affect the recurrence. Consistent with the relatively low recurrence of phytoplankton producers, phytoplankton—bacteria coupling shows relatively low recurrence, i.e. low specificity. The primary substrate for the consumer *pol* is mostly *chr* and *gl2*, although it does change from year to year with varying DOM, consistent with the known assimilation capabilities of *pol* (*Polaribacter)* [37] (Fig. 6B). However, the primary associated phytoplankton for *pol* is different each year, although it is always a diatom. The de-coupling of phytoplankton production and bacteria consumption was also concluded from the lower recurrence of phytoplankton and higher recurrence of bacteria abundance in the same dataset [27]. It suggests that carbon processing is resilient to changes in phytoplankton, which may arise from factors like species invasion or climate change.

The above discussion focused on one-way/commensal (phytoplankton > DOM > bacteria) interactions, but the network also includes specific two-way/mutualistic phytoplankton-bacteria interactions. *Phaeocystis* (*pha*) has the highest exudation fraction and Bacteroidetes *nvi* the highest affinity for DOM *d04*, whereas *nvi* has the highest exudation fraction and *pha* the highest requirement for micronutrient *m15*. Such mutualism is observed in other systems and the interactions predicted here can be tested experimentally [20]. Alternatively, experimentally-observed interactions could be used as input to the method, as constraints.

### Robustness of the analysis

**Fig. 7:**
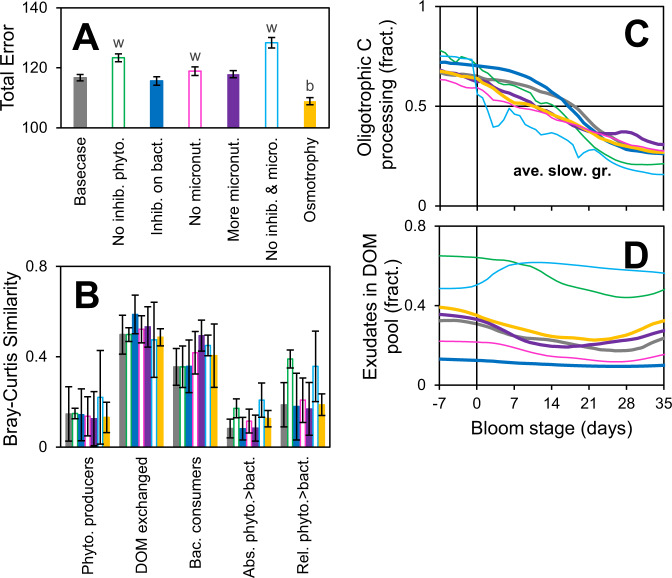
Reproducibility of main results. **A** Total error for runs with different models. “w” or “b” indicates performance is significantly worse (open bars, think lines) or better than basecase, *p* < 0.01, *N* = 128. **B**–**D** Main emerging patterns, as in Fig. 4C and 5A.

To understand the effect of some of the choices made in the model structure we repeated the analysis with added or removed components or processes. Models without micronutrients or inhibitors produce significantly worse fit to the data (Fig. 7A), highlighting the need for a two-way interaction between phytoplankton and bacteria to maintain diversity. Models with more micronutrients or inhibitors are similar to the basecase. Together, these results provide some justification for the complexity (i.e., number of parameters) in the basecase model. The analysis including osmotrophy (aka absorbotrophy, i.e., phytoplankton can perform heterotrophy) produces a better fit to the observations, but that model was not adopted as basecase, because the osmotrophy process is poorly constrained and includes some probably unrealistic features/fluxes, like significant exudation and uptake of the same substance by one phytoplankton species. Importantly, excluding the runs with worse fit to the observations, the main conclusions (as shown in Figs. 4C and 5A) are the same, confirming that the results are reproducible and robust to some of the choices made in model structure.

### Empirical method shows limitations

We also analyze the dataset using the empirical LSA method [38], which identifies many of the same interactions. For the spring 2009 bloom, the *rst* > *ns9* interaction ranks in the top 1% for LSA and FluxNet (relative interaction). However, the lack of mechanistic constraints is evident. One of the strongest links for the 2009 spring bloom (rank 13%) is between the diatom *Chaetoceros debilis* (*cde*) and *Roseobacters* (*ros*) (Fig. 6C, D). The shifted peaks line up nicely, but the bacteria biomass is higher than that of the phytoplankton and genome analysis suggests *ros* do not assimilate chrysolaminarin [37], which is a major death product of diatoms. Considering this, growth yield and other competing consumers, it is unlikely that *cde* is a major source of carbon to *ros*.

### Summary and outlook

Modern observational tools are generating high-resolution descriptions of the components of microbial ecosystems, and an ongoing grand challenge is to use these data to understand how systems function. Our method predicts dynamic mass fluxes between marine phytoplankton and bacteria, which provides insights into the functioning of the ecosystem. Specifically, it showed that there is a strong correlation between concentration and flux of bacteria during blooms, but oligotrophs are relatively less important than copiotrophs. However, due to their higher biomass, they are major carbon processors during early phases of blooms, well past the peak. Oligotrophs grow preferentially on exudation products, which are more abundant earlier in the bloom. Also, our results suggest that phytoplankton are functionally similar in terms of what organic carbon species they produce, and that this decouples them from bacteria.

FluxNet is an inference method for microbial time series data that serves the same general purpose as existing empirical inference methods, like LSA [38]. In general, both approaches have strengths and weaknesses (see Introduction) and may complement each other. A main advantage of FluxNet is that it produces quantitative concentrations and fluxes, and associated conclusions (e.g., preferential use of exudates by oligotrophs). Also, it is constrained by mass balance and additional information from the literature (i.e., beyond the time series data), which make the results more realistic.

The existing FluxNet code can readily be applied at a higher resolution (microdiversity), explicit representation of other ecosystem components, like viruses and zooplankton, and more processes, like photoheterotrophy and mixotrophy. It may also be applied to understand other microbial ecosystems, like the human gut or wastewater treatment plants. For an inference method it is important to be applicable to various types of observations, including modern environmental -omics observations, like transcript, protein and metabolite levels, and the present model will have to evolve in this direction [39]. The present model includes a relatively simple representation of the various processes, and the current biological understanding supports increasing the mechanistic realism (and complexity). For example, the present version assumes constant composition of DOM produced by phytoplankton, but observations show that it changes with physiology and interaction with bacteria [18, 40]. Also, the model assumes simple first-order dissolution of POM to DOM and direct utilization by bacteria, whereas break-down of especially polysaccharides is often mediated by extracellular enzymes [41].

## Methods

A complete description of the methods is presented in the SI. At the beginning of the Results and discussion we provide a brief overview for all readers. Below we give some additional details about the mechanistic microbial ecosystem model and optimization method for modeling specialists.

### Mechanistic microbial ecosystem model

The modeling concepts and equations are generally based on past models of phytoplankton and bacteria [13, 14, 23, 42, 43]. Novel aspects include consideration of dormancy, phytoplankton micronutrient limitation and inhibitors.

To illustrate the approach, we present mass balance equations for a reduced system consisting of one phytoplankton (*rst*), two DOM (*chr*, *gl2*) and one bacteria (*pol*), and no micronutrients or inhibitors. Processes affecting phytoplankton concentration include photosynthesis, respiration, exudation, inhibition, death, settling and outflow. The mass balance equation for *rst*, excluding inhibition loss, is:

1 $$\frac{d}{{dt}}C_{rst} =	\; kp_{rst}Lp_{rst}C_{rst} - kr_{rst}Lr_{rst}C_{rst} - \left( {ke_{rst} + ef_{rst}kp_{rst}Lp_{rst}} \right)\ C_{rst} \\ 	- ku_{rst}Lu_{rst}C_{rst} - \frac{{vs_{rst}}}{H}C_{rst} - \frac{Q}{V}C_{rst}$$

*t* (d) = time, *C* (mmolC/L) = concentration, *kp* (1/d) = max. photosynthesis rate constant, *Lp* = limitation/modification factor for photosynthesis (light, nutrients, temperature, salinity), *kr* (1/d) = max. respiration rate constant, *Lr* = limitation/modification factor for respiration (temperature), *ke* (1/d) = basal exudation rate constant, *ef* (1/d) = exudation/photosynthesis fraction, *ku* (1/d) = maximum death rate, *Lu* = limitation/modification factor for death (time-of-year, salinity), vs (m/d) = settling velocity, *H* (m) = water column depth, *Q* (m^3^/d) = flow rate, *V* (m^3^) = volume.

Processes affecting DOM include microbial exudation and death, POM dissolution, heterotrophy and outflow. The mass balance equation for *chr*, considering only *rst* as source, is:

2 $$\frac{d}{{dt}}C_{chr} =	\; Fe_{rst,chr}\left( {ke_{rst} + ef_{rst}kp_{rst}Lp_{rst}} \right)C_{rst} + Fx_{rst,chr}ku_{rst}Lu_{rst}\ C_{rst} + kf_{phr}Lf_{phr}C_{phr} \\ 	- kh_{pol}\frac{{C_{chr}/Ksh_{pol,chr}}}{{1 + C_{chr}/Ksh_{pol,chr} + C_{gl2}/Ksh_{pol,gl2}}}Lh_{pol}C_{pol} - \frac{Q}{V}C_{chr}$$

*Fe* = exudation fraction, *Fx* = composition fraction, *kf* (1/d) = dissolution rate constant, *Lf* = limitation/modification factor for dissolution (temperature), *kh* (1/d) = max. heterotrophy rate, *Ksh* (mmolC/L) = half-saturation constant, *Lh* = limitation/modification factor for heterotrophy (temperature, salinity, light).

Processes affecting bacteria concentration include heterotrophy, death and outflow. The mass balance equation for *pol*, considering only growth on *chr* and *gl2*, is:

3 $$\frac{d}{{dt}}C_{pol} =	\; Yh_{pol}kh_{pol}\frac{{C_{chr}/Ksh_{pol,chr} + C_{gl2}/Ksh_{pol,gl2}}}{{1 + C_{chr}/Ksh_{pol,chr} + C_{gl2}/Ksh_{pol,gl2}}}Lh_{pol}\ C_{pol} \\ 	- ku_{pol}Lu_{pol}C_{pol} - \frac{Q}{V}C_{pol}$$

*Yh* = yield coefficient.

Dormancy is modeled by specifying a floor concentration (*Cflr*) and reducing any loss rate that would result in concentration below this value, an approach similar to previous models [26]. Micronutrients are exuded by bacteria and limit photosynthesis of phytoplankton, which is modeled analogous to macronutrient limitation, using a Monod limitation term for each micronutrient that are combined using a minimum formulation. Inhibitors are produced by phytoplankton and bacteria via exudation, and they kill phytoplankton or bacteria in a concentration-dependent manner, also using a Monod saturation term.

### Optimization method

The optimization method adjusts parameter values within literature ranges to minimize the disagreement i.e. error between model and data, which includes both bulk/summary (e.g., Chlorophyll *a*, gammaproteobacteria) and more specific (e.g., *Rhizosolenia styliformis*, SAR11) observations. The method generally follows previous numerical optimization approaches for microbial ecosystem models [13, 14, 23]. Novel aspects include a two-dimensional (concentration and time) quantification of model-data disagreement, numerical optimization methods customized for microbial ecosystems and gradual increase in model complexity (de-lumping).

To account for disagreement between model and data in the concentration and time dimensions (e.g. a temporal offset) [43], the discrepancy between an individual data point and the model is quantified as the minimum error square (*ES*) in two-dimensional space:

4 $$ES = \min \left[ {\left( {C_d - C_{m,k}} \right)^2 + \left( {\left[ {t_d - t_{m,k}} \right]ave\left[ {C_d,C_m} \right]k_{ch}} \right)^2} \right]$$

*C*_*d*_ = data value, *C*_*m*_ = model value, *t*_*d*_ = data time, *t*_*m*_ = model time, *k*_*ch*_ (1/d) = first-order rate constant relating the value and time dimensions, *k* = index for the model point. Error values for individual data points are combined as a normalized root mean squared error, and then weighted and summed across data series into a total error. In previous optimizations of ecosystem models, the error was quantified in arithmetic or log space [13, 14, 23]. Here we use arithmetic space and also present our model-data comparison that way (e.g., Fig. 2), because we are interested in the higher fluxes, which presumably are associated with the higher concentrations.

The optimization problem is characterized by a large number of dependent parameters and local minima in the objective function. The routine performs a number of iterations until a convergence criterion is reached. Each iteration includes single-parameter optimization on the entire parameter set. Following that is single-parameter optimization on a smaller parameter set identified as most sensitive in the prior complete single-parameter optimization. Then, the method performs multi-parameter optimization on subsets of parameters identified *a priori* as dependent, like the max. photosynthesis and respiration rates within a component or the Chlorophyll *a* content across components. Monte Carlo/Latin Hypercube Sampling is performed, steps are repeated using different sets of random numbers and several instances are run in parallel (typically 128, based on distribution of final errors, see Fig. S3) on a cluster, to decrease the chances of getting stuck in a local minima in the objective function.

## Supplementary Information

Movie S1

Figure S1

Supplementary Information

Dataset S1

## Data Availability

The code and input will be made available on the corresponding author’s GitHub page (https://github.com/fhellweger).
