# Supplementary material for: Dynamic carbon flux network of a diverse marine microbial community
Source: ISME Commun. 2021 Sep 25;1:50. doi: 10.1038/s43705-021-00055-7 (PMC9723560; doi:10.1038/s43705-021-00055-7)
Supplement: Supplementary file 2 — Figure S1 [file 43705_2021_55_MOESM2_ESM.pdf]

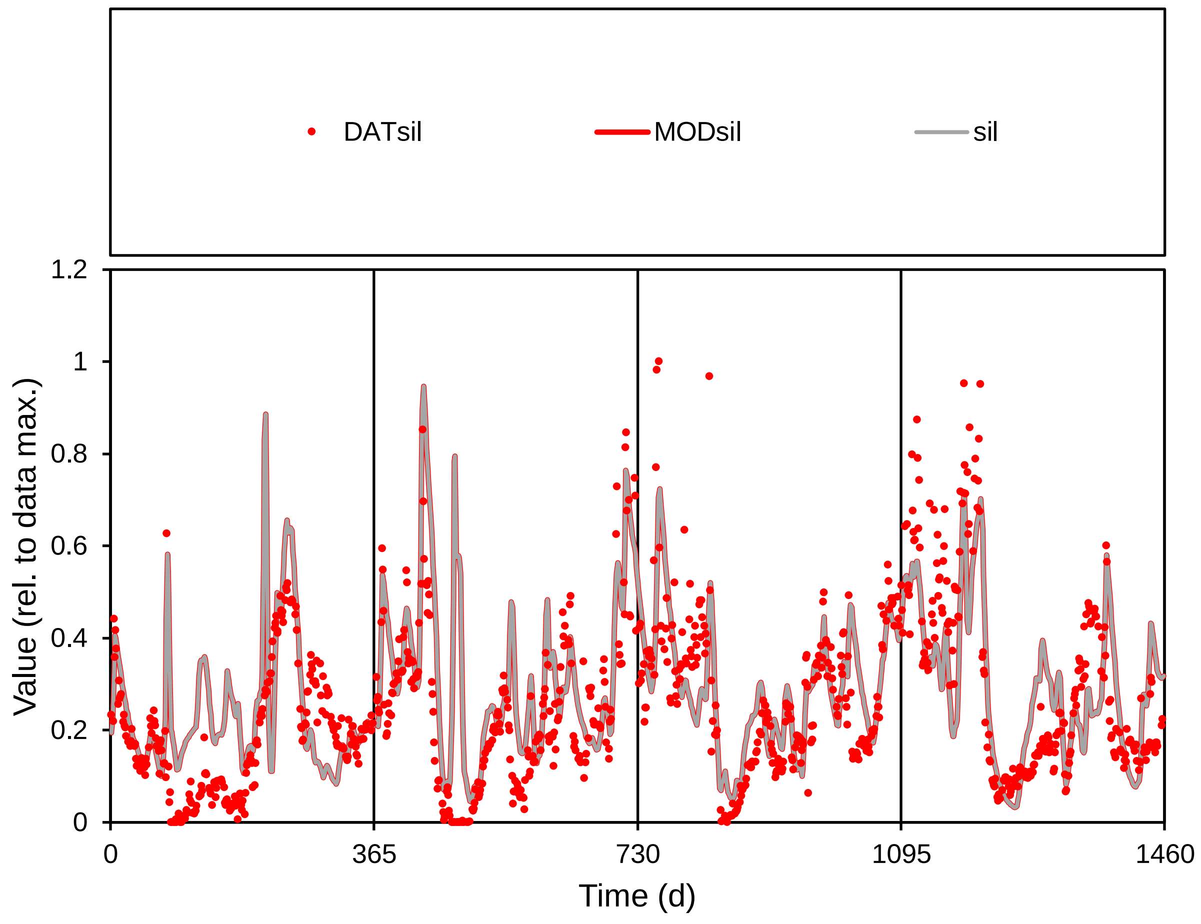

Figure S1(1). Model - data comparison for observation: sil
Normalized to max. value of data. Symbols are observations and lines are model. Red line corresponds to observations (e.g. Chlorophyll *a*), others are sub-components (e.g. individual phytoplankton species). See Tables S19 and S24 for observation and model component IDs, and mapping.

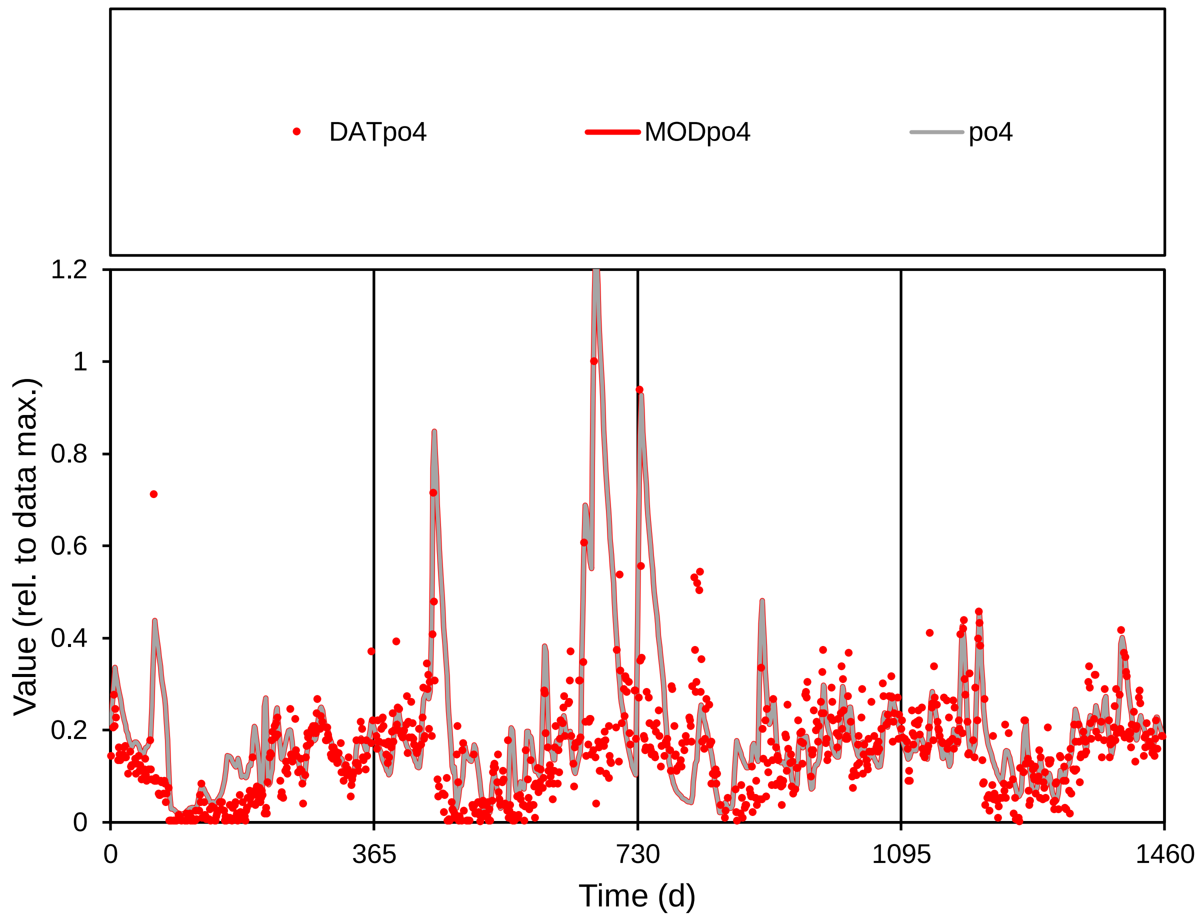

Figure S1(2). Model - data comparison for observation: po4
Normalized to max. value of data. Symbols are observations and lines are model. Red line corresponds to observations (e.g. Chlorophyll *a*), others are sub-components (e.g. individual phytoplankton species). See Tables S19 and S24 for observation and model component IDs, and mapping.

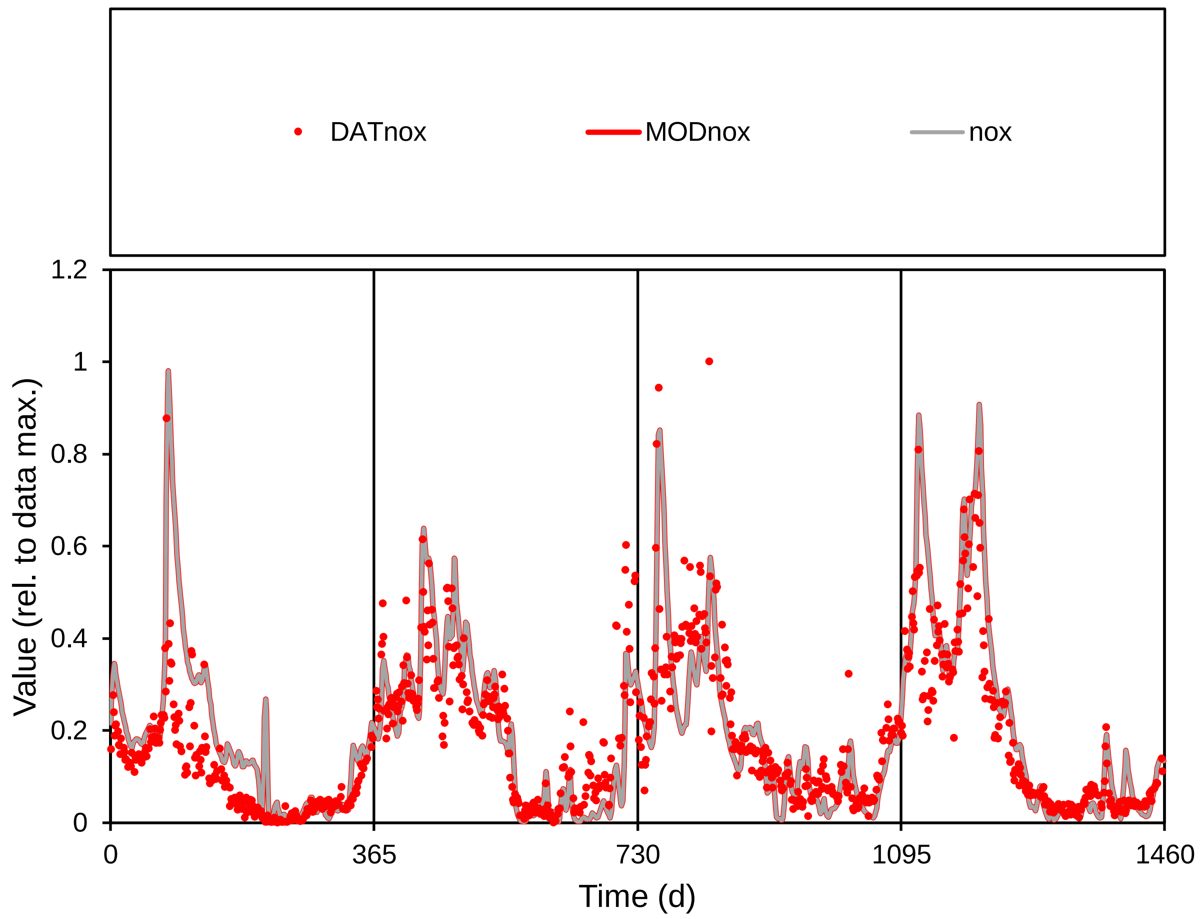

Figure S1(3). Model - data comparison for observation: nox
Normalized to max. value of data. Symbols are observations and lines are model. Red line corresponds to observations (e.g. Chlorophyll *a*), others are sub-components (e.g. individual phytoplankton species). See Tables S19 and S24 for observation and model component IDs, and mapping.

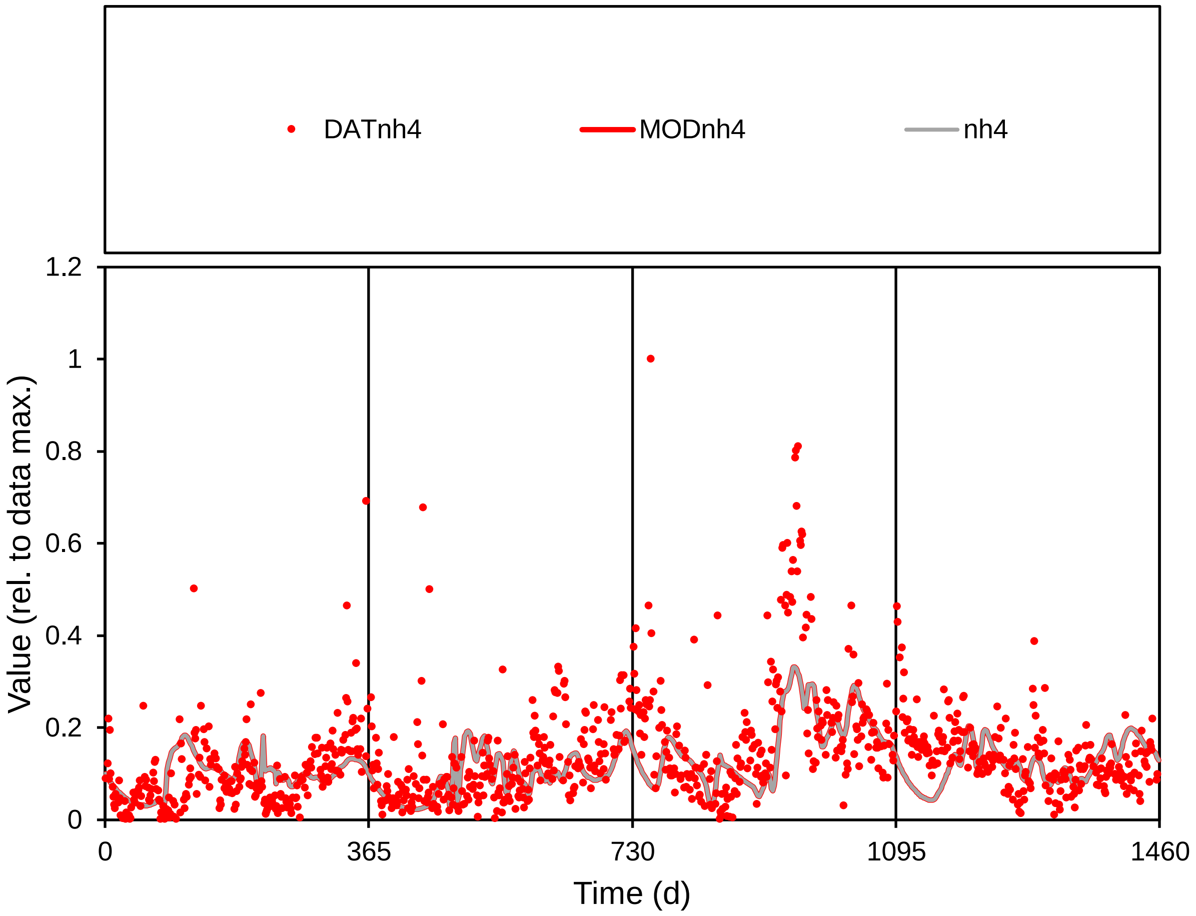

Figure S1(4). Model - data comparison for observation: nh4
Normalized to max. value of data. Symbols are observations and lines are model. Red line corresponds to observations (e.g. Chlorophyll *a*), others are sub-components (e.g. individual phytoplankton species). See Tables S19 and S24 for observation and model component IDs, and mapping.

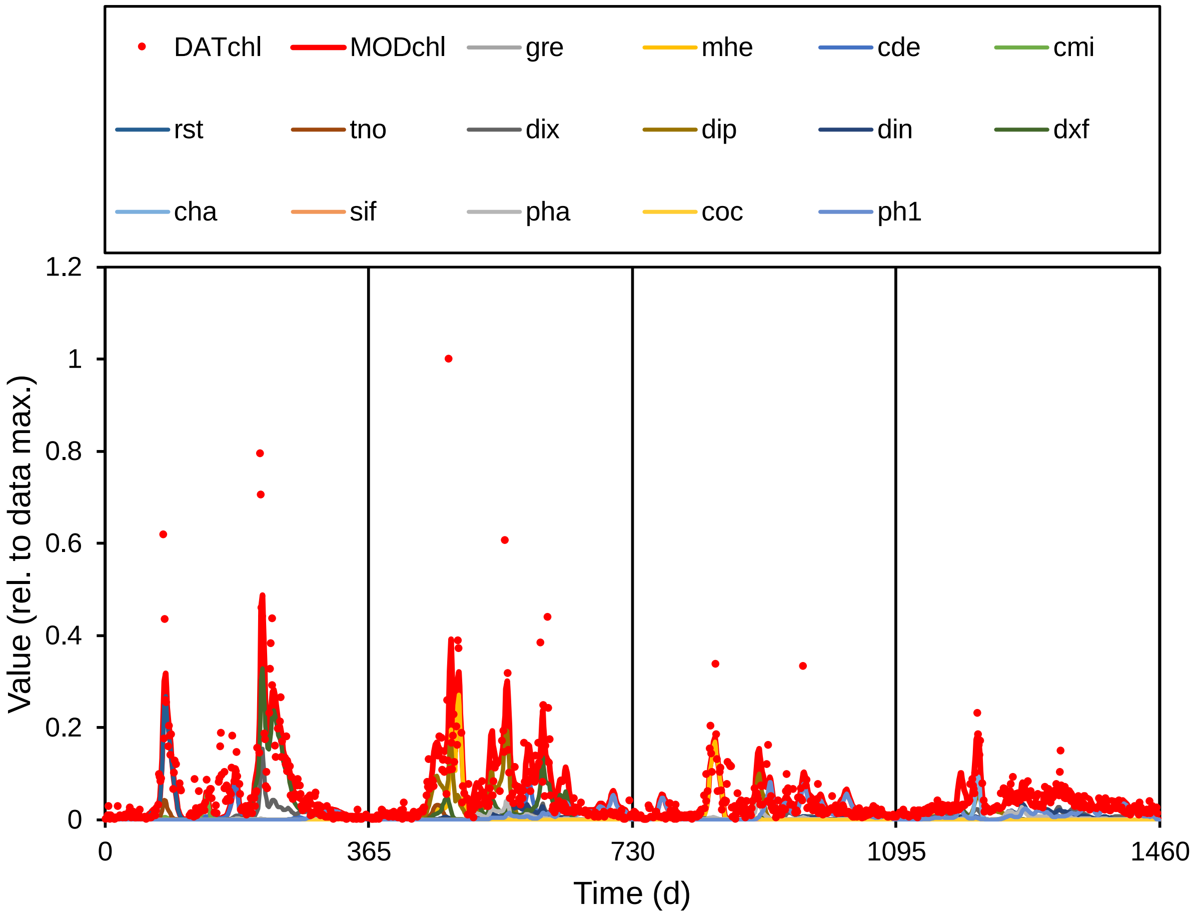

Figure S1(5). Model - data comparison for observation: chl
Normalized to max. value of data. Symbols are observations and lines are model. Red line corresponds to observations (e.g. Chlorophyll *a*), others are sub-components (e.g. individual phytoplankton species). See Tables S19 and S24 for observation and model component IDs, and mapping.

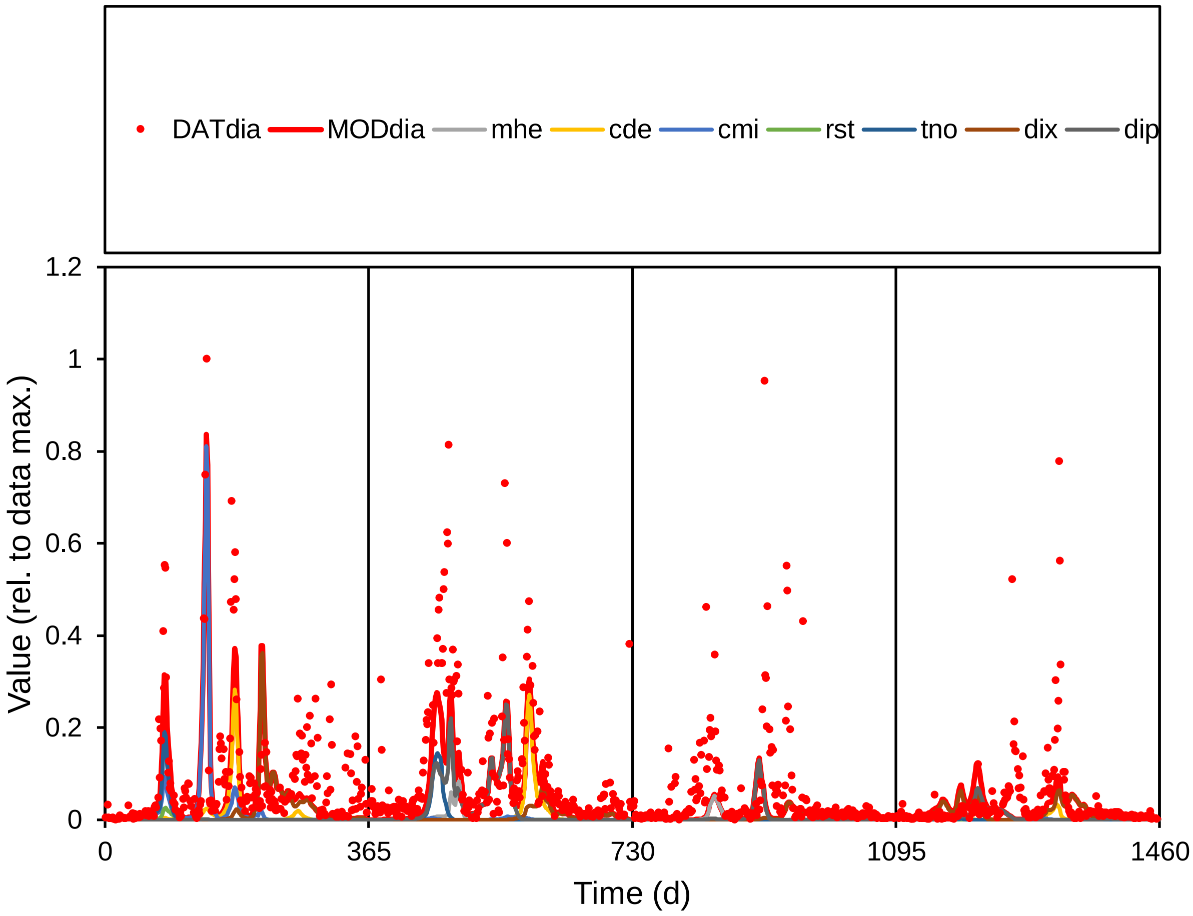

Figure S1(6). Model - data comparison for observation: dia
Normalized to max. value of data. Symbols are observations and lines are model. Red line corresponds to observations (e.g. Chlorophyll *a*), others are sub-components (e.g. individual phytoplankton species). See Tables S19 and S24 for observation and model component IDs, and mapping.

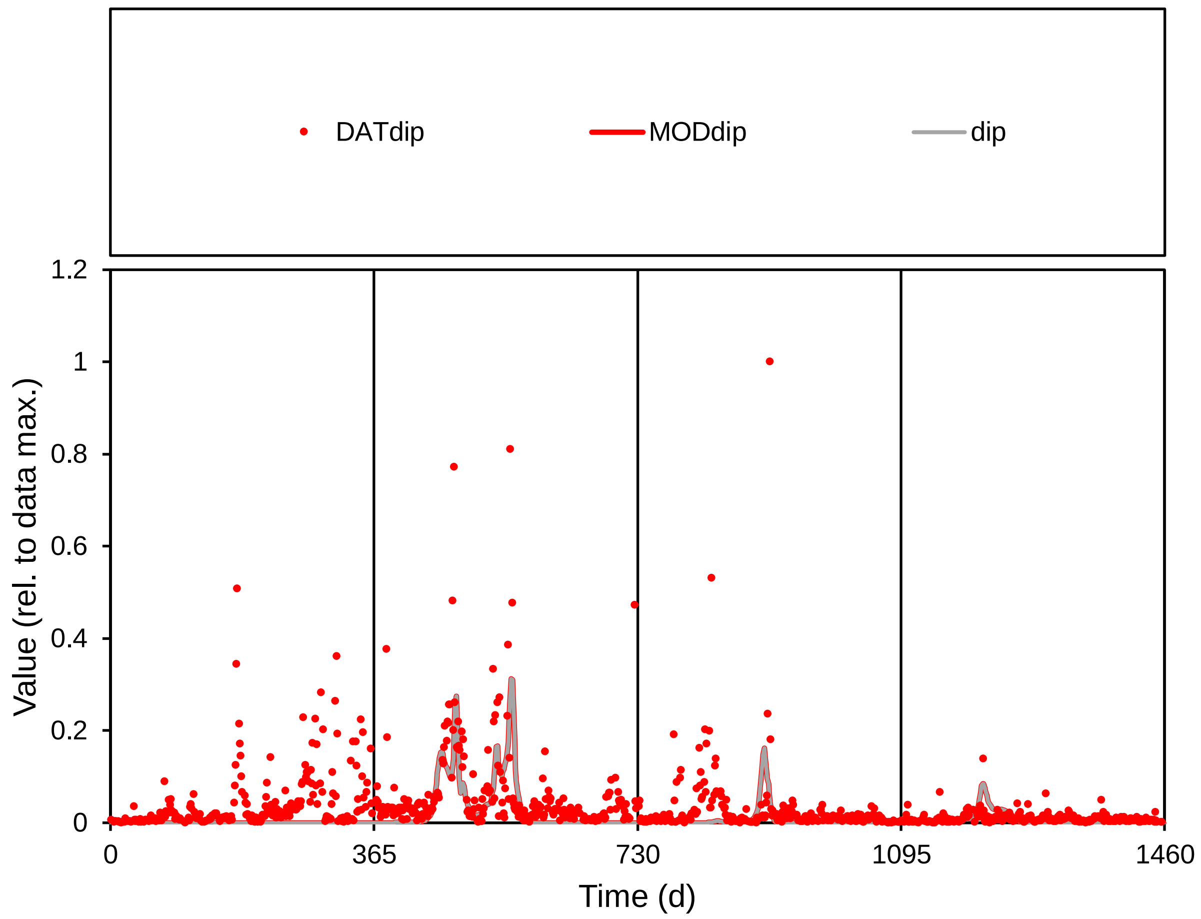

Figure S1(7). Model - data comparison for observation: dip
Normalized to max. value of data. Symbols are observations and lines are model. Red line corresponds to observations (e.g. Chlorophyll *a*), others are sub-components (e.g. individual phytoplankton species). See Tables S19 and S24 for observation and model component IDs, and mapping.

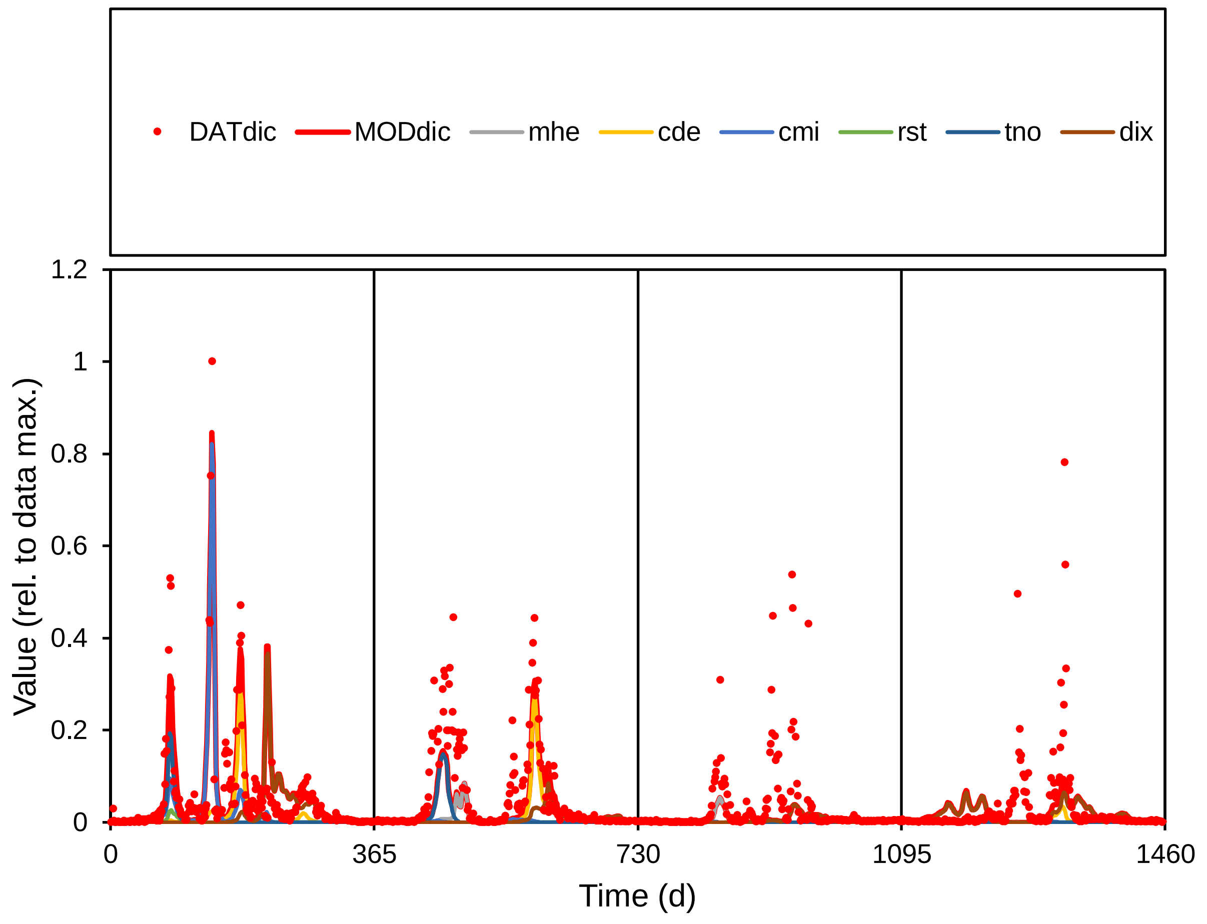

Figure S1(8). Model - data comparison for observation: dic
Normalized to max. value of data. Symbols are observations and lines are model. Red line corresponds to observations (e.g. Chlorophyll *a*), others are sub-components (e.g. individual phytoplankton species). See Tables S19 and S24 for observation and model component IDs, and mapping.

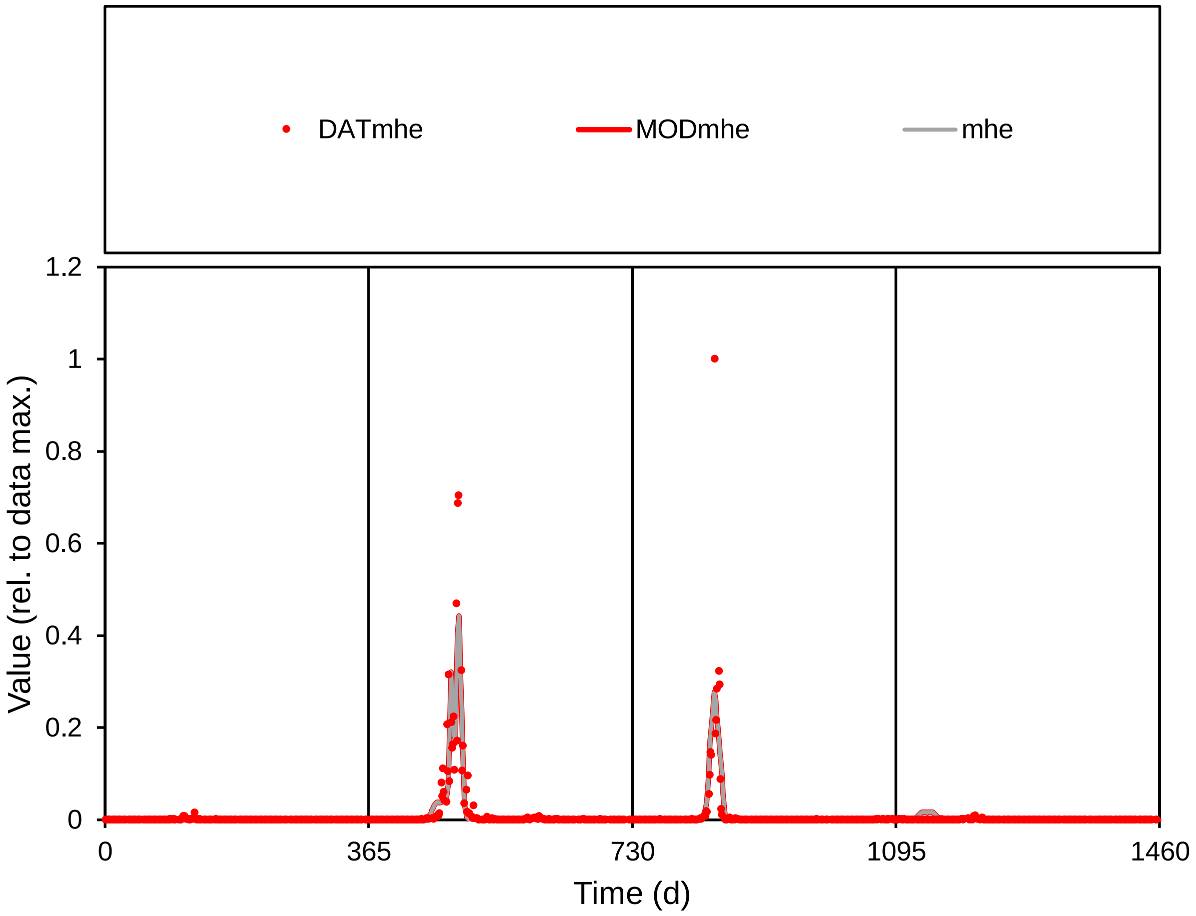

Figure S1(9). Model - data comparison for observation: mhe
Normalized to max. value of data. Symbols are observations and lines are model. Red line corresponds to observations (e.g. Chlorophyll *a*), others are sub-components (e.g. individual phytoplankton species). See Tables S19 and S24 for observation and model component IDs, and mapping.

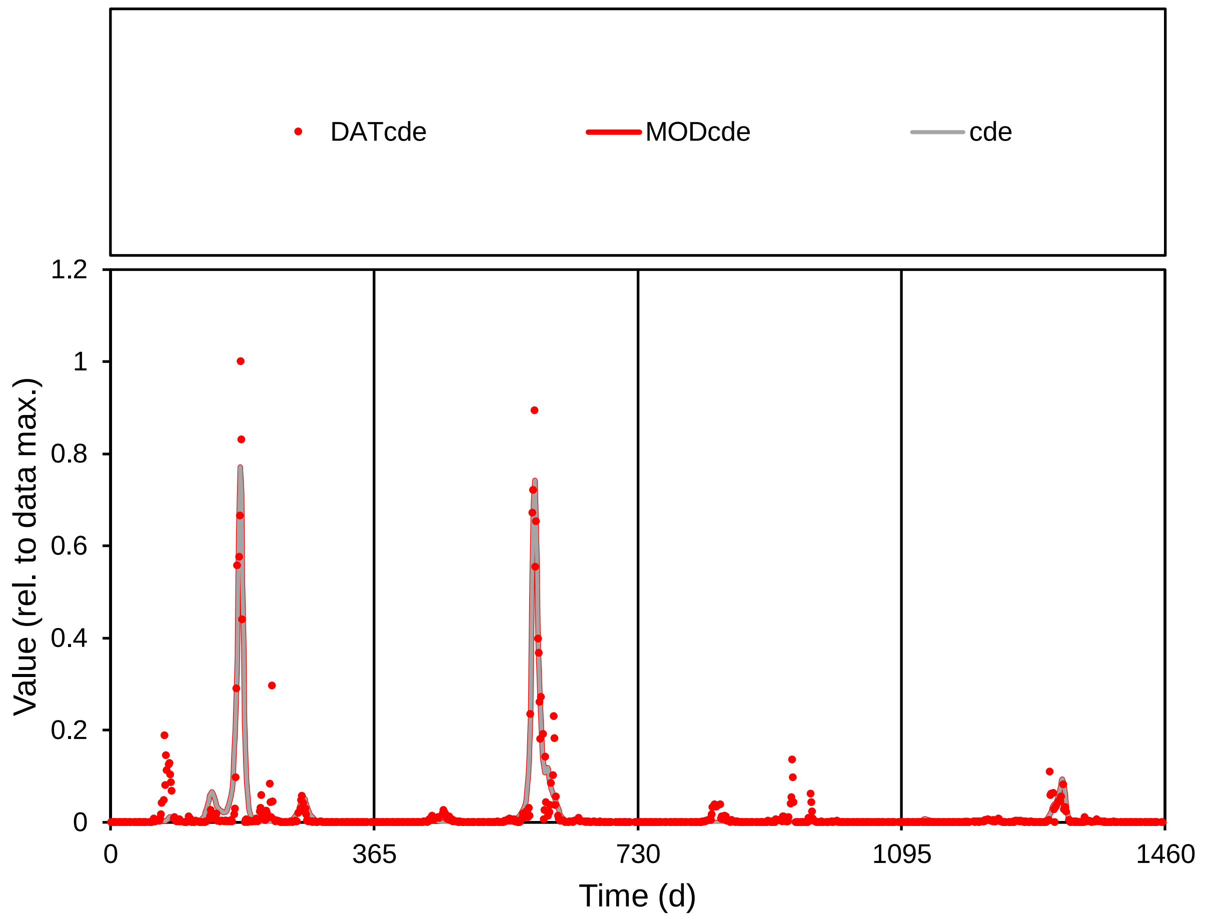

Figure S1(10). Model - data comparison for observation: cde
Normalized to max. value of data. Symbols are observations and lines are model. Red line corresponds to observations (e.g. Chlorophyll *a*), others are sub-components (e.g. individual phytoplankton species). See Tables S19 and S24 for observation and model component IDs, and mapping.

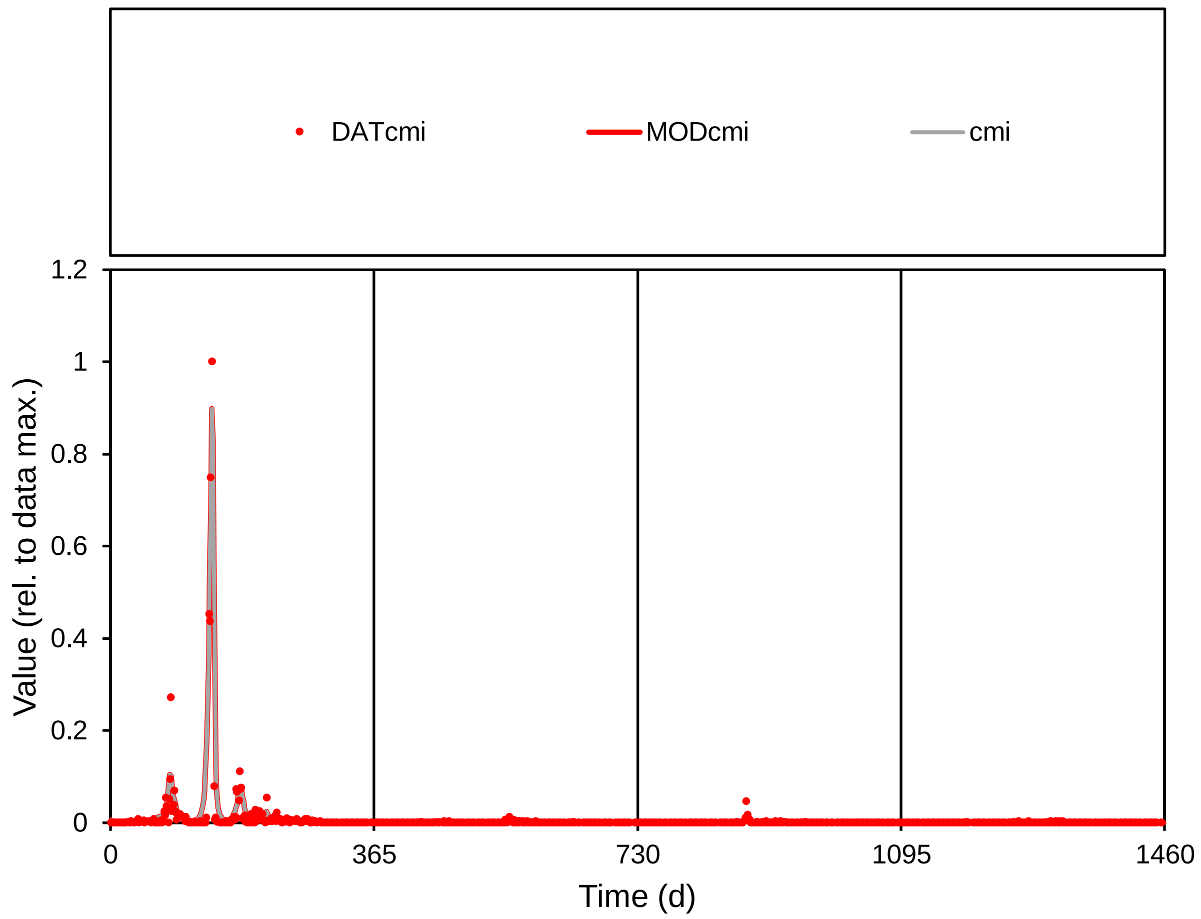

Figure S1(11). Model - data comparison for observation: cmi
Normalized to max. value of data. Symbols are observations and lines are model. Red line corresponds to observations (e.g. Chlorophyll *a*), others are sub-components (e.g. individual phytoplankton species). See Tables S19 and S24 for observation and model component IDs, and mapping.

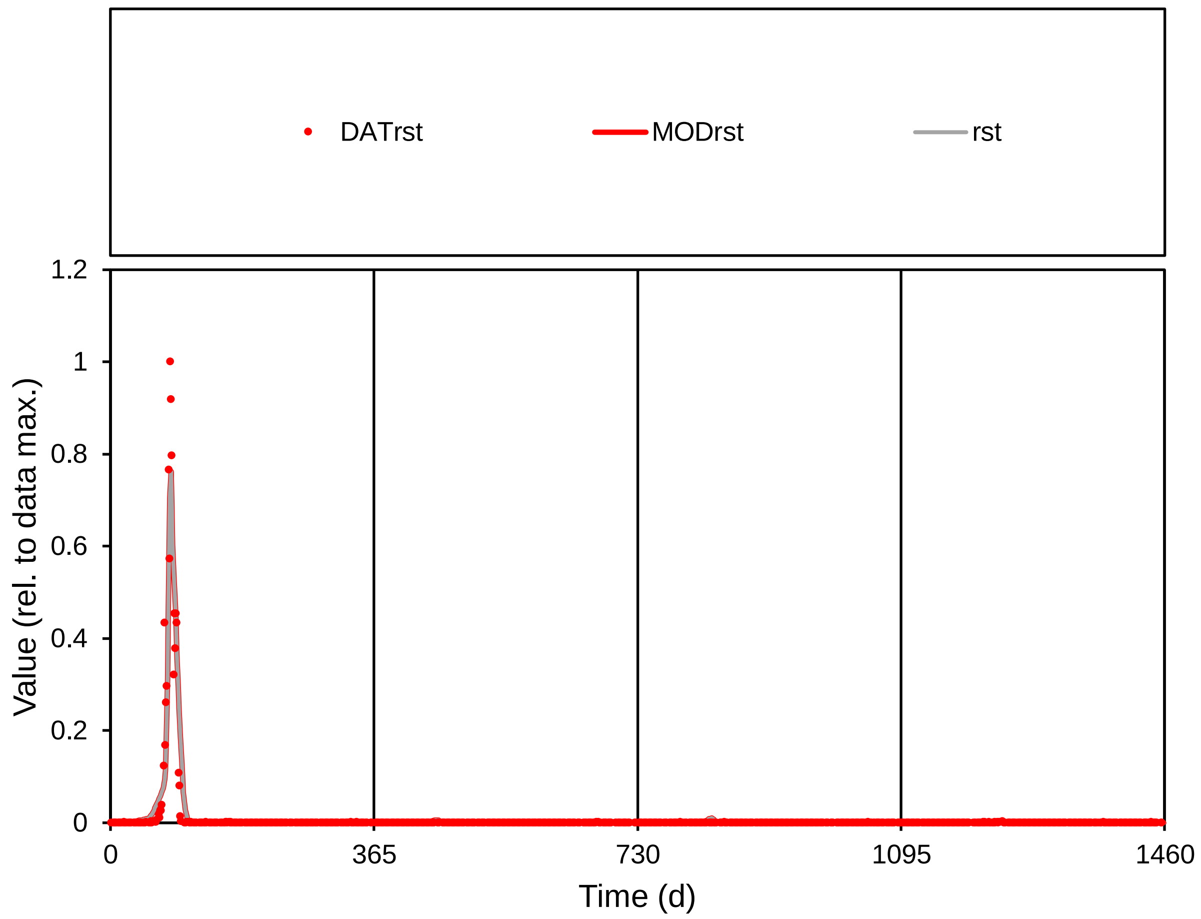

Figure S1(12). Model - data comparison for observation: rst
Normalized to max. value of data. Symbols are observations and lines are model. Red line corresponds to observations (e.g. Chlorophyll *a*), others are sub-components (e.g. individual phytoplankton species). See Tables S19 and S24 for observation and model component IDs, and mapping.

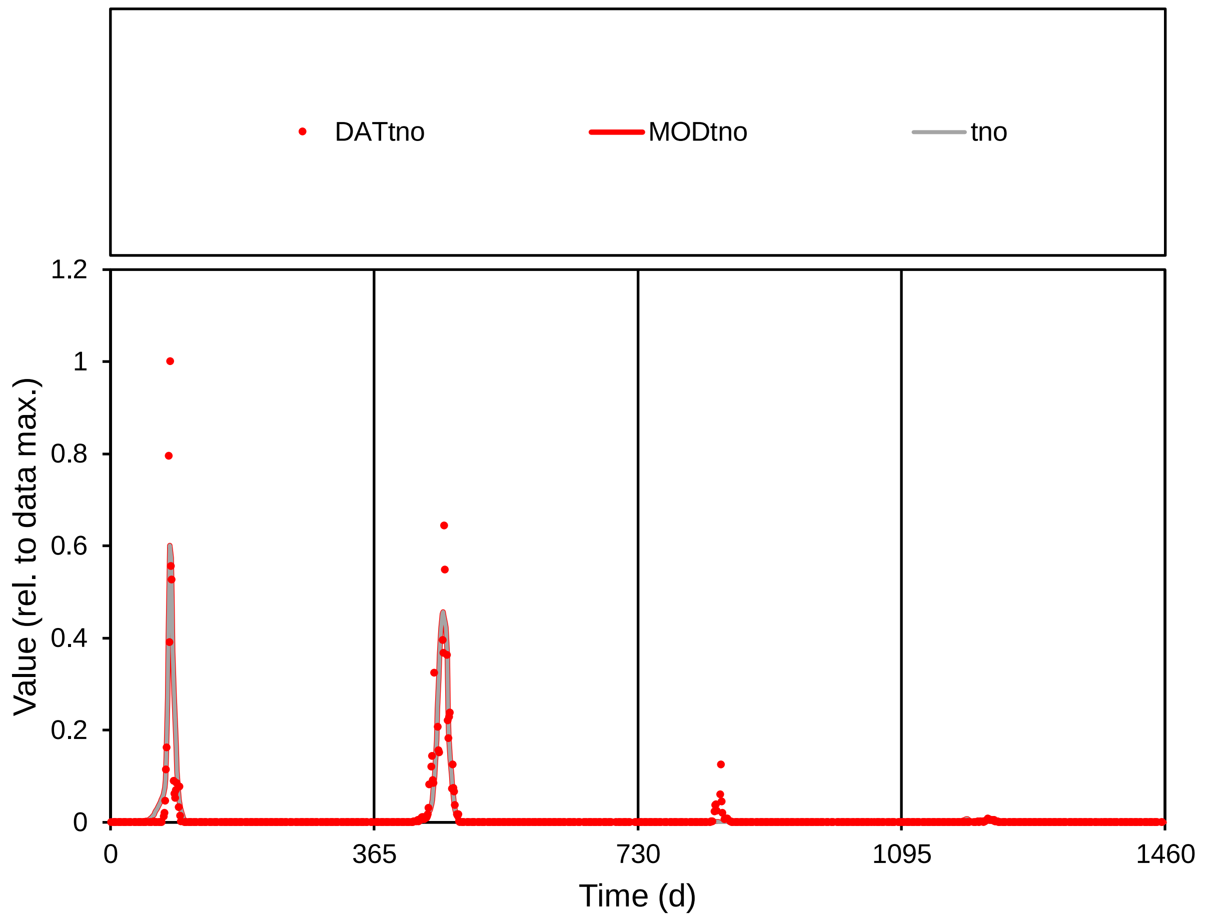

Figure S1(13). Model - data comparison for observation: tno
Normalized to max. value of data. Symbols are observations and lines are model. Red line corresponds to observations (e.g. Chlorophyll *a*), others are sub-components (e.g. individual phytoplankton species). See Tables S19 and S24 for observation and model component IDs, and mapping.

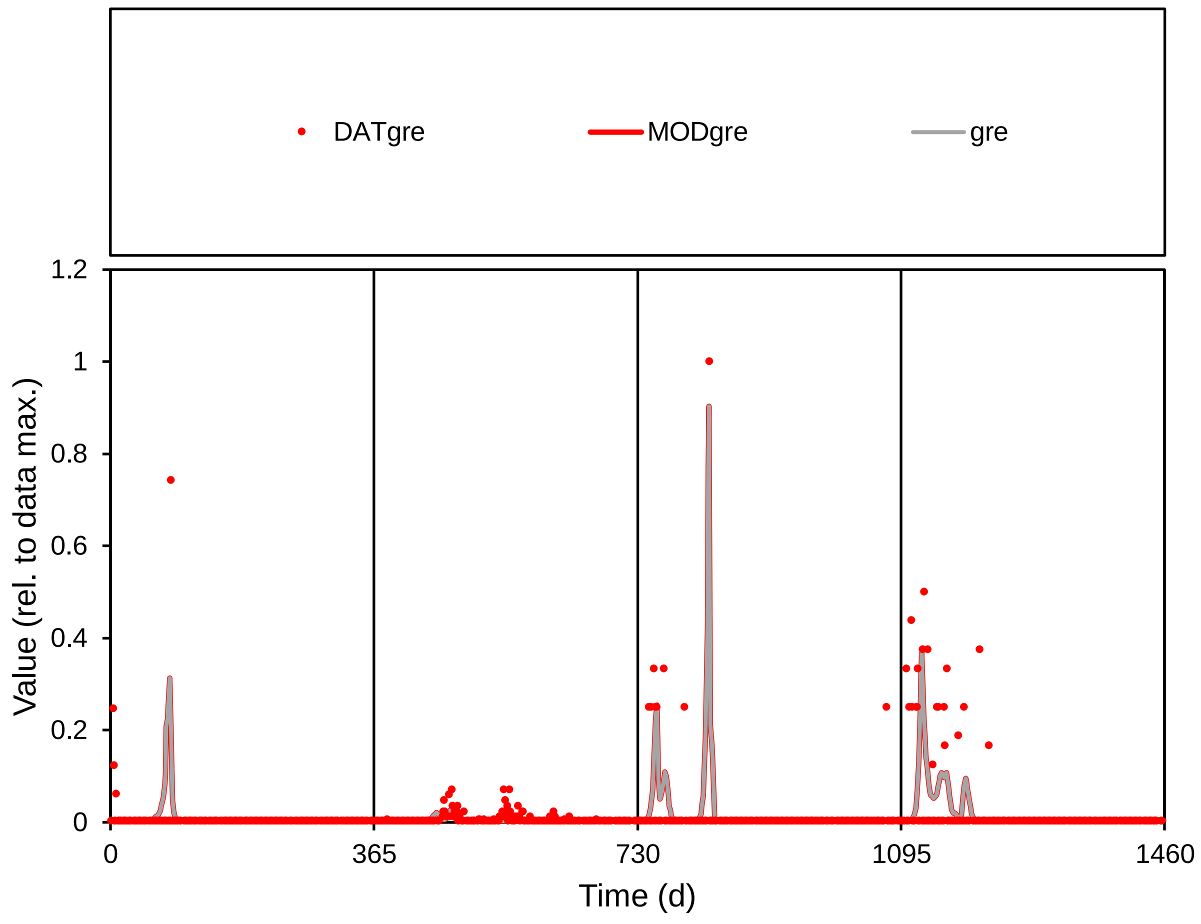

Figure S1(14). Model - data comparison for observation: gre
Normalized to max. value of data. Symbols are observations and lines are model. Red line corresponds to observations (e.g. Chlorophyll *a*), others are sub-components (e.g. individual phytoplankton species). See Tables S19 and S24 for observation and model component IDs, and mapping.

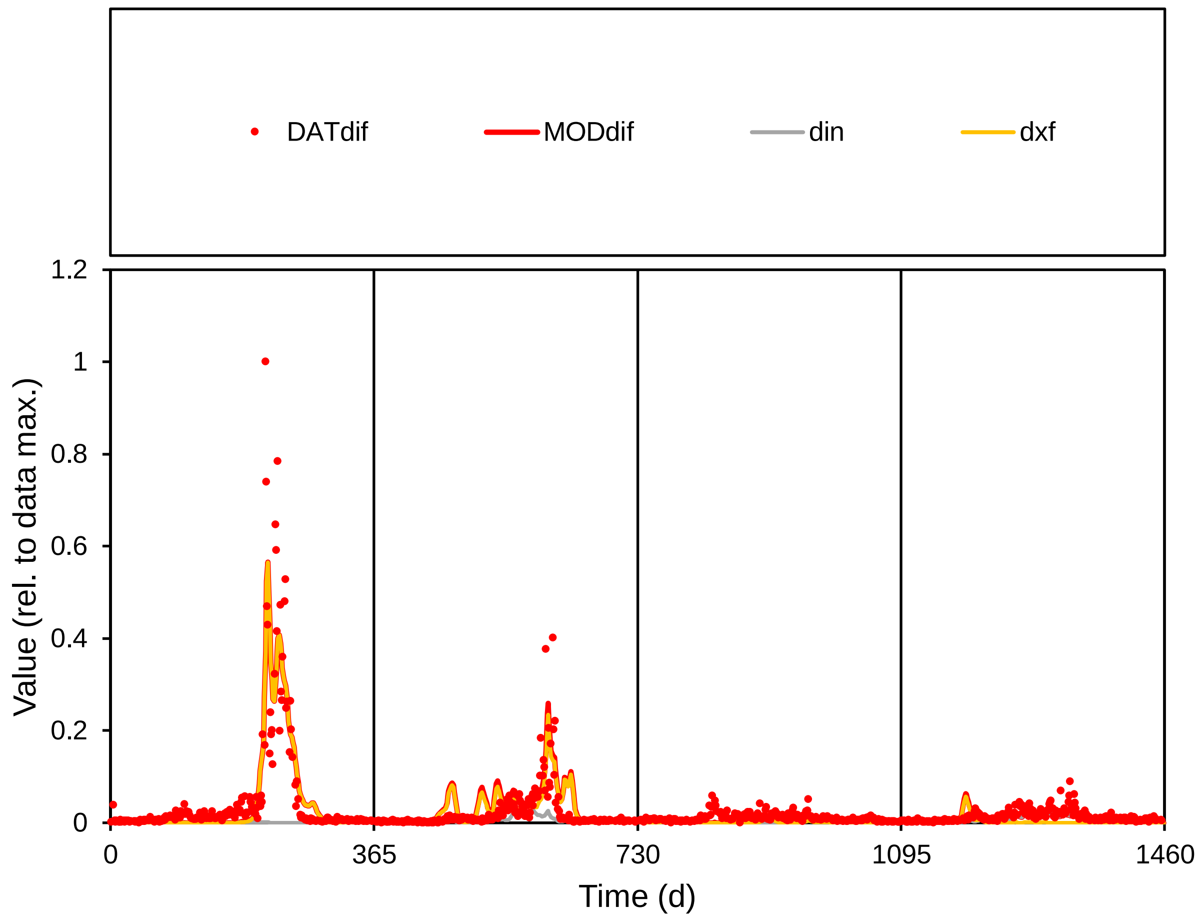

Figure S1(15). Model - data comparison for observation: dif
Normalized to max. value of data. Symbols are observations and lines are model. Red line corresponds to observations (e.g. Chlorophyll *a*), others are sub-components (e.g. individual phytoplankton species). See Tables S19 and S24 for observation and model component IDs, and mapping.

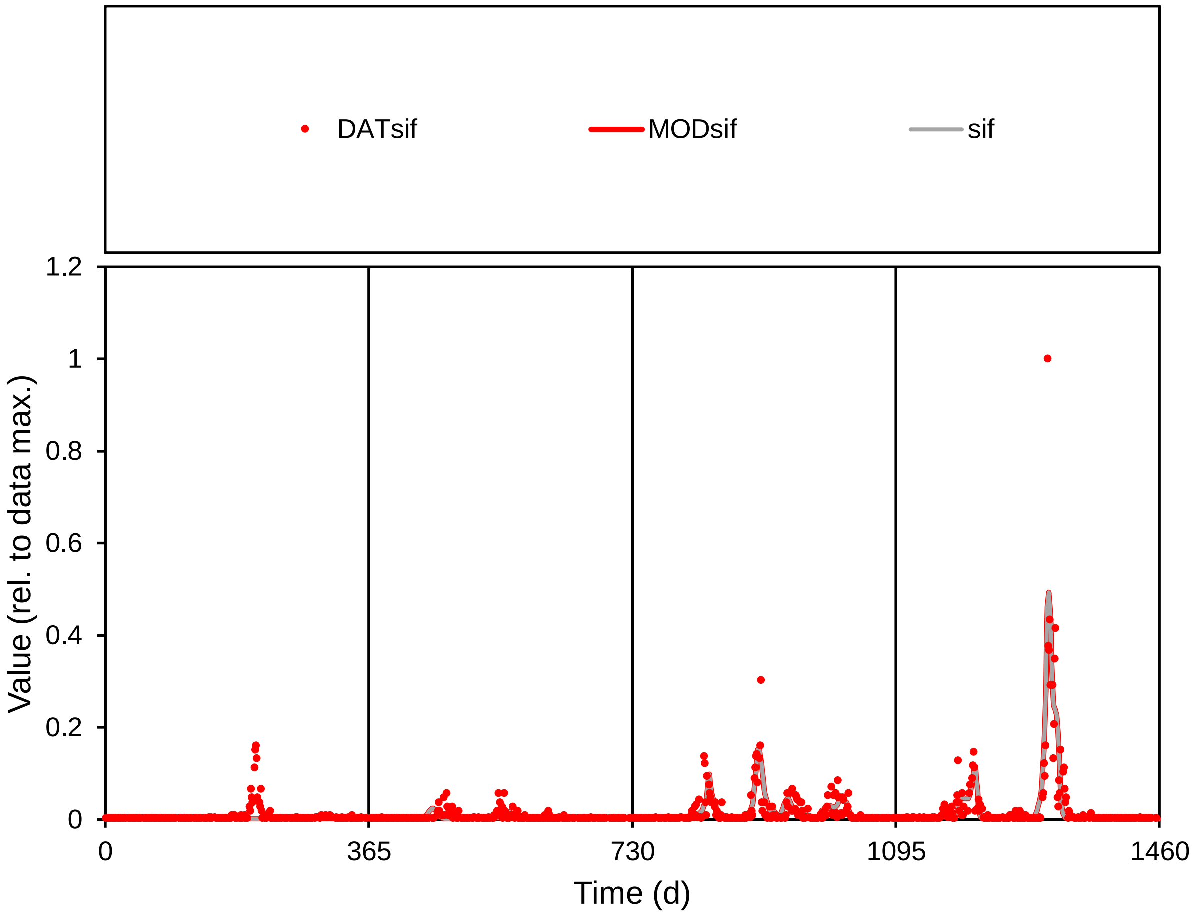

Figure S1(16). Model - data comparison for observation: sif
Normalized to max. value of data. Symbols are observations and lines are model. Red line corresponds to observations (e.g. Chlorophyll *a*), others are sub-components (e.g. individual phytoplankton species). See Tables S19 and S24 for observation and model component IDs, and mapping.

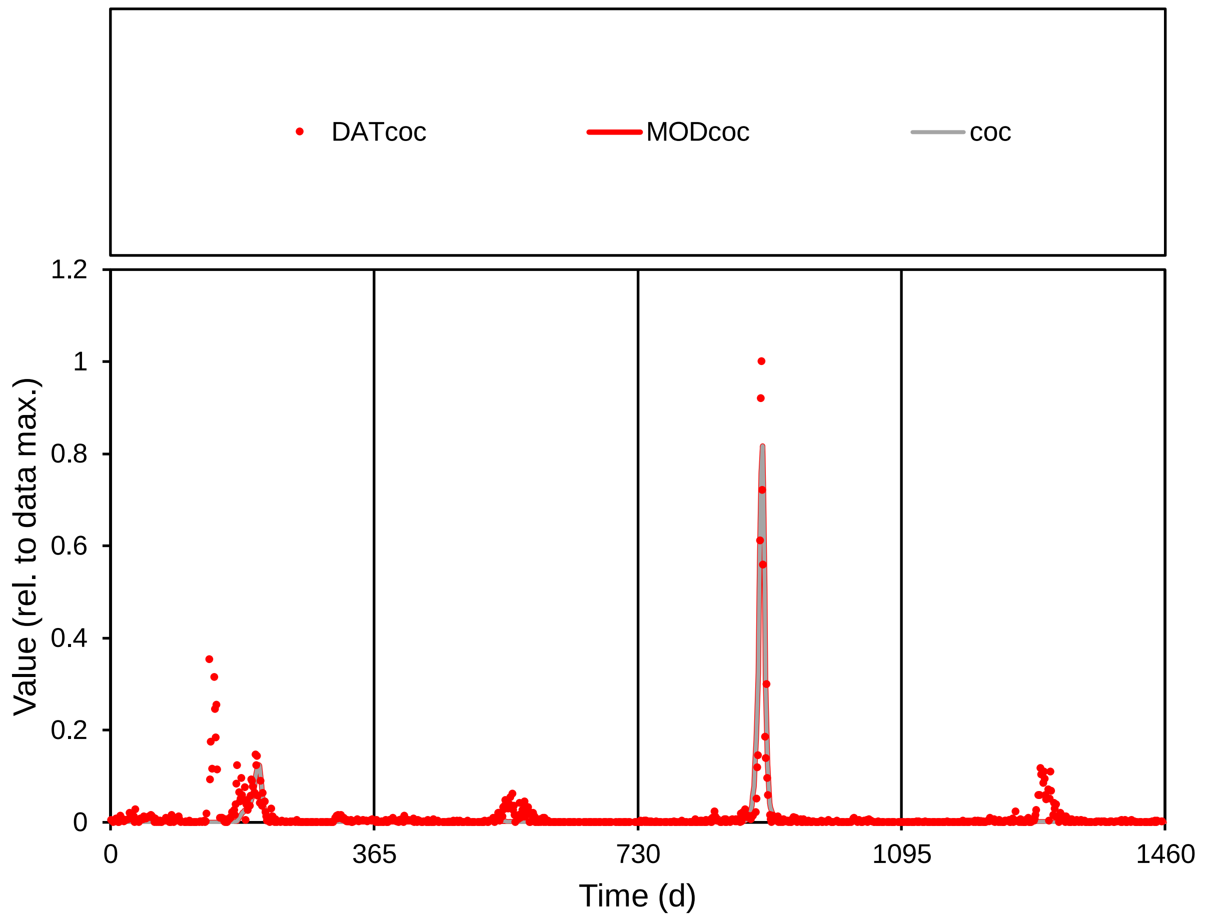

Figure S1(17). Model - data comparison for observation: coc
Normalized to max. value of data. Symbols are observations and lines are model. Red line corresponds to observations (e.g. Chlorophyll *a*), others are sub-components (e.g. individual phytoplankton species). See Tables S19 and S24 for observation and model component IDs, and mapping.

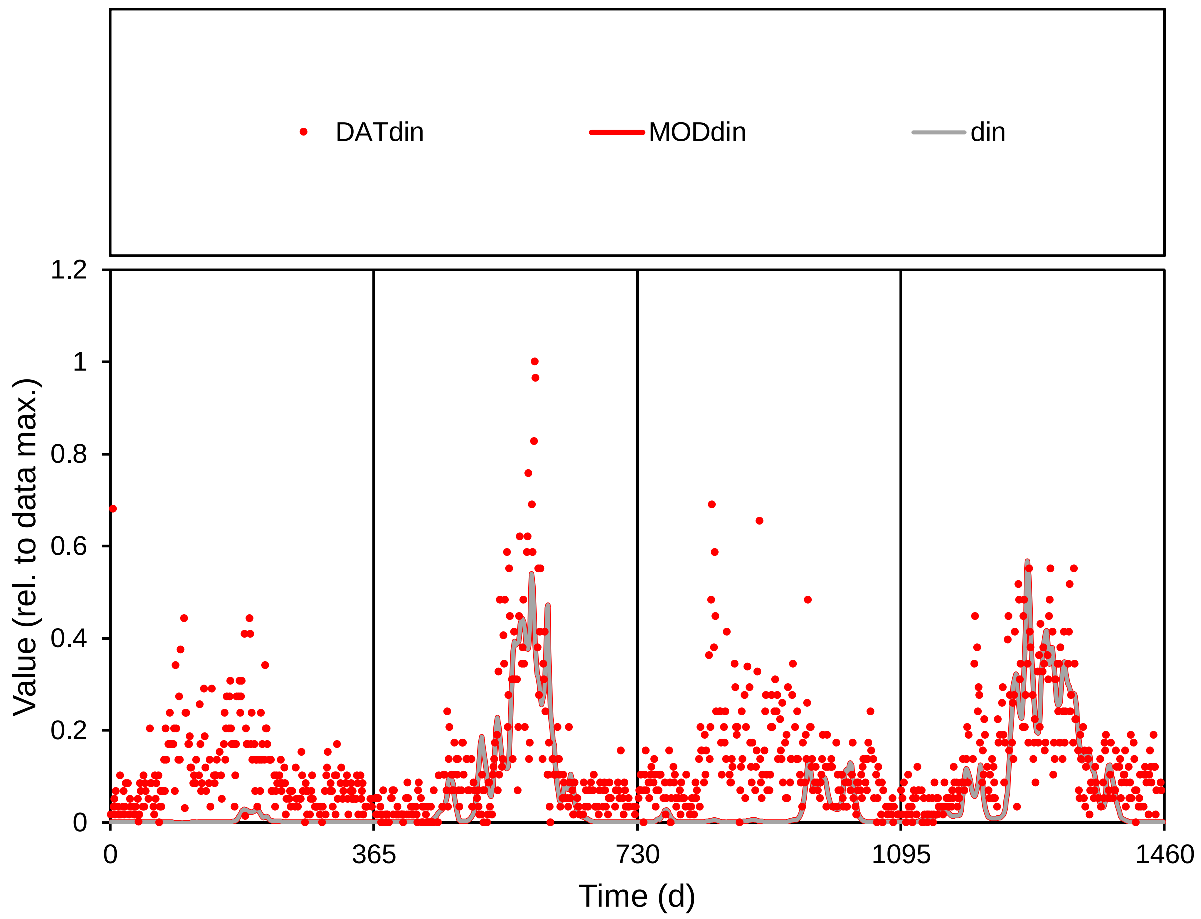

Figure S1(18). Model - data comparison for observation: din
Normalized to max. value of data. Symbols are observations and lines are model. Red line corresponds to observations (e.g. Chlorophyll *a*), others are sub-components (e.g. individual phytoplankton species). See Tables S19 and S24 for observation and model component IDs, and mapping.

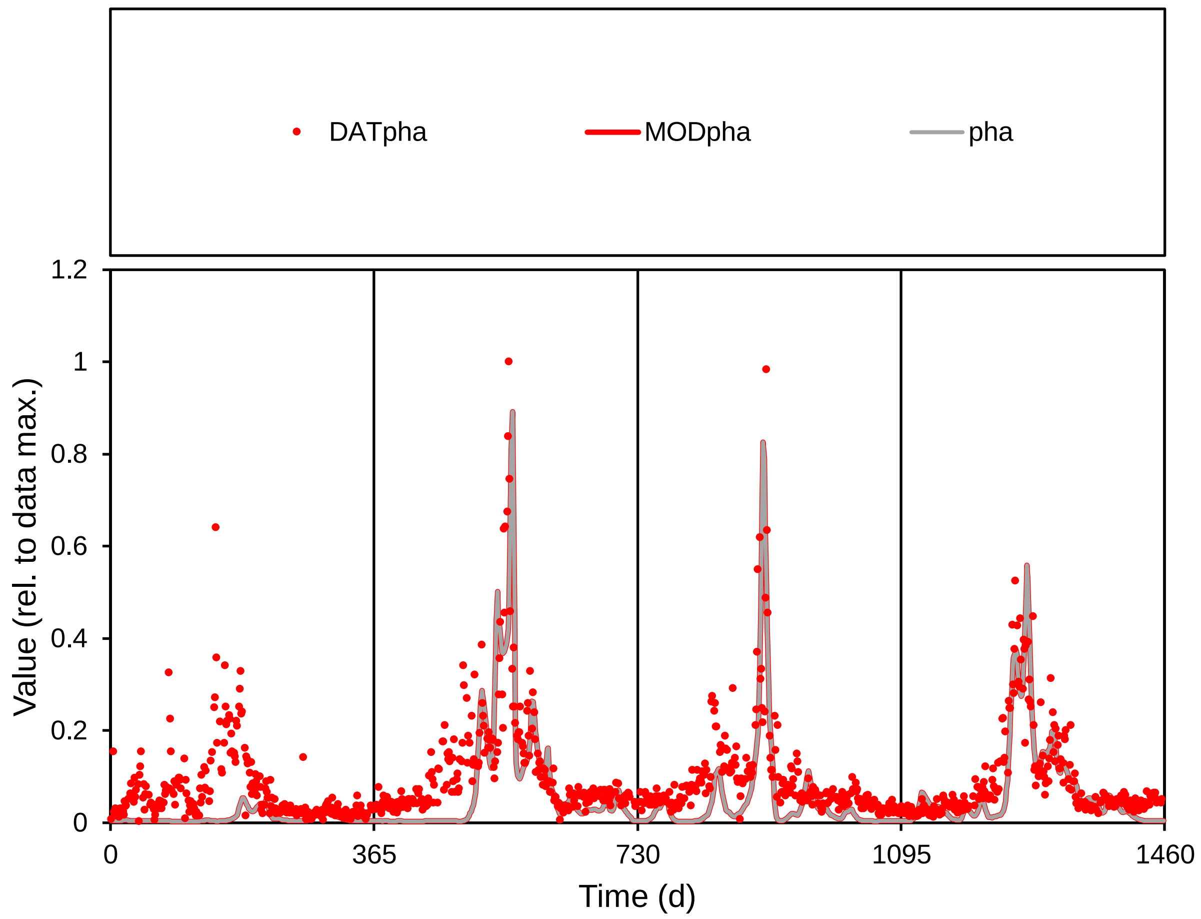

Figure S1(19). Model - data comparison for observation: pha
Normalized to max. value of data. Symbols are observations and lines are model. Red line corresponds to observations (e.g. Chlorophyll *a*), others are sub-components (e.g. individual phytoplankton species). See Tables S19 and S24 for observation and model component IDs, and mapping.

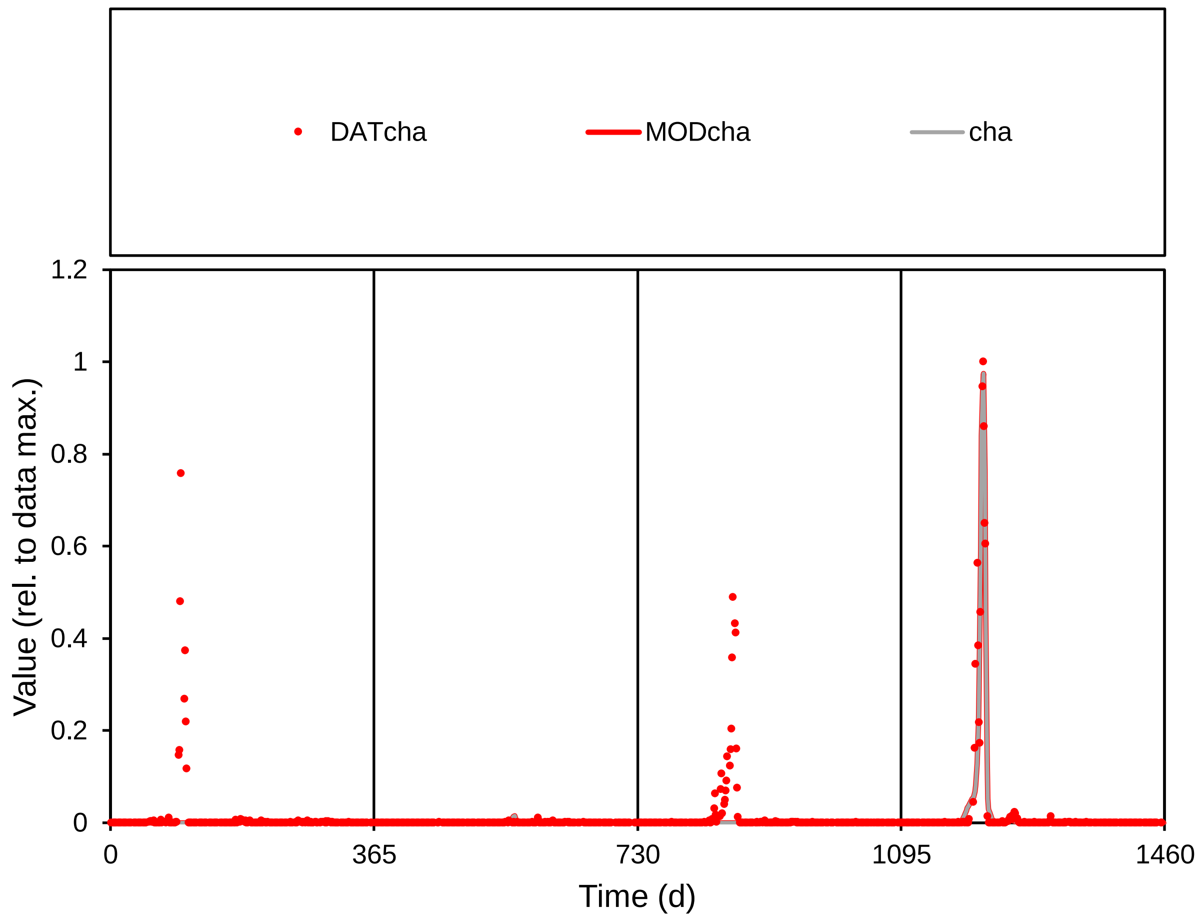

Figure S1(20). Model - data comparison for observation: cha
Normalized to max. value of data. Symbols are observations and lines are model. Red line corresponds to observations (e.g. Chlorophyll *a*), others are sub-components (e.g. individual phytoplankton species). See Tables S19 and S24 for observation and model component IDs, and mapping.

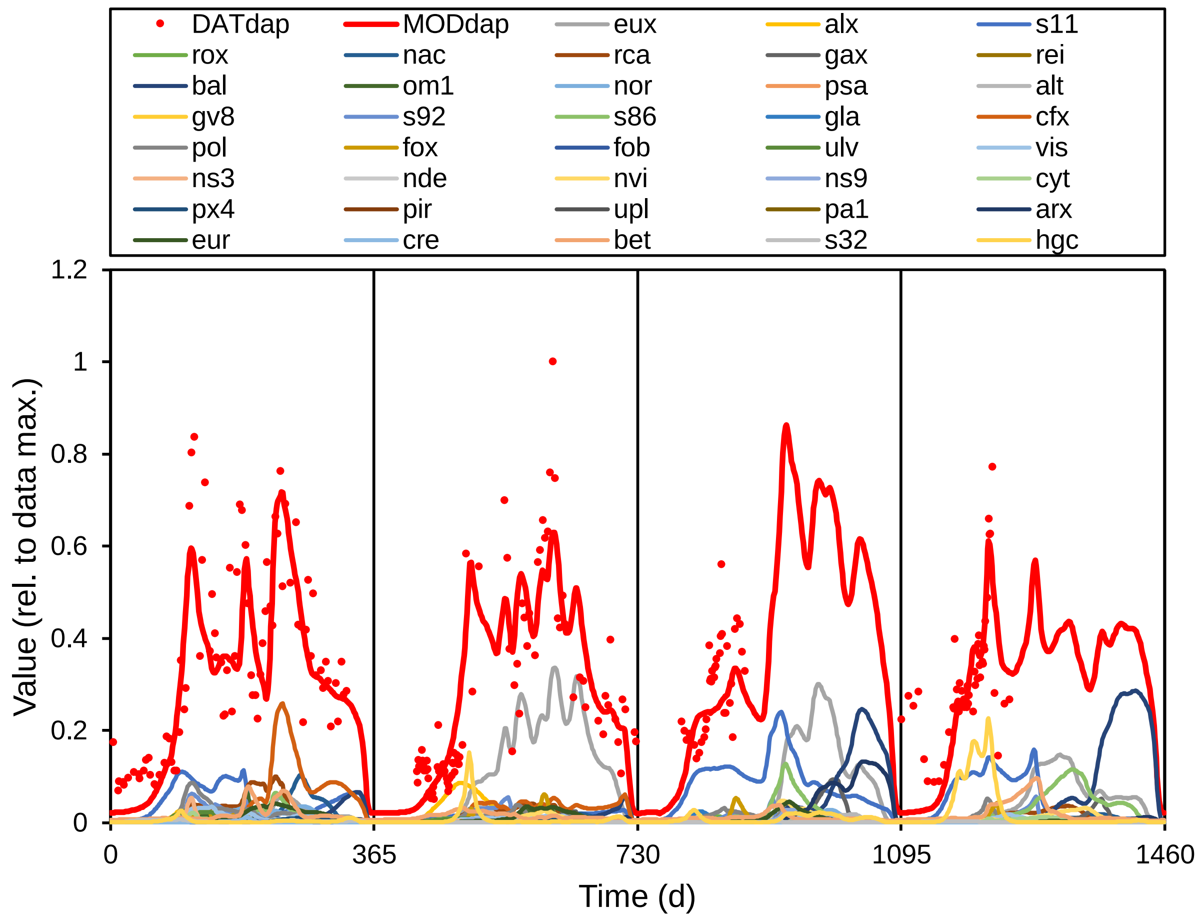

Figure S1(21). Model - data comparison for observation: dap
Normalized to max. value of data. Symbols are observations and lines are model. Red line corresponds to observations (e.g. Chlorophyll *a*), others are sub-components (e.g. individual phytoplankton species). See Tables S19 and S24 for observation and model component IDs, and mapping.

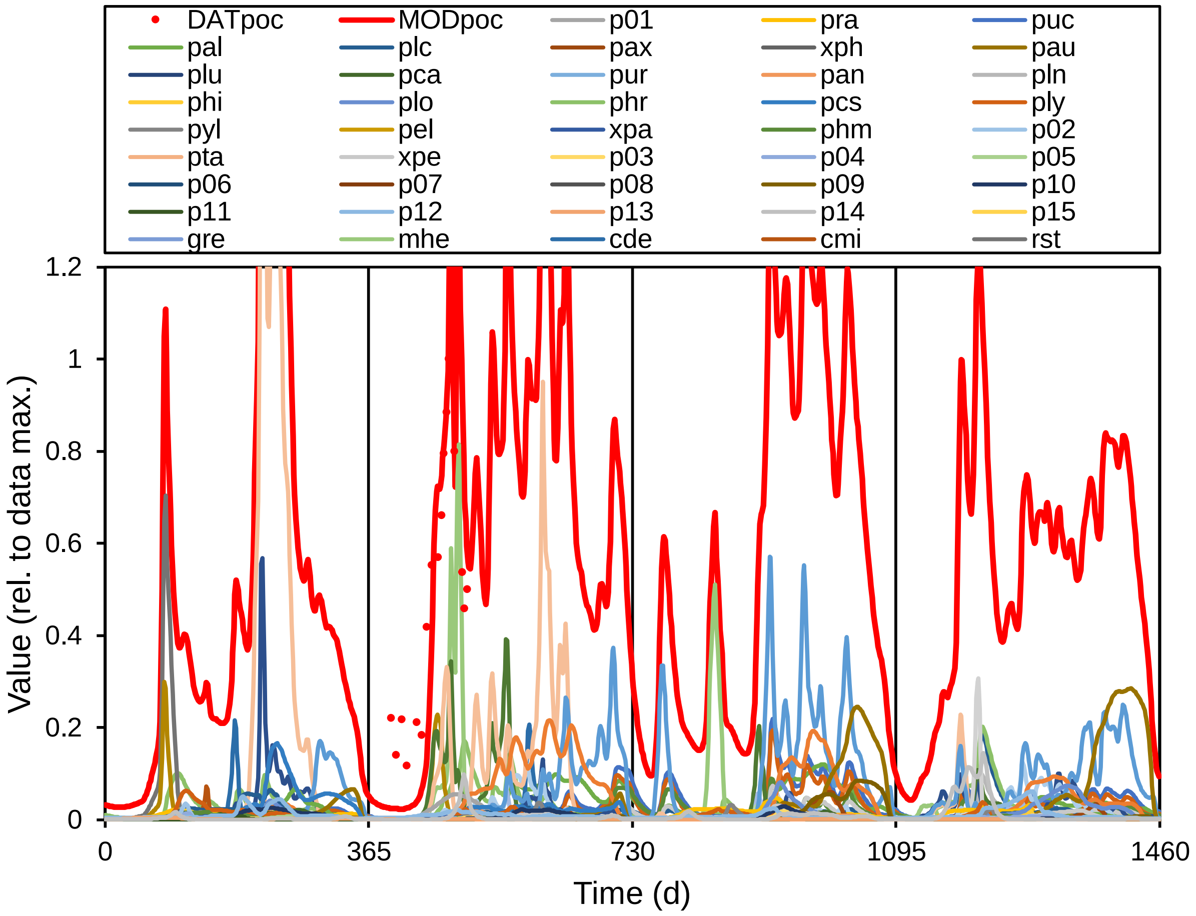

Figure S1(22). Model - data comparison for observation: poc

Normalized to max. value of data. Symbols are observations and lines are model. Red line corresponds to observations (e.g. Chlorophyll *a*), others are sub-components (e.g. individual phytoplankton species). See Tables S19 and S24 for observation and model component IDs, and mapping.

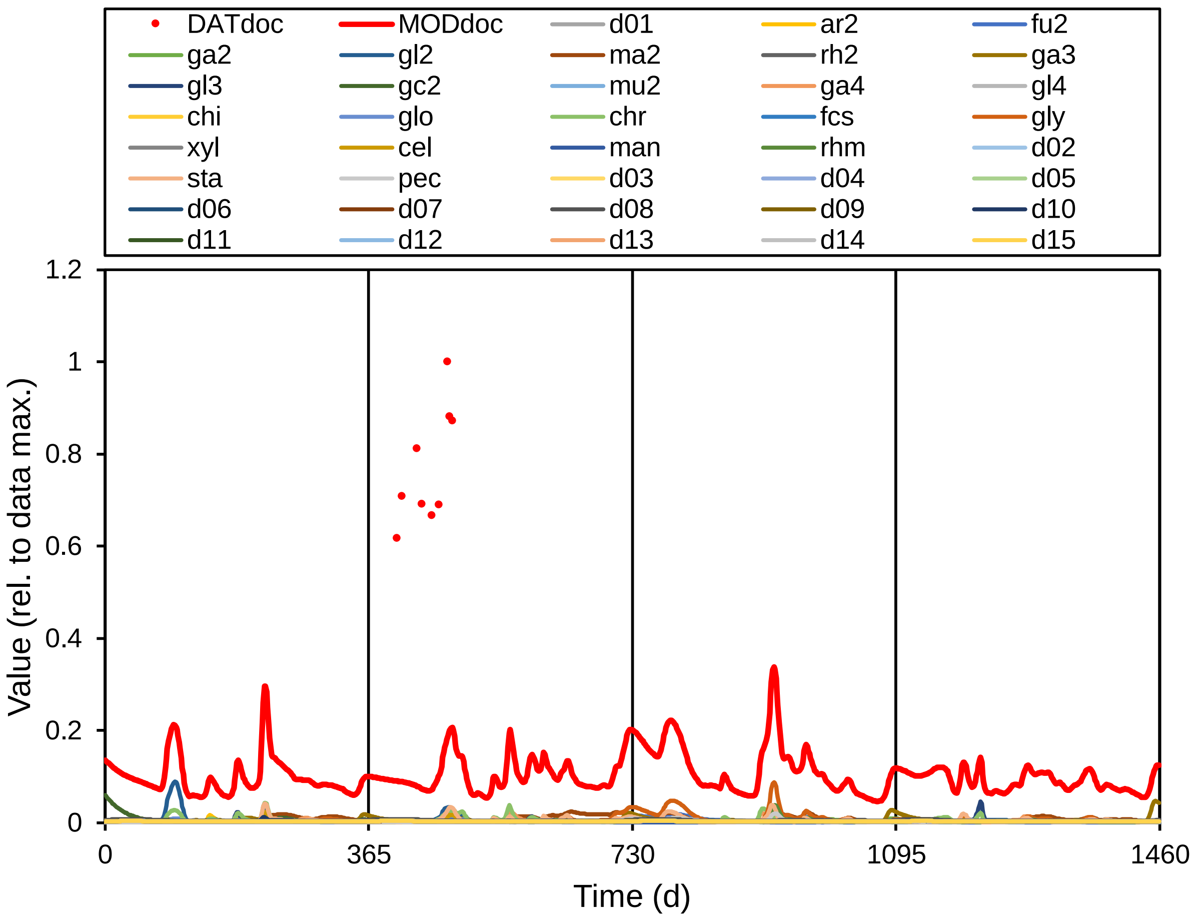

Figure S1(23). Model - data comparison for observation: doc

Normalized to max. value of data. Symbols are observations and lines are model. Red line corresponds to observations (e.g. Chlorophyll *a*), others are sub-components (e.g. individual phytoplankton species). See Tables S19 and S24 for observation and model component IDs, and mapping.

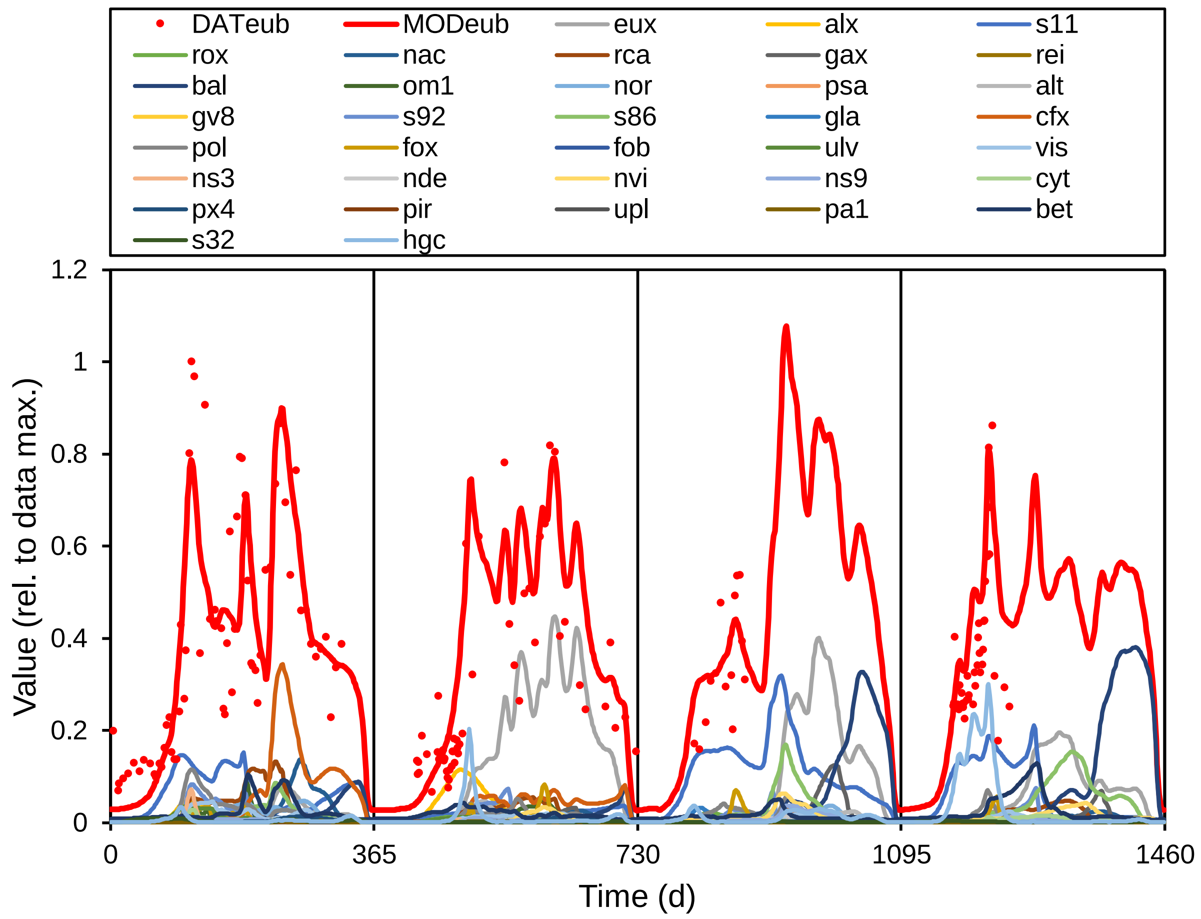

Figure S1(24). Model - data comparison for observation: eub
Normalized to max. value of data. Symbols are observations and lines are model. Red line corresponds to observations (e.g. Chlorophyll *a*), others are sub-components (e.g. individual phytoplankton species). See Tables S19 and S24 for observation and model component IDs, and mapping.

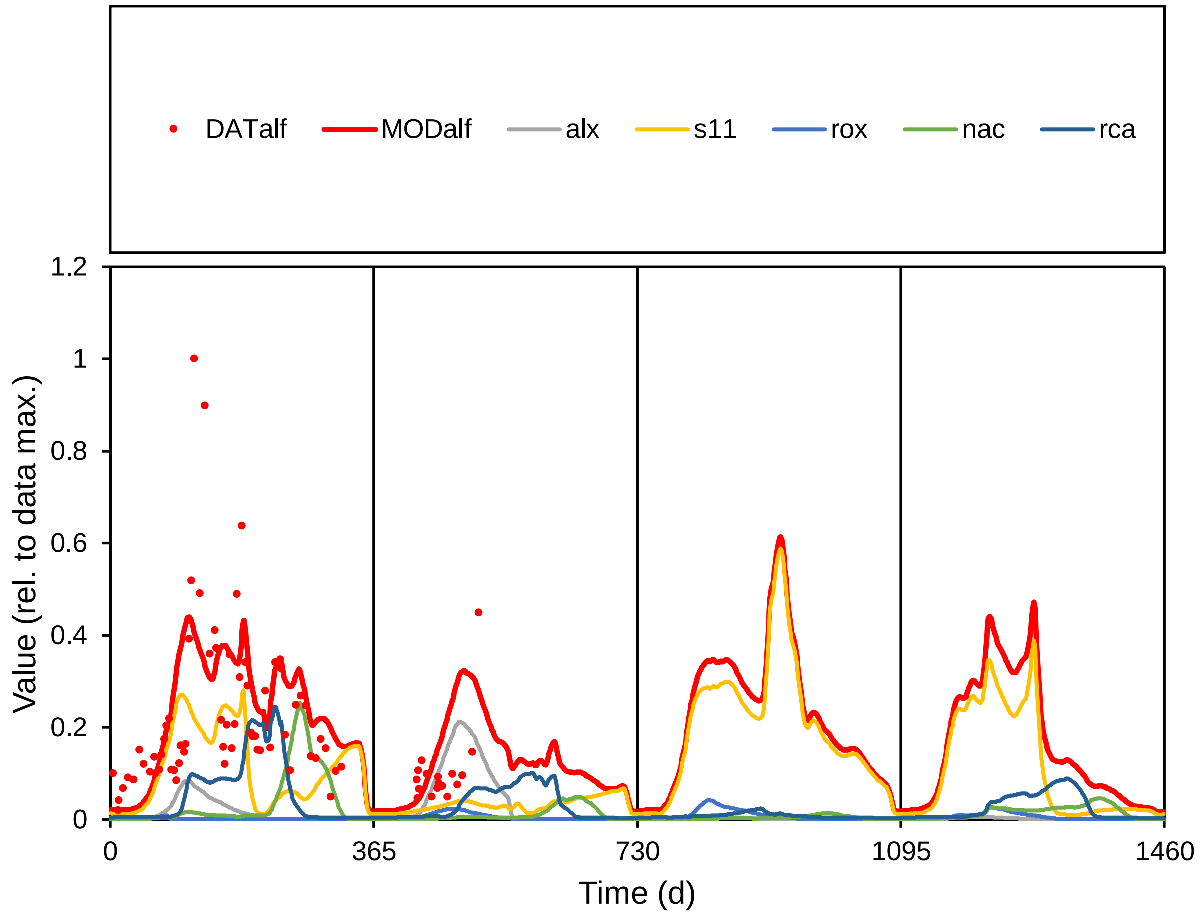

Figure S1(25). Model - data comparison for observation: alf
Normalized to max. value of data. Symbols are observations and lines are model. Red line corresponds to observations (e.g. Chlorophyll *a*), others are sub-components (e.g. individual phytoplankton species). See Tables S19 and S24 for observation and model component IDs, and mapping.

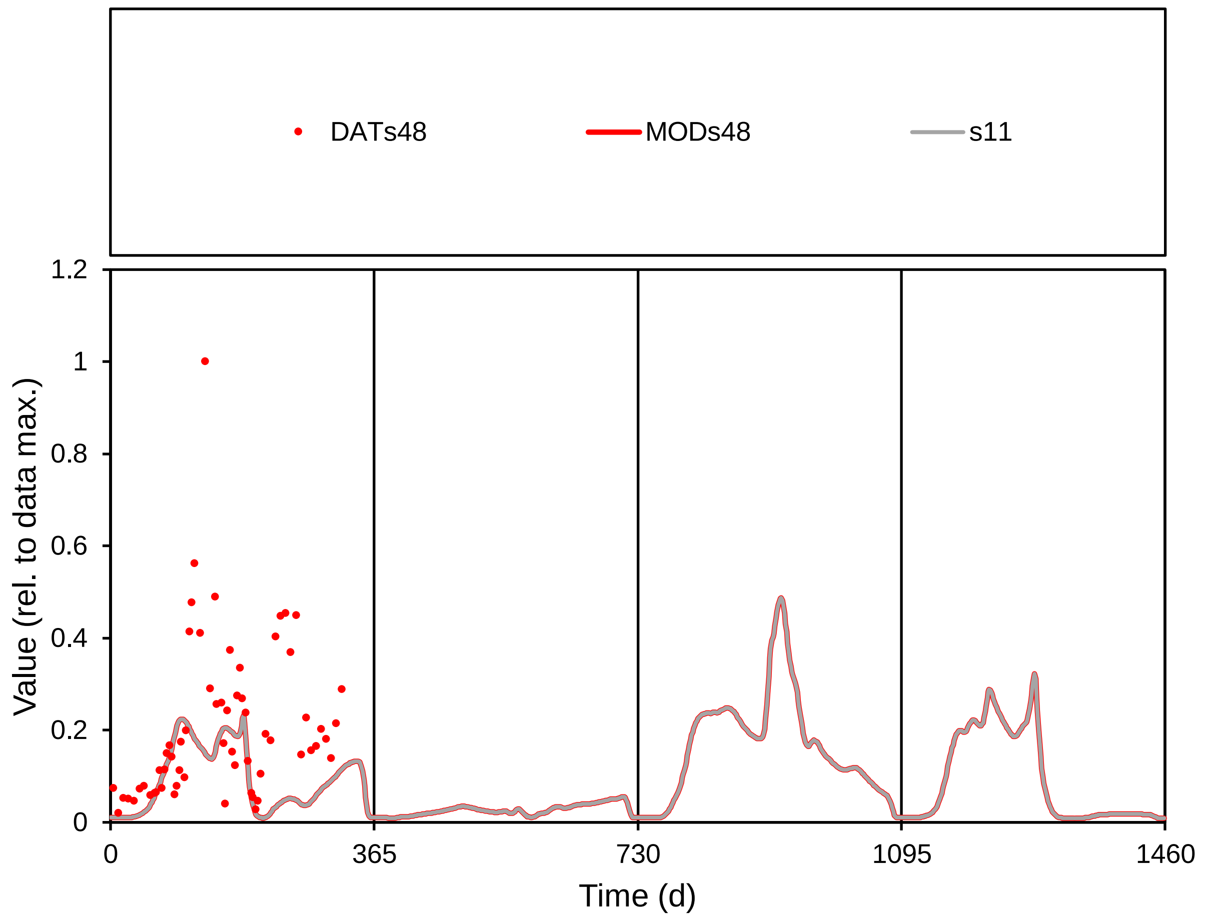

Figure S1(26). Model - data comparison for observation: s48
Normalized to max. value of data. Symbols are observations and lines are model. Red line corresponds to observations (e.g. Chlorophyll *a*), others are sub-components (e.g. individual phytoplankton species). See Tables S19 and S24 for observation and model component IDs, and mapping.

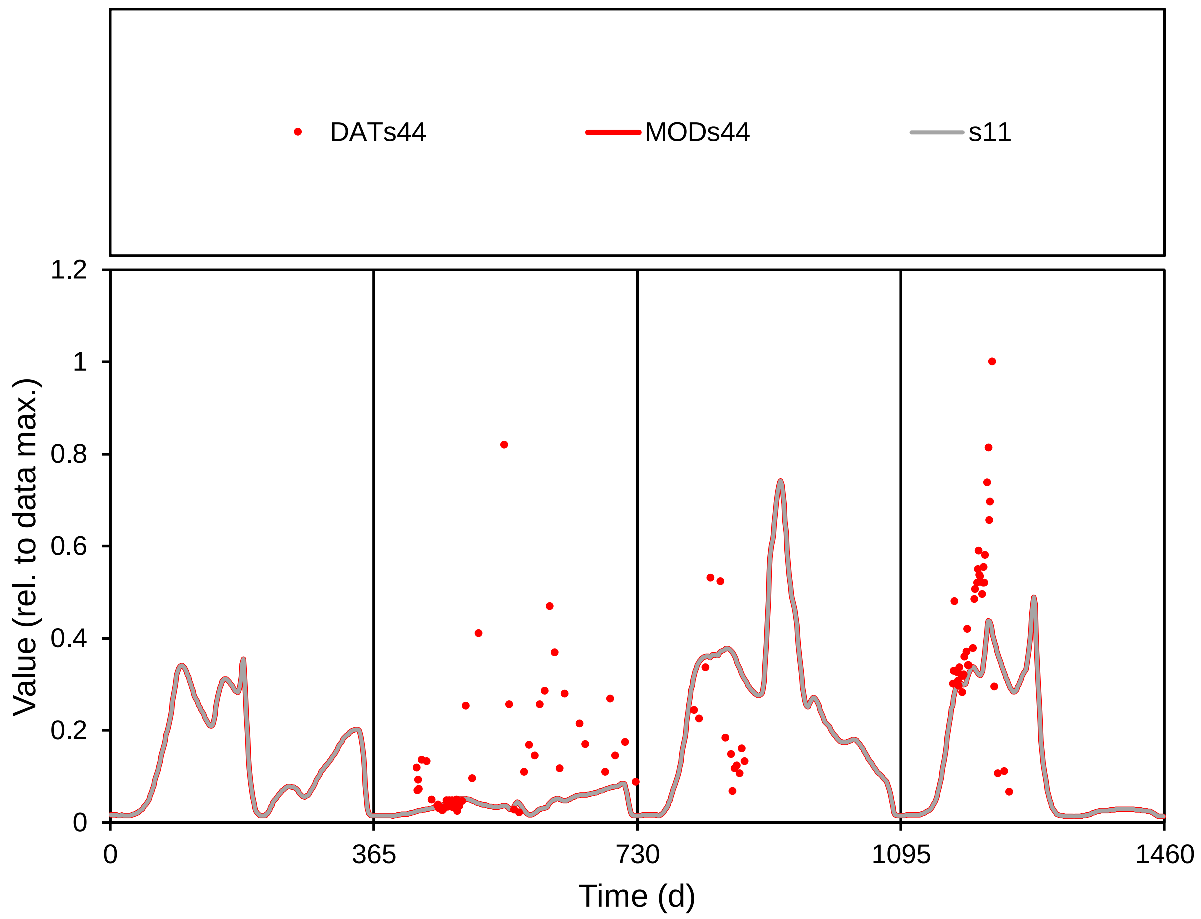

Figure S1(27). Model - data comparison for observation: s44
Normalized to max. value of data. Symbols are observations and lines are model. Red line corresponds to observations (e.g. Chlorophyll *a*), others are sub-components (e.g. individual phytoplankton species). See Tables S19 and S24 for observation and model component IDs, and mapping.

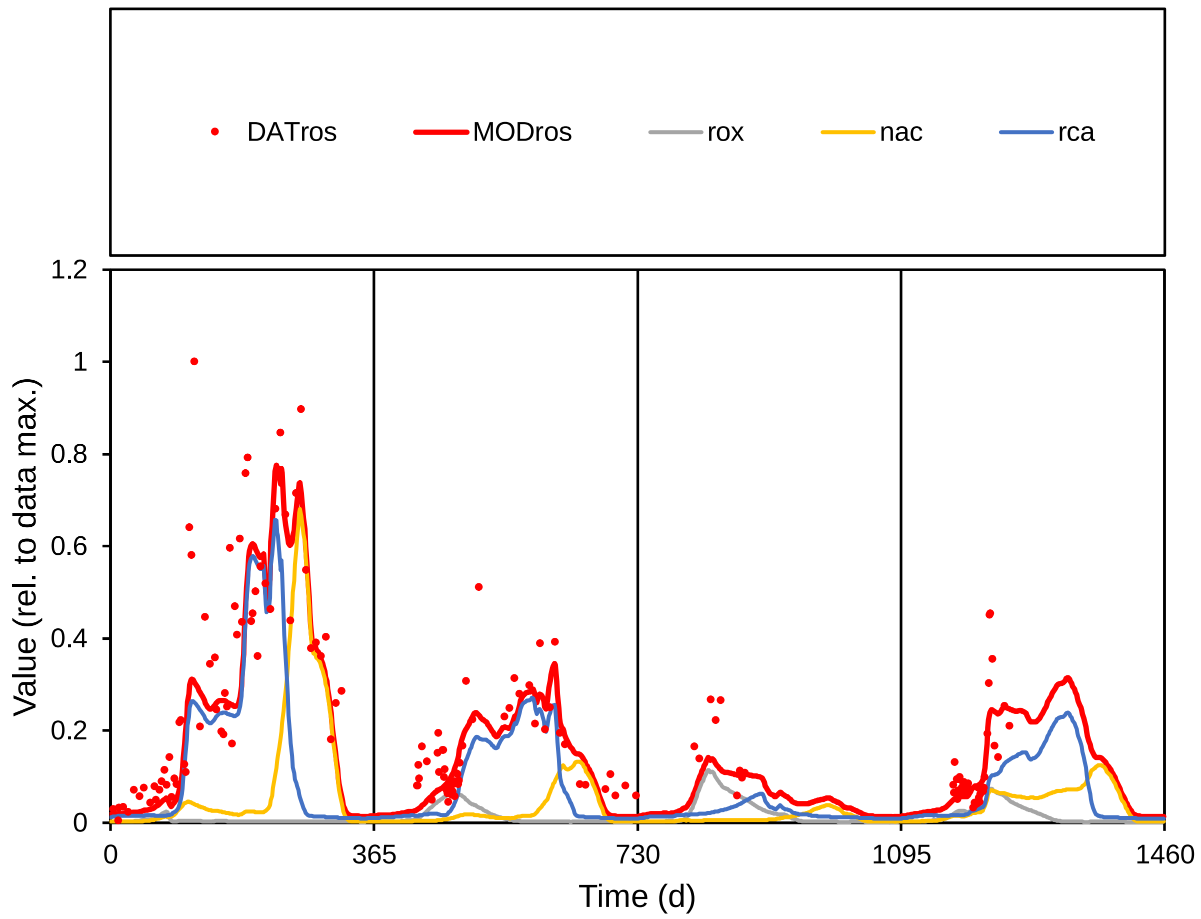

Figure S1(28). Model - data comparison for observation: ros
Normalized to max. value of data. Symbols are observations and lines are model. Red line corresponds to observations (e.g. Chlorophyll *a*), others are sub-components (e.g. individual phytoplankton species). See Tables S19 and S24 for observation and model component IDs, and mapping.

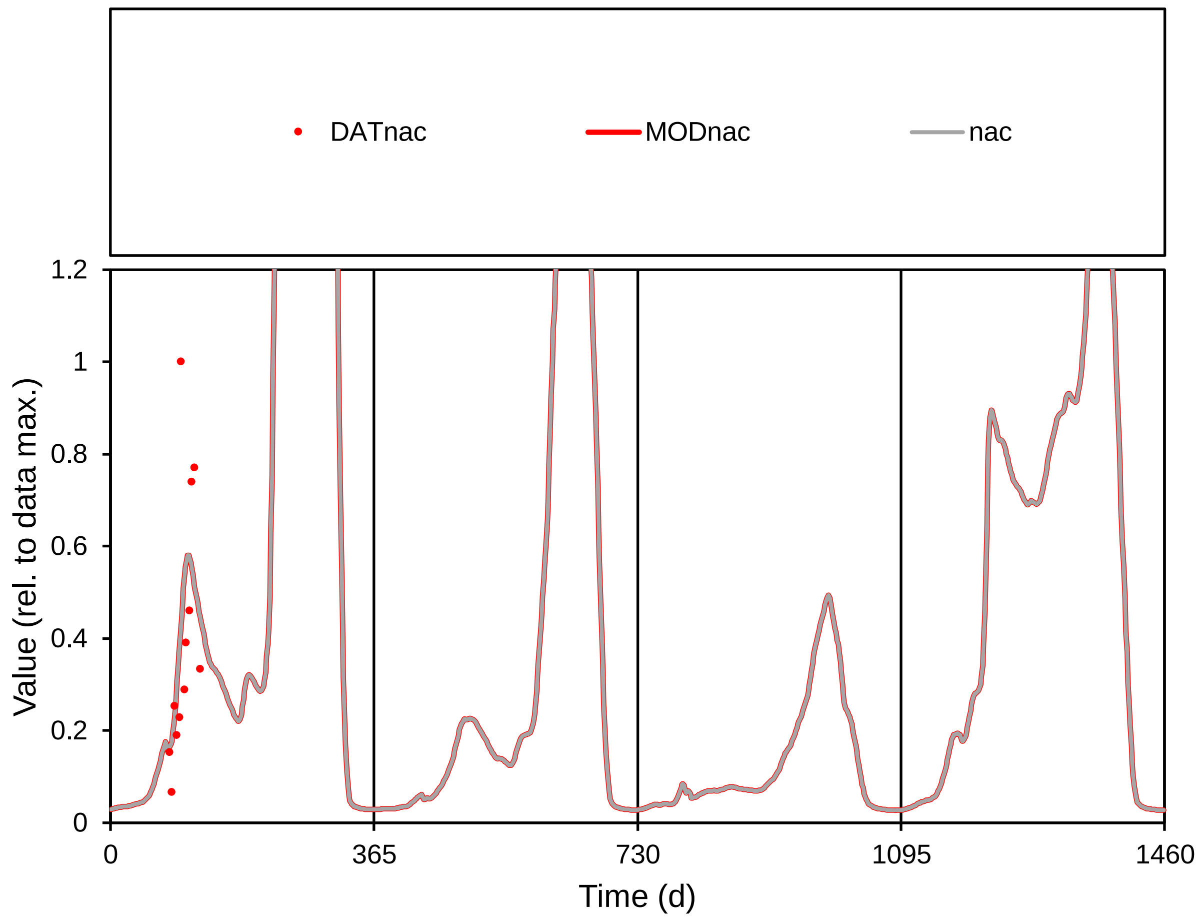

Figure S1(29). Model - data comparison for observation: nac
Normalized to max. value of data. Symbols are observations and lines are model. Red line corresponds to observations (e.g. Chlorophyll *a*), others are sub-components (e.g. individual phytoplankton species). See Tables S19 and S24 for observation and model component IDs, and mapping.

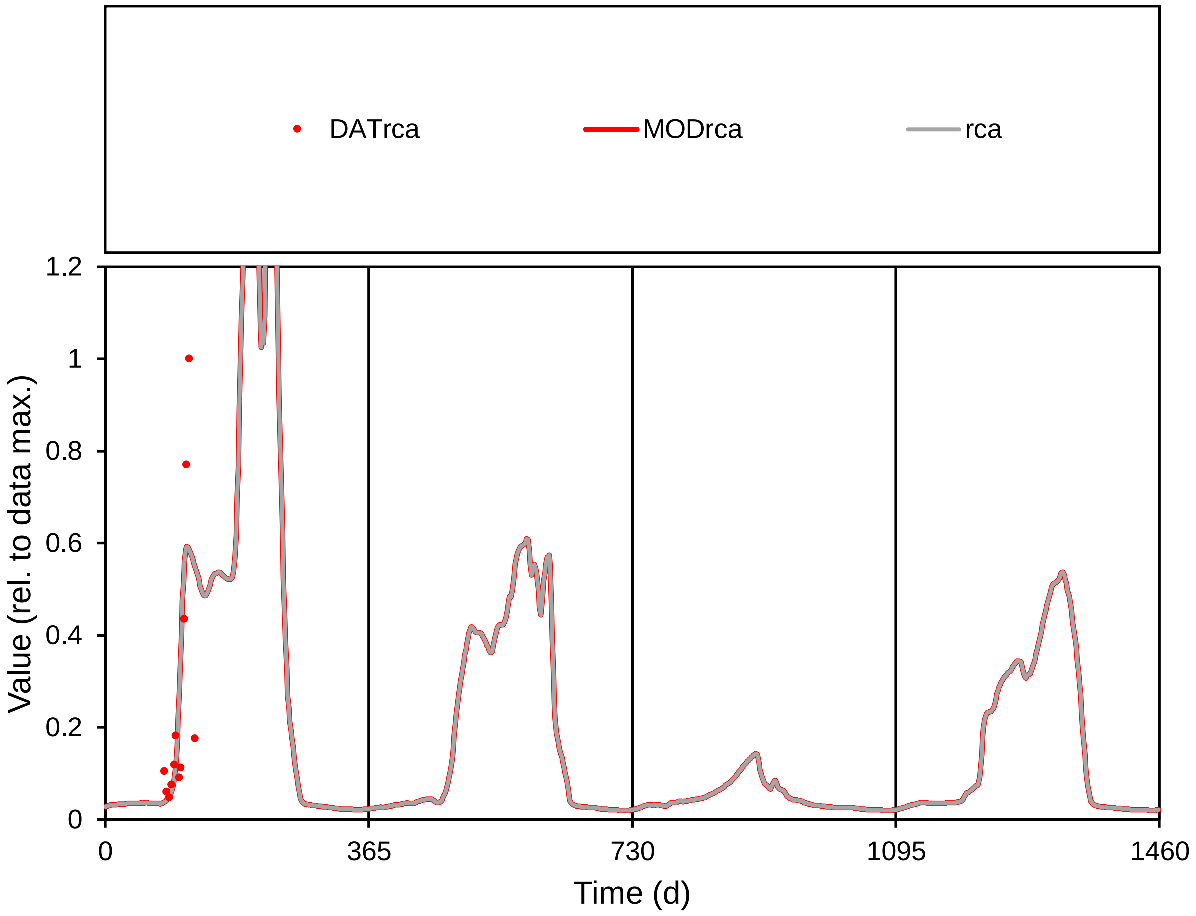

Figure S1(30). Model - data comparison for observation: rca
Normalized to max. value of data. Symbols are observations and lines are model. Red line corresponds to observations (e.g. Chlorophyll *a*), others are sub-components (e.g. individual phytoplankton species). See Tables S19 and S24 for observation and model component IDs, and mapping.

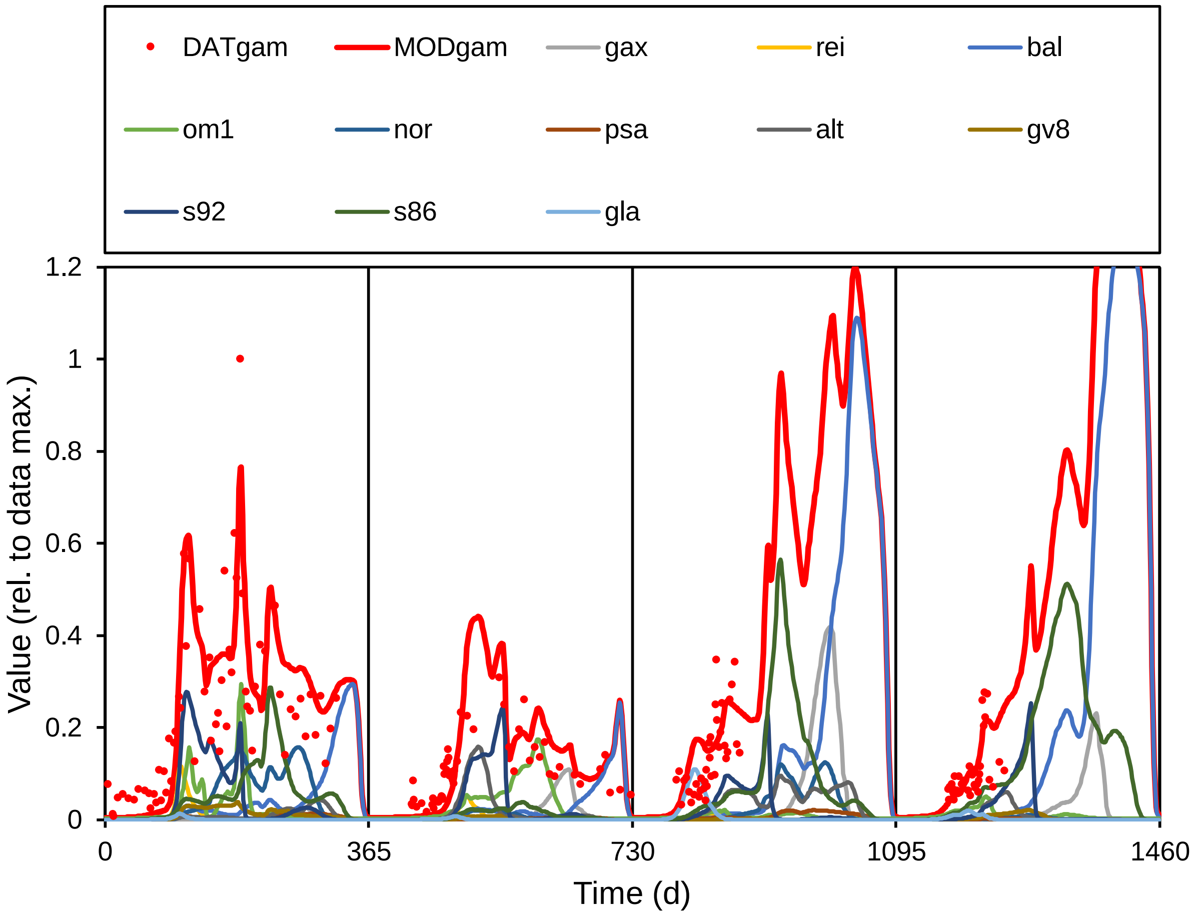

Figure S1(31). Model - data comparison for observation: gam
Normalized to max. value of data. Symbols are observations and lines are model. Red line corresponds to observations (e.g. Chlorophyll *a*), others are sub-components (e.g. individual phytoplankton species). See Tables S19 and S24 for observation and model component IDs, and mapping.

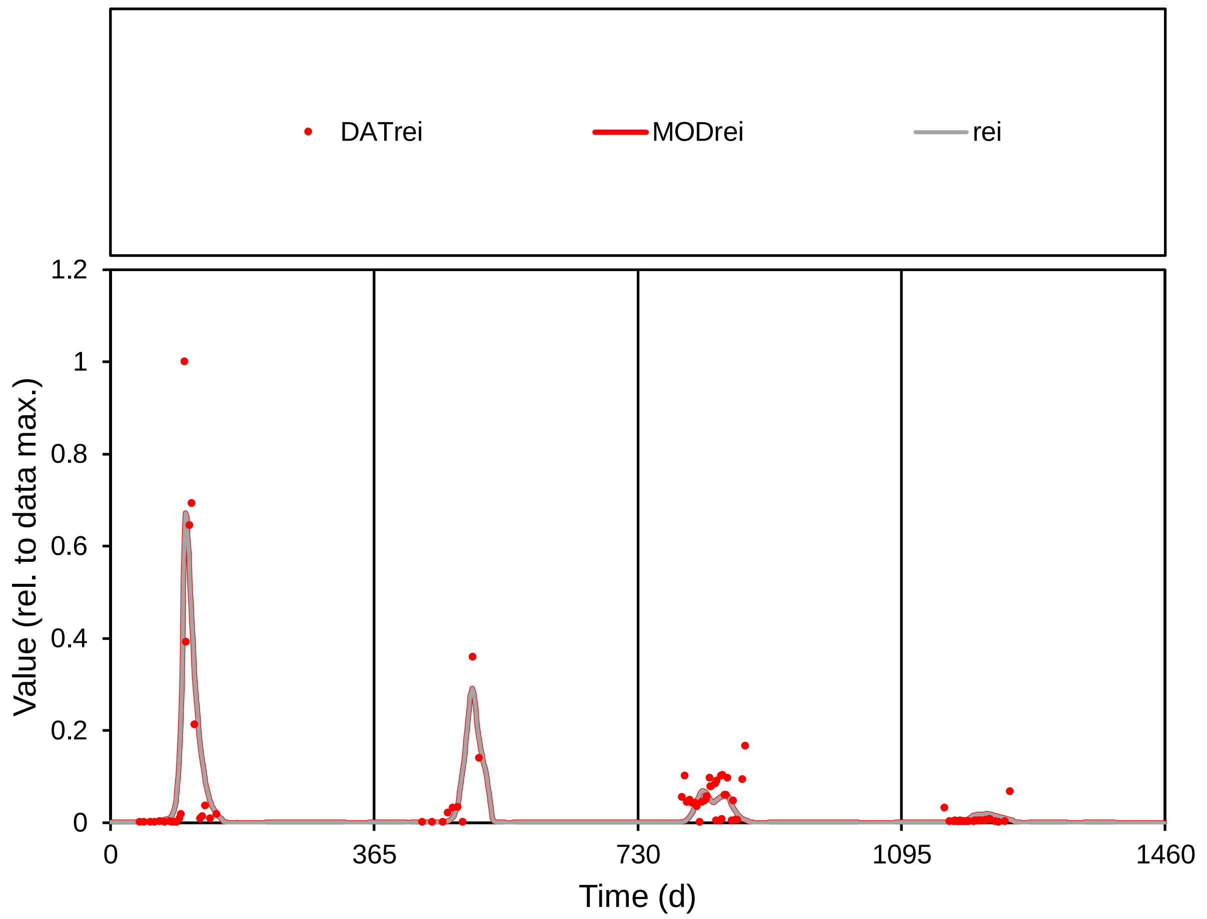

Figure S1(32). Model - data comparison for observation: rei
Normalized to max. value of data. Symbols are observations and lines are model. Red line corresponds to observations (e.g. Chlorophyll *a*), others are sub-components (e.g. individual phytoplankton species). See Tables S19 and S24 for observation and model component IDs, and mapping.

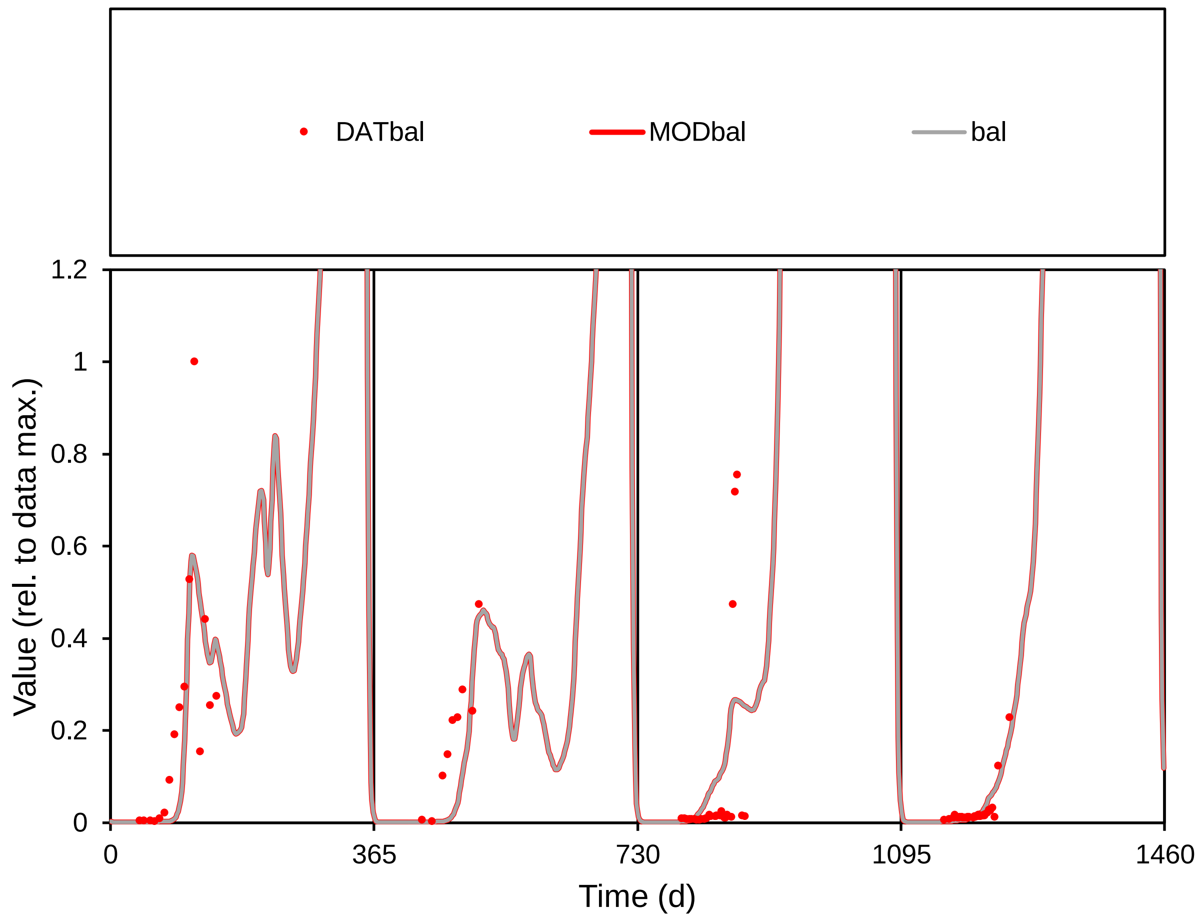

Figure S1(33). Model - data comparison for observation: bal
Normalized to max. value of data. Symbols are observations and lines are model. Red line corresponds to observations (e.g. Chlorophyll *a*), others are sub-components (e.g. individual phytoplankton species). See Tables S19 and S24 for observation and model component IDs, and mapping.

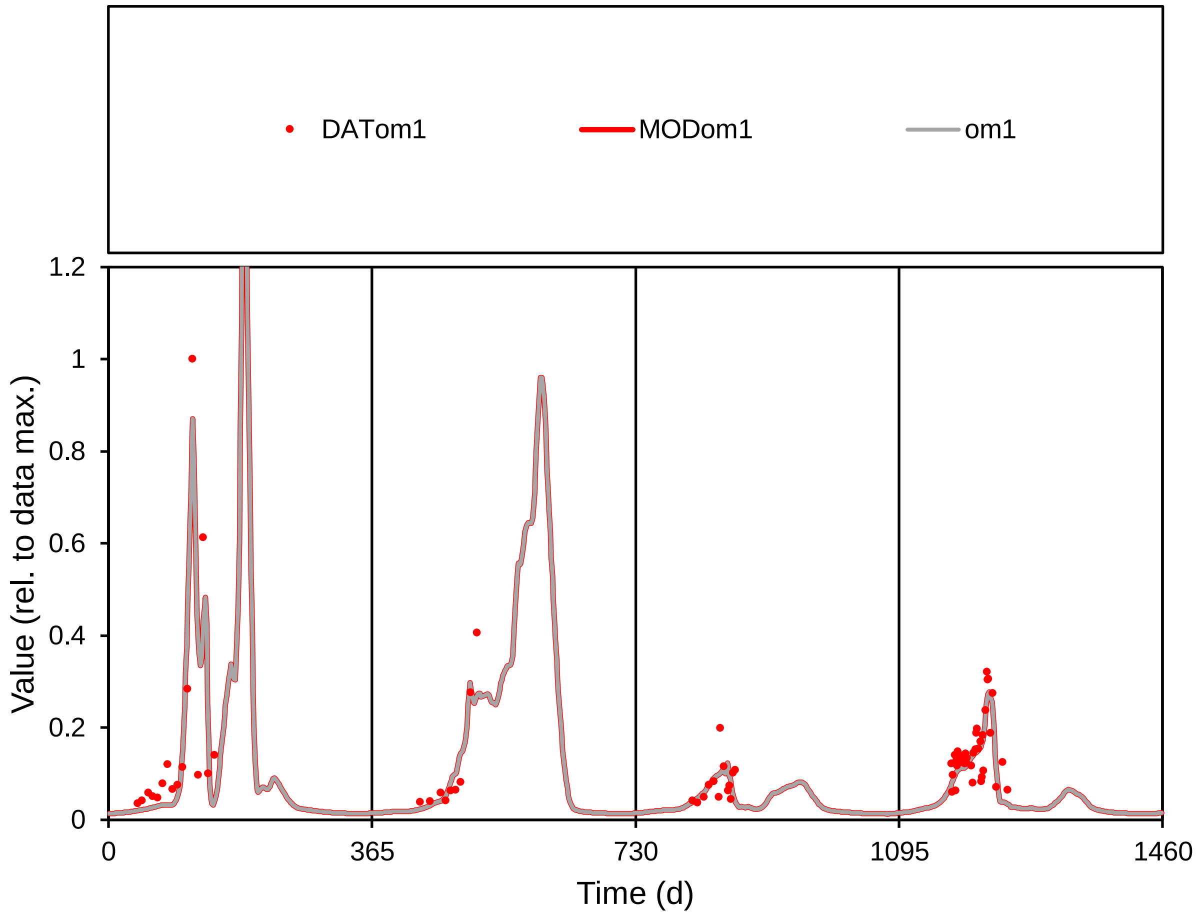

Figure S1(34). Model - data comparison for observation: om1
Normalized to max. value of data. Symbols are observations and lines are model. Red line corresponds to observations (e.g. Chlorophyll *a*), others are sub-components (e.g. individual phytoplankton species). See Tables S19 and S24 for observation and model component IDs, and mapping.

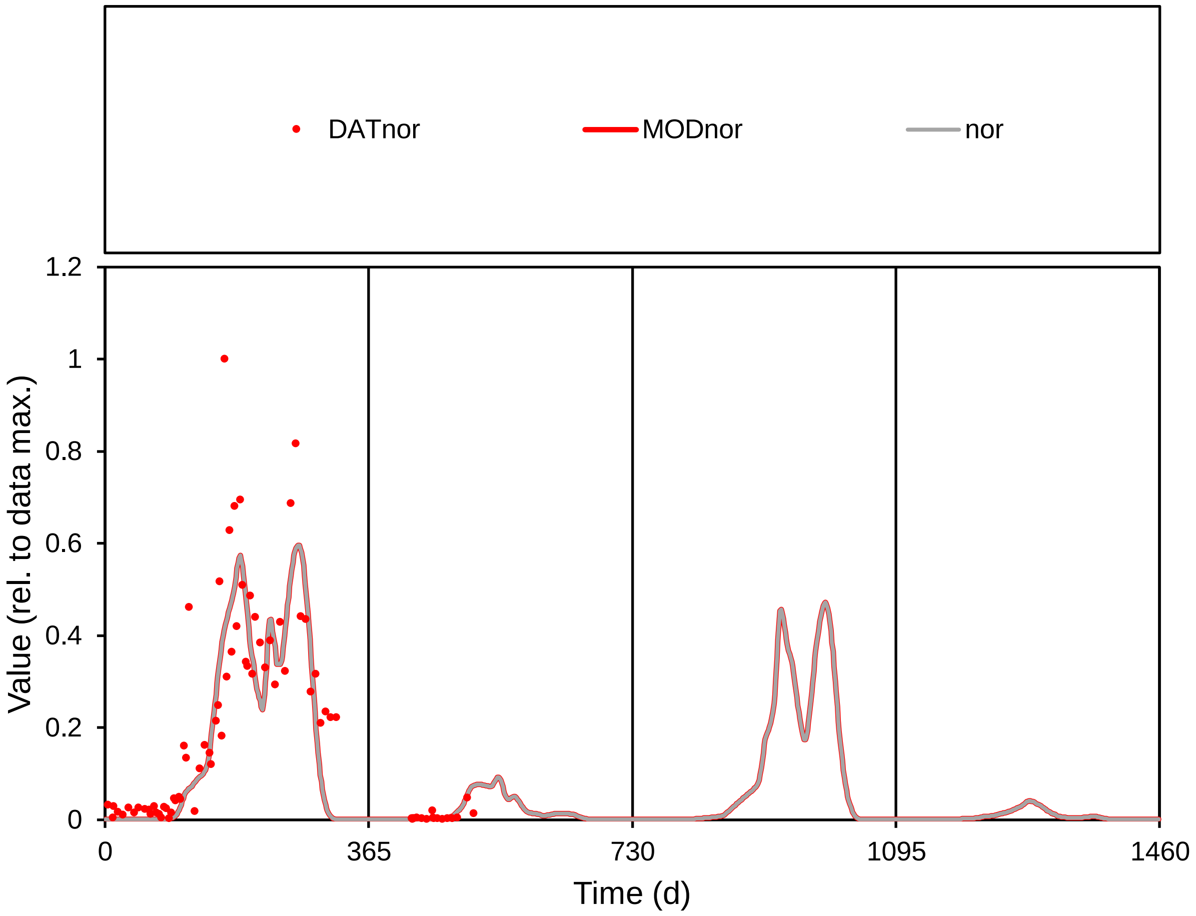

Figure S1(35). Model - data comparison for observation: nor
Normalized to max. value of data. Symbols are observations and lines are model. Red line corresponds to observations (e.g. Chlorophyll *a*), others are sub-components (e.g. individual phytoplankton species). See Tables S19 and S24 for observation and model component IDs, and mapping.

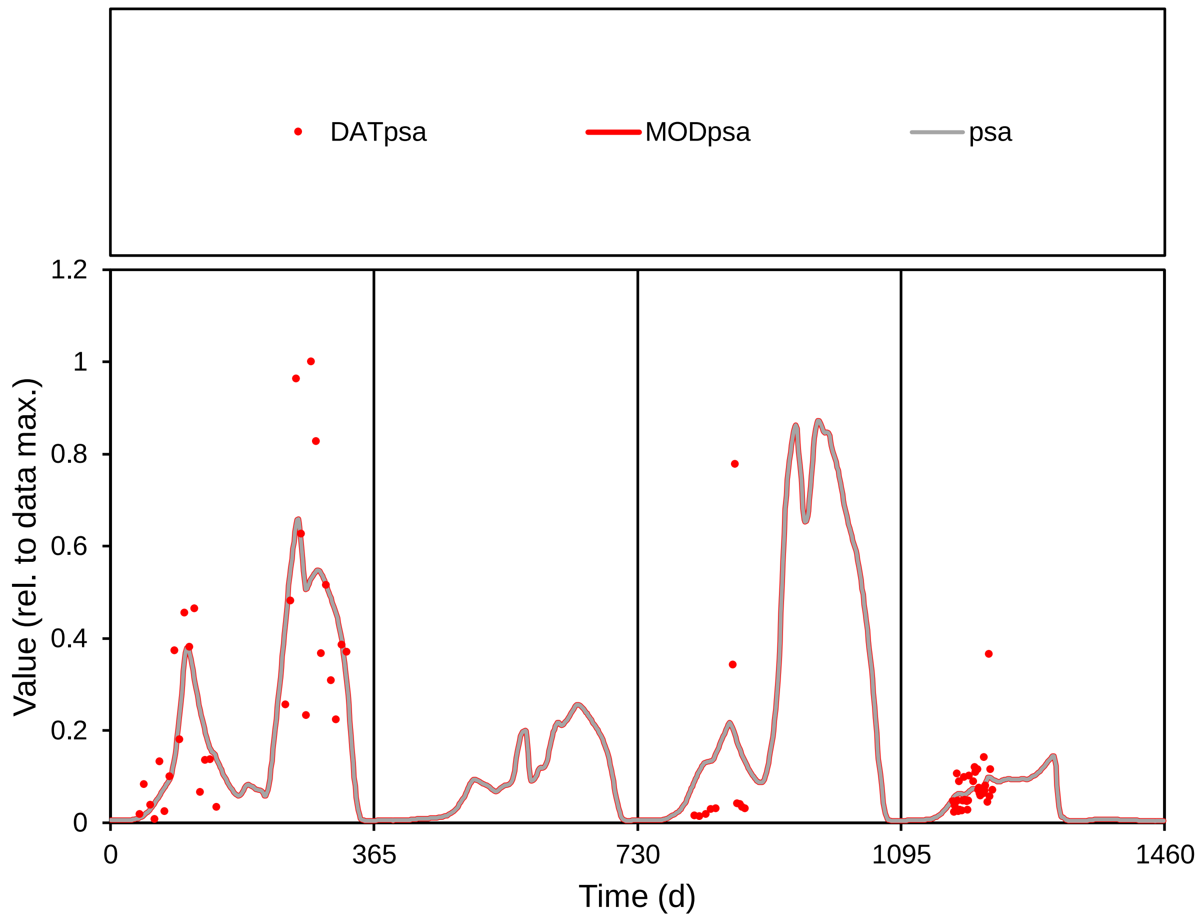

Figure S1(36). Model - data comparison for observation: psa
Normalized to max. value of data. Symbols are observations and lines are model. Red line corresponds to observations (e.g. Chlorophyll *a*), others are sub-components (e.g. individual phytoplankton species). See Tables S19 and S24 for observation and model component IDs, and mapping.

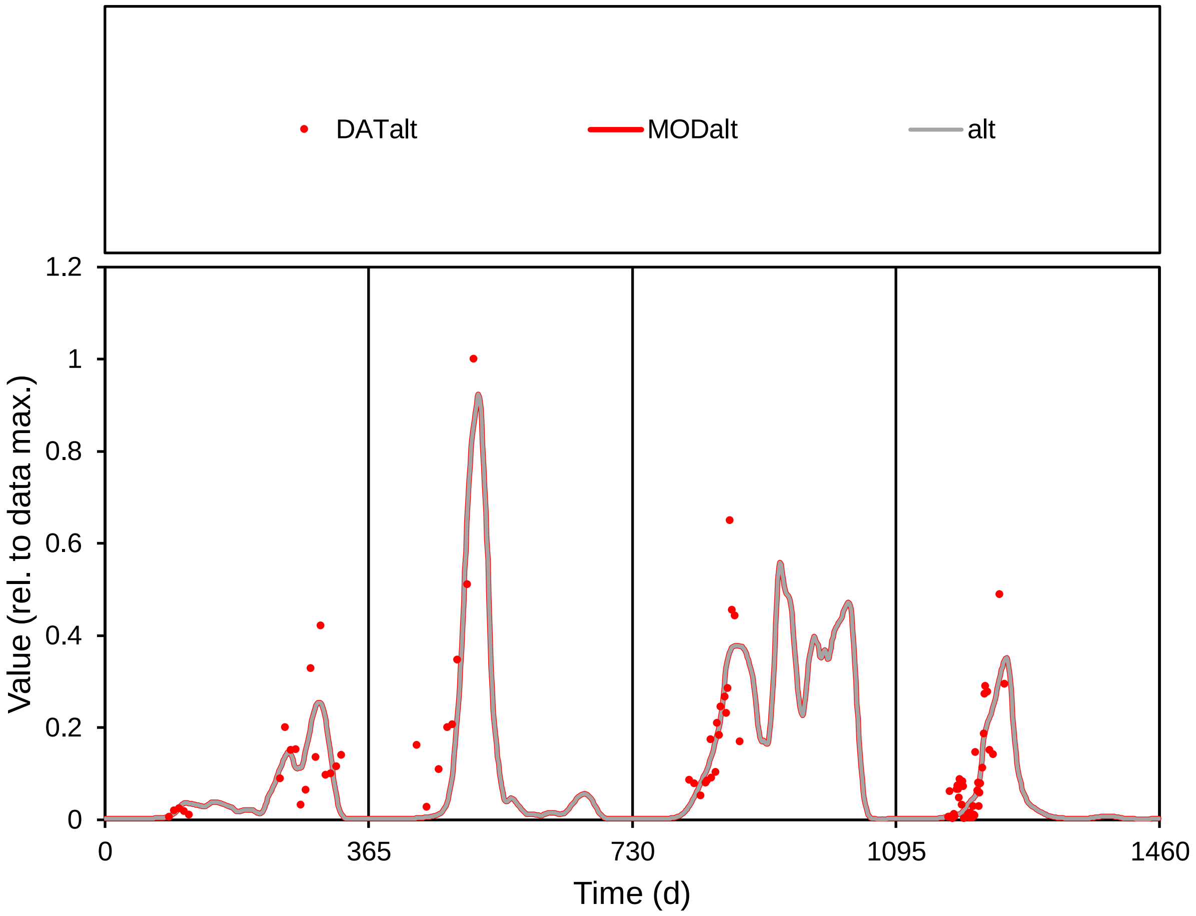

Figure S1(37). Model - data comparison for observation: alt
Normalized to max. value of data. Symbols are observations and lines are model. Red line corresponds to observations (e.g. Chlorophyll *a*), others are sub-components (e.g. individual phytoplankton species). See Tables S19 and S24 for observation and model component IDs, and mapping.

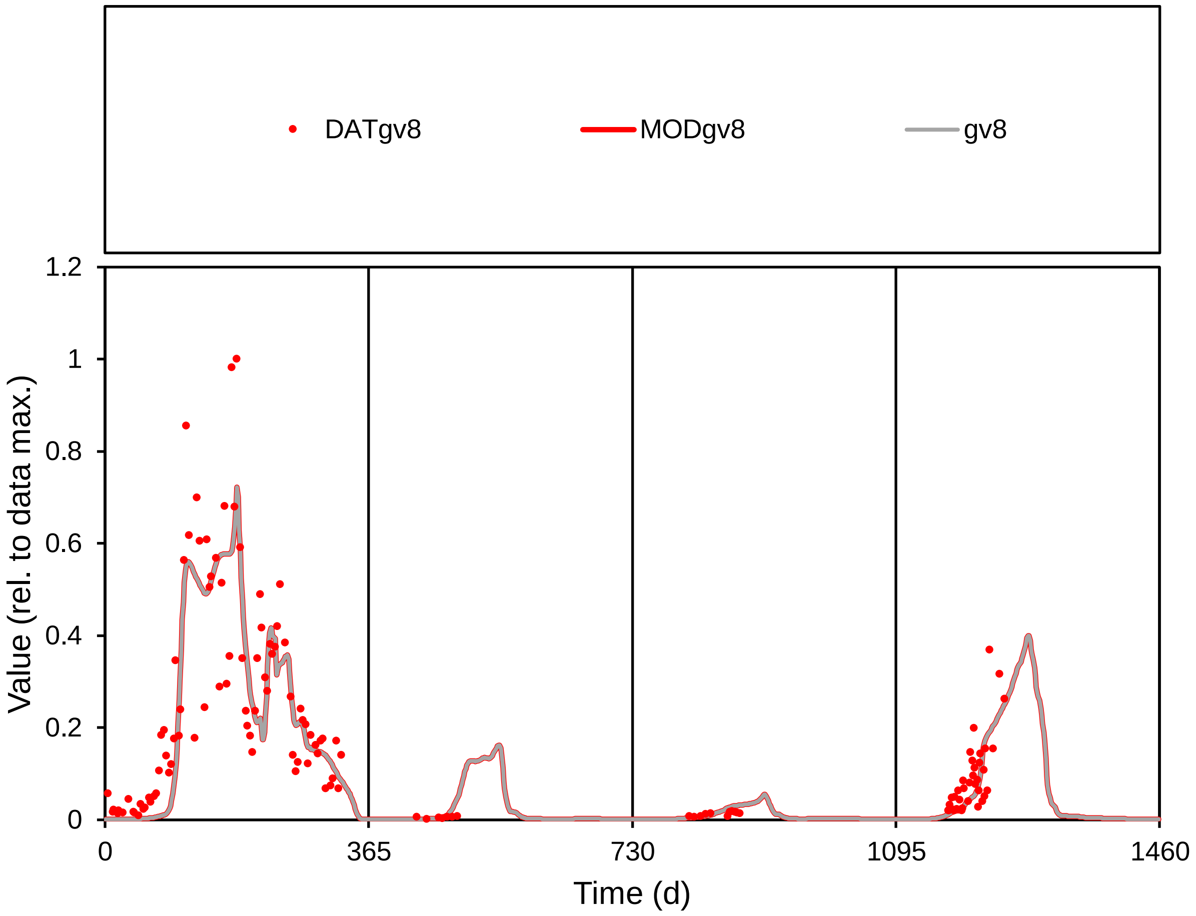

Figure S1(38). Model - data comparison for observation: gv8
Normalized to max. value of data. Symbols are observations and lines are model. Red line corresponds to observations (e.g. Chlorophyll *a*), others are sub-components (e.g. individual phytoplankton species). See Tables S19 and S24 for observation and model component IDs, and mapping.

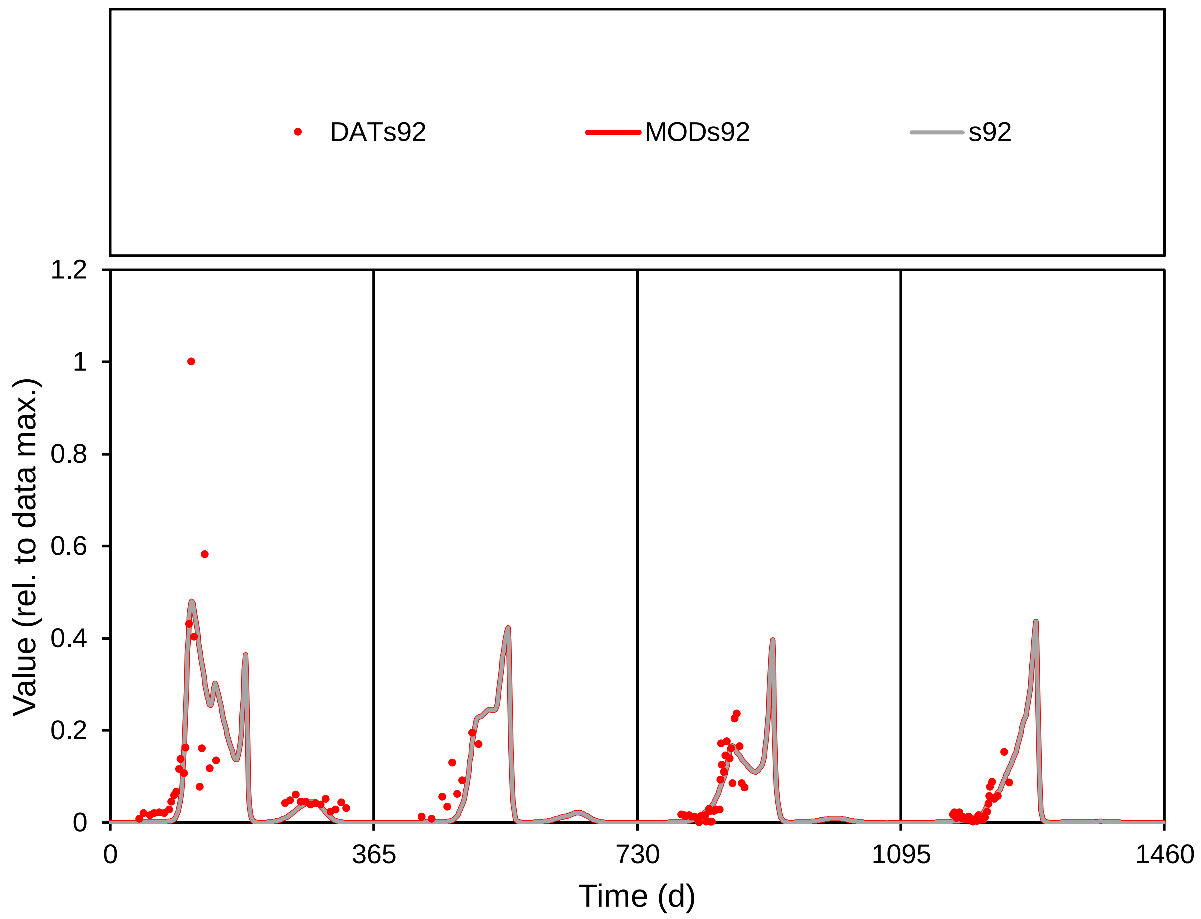

Figure S1(39). Model - data comparison for observation: s92
Normalized to max. value of data. Symbols are observations and lines are model. Red line corresponds to observations (e.g. Chlorophyll *a*), others are sub-components (e.g. individual phytoplankton species). See Tables S19 and S24 for observation and model component IDs, and mapping.

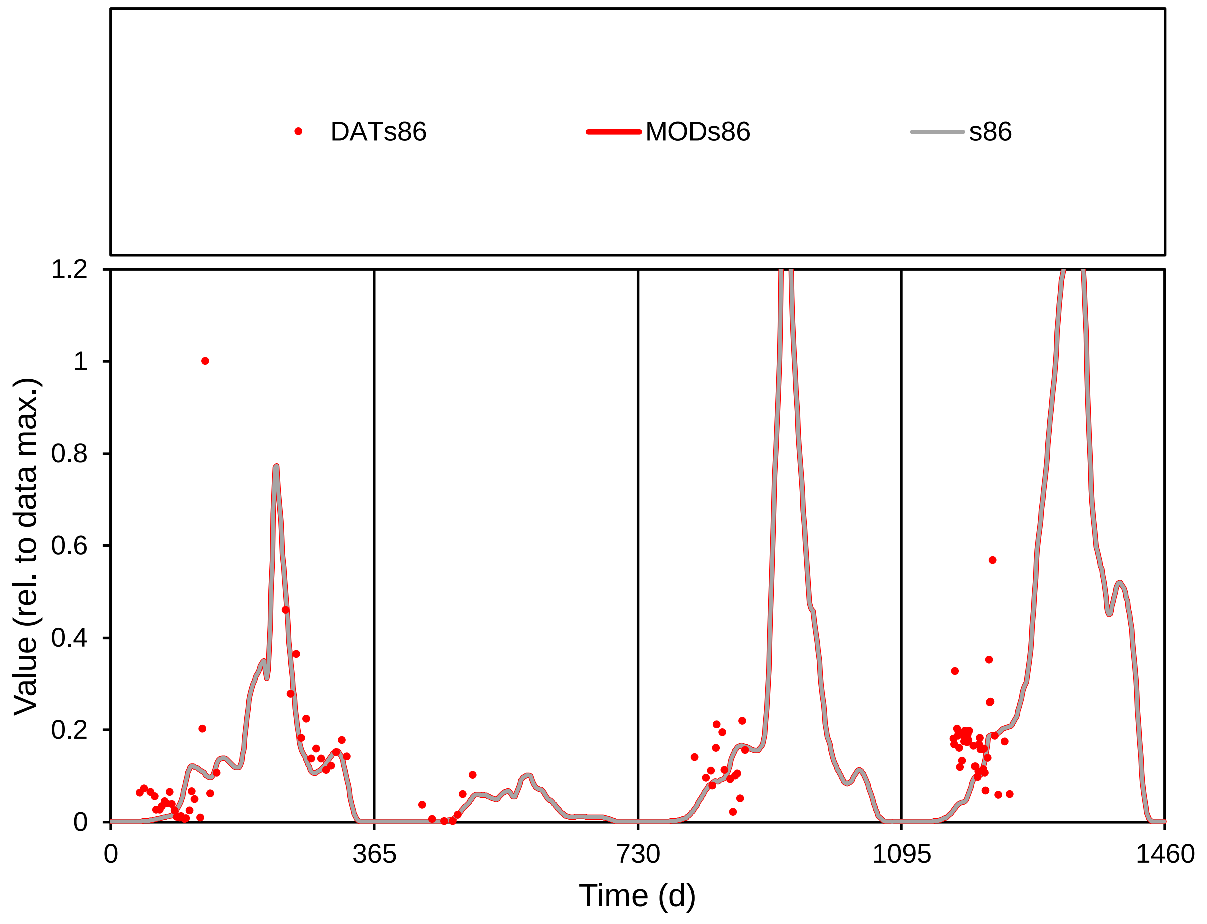

Figure S1(40). Model - data comparison for observation: s86
Normalized to max. value of data. Symbols are observations and lines are model. Red line corresponds to observations (e.g. Chlorophyll *a*), others are sub-components (e.g. individual phytoplankton species). See Tables S19 and S24 for observation and model component IDs, and mapping.

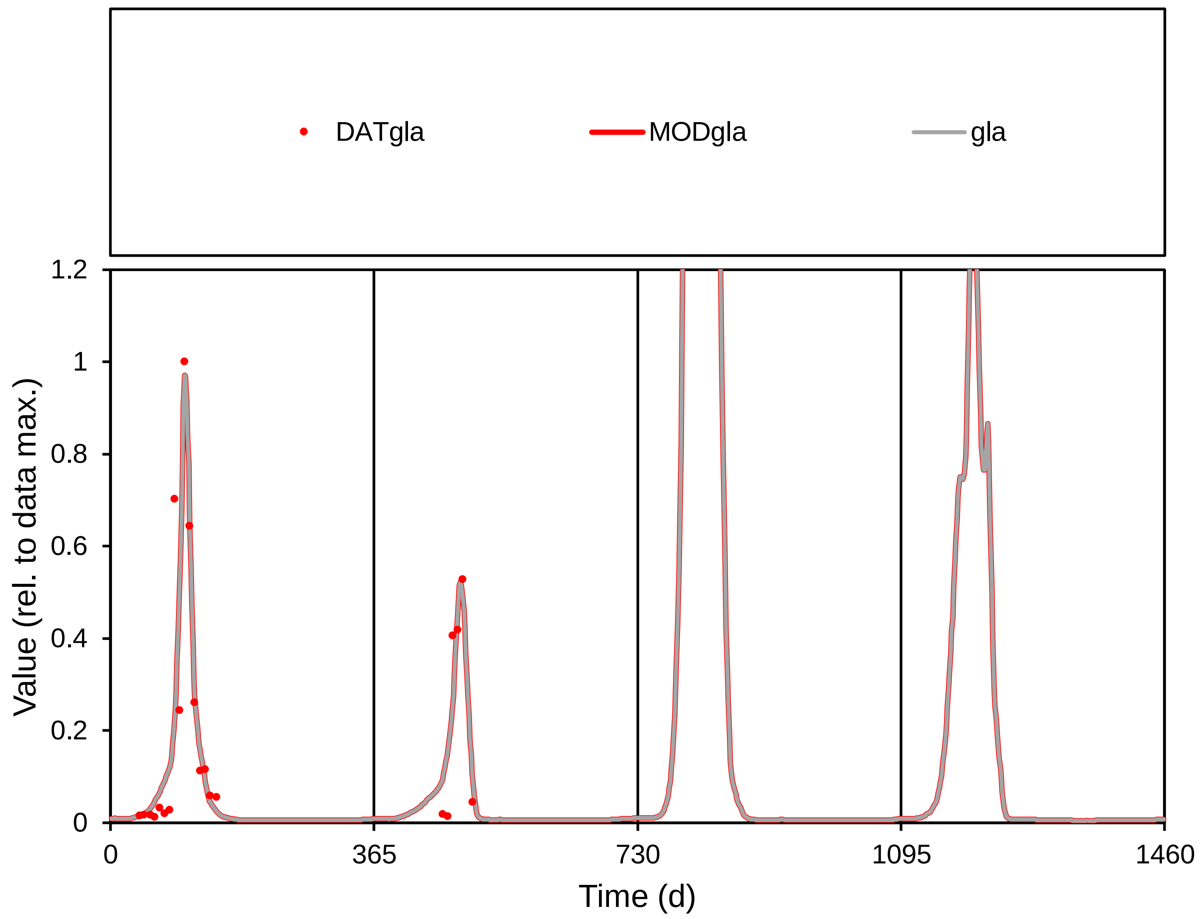

Figure S1(41). Model - data comparison for observation: gla
Normalized to max. value of data. Symbols are observations and lines are model. Red line corresponds to observations (e.g. Chlorophyll *a*), others are sub-components (e.g. individual phytoplankton species). See Tables S19 and S24 for observation and model component IDs, and mapping.

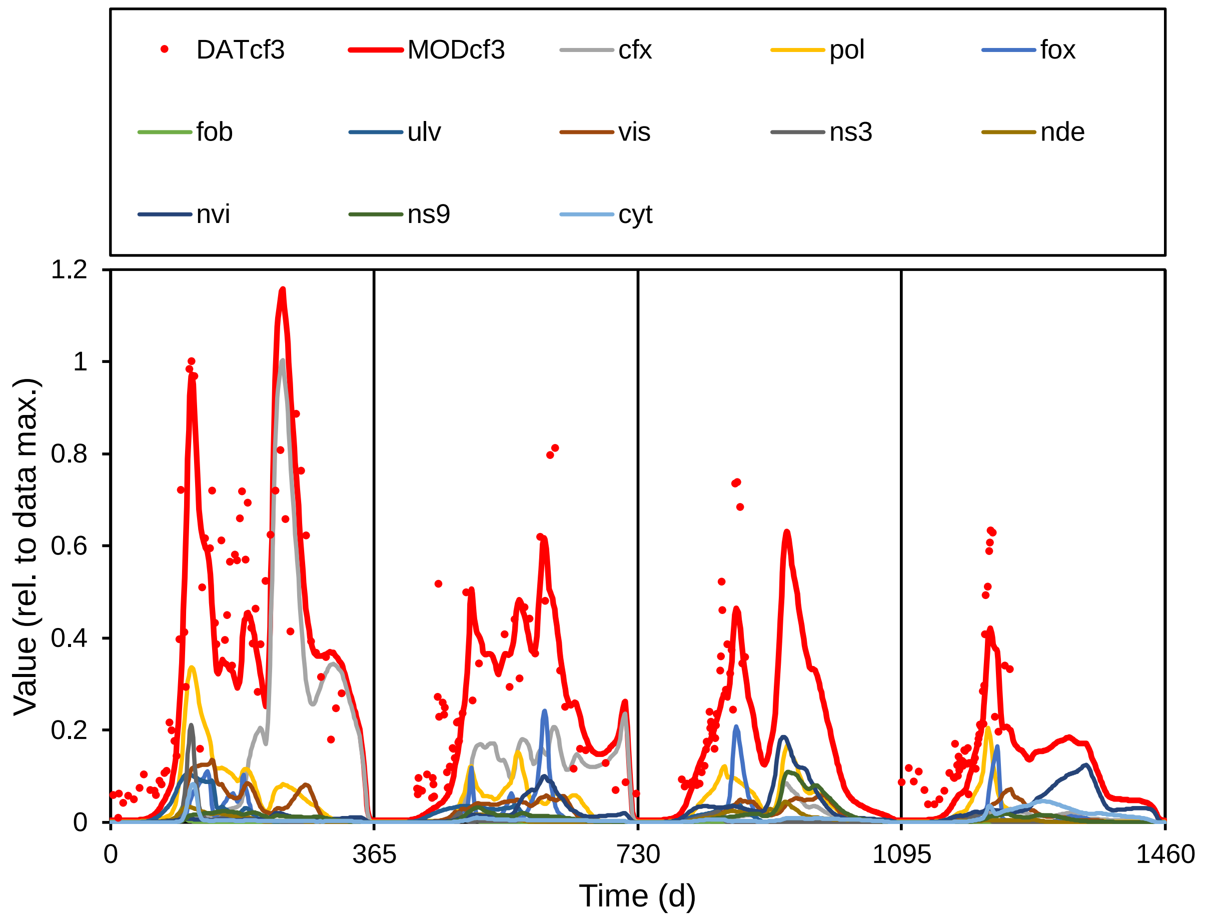

Figure S1(42). Model - data comparison for observation: cf3
Normalized to max. value of data. Symbols are observations and lines are model. Red line corresponds to observations (e.g. Chlorophyll *a*), others are sub-components (e.g. individual phytoplankton species). See Tables S19 and S24 for observation and model component IDs, and mapping.

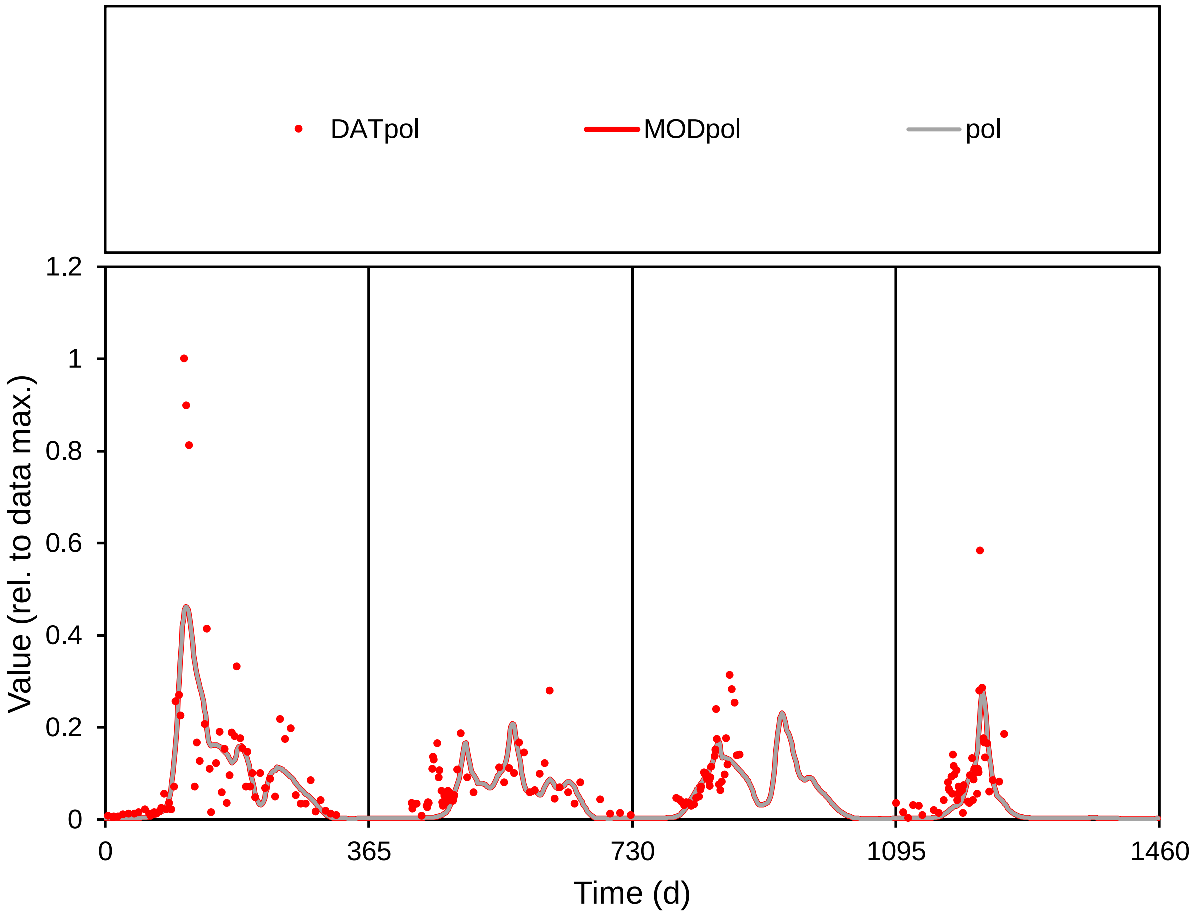

Figure S1(43). Model - data comparison for observation: pol
Normalized to max. value of data. Symbols are observations and lines are model. Red line corresponds to observations (e.g. Chlorophyll *a*), others are sub-components (e.g. individual phytoplankton species). See Tables S19 and S24 for observation and model component IDs, and mapping.

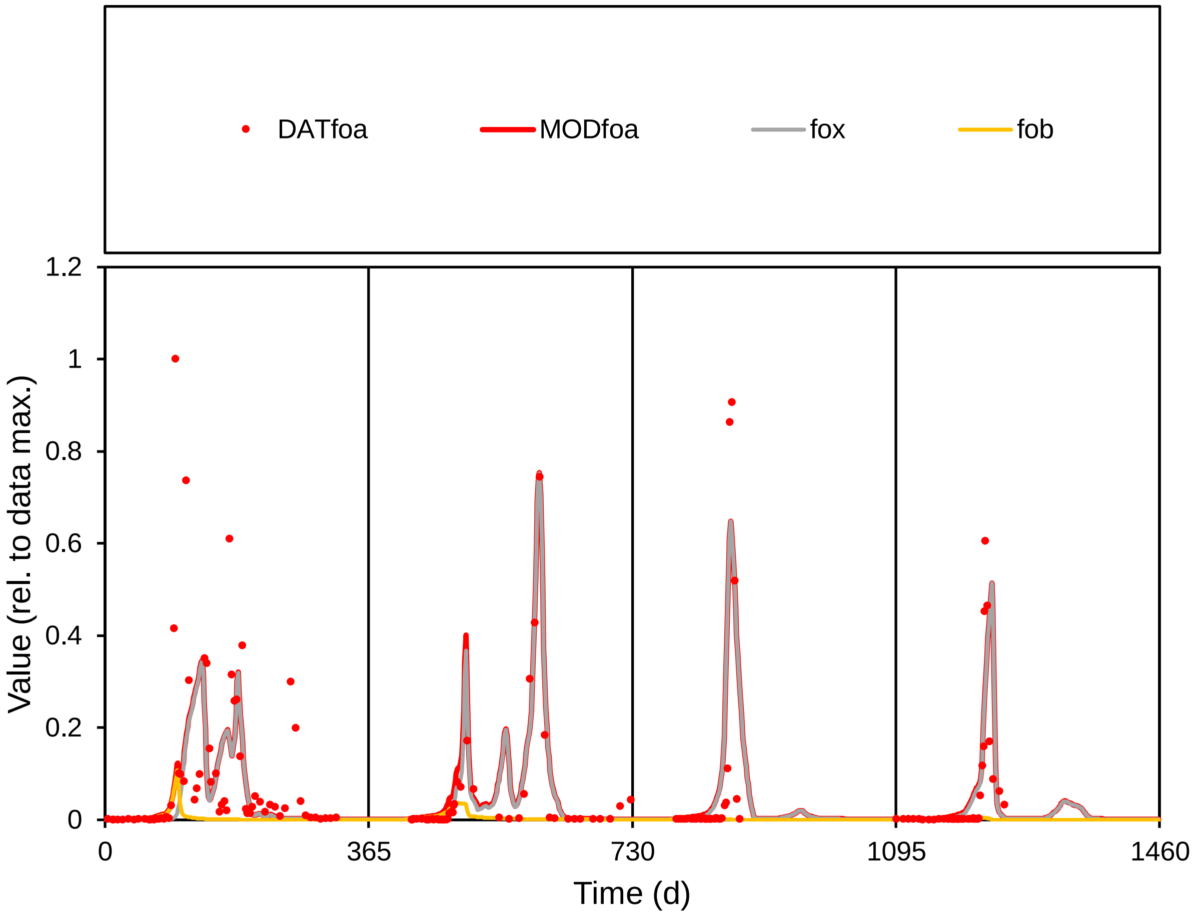

Figure S1(44). Model - data comparison for observation: foa
Normalized to max. value of data. Symbols are observations and lines are model. Red line corresponds to observations (e.g. Chlorophyll *a*), others are sub-components (e.g. individual phytoplankton species). See Tables S19 and S24 for observation and model component IDs, and mapping.

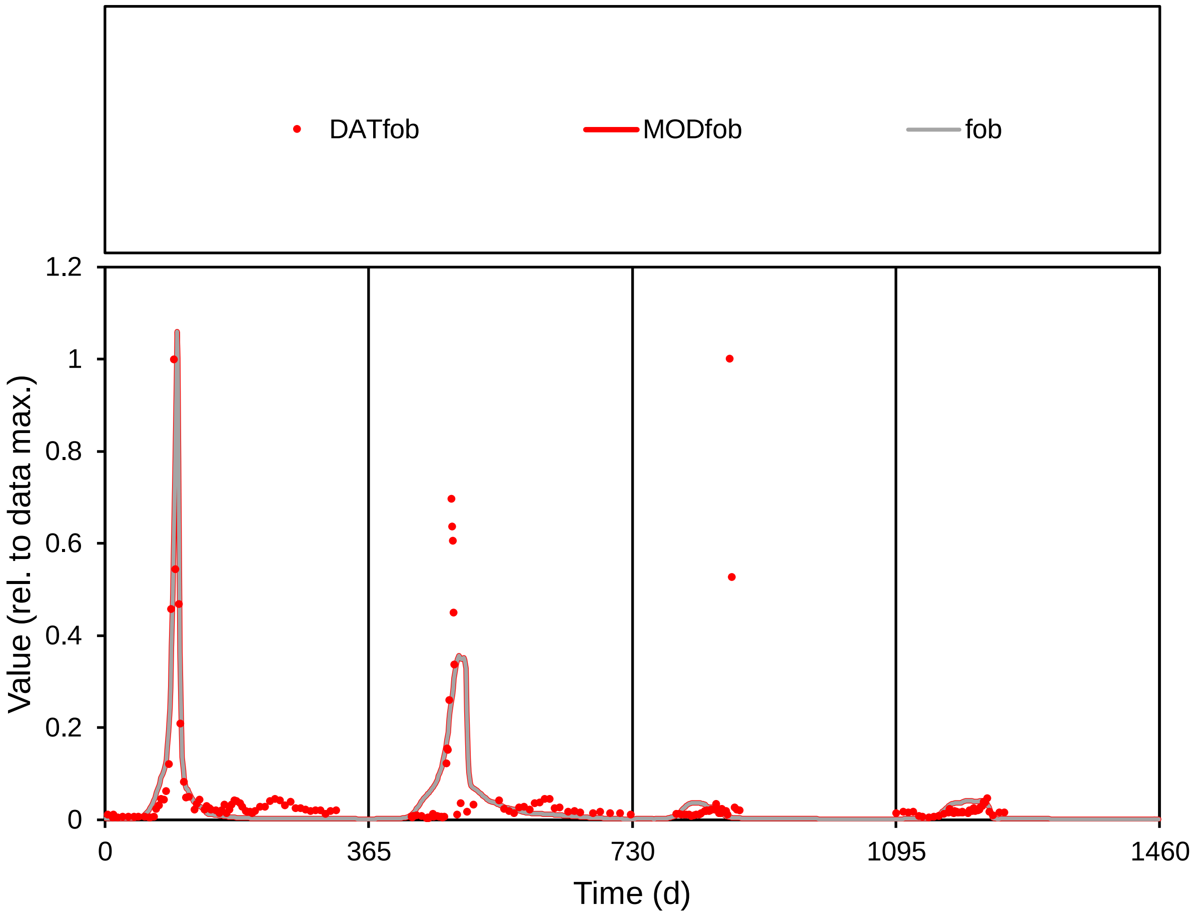

Figure S1(45). Model - data comparison for observation: fob
Normalized to max. value of data. Symbols are observations and lines are model. Red line corresponds to observations (e.g. Chlorophyll *a*), others are sub-components (e.g. individual phytoplankton species). See Tables S19 and S24 for observation and model component IDs, and mapping.

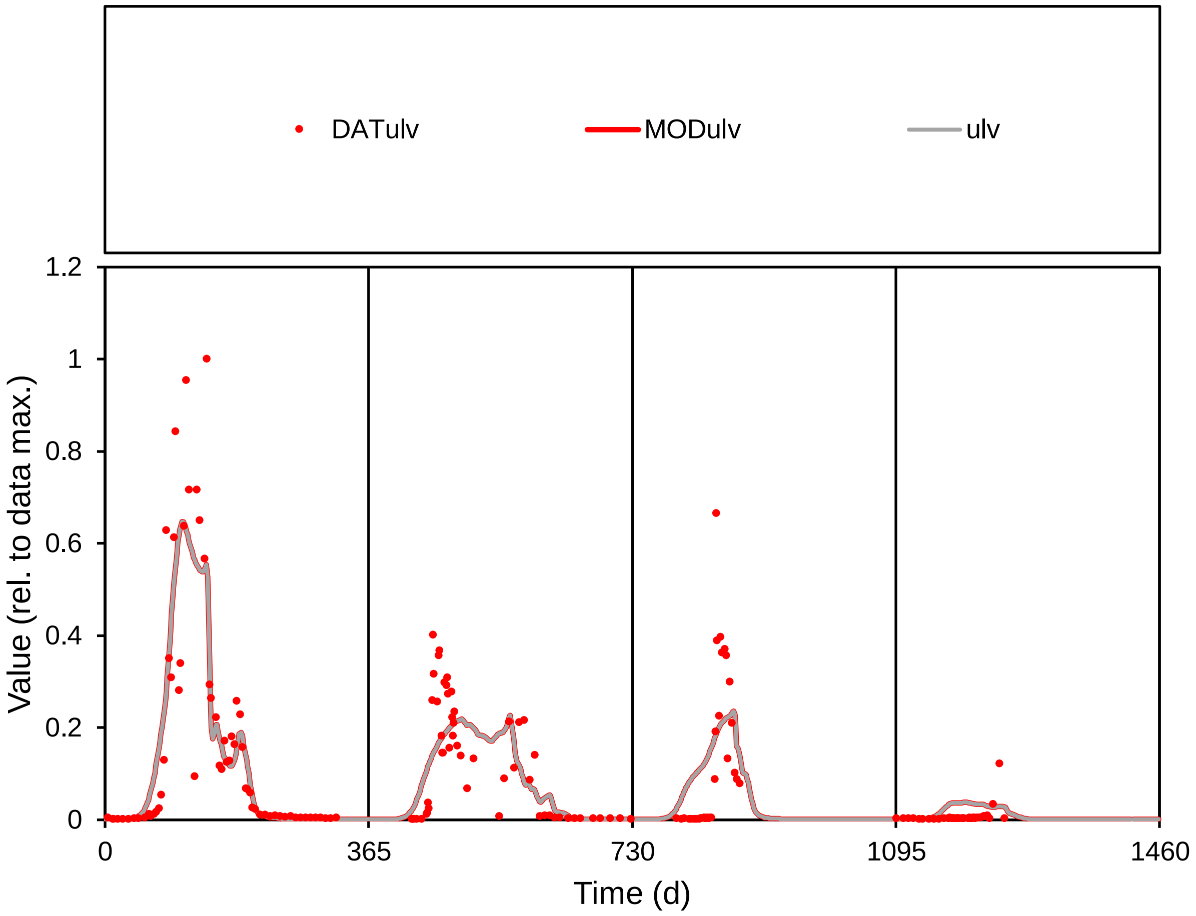

Figure S1(46). Model - data comparison for observation: ulv
Normalized to max. value of data. Symbols are observations and lines are model. Red line corresponds to observations (e.g. Chlorophyll *a*), others are sub-components (e.g. individual phytoplankton species). See Tables S19 and S24 for observation and model component IDs, and mapping.

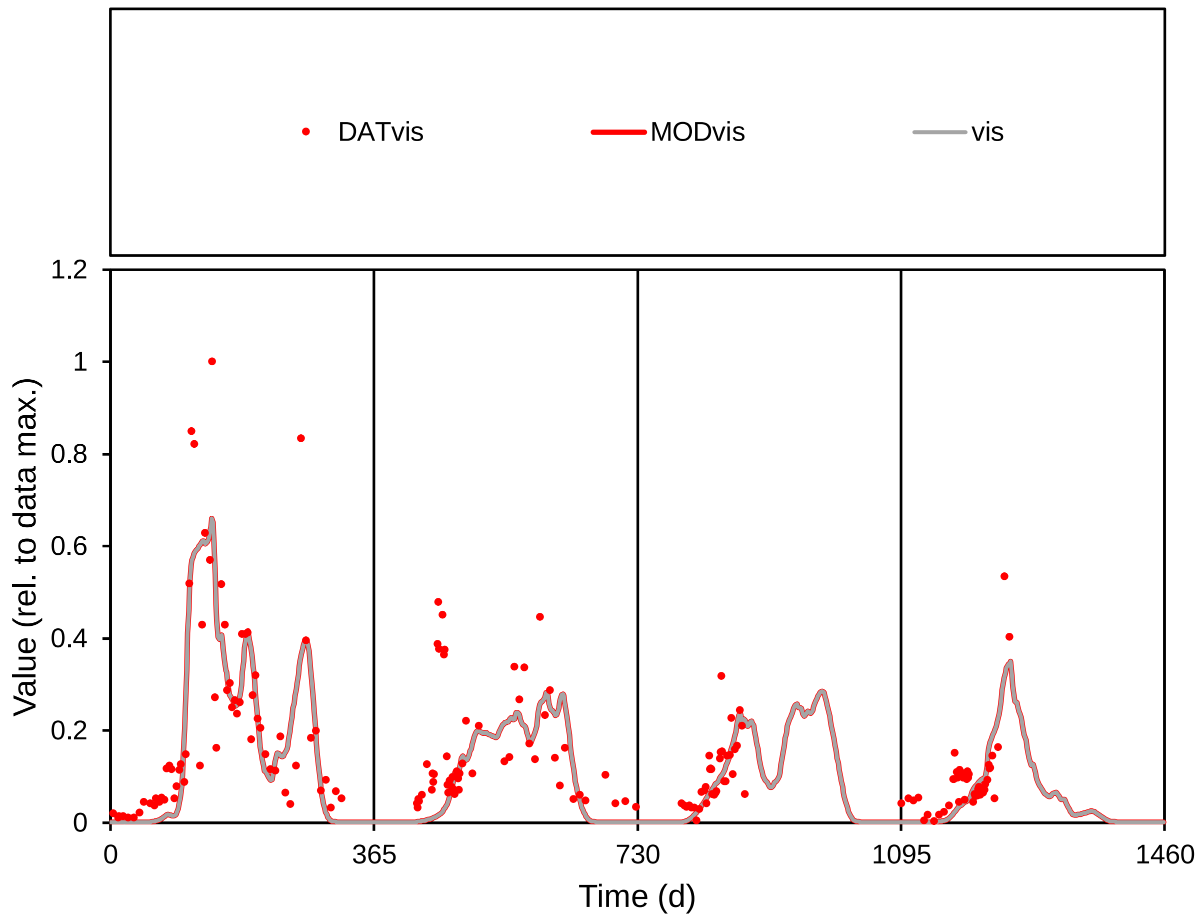

Figure S1(47). Model - data comparison for observation: vis
Normalized to max. value of data. Symbols are observations and lines are model. Red line corresponds to observations (e.g. Chlorophyll *a*), others are sub-components (e.g. individual phytoplankton species). See Tables S19 and S24 for observation and model component IDs, and mapping.

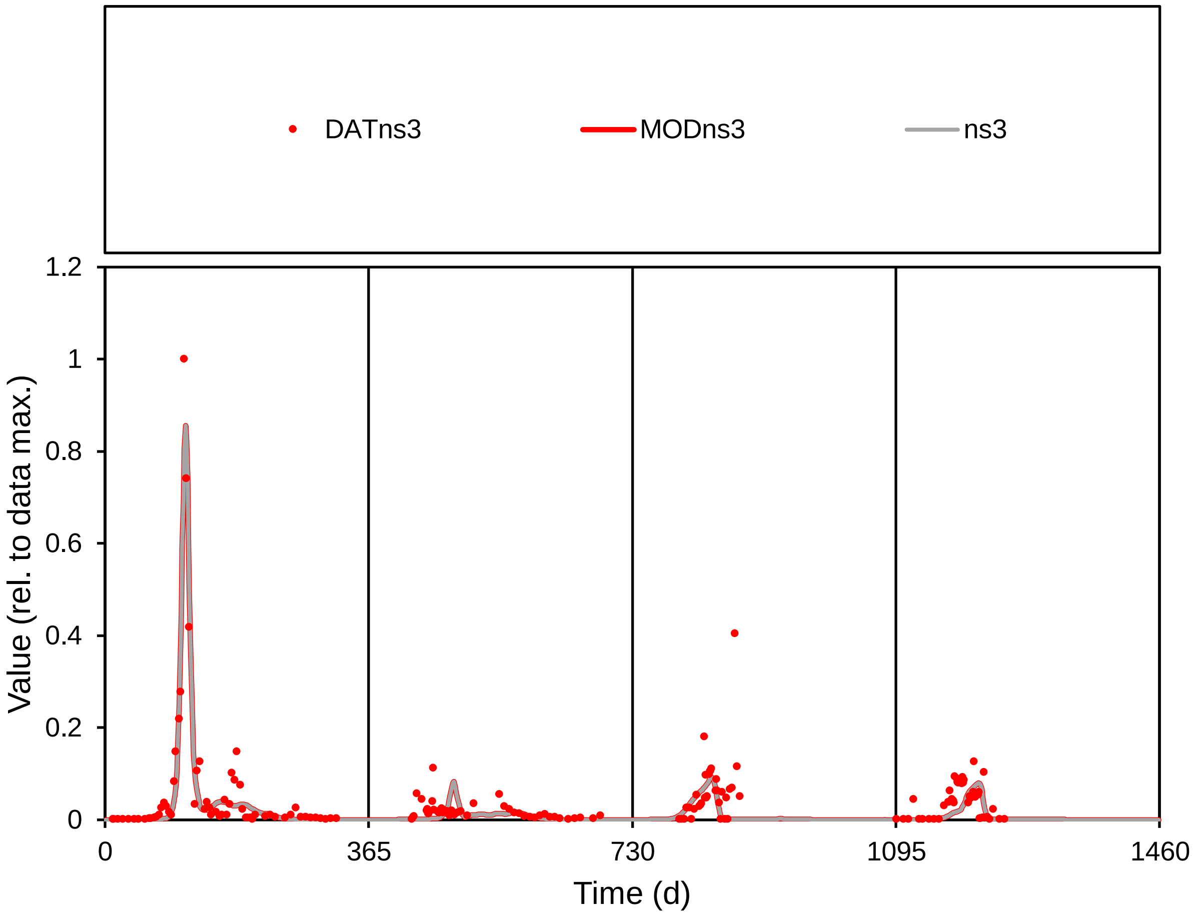

Figure S1(48). Model - data comparison for observation: ns3
Normalized to max. value of data. Symbols are observations and lines are model. Red line corresponds to observations (e.g. Chlorophyll *a*), others are sub-components (e.g. individual phytoplankton species). See Tables S19 and S24 for observation and model component IDs, and mapping.

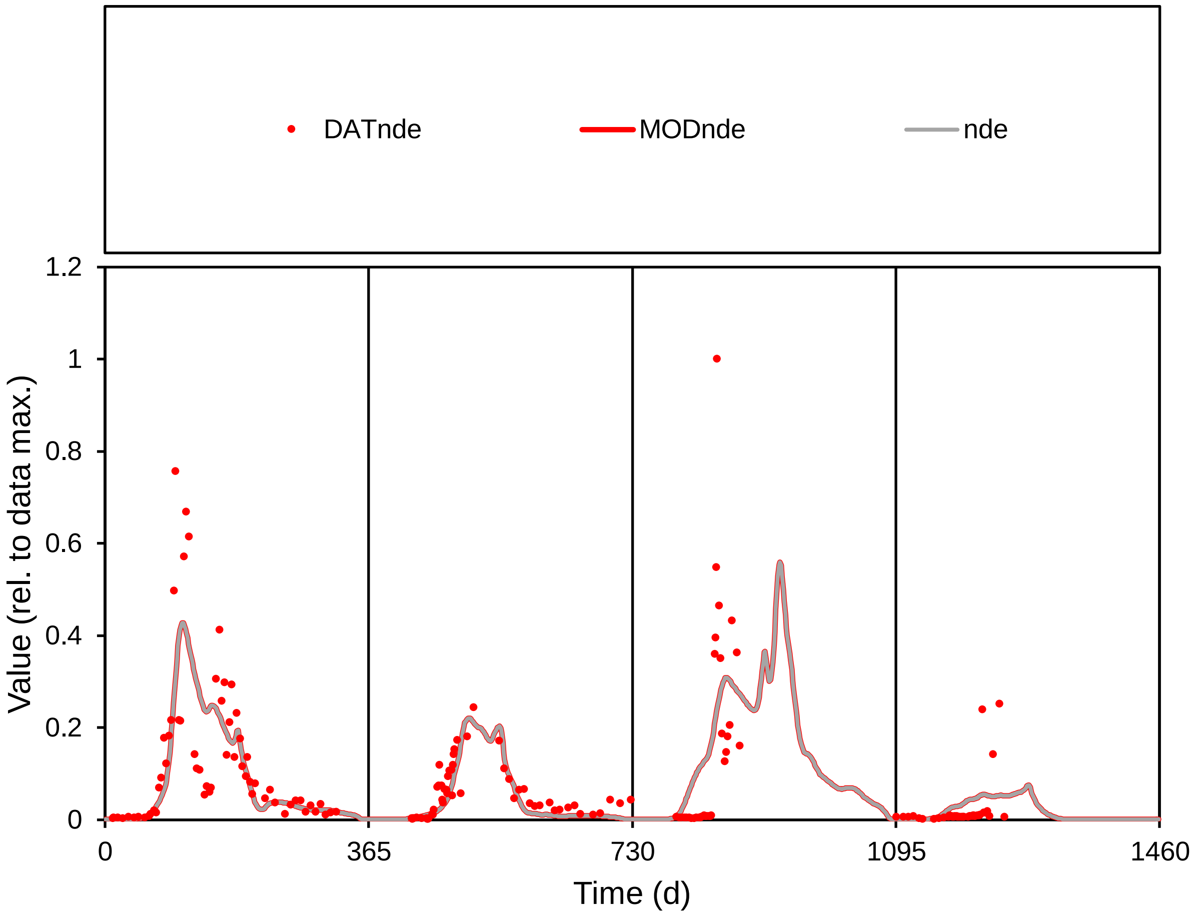

Figure S1(49). Model - data comparison for observation: nde
Normalized to max. value of data. Symbols are observations and lines are model. Red line corresponds to observations (e.g. Chlorophyll *a*), others are sub-components (e.g. individual phytoplankton species). See Tables S19 and S24 for observation and model component IDs, and mapping.

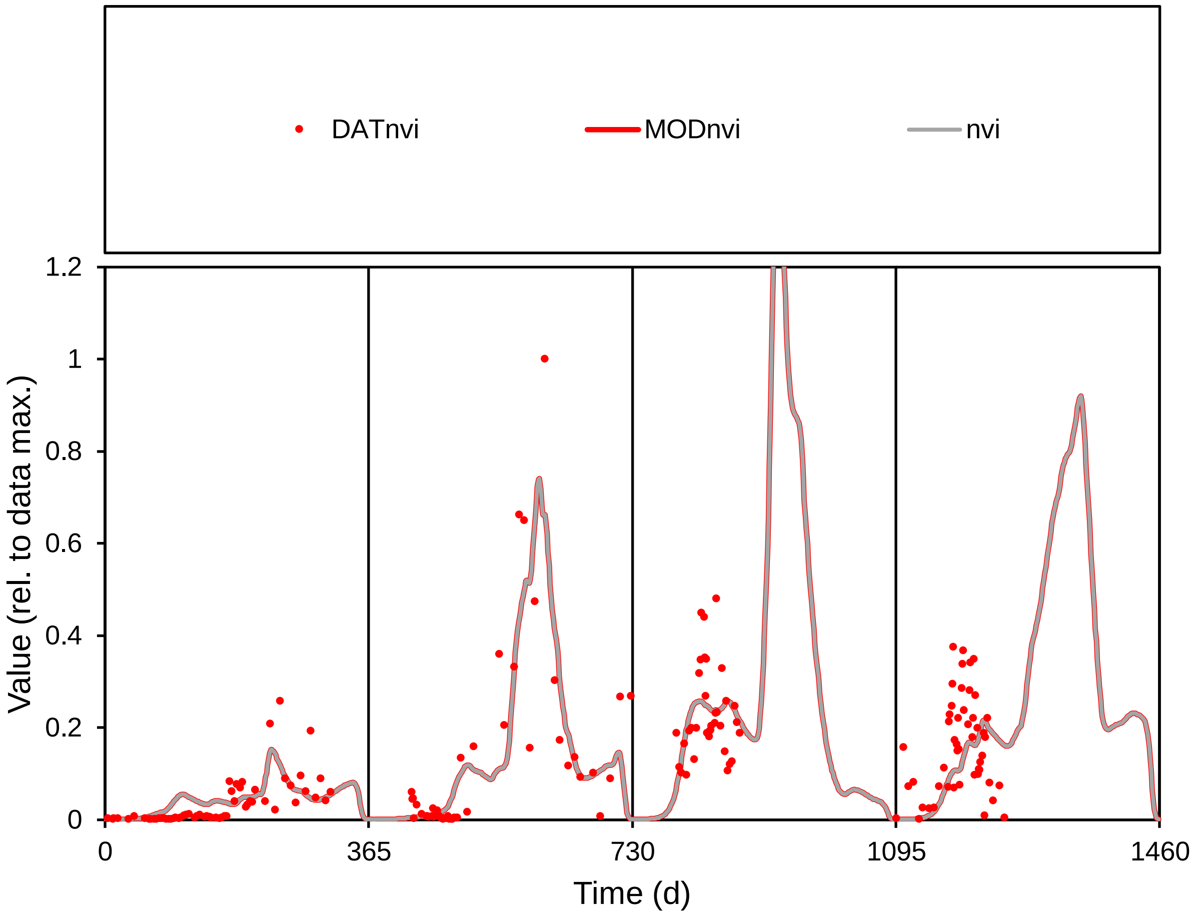

Figure S1(50). Model - data comparison for observation: nvi
Normalized to max. value of data. Symbols are observations and lines are model. Red line corresponds to observations (e.g. Chlorophyll *a*), others are sub-components (e.g. individual phytoplankton species). See Tables S19 and S24 for observation and model component IDs, and mapping.

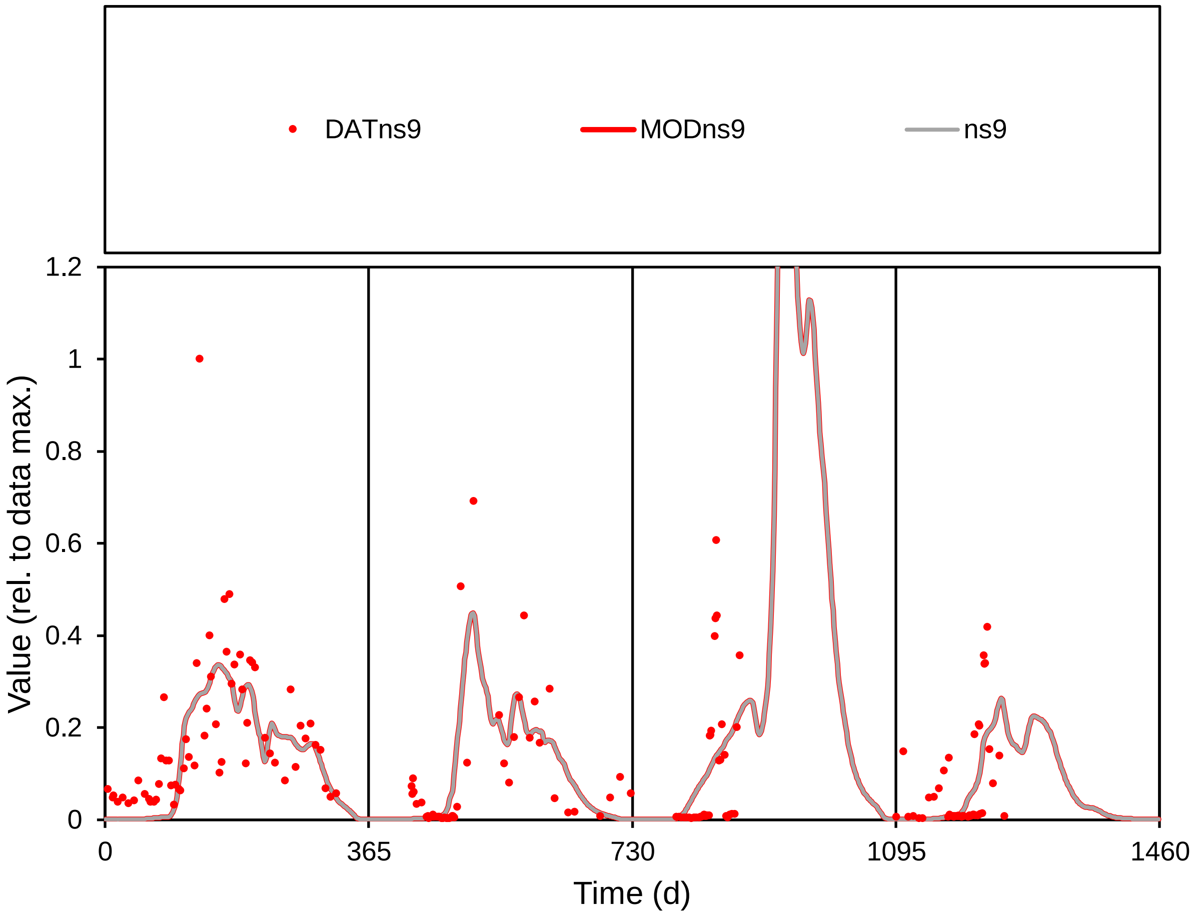

Figure S1(51). Model - data comparison for observation: ns9
Normalized to max. value of data. Symbols are observations and lines are model. Red line corresponds to observations (e.g. Chlorophyll *a*), others are sub-components (e.g. individual phytoplankton species). See Tables S19 and S24 for observation and model component IDs, and mapping.

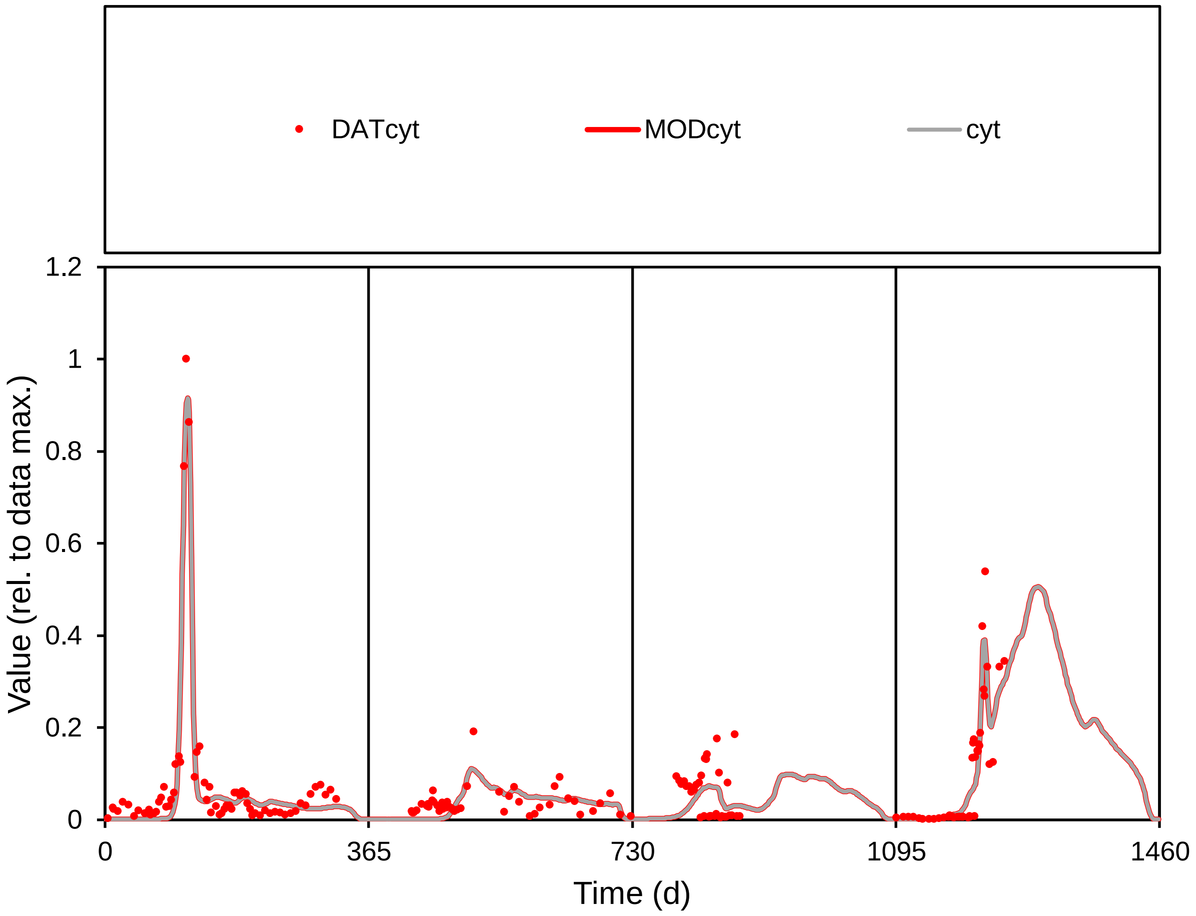

Figure S1(52). Model - data comparison for observation: cyt
Normalized to max. value of data. Symbols are observations and lines are model. Red line corresponds to observations (e.g. Chlorophyll *a*), others are sub-components (e.g. individual phytoplankton species). See Tables S19 and S24 for observation and model component IDs, and mapping.

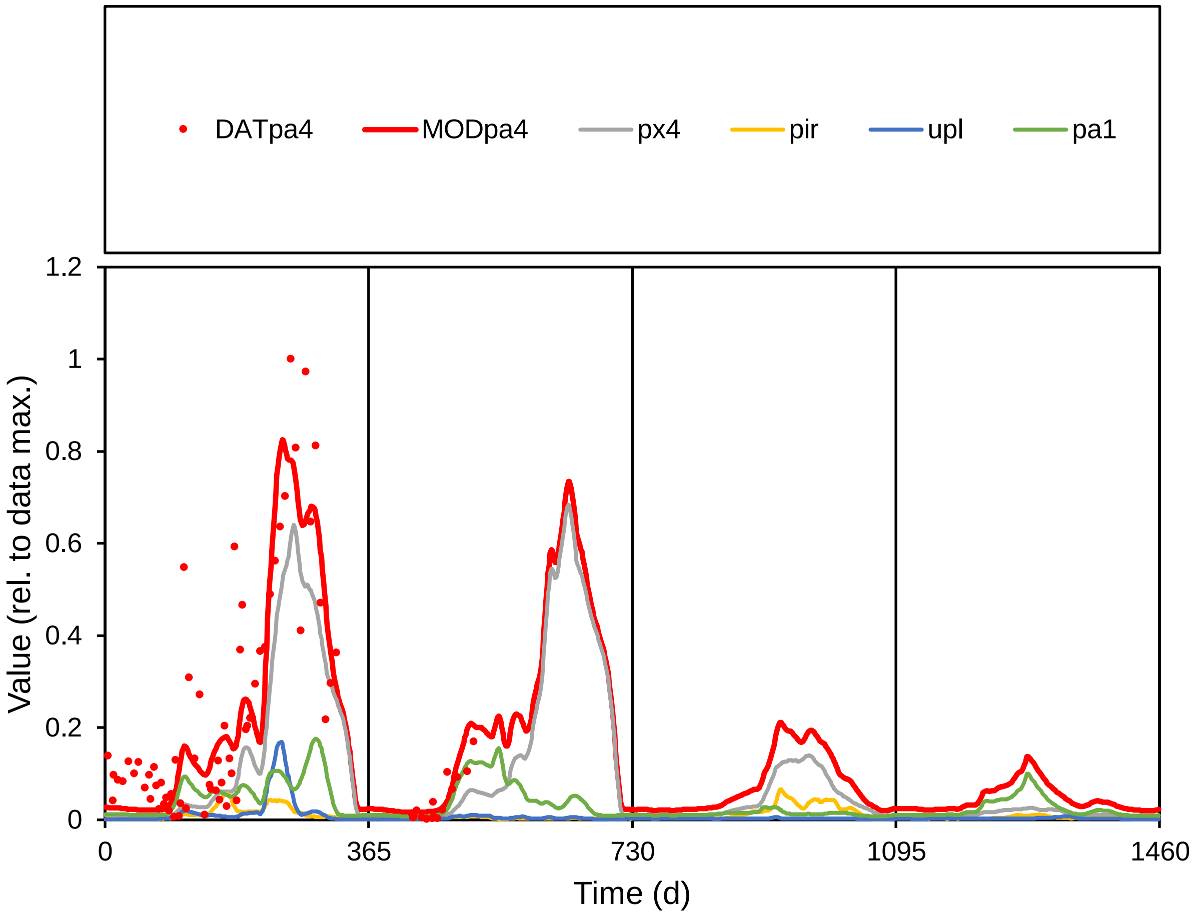

Figure S1(53). Model - data comparison for observation: pa4
Normalized to max. value of data. Symbols are observations and lines are model. Red line corresponds to observations (e.g. Chlorophyll *a*), others are sub-components (e.g. individual phytoplankton species). See Tables S19 and S24 for observation and model component IDs, and mapping.

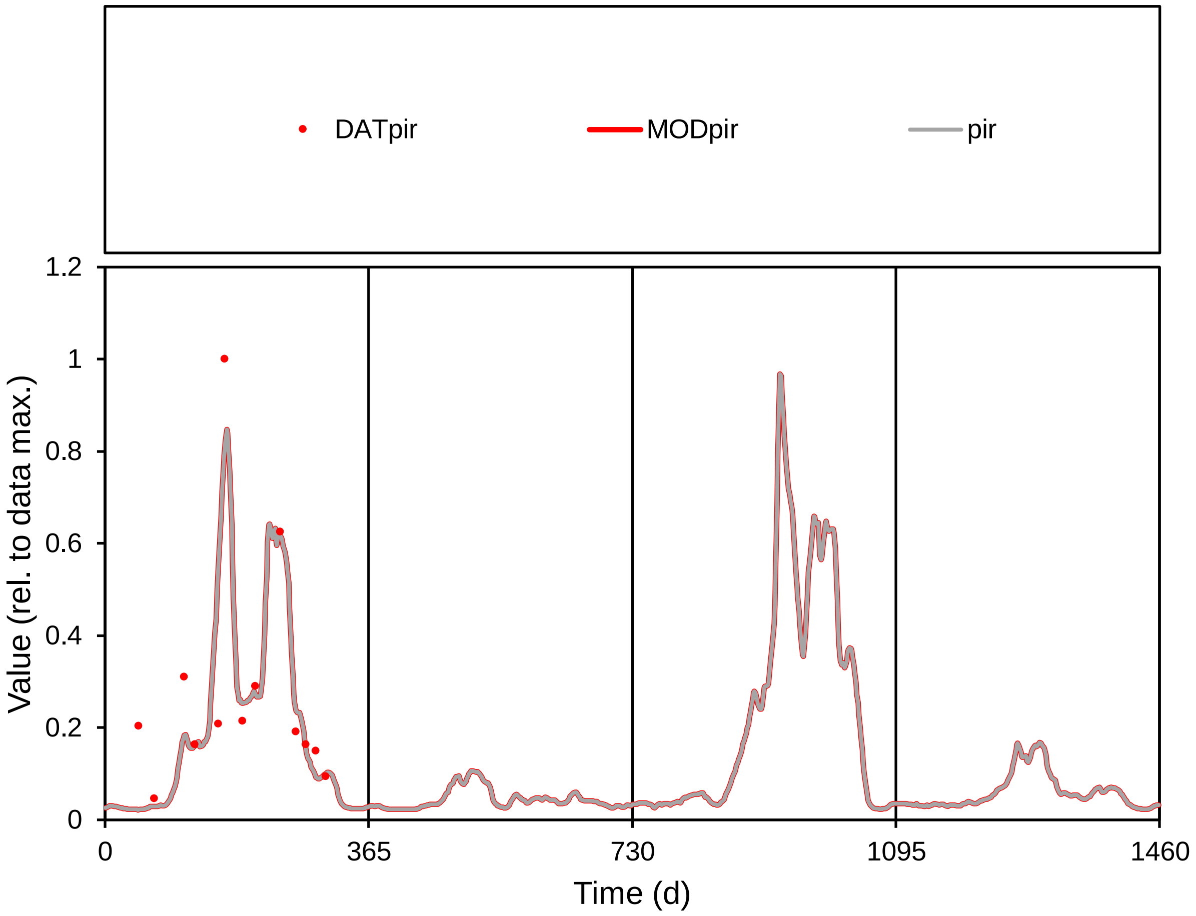

Figure S1(54). Model - data comparison for observation: pir
Normalized to max. value of data. Symbols are observations and lines are model. Red line corresponds to observations (e.g. Chlorophyll *a*), others are sub-components (e.g. individual phytoplankton species). See Tables S19 and S24 for observation and model component IDs, and mapping.

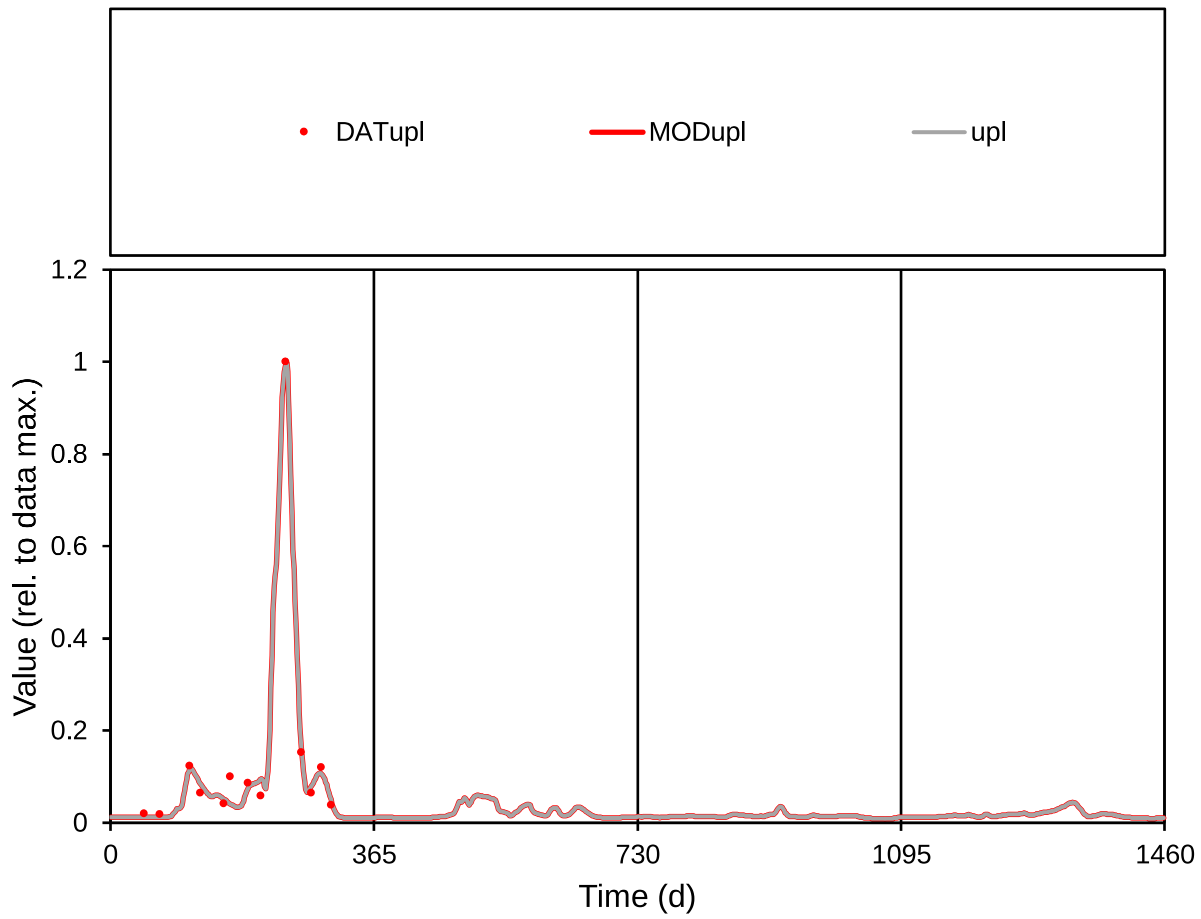

Figure S1(55). Model - data comparison for observation: upl
Normalized to max. value of data. Symbols are observations and lines are model. Red line corresponds to observations (e.g. Chlorophyll *a*), others are sub-components (e.g. individual phytoplankton species). See Tables S19 and S24 for observation and model component IDs, and mapping.

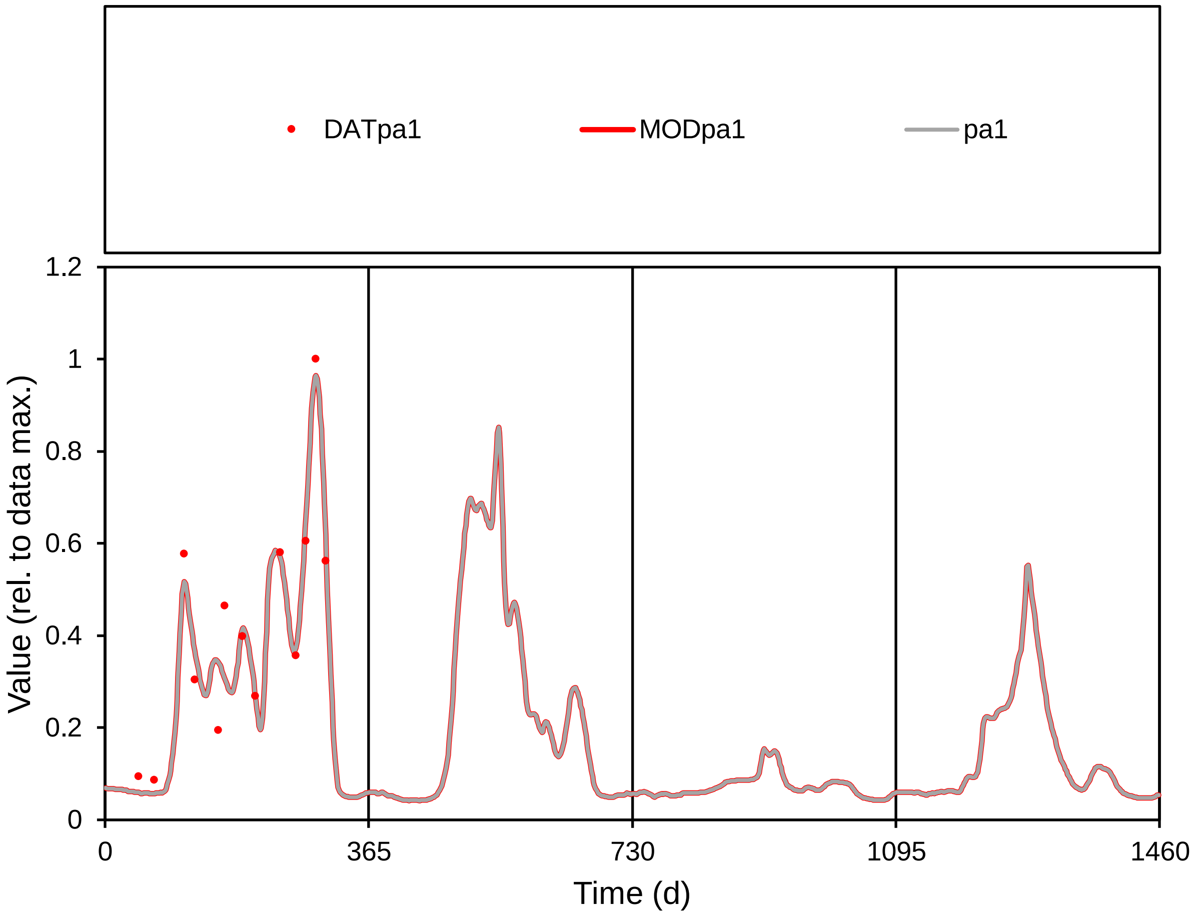

Figure S1(56). Model - data comparison for observation: pa1
Normalized to max. value of data. Symbols are observations and lines are model. Red line corresponds to observations (e.g. Chlorophyll *a*), others are sub-components (e.g. individual phytoplankton species). See Tables S19 and S24 for observation and model component IDs, and mapping.

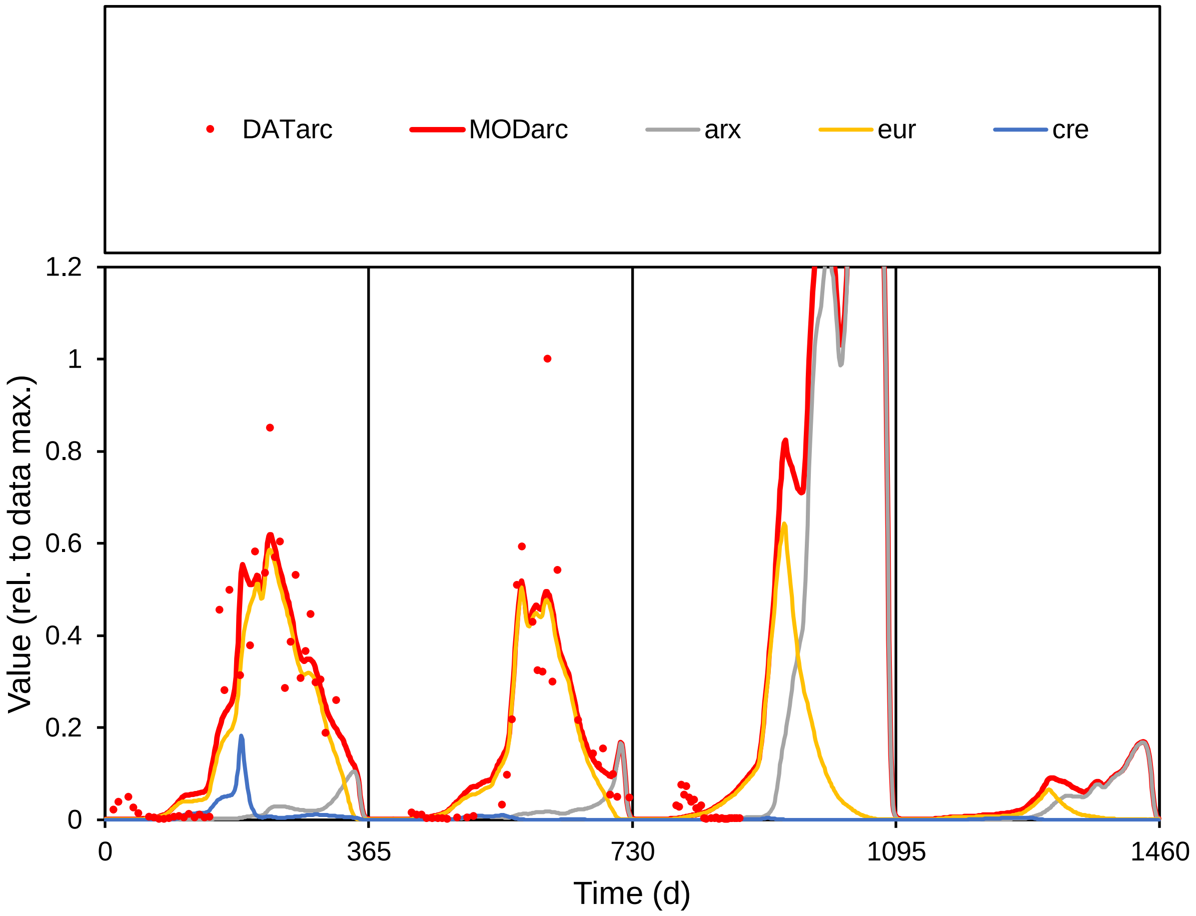

Figure S1(57). Model - data comparison for observation: arc
Normalized to max. value of data. Symbols are observations and lines are model. Red line corresponds to observations (e.g. Chlorophyll *a*), others are sub-components (e.g. individual phytoplankton species). See Tables S19 and S24 for observation and model component IDs, and mapping.

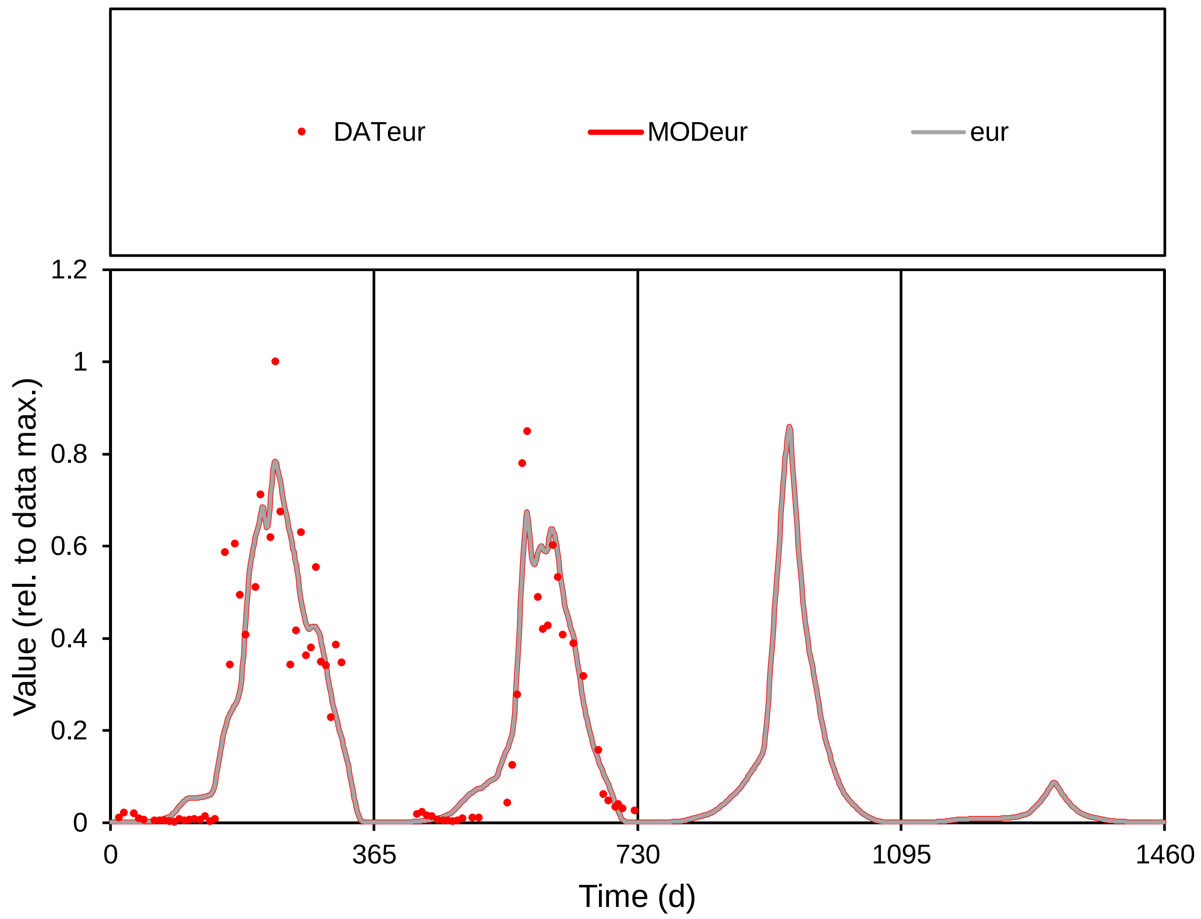

Figure S1(58). Model - data comparison for observation: eur
Normalized to max. value of data. Symbols are observations and lines are model. Red line corresponds to observations (e.g. Chlorophyll *a*), others are sub-components (e.g. individual phytoplankton species). See Tables S19 and S24 for observation and model component IDs, and mapping.

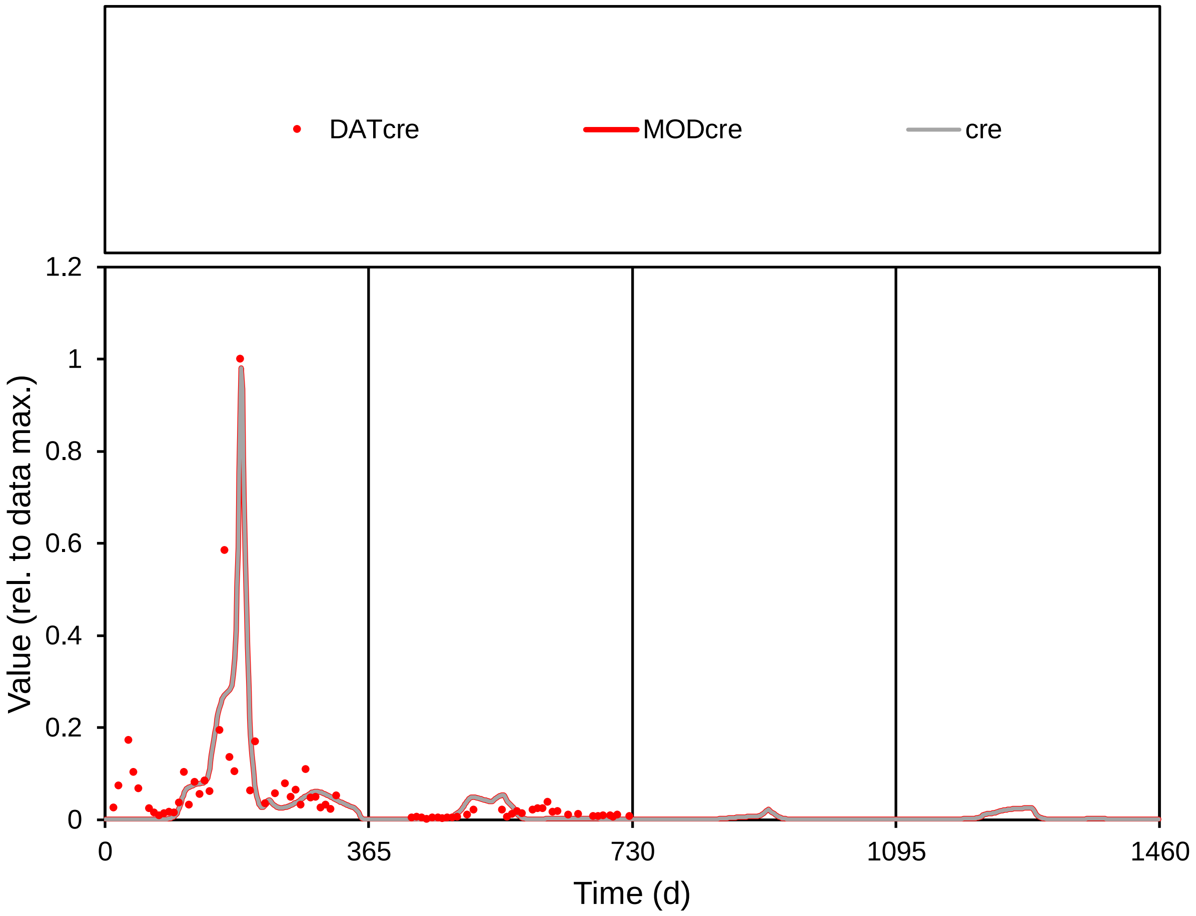

Figure S1(59). Model - data comparison for observation: cre
Normalized to max. value of data. Symbols are observations and lines are model. Red line corresponds to observations (e.g. Chlorophyll *a*), others are sub-components (e.g. individual phytoplankton species). See Tables S19 and S24 for observation and model component IDs, and mapping.

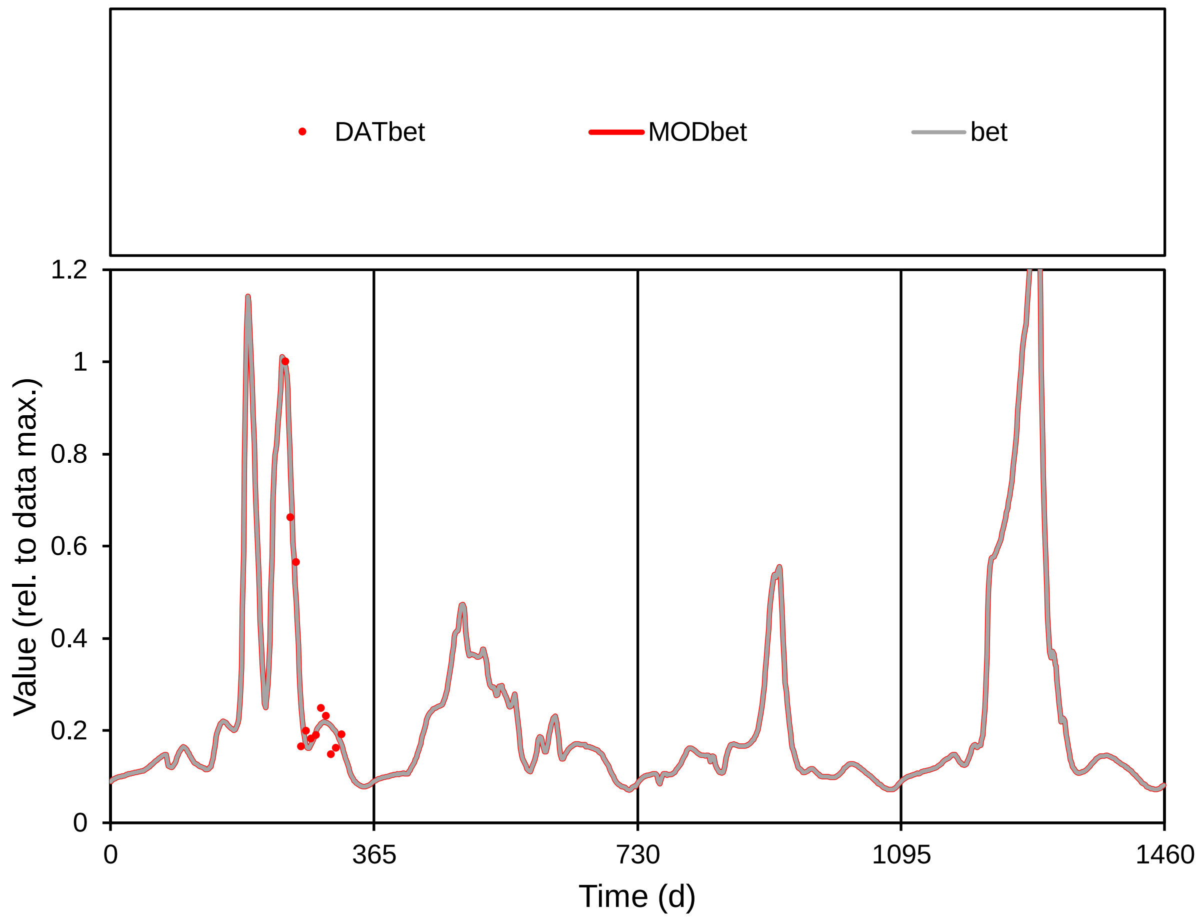

Figure S1(60). Model - data comparison for observation: bet
Normalized to max. value of data. Symbols are observations and lines are model. Red line corresponds to observations (e.g. Chlorophyll *a*), others are sub-components (e.g. individual phytoplankton species). See Tables S19 and S24 for observation and model component IDs, and mapping.

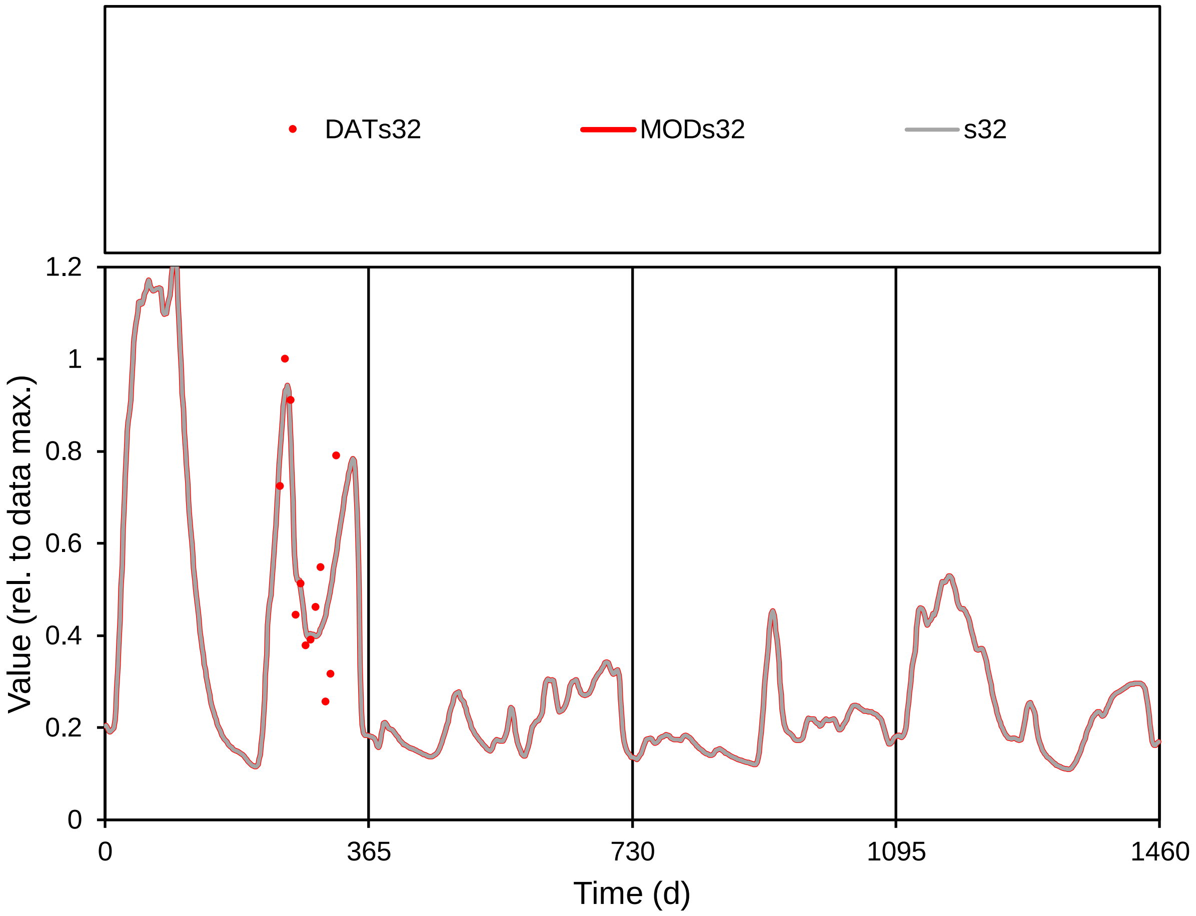

Figure S1(61). Model - data comparison for observation: s32
Normalized to max. value of data. Symbols are observations and lines are model. Red line corresponds to observations (e.g. Chlorophyll *a*), others are sub-components (e.g. individual phytoplankton species). See Tables S19 and S24 for observation and model component IDs, and mapping.

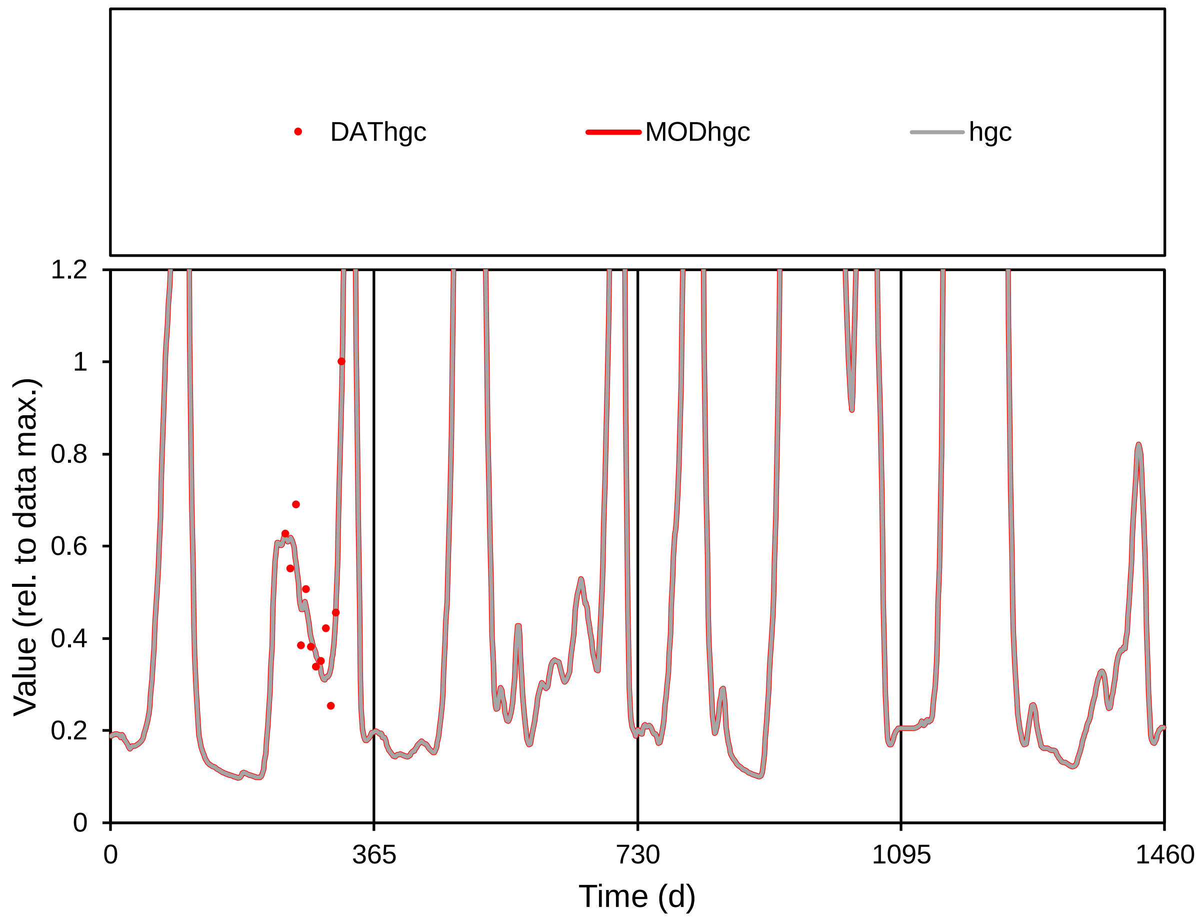

Figure S1(62). Model - data comparison for observation: hgc
Normalized to max. value of data. Symbols are observations and lines are model. Red line corresponds to observations (e.g. Chlorophyll *a*), others are sub-components (e.g. individual phytoplankton species). See Tables S19 and S24 for observation and model component IDs, and mapping.

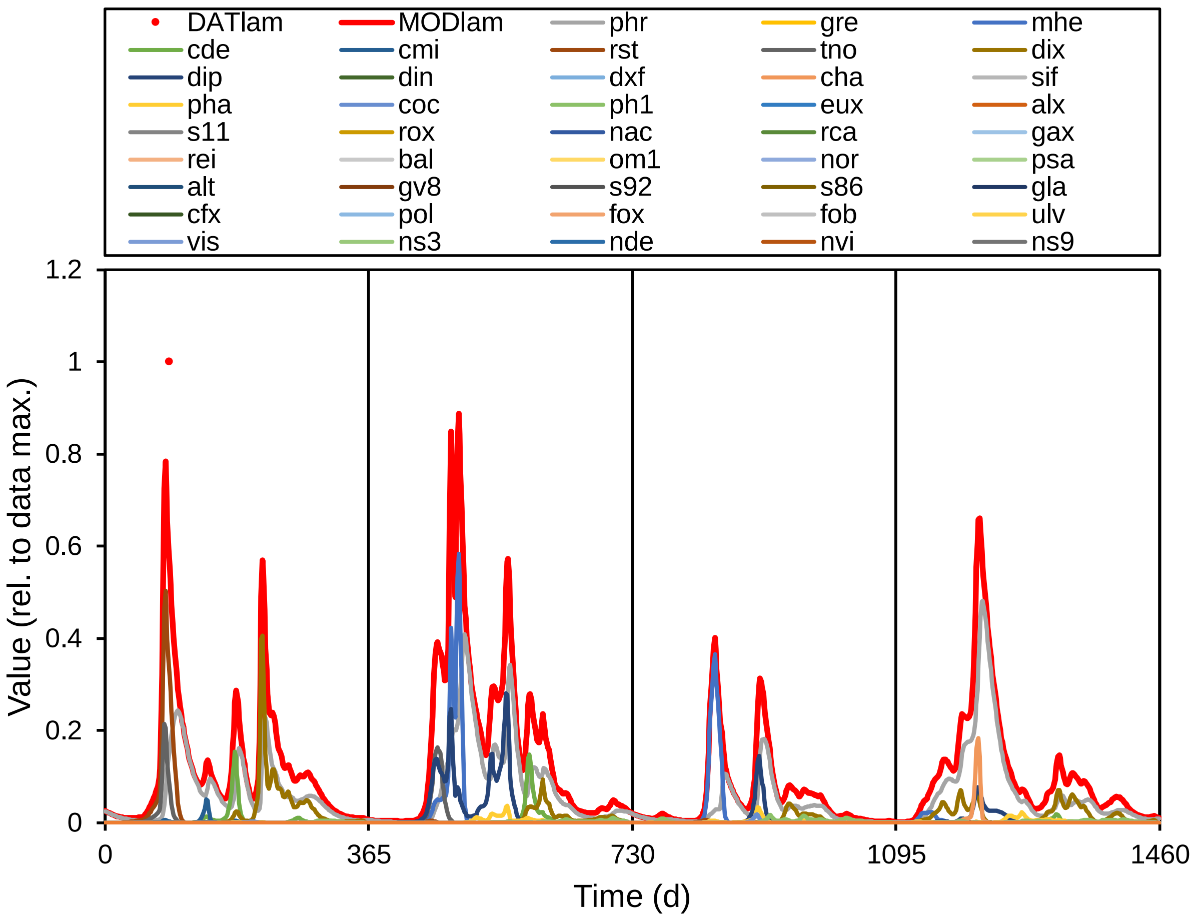

Figure S1(63). Model - data comparison for observation: lam

Normalized to max. value of data. Symbols are observations and lines are model. Red line corresponds to observations (e.g. Chlorophyll *a*), others are sub-components (e.g. individual phytoplankton species). See Tables S19 and S24 for observation and model component IDs, and mapping.

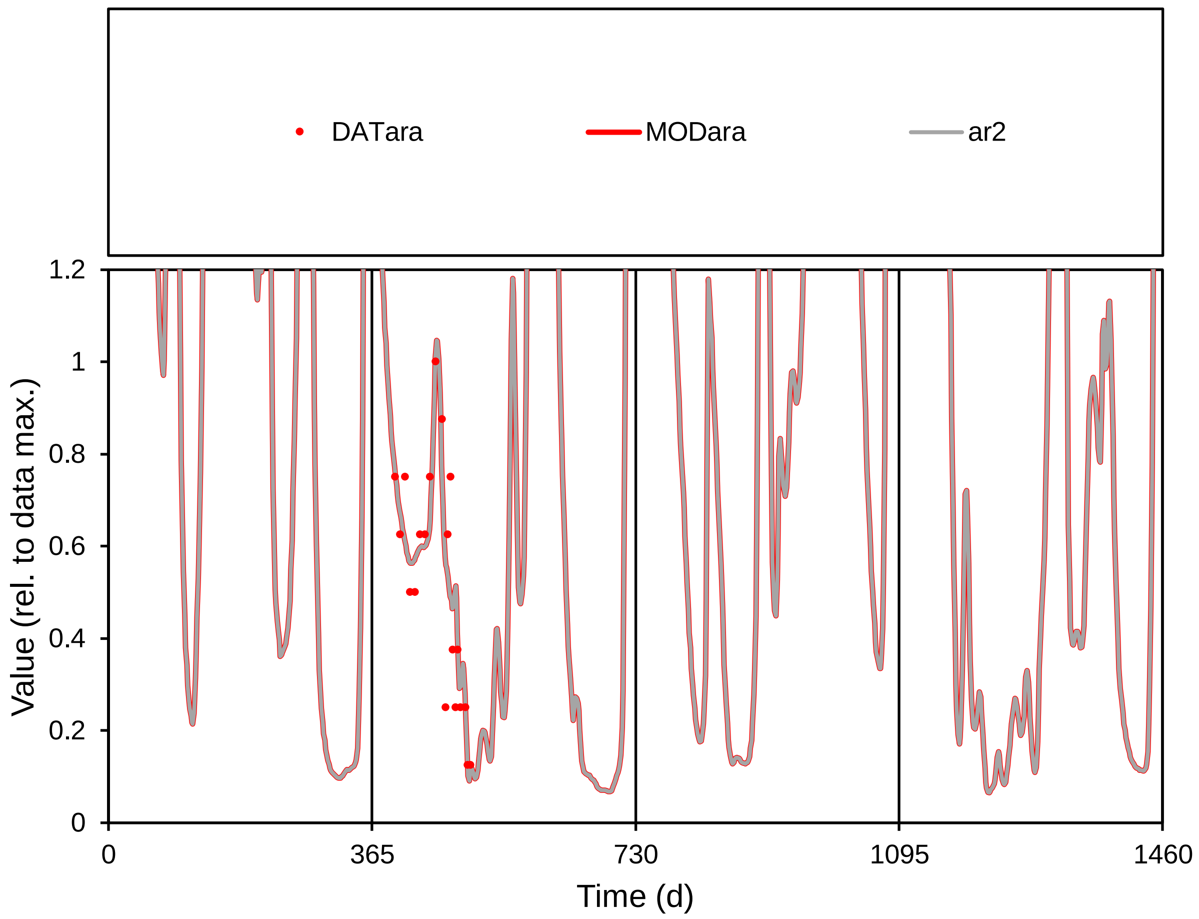

Figure S1(64). Model - data comparison for observation: ara
Normalized to max. value of data. Symbols are observations and lines are model. Red line corresponds to observations (e.g. Chlorophyll *a*), others are sub-components (e.g. individual phytoplankton species). See Tables S19 and S24 for observation and model component IDs, and mapping.

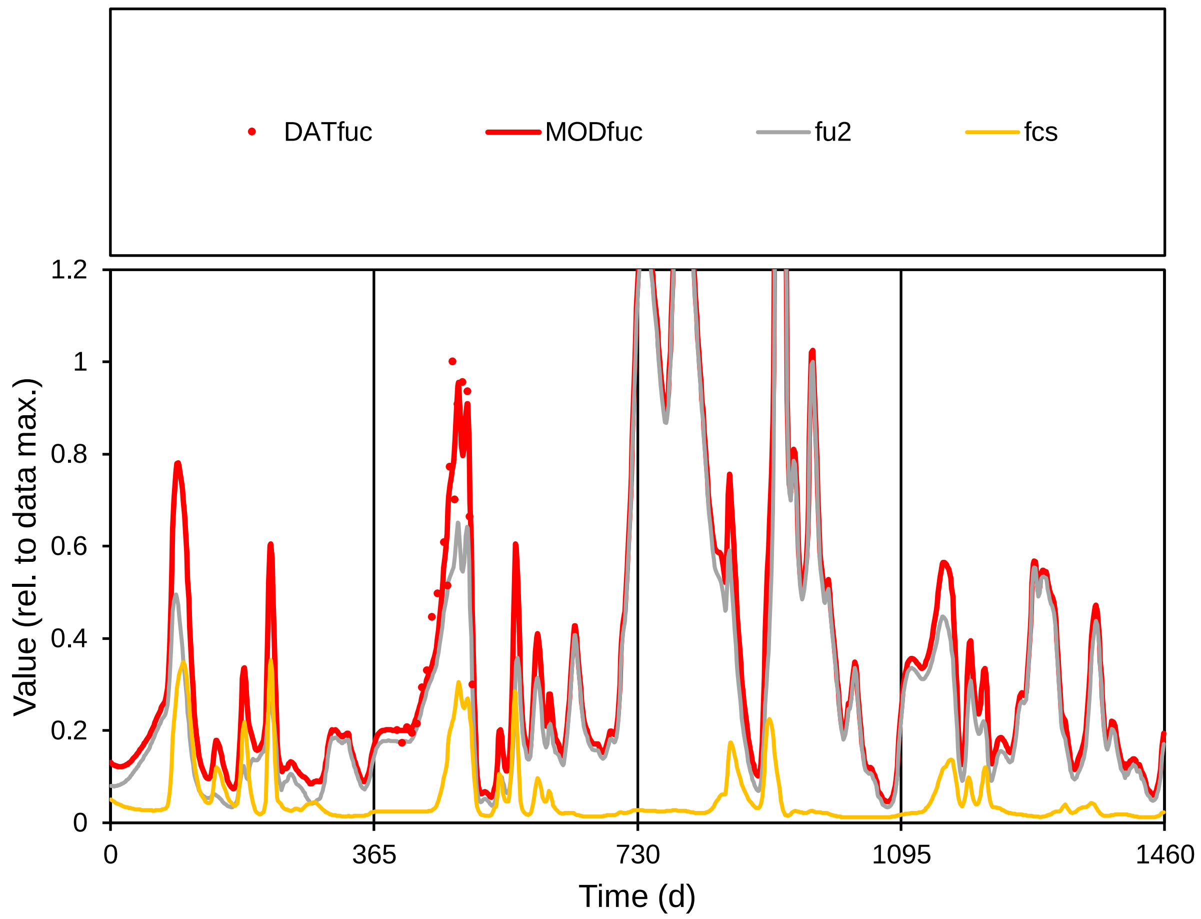

Figure S1(65). Model - data comparison for observation: fuc
Normalized to max. value of data. Symbols are observations and lines are model. Red line corresponds to observations (e.g. Chlorophyll *a*), others are sub-components (e.g. individual phytoplankton species). See Tables S19 and S24 for observation and model component IDs, and mapping.

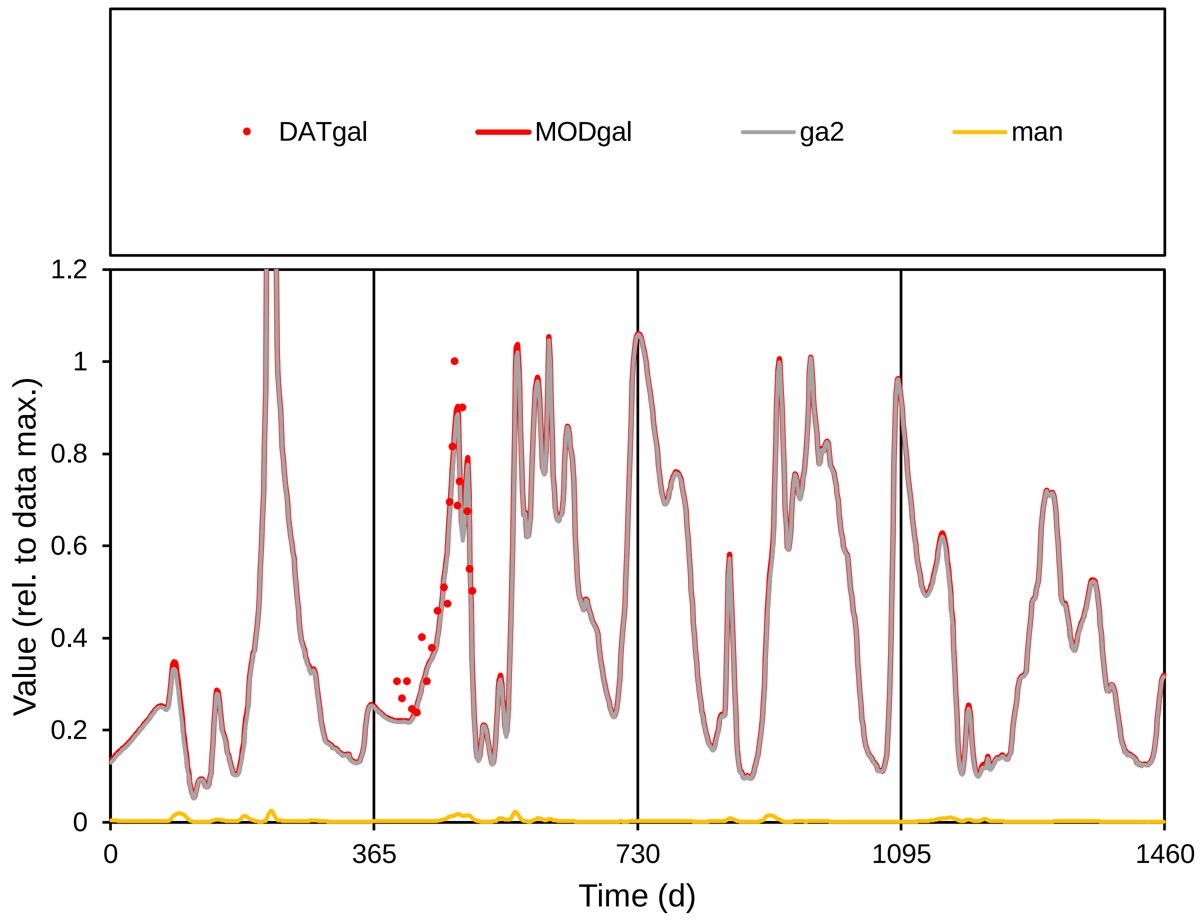

Figure S1(66). Model - data comparison for observation: gal
Normalized to max. value of data. Symbols are observations and lines are model. Red line corresponds to observations (e.g. Chlorophyll *a*), others are sub-components (e.g. individual phytoplankton species). See Tables S19 and S24 for observation and model component IDs, and mapping.

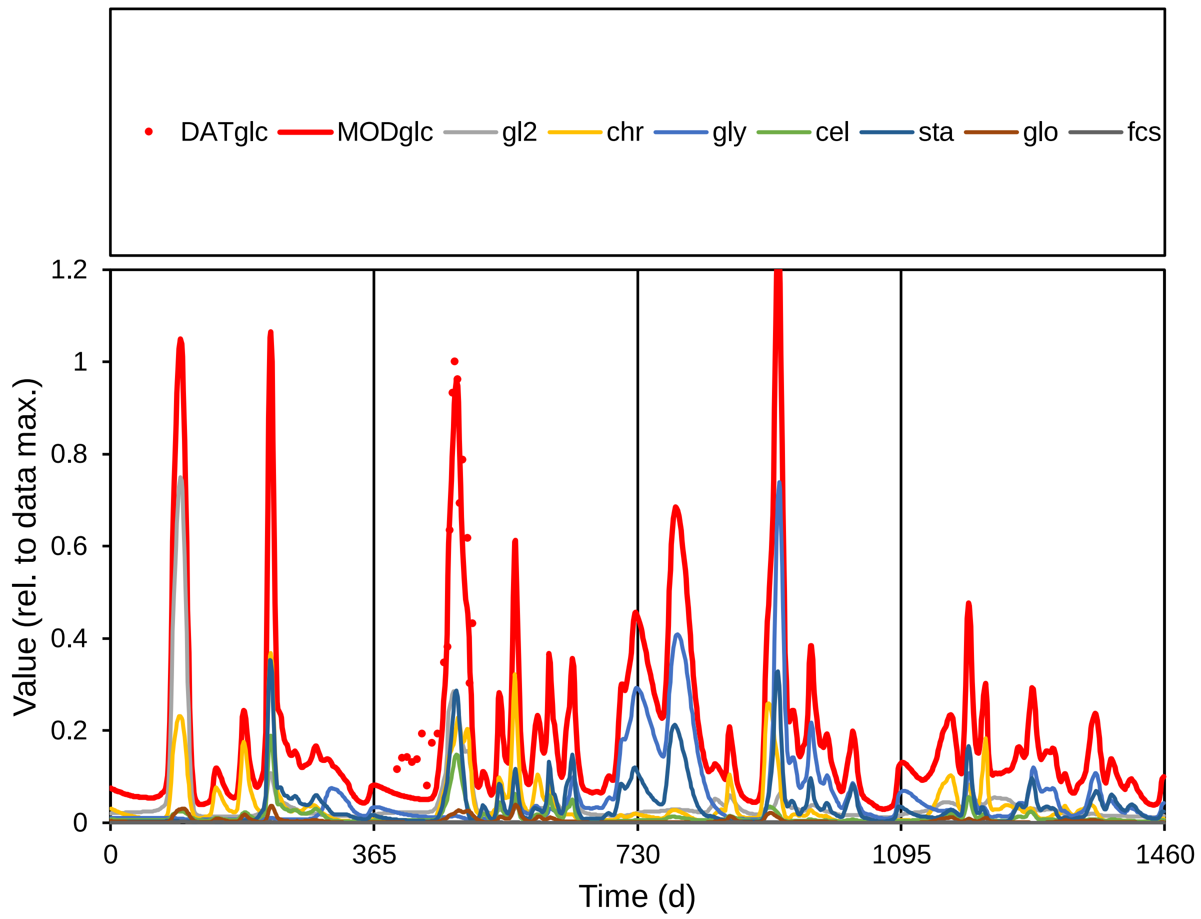

Figure S1(67). Model - data comparison for observation: glc
Normalized to max. value of data. Symbols are observations and lines are model. Red line corresponds to observations (e.g. Chlorophyll *a*), others are sub-components (e.g. individual phytoplankton species). See Tables S19 and S24 for observation and model component IDs, and mapping.

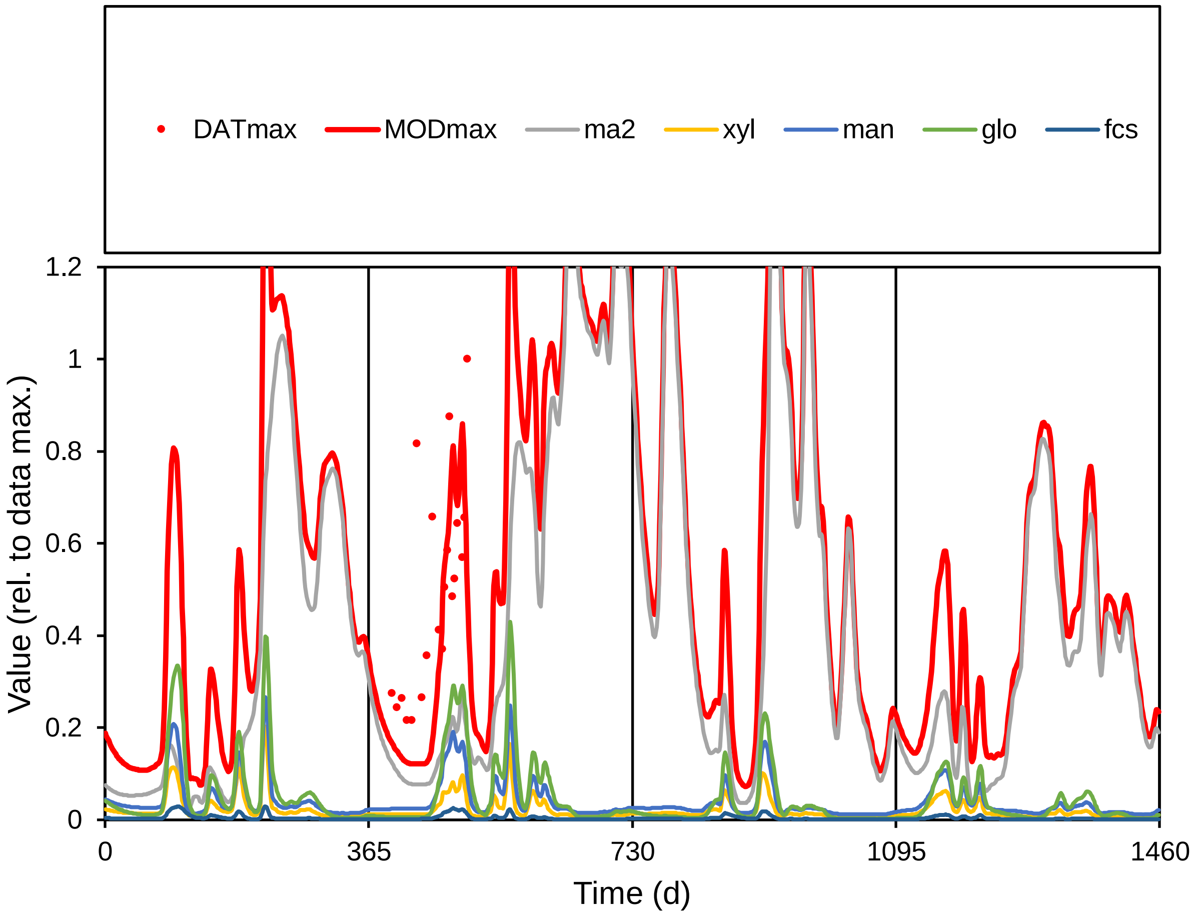

Figure S1(68). Model - data comparison for observation: max
Normalized to max. value of data. Symbols are observations and lines are model. Red line corresponds to observations (e.g. Chlorophyll *a*), others are sub-components (e.g. individual phytoplankton species). See Tables S19 and S24 for observation and model component IDs, and mapping.

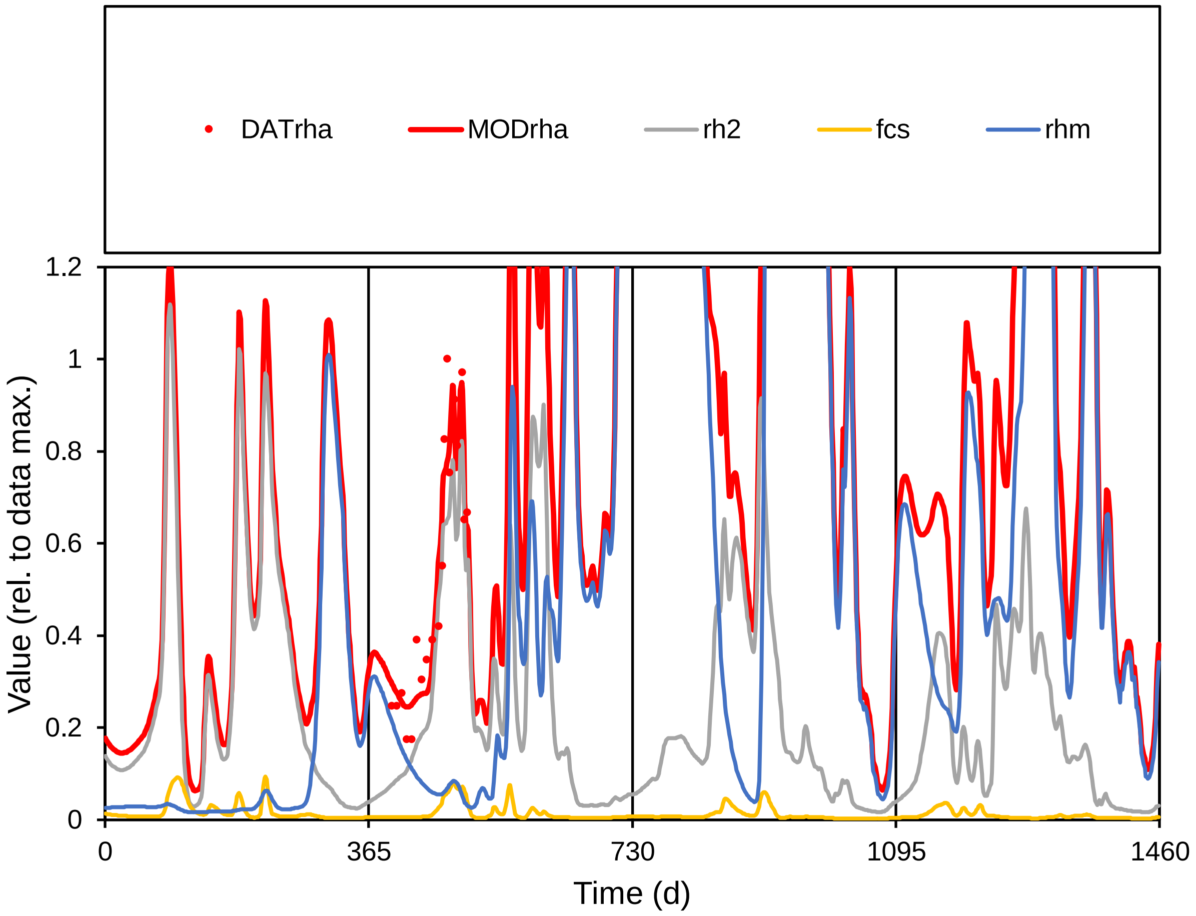

Figure S1(69). Model - data comparison for observation: rha
Normalized to max. value of data. Symbols are observations and lines are model. Red line corresponds to observations (e.g. Chlorophyll *a*), others are sub-components (e.g. individual phytoplankton species). See Tables S19 and S24 for observation and model component IDs, and mapping.

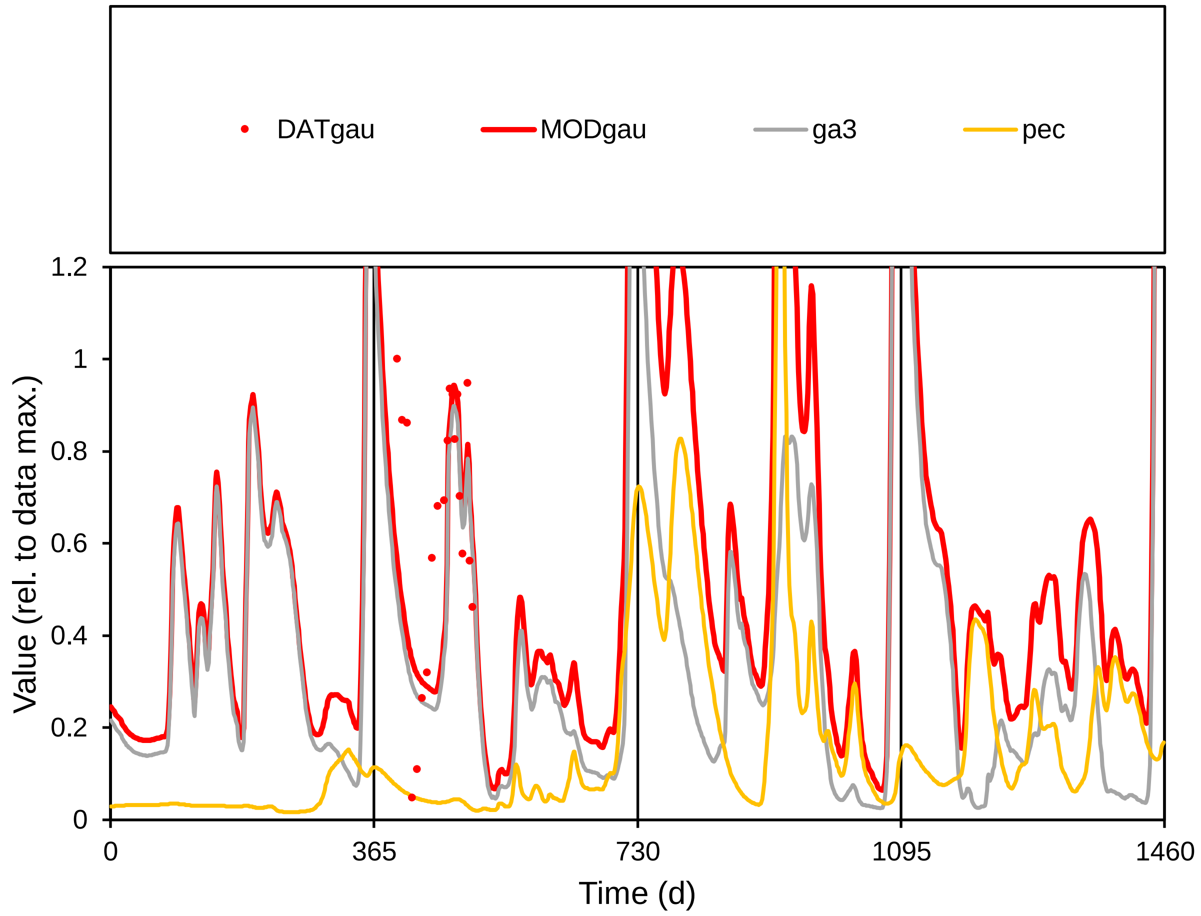

Figure S1(70). Model - data comparison for observation: gau
Normalized to max. value of data. Symbols are observations and lines are model. Red line corresponds to observations (e.g. Chlorophyll *a*), others are sub-components (e.g. individual phytoplankton species). See Tables S19 and S24 for observation and model component IDs, and mapping.

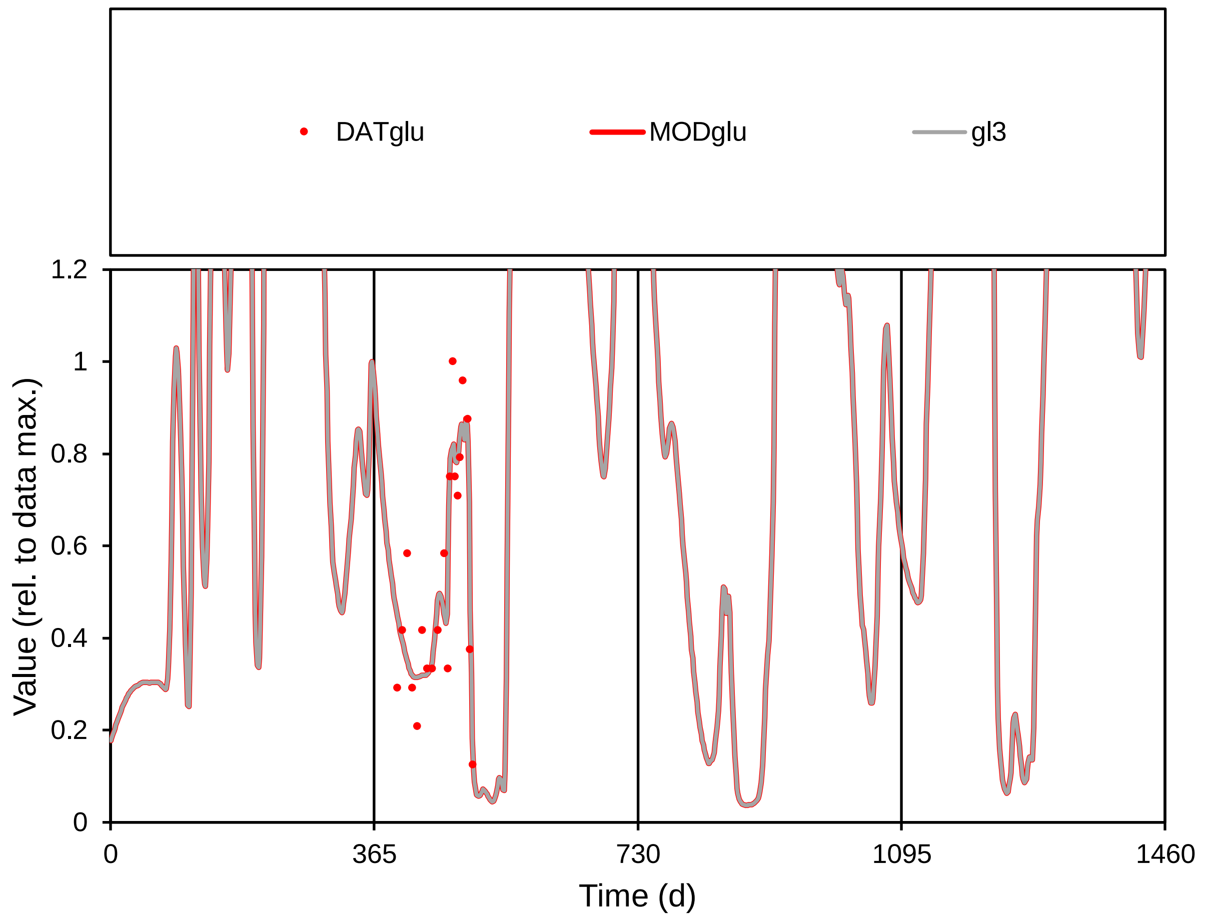

Figure S1(71). Model - data comparison for observation: glu
Normalized to max. value of data. Symbols are observations and lines are model. Red line corresponds to observations (e.g. Chlorophyll *a*), others are sub-components (e.g. individual phytoplankton species). See Tables S19 and S24 for observation and model component IDs, and mapping.

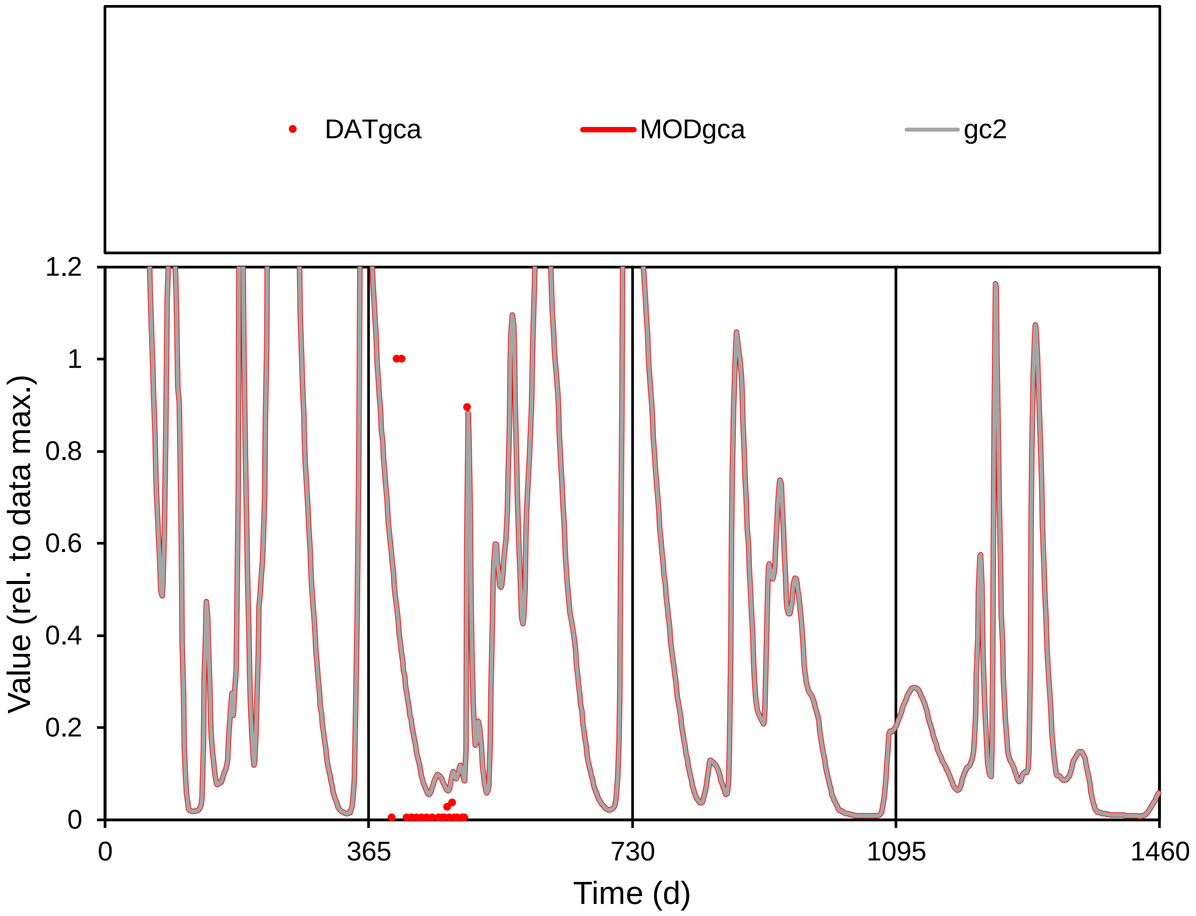

Figure S1(72). Model - data comparison for observation: gca
Normalized to max. value of data. Symbols are observations and lines are model. Red line corresponds to observations (e.g. Chlorophyll *a*), others are sub-components (e.g. individual phytoplankton species). See Tables S19 and S24 for observation and model component IDs, and mapping.

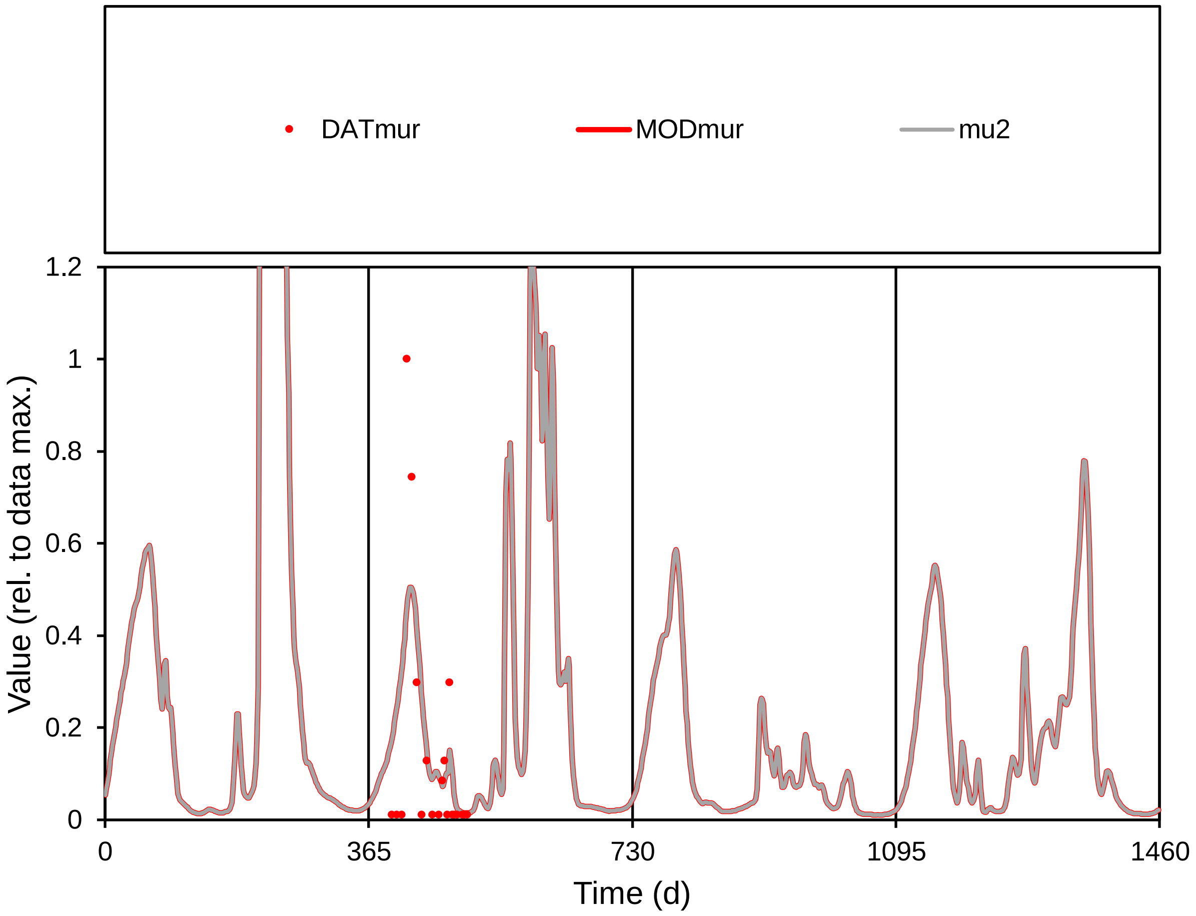

Figure S1(73). Model - data comparison for observation: mur
Normalized to max. value of data. Symbols are observations and lines are model. Red line corresponds to observations (e.g. Chlorophyll *a*), others are sub-components (e.g. individual phytoplankton species). See Tables S19 and S24 for observation and model component IDs, and mapping.

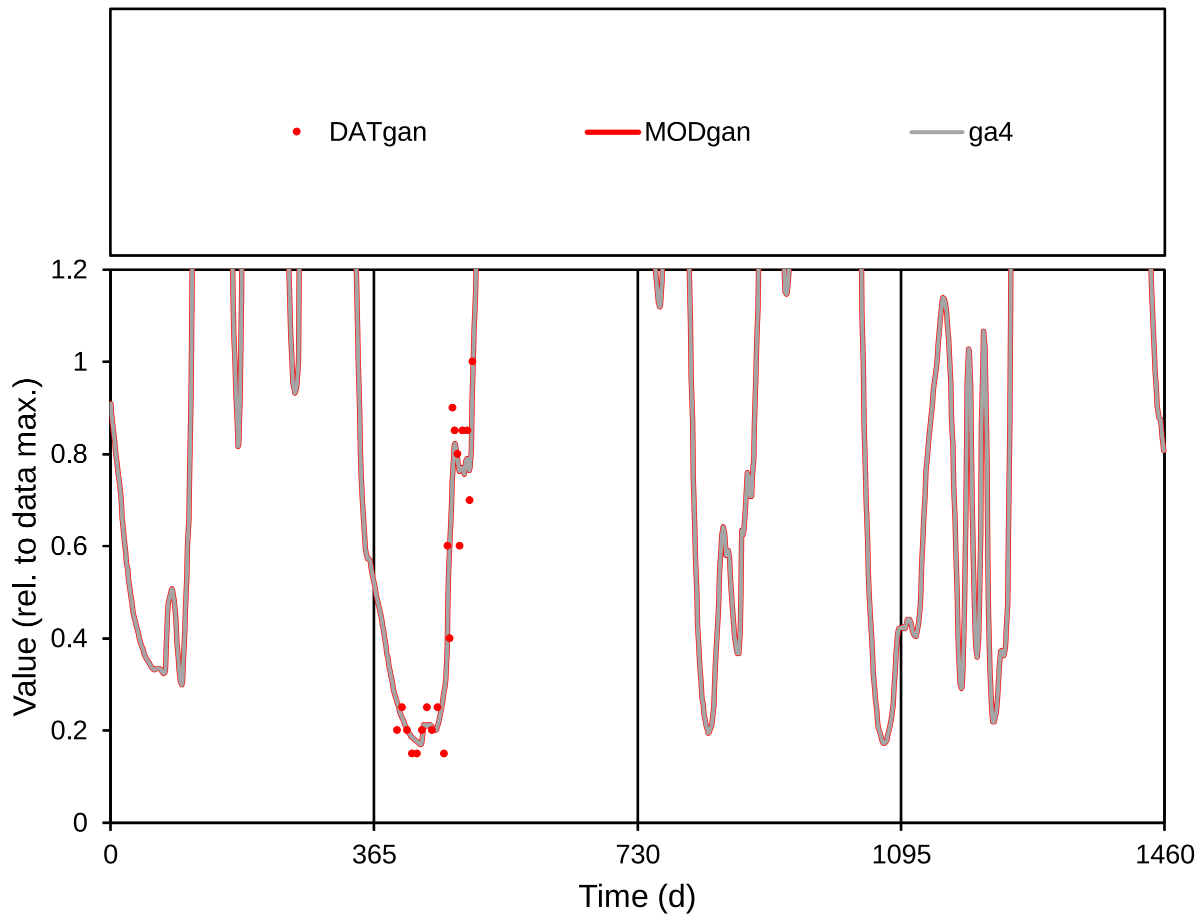

Figure S1(74). Model - data comparison for observation: gan
Normalized to max. value of data. Symbols are observations and lines are model. Red line corresponds to observations (e.g. Chlorophyll *a*), others are sub-components (e.g. individual phytoplankton species). See Tables S19 and S24 for observation and model component IDs, and mapping.

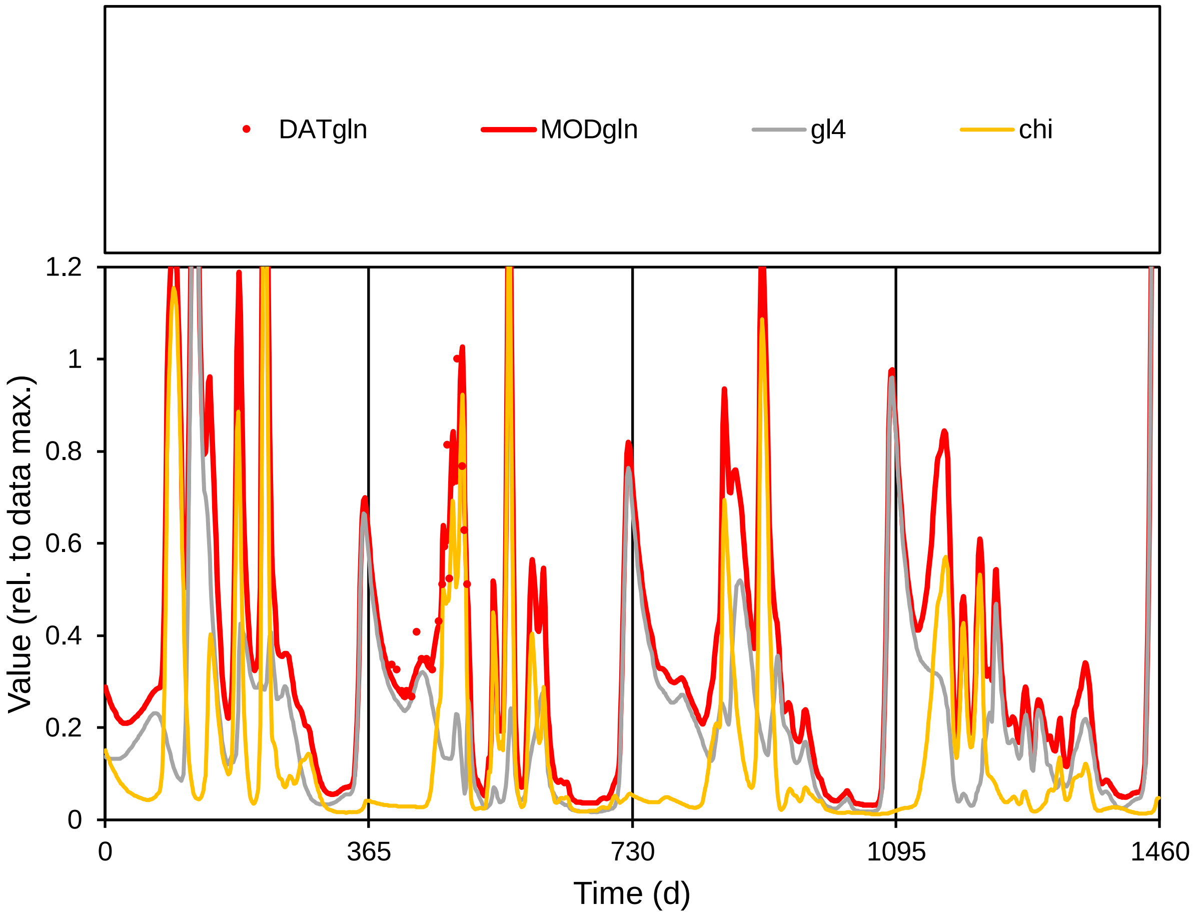

Figure S1(75). Model - data comparison for observation: gln
Normalized to max. value of data. Symbols are observations and lines are model. Red line corresponds to observations (e.g. Chlorophyll *a*), others are sub-components (e.g. individual phytoplankton species). See Tables S19 and S24 for observation and model component IDs, and mapping.
