# Supplementary material for: Dynamic carbon flux network of a diverse marine microbial community
Source: ISME Commun. 2021 Sep 25;1:50. doi: 10.1038/s43705-021-00055-7 (PMC9723560; doi:10.1038/s43705-021-00055-7)
Supplement: Supplementary file 3 — Supplementary Information [file 43705_2021_55_MOESM3_ESM.docx]

**Supplementary Information**

Dynamic carbon flux network of a diverse marine microbial community

Mayerhofer, et al.

correspondence to: [ferdi.hellweger@tu-berlin.de](mailto:ferdi.hellweger@tu-berlin.de)

**Included in this document:**

S1. Details of the mechanistic microbial ecosystem model

S2. Optimization routine

S3. LSA empirical network analysis

S4. References

S5. Figs. S2 to S8 and Tables S1 to S26

S6. Description of Dataset S1

**Other files:**

Fig. S1

Dataset S1

Movie S1

# Details of the mechanistic microbial ecosystem model

## Introduction

The purpose of this section is twofold: (1) document the modeling framework and (2) present details of the application to Helgoland presented in the main paper. The modeling framework includes additional features not used in the Helgoland application, like explicit simulation of zooplankton and viruses. Those features are documented here for completeness.

## Components and processes

The model consists of a number of components (concentrations, state variables) that interact via a number of processes (functions) (see Fig. S1 for components used in the Helgoland application). The model structure is flexible and puts no constraints on the number and types of processes that connect components, so the model can, for example, simulate mixotrophy. However, it helps to think in terms of and organize components into traditional ecological compartments. In that case, the processes corresponding to each component depend on the ecological compartment it is in. For example, algae/phytoplankton interact with DOM via exudation and death. Most ecological compartments (defined further in the next section) include a number of components (marked with “*” in Fig. S1).

Model components are generally selected based on observations. However, for a mechanistic model, it is important to close the mass balance and that puts some constraints on the definition of components. First, state variables cannot overlap. For example, the Helgoland data includes observations for “diatoms” as well as various diatom species (e.g. *Thalassiosira nordenskioeldii*, *Mediopyxis helysia*). A valid choice for state variables would be the individual diatom species, but excluding the “diatoms” observation, because it includes the individual species and thus overlaps. The “diatoms” observations can still be used in the optimization by making it a derived parameter. Second, the state variables have to cover the trophic levels. For example, *T. nordenskioeldii* and *M. helysia* would not be a good choice for a system that includes other phytoplankton, because they themselves cannot account for the total interaction with nutrients (e.g. uptake of nitrate). To cover the trophic level, “cryptic” species are added (conceptually similar to “others” in [Hellweger et al. (1)](#_ENREF_1)). The above examples are for phytoplankton, but the same applies to other classes, like heterotrophic bacteria, DOM, etc.

Further, we include micronutrients and inhibitors (described further below) as cryptic species, conceptually similar to “unknown compound” in [D'hoe et al. (2)](#_ENREF_2).

For processes, the model includes loading [w], flow [q], settling [s], first-order [f], respiration [r], photosynthesis [p], grazing [z], heterotrophy [h], viral [v], exudation [e], death [y], loss [u] and inhibition [i] (see Fig. S1 for processes used in the Helgoland application). Details of the processes are defined in Table S1 through Table S12).

## Dormancy

For microbes, at unfavorable conditions and corresponding low concentrations, dormancy may decrease loss by respiration as well as zooplankton and viral predation ([3](#_ENREF_3)). The effect of these factors is included here using a simplified approach. Specifically, a floor concentration (*Cflr*) is specified, and any loss process that would reduce the concentration below this value is blocked. A similar approach was used in previous models ([4](#_ENREF_4), [5](#_ENREF_5)).

Besides the fact that dormancy is a real biological process, it is included here for a more practical modeling reason, to control chaos. Specifically, the model includes many nonlinear processes that result in positive feedbacks (e.g. more phytoplankton > more DOM > more bacteria > more micronutrients > more phytoplankton …). Consequently, small changes in parameters may result in large differences in model results (i.e. chaos), which is problematic for the optimization routine. Chaos may be real and has important consequences for prediction ([Huisman and Weissing (6)](#_ENREF_6)), but here we are primarily interested in describing the ecosystem under present conditions, and less in predictions for different conditions. Including a dormancy concentration and forcing (e.g. grazing) microbes down to this level once a year helps to prevent chaos.

## Phytoplankton micronutrient limitation

Phytoplankton micronutrient limitation is added as an additional limitation term, along with macronutrient (DIN, PO4 and SIL) and other limitation. The formulation for micronutrient limitation and uptake generally follows that for macronutrients. That is, a Monod limitation term for each micronutrient is calculated and those are combined using a minimum formulation (Table S2). The micronutrient is taken up at a rate proportional to the photosynthesis rate and the micronutrient content (biomass fraction). To simplify the model, one parameter is used to describe the requirements for each micronutrient (*Fm*), which is used to scale a base half-saturation constant (*KspMbase*) and a base micronutrient content (*Smbase*, Table S1).

## Inhibitors

The model includes inhibitors, which can be produced by phytoplankton and bacteria via the exudation process. Inhibitors decay via a first-order process and kill phytoplankton or bacteria in a concentration-dependent manner.

## Environment and boundary conditions

The environment is a completely-mixed reactor with specified area (*A*) and depth (*H*). Boundary conditions include light intensity (*IT*), photoperiod (*f*), temperature (*T*) and nutrient loadings (*WNOX*, *WNH4*, *WPO4*, *WSIL*).

For the Helgoland application, external nutrient inputs (loadings) are unknown and therefore were calculated from observations *a priori* using a simplified approach. Simply put, we input sufficient NOX and PO4 to support the observed TN and TP concentrations. For nitrogen, it was assumed total nitrogen (TN) is made up of NOX, NH4 and phytoplankton nitrogen (PN), which was calculated from Chlorophyll *a* using conversions (see Table S3). It was further assumed the only loss processes are settling of PN and outflow of TN. These assumptions result in the following mass balance equation.

$$V \frac{dTN}{dt}=W_{NOX}-vs A PN-Q TN$$

This equation can be discretized and solved for the nitrogen input.

$$W_{NOX}=V\frac{\Delta TN}{\Delta t}+vs A \bar{PN}+Q \bar{TN}$$

When this calculation is applied to a data set with high variability, it can lead to negative values, which does not agree with the concept of external input. Therefore, the load is kept positive, but a deficit is tracked so that the resulting cumulative input is consistent with the mass balance. Input of PO4 and SIL are handled equivalently. For the Helgoland application, there is no input of NH4.

# Optimization routine

## Observations

The optimization routine minimizes an objective function (*OFXNG*) that quantifies the discrepancy between model and data. It can consider any number and type of observations, including concentration of individual types (e.g. *Polaribacter*, nitrate) and summary parameters (e.g. diatoms, Chlorophyll *a*), and other parameters (e.g. Secchi Disk depth).

Some curation of the observations may be required. For example, it is important to distinguish between “no observation” and an observation “below the detection limit”. For the Helgoland application, for nutrients, phytoplankton and bacteria, values below the detection limit (reported as 0) were replaced with half the minimum reported value.

## Objective function

The discrepancy between an individual data point and the model is quantified as the minimum error square (*ES*) in two-dimensional space, as shown schematically in Fig. S2, using:

$${ES}_{i,j}=min\left[ \left( C_{d,i}-C_{m,k} \right)^{2}+\left( \left[ t_{d,i}-t_{m,k} \right] ave\left[ C_{d,i},C_{m,k} \right] k_{ch} \right)^{2} \right]$$

Where *i* is the index for data series, *j* is the index for data point, *k* is the index for the model point, *C_d_* is the data value, *C_m_* is the model value, *t_d_* is the data time, *t_m_* is the model time and *k_ch_* is a first-order rate constant relating the value and time dimensions.

The individual error values (*ES*) are combined as a normalized root mean squared error (*NRMSE*):

$${NRMSE}_{i}=\frac{\sqrt{\frac{\sum_{j} {ES}_{i,j}}{n_{i}}}}{a_{i}}$$

Where *n* is the number of data points, *a* is the average of the data.

Individual data series are weighted using user-specified weight (*w*), and then summed. The total error (*TOTERR*), as shown in Fig. 1 of the main paper is:

$$TOTERR=\sum_{i} {NRMSE}_{i} w_{i}$$

This parameter corresponds to the objective function, but at lower de-lump levels, the individual data series are also weighted using the number of components contributing to the parameter (*nc*, see below), and then summed into one objective function (*OFXNG*).

$$OFXNG=\sum_{i} {NRMSE}_{i} \frac{{nc}_{i,l}}{{nc}_{i,max}} w_{i}$$

where *l* is the de-lump step. This parameter is the objective function used by the optimization routine. At the final de-lump level *TOTERR* = *OFXNG*.

The adjustment based on the de-lump level is done so that summary parameters are discounted at early de-lump levels and do not outweigh individual parameters. Consider, for example, a model with a summary parameter *chl* with weight (*w*) 10 and several individual parameters including *dif* with weight 1. Without the adjustment, when only *dif* is turned on at de-lump level 1, then any parameter changes affecting *dif* would affect mostly the *chl* error and the *dif* error would be effectively discounted. Only at later de-lump levels, when more components contribute to *chl*, would the *dif* error become relevant.

In the Helgoland application, the first-order rate constant relating the value and time dimensions (*k_ch_*) was assigned to 0.3/day, which was found to produce the best results. The weight is used to attach higher importance to some observations. For example, in the Helgoland application a higher weight is given to total bacteria quantified with DAPI than each of the ~40 individual bacteria types identified with CARD-FISH probes. The weights for the Helgoland application for each observation shown in Table S19 - Table S21.

## Constraints

Model parameters are listed along with literature ranges and notes (where applicable) in Table S3 through Table S18. Parameter ranges are generally based on literature. In some cases, the model range is assigned wider or narrower than the literature range, for reasons described below.

- Many parameter ranges are from highly lumped observations or models, like models that simulate one phytoplankton species or field observations of Chlorophyll *a*. It is reasonable to assume that the parameters for a diverse community have a larger range.
- The model is structured to allow interactions between all components and a specific interaction is “turned off” via the corresponding parameter value rather than a binary switch. This is done to avoid discontinuities, which are problematic for the optimization routine. In that case the parameter value may not be comparable to observations. For example, a very high heterotrophy saturation parameter (*Ksh*) is used to turn a substrate off, and the value may be much larger than any observation from a laboratory experiment.
- In some cases parameter ranges may be constrained further to avoid unreasonable parameter combinations and model behavior. This applies, for example, to the max. heterotrophy rate (*kh*) and half-saturation constant (*Ksh*), where literature ranges are very large. The optimization routine, which adjusts parameters freely within the specified ranges, may end up with a high *kh* and low *Ksh*, resulting in rapid DOM consumption and very low time step to maintain stability. Consider, for example, a reasonable bacteria concentrations of 5 μmolC/L, a very high heterotrophy rate of 290 1/d, and very low half-saturation constant of 0.043 μmolC/L, and the case where the substrate concentration is equal to the half -saturation constant. In this case the substrate would be used up in about 5 seconds, requiring a time step substantially below that to maintain stability.

Parameters can be constrained on a general or specific basis. A general constrain may be put on the PO4 half-saturation coefficient for photosynthesis, which would apply to all phytoplankton. A component specific constraint may be to prevent some bacteria from using the glycolate based on absence of the *glcD* gene ([7](#_ENREF_7)).

In addition to the min. and max. constraints on individual parameters, constraints may be imposed on derived parameters. This was also done by [Weitz et al. (8)](#_ENREF_8). For example, several parameter combinations may lead to collapse of the ecosystem. This is expected due to the positive feedbacks in the model (e.g. less phytoplankton > less DOM > less bacteria > less micronutrients > less phytoplankton). Therefore, a constrained is added to keep the average concentration above the dormancy concentration (which would indicate extinction).

## Optimization method

The optimization problem is characterized by a large number of dependent parameters and local minima in the objective function. Consequently, the optimization method includes a combination of single- and multi-parameter optimization (SPO, MPO) routines and Monte Carlo/Latin Hypercube Sampling (MTC) steps. The method is illustrated in Fig. S3 and described further below.

For each de-lump level (DELU), a number of iterations (ITER) are performed until a convergence criteria is reached. Within each iteration a number of single-parameter optimization steps are performed on the complete parameter set (COMP). Then, several focused optimizations are performed on a subset of parameters, including:

- A number of single-parameter optimization steps on those parameters identified as most sensitive in the prior complete optimization (BEST).
- Multi-parameter optimization on globally-dependent parameters, like the Chlorophll *a* content of phytoplankton (DEPG).
- Multi-parameter optimization on locally-dependent parameters, like the maximum rate and half-saturation parameters of Monod functions (DEPL).
- Multi-parameter optimization on local de-lumped parameters, meaning all the parameters of an old and new species (LDEL).
- Multi-parameter optimization on pointer de-lumped parameters, like half-saturation constants corresponding to an old and new species (PDEL).
- A multi-parameter optimization on cross-feeding parameters, like all the parameters controlling the production and consumption of a specific substrate (XFED).
- A Monte Carlo scan across the entire parameter range for selected parameters (GSCA).
- A Monte Carlo scan across a limited parameter range around the current value for selected parameters (LSCA).
- A single-parameter optimization on half-saturation parameters of Monod functions, with a compensation in other parameters to keep the limitation term constant (MONO).

Then, the entire array of optimization functions within one de-lump level is repeated several times (REDO).

Finally, multiple simulations are run in parallel. The number of replicate runs can be based on their error distribution (See Fig. S4).

## De-lumping

See the description in the Methods section of the main paper. Parameters corresponding to absolute concentrations (e.g. *Cic*, *Cin*, *Cflr*) are reduced according to a specified de-lump fraction. For example, if *dix* with *Cflr* = 9.6e-4 (μmolC/L) de-lumps with 25% to *mhe*, the *Cflr* of *dix* and *mhe* becomes 0.75 9.6e-4 = 7.2e-4 and 0.25 9.6e-4 = 2.4e-4, respectively.

# LSA empirical network analysis

The same observations used for the FluxNet inference are analyzed using local similarity analysis (LSA) ([Ruan et al. (9)](#_ENREF_9)). Consistent with the focus of this study on phytoplankton – bacteria interaction via DOM substrate, only phytoplankton and bacteria are included. Also, interactions are limited to phytoplankton – bacteria (i.e. no phytoplankton – phytoplankton) and only positive correlations with bacteria lagging phytoplankton are considered. The analysis focuses on spring blooms and the dataset is therefore split into four groups, each including from 30 days before to 60 days after the bloom, defined as max. observed Chl. *a* concentration (23.03.2009, 22.04.2010, 26.04.2011, 23.04.2012). Gaps in the data were filled using linear interpolation ([9](#_ENREF_9)).

# References

1. Hellweger FL, Kravchuk ES, Novotny V, & Gladyshev MI (2008) Agent-Based Modeling of the Complex Life Cycle of a Cyanobacterium (Anabaena) in a Shallow Reservoir. *Limnology and Oceanography* 53(4):1227-1241.

2. D'hoe K*, et al.* (2018) Integrated culturing, modeling and transcriptomics uncovers complex interactions and emergent behavior in a synthetic gut community. *bioRxiv*:299644.

3. Lennon JT & Jones SE (2011) Microbial seed banks: the ecological and evolutionary implications of dormancy. *Nat Rev Micro* 9(2):119-130.

4. Daines SJ, Clark JR, & Lenton TM (2014) Multiple environmental controls on phytoplankton growth strategies determine adaptive responses of the N : P ratio. *Ecology Letters* 17(4):414-425.

5. Salihoglu B, Garçon V, Oschlies A, & Lomas MW (2008) Influence of nutrient utilization and remineralization stoichiometry on phytoplankton species and carbon export: A modeling study at BATS. *Deep Sea Research Part I: Oceanographic Research Papers* 55(1):73-107.

6. Huisman J & Weissing FJ (2001) Fundamental Unpredictability in Multispecies Competition. *The American Naturalist* 157(5):488-494.

7. Paver SF & Kent AD (2010) Temporal Patterns in Glycolate-Utilizing Bacterial Community Composition Correlate with Phytoplankton Population Dynamics in Humic Lakes. *Microb Ecol* 60(2):406-418.

8. Weitz JS*, et al.* (2015) A multitrophic model to quantify the effects of marine viruses on microbial food webs and ecosystem processes. *The Isme Journal* 9:1352.

9. Ruan Q*, et al.* (2006) Local similarity analysis reveals unique associations among marine bacterioplankton species and environmental factors. *Bioinformatics* 22(20):2532-2538.

10. Hellweger FL & Lall U (2004) Modeling the Effect of Algal Dynamics on Arsenic Speciation in Lake Biwa. *Environmental Science & Technology* 38(24):6716-6723.

11. Chapra SC (1997) *Surface Water-Quality Modeling* (McGraw-Hill, Boston).

12. Jakobsen HH & Markager S (2016) Carbon-to-chlorophyll ratio for phytoplankton in temperate coastal waters: Seasonal patterns and relationship to nutrients. *Limnology and Oceanography* 61(5):1853-1868.

13. Bertilsson S, Berglund O, Karl DM, & Chisholm SW (2003) Elemental composition of marine Prochlorococcus and Synechococcus: Implications for the ecological stoichiometry of the sea. *Limnology and Oceanography* 48(5):1721-1731.

14. Teeling H*, et al.* (2016) Recurring patterns in bacterioplankton dynamics during coastal spring algae blooms. *eLife* 5:e11888.

15. Thomann RV & Mueller JA (1987) *Principles of surface water quality modeling and control* (Harper & Row, Publishers).

16. Sperling M*, et al.* (2017) Combined Carbohydrates Support Rich Communities of Particle-Associated Marine Bacterioplankton. *Frontiers in Microbiology* 8(65).

17. White AE, Giovannoni SJ, Zhao Y, Vergin K, & Carlson CA (2019) Elemental content and stoichiometry of SAR11 chemoheterotrophic marine bacteria. *Limnology and Oceanography Letters* 4(2):44-51.

18. Hellweger FL, Huang Y, & Luo H (2018) Carbon limitation drives GC content evolution of a marine bacterium in an individual-based genome-scale model. *The ISME Journal*.

19. Painter TJ (1983) Algal Polysaccharides.

20. Franklin DJ & Berges JA (2004) Mortality in cultures of the dinoflagellate Amphidinium carterae during culture senescence and darkness. *Proceedings. Biological sciences / The Royal Society* 271:2099-2107.

21. Suzuki E & Suzuki R (2013) Variation of storage polysaccharides in phototrophic microorganisms. *Journal of Applied Glycoscience* 60:21-27.

22. Hildebrand M, Manandhar-Shrestha K, & Abbriano R (2017) Effects of chrysolaminarin synthase knockdown in the diatom Thalassiosira pseudonana: Implications of reduced carbohydrate storage relative to green algae. *Algal Research* 23:66-77.

23. Becker S, Scheffel A, Polz MF, & Hehemann J-h (2017) Accurate quantification of laminarin in marine organic matter with enzymes from marine microbes. *Applied and environmental microbiology* 83:1-14.

24. Poulicek M & Jeuniaux C (1991) Chitin biodegradation in marine environments: An experimental approach. *Biochemical Systematics and Ecology* 19:385-394.

25. Anonymous (<Jeuniaux, Voss-Foucart - 1991 - Chitin biomass and production in the marine environment.pdf>.

26. Sturm K-D & Hesse K-J (2000) Chitin and Chitosan. Natural Polymers from the Sea. *Ocean Challenge* 10:20-24.

27. Cauchie HM (2002) Chitin production by arthropods in the hydrosphere. *Hydrobiologia* 470:63-95.

28. Chiovitti A, Ngoh JE, & Wetherbee R (2006) 1,3-β-D-glucans from Haramonas dimorpha (Raphidophyceae). *Botanica Marina* 49:360-362.

29. Alderkamp AC, Buma AGJ, & Van Rijssel M (2007) The carbohydrates of Phaeocystis and their degradation in the microbial food web. *Biogeochemistry* 83:99-118.

30. Alderkamp A-c, Rijssel MV, & Bolhuis H (2007) Characterization of marine bacteria and the activity of their enzyme systems involved in degradation of the algal storage glucan laminarin. 59:108-117.

31. Cole JK*, et al.* (2014) Phototrophic biofilm assembly in microbial-mat-derived unicyanobacterial consortia: model systems for the study of autotroph-heterotroph interactions. *Frontiers in microbiology* 5:109.

32. Msanne J*, et al.* (2012) Metabolic and gene expression changes triggered by nitrogen deprivation in the photoautotrophically grown microalgae Chlamydomonas reinhardtii and Coccomyxa sp. C-169. *Phytochemistry* 75:50-59.

33. Gardner RD, Lohman E, Gerlach R, Cooksey KE, & Peyton BM (2013) Comparison of CO2 and bicarbonate as inorganic carbon sources for triacylglycerol and starch accumulation in Chlamydomonas reinhardtii. *Biotechnology and Bioengineering* 110:87-96.

34. Jeuniaux C & Voss-Foucart MF (1991) Chitin biomass and production in the marine environment. *Biochemical Systematics and Ecology* 19:347-356.

35. Michel G, Tonon T, Scornet D, Cock JM, & Kloareg B (2010) Central and storage carbon metabolism of the brown alga Ectocarpus siliculosus: Insights into the origin and evolution of storage carbohydrates in Eukaryotes. *New Phytologist* 188:67-81.

36. Anonymous (2012) Plant carbohydrates I: Intracellular carbohydrates.

37. Myklestad SM & Granum E (2009) Chemistry, Biochemistry, and Biology of 1-3 Beta Glucans and Related Polysaccharides.663-677.

38. Sumbali G & Mehrotra R (2009) Principles of microbiology.

39. Willis A, Chiovitti A, Dugdale TM, & Wetherbee R (2013) Characterization of the extracellular matrix of Phaeodactylum tricornutum (Bacillariophyceae): Structure, composition, and adhesive characteristics. *Journal of Phycology* 49:937-949.

40. Bernaerts TMM*, et al.* (2018) Comparison of microalgal biomasses as functional food ingredients: Focus on the composition of cell wall related polysaccharides. *Algal Research* 32:150-161.

41. Janse I, Zwart G, van der Maarel M, & Gottschal J (2000) Composition of the bacterial community degrading Phaeocystis mucopolysaccharides in enrichment cultures. *Aquatic Microbial Ecology* 22:119-133.

42. Bold HC & Wynne MJ (1985) Introduction to the algae: structure and reproduction.

43. Calvo P, Fernandez-Aliseda MC, Garrido J, & Torres A (2003) Ultrastructure, encystment and cyst wall composition of the resting cyst of the peritrich ciliate Opisthonecta henneguyi. *Journal of Eukaryotic Microbiology* 50:49-56.

44. Schnoor JL (1996) *Environmental modeling: fate and transport of pollutants in water, air, and soil* (John Wiley and Sons).

45. Connolly JP, Coffin RB, & Landeck RE (1992) Modeling carbon utilization by bacteria in natural water systems. *Modeling the Metabolic and Physiologic Activities of Micro-organisms*, ed Hurst CJ (Wiley, New York), pp 249-276.

46. Gómez-Consarnau L*, et al.* (2019) Microbial rhodopsins are major contributors to the solar energy captured in the sea. *Science Advances* 5(8):eaaw8855.

47. Di Toro DM (1980) Applicability of cellular equilibrium and monod theory to phytoplankton growth kinetics. *Ecological Modelling* 8:201-218.

48. Morel FMM (1987) KINETICS OF NUTRIENT UPTAKE AND GROWTH IN PHYTOPLANKTON1. *Journal of Phycology* 23(1):137-150.

49. Sommer U (1998) *Biologische meereskunde* (Springer).

50. Maestrini SY, Robert J-M, Leftley JW, & Collos Y (1986) Ammonium thresholds for simultaneous uptake of ammonium and nitrate by oyster-pond algae. *Journal of Experimental Marine Biology and Ecology* 102(1):75-98.

51. Mentges A, Feenders C, Deutsch C, Blasius B, & Dittmar T (2019) Long-term stability of marine dissolved organic carbon emerges from a neutral network of compounds and microbes. *Scientific Reports* 9(1):17780.

52. Bucci V, Hoover S, & Hellweger F (2011) Modeling Adaptive Mutation of Enteric Bacteria in Surface Water Using Agent-Based Methods. *Water Air Soil Pollut* 223(5):2035–2049.

53. Kirchman DL (2016) Growth Rates of Microbes in the Oceans. *Annual Review of Marine Science* 8(1):285-309.

54. Long AM, Hou S, Ignacio-Espinoza JC, & Fuhrman JA (2019) Benchmarking metagenomic marine microbial growth prediction from codon usage bias and peak-to-trough ratios. *bioRxiv*:786939.

55. Vieira-Silva S & Rocha EPC (2010) The Systemic Imprint of Growth and Its Uses in Ecological (Meta)Genomics. *PLOS Genetics* 6(1):e1000808.

56. Muscarella ME, Howey XM, & Lennon JT (Trait-based approach to bacterial growth efficiency. *Environmental Microbiology* n/a(n/a).

57. Giorgio PAd & Cole JJ (1998) BACTERIAL GROWTH EFFICIENCY IN NATURAL AQUATIC SYSTEMS. *Annual Review of Ecology and Systematics* 29(1):503-541.

58. Ihssen J & Egli T (2005) Global physiological analysis of carbon‐and energy‐limited growing Escherichia coli confirms a high degree of catabolic flexibility and preparedness for mixed substrate utilization. *Environmental microbiology* 7(10):1568-1581.

59. Egli T (2010) How to live at very low substrate concentration. *Water Research* 44(17):4826-4837.

60. Lombard V, Golaconda Ramulu H, Drula E, Coutinho PM, & Henrissat B (2013) The carbohydrate-active enzymes database (CAZy) in 2013. *Nucleic Acids Research* 42:D490-D495.

61. Klindworth A*, et al.* (2014) Marine Genomics Diversity and activity of marine bacterioplankton during a diatom bloom in the North Sea assessed by total RNA and pyrotag sequencing. *Marine Genomics* 18:185-192.

62. Hahnke RL*, et al.* (2015) Dilution cultivation of marine heterotrophic bacteria abundant after a spring phytoplankton bloom in the North Sea. *Environmental Microbiology* 17:3515-3526.

63. Avcı B*, et al.* (2017) Genomic and physiological analyses of ‘Reinekea forsetii’ reveal a versatile opportunistic lifestyle during spring algae blooms. *Environmental Microbiology* 19:1209-1221.

64. Neumann AM*, et al.* (2015) Different utilization of alginate and other algal polysaccharides by marine Alteromonas macleodii ecotypes. *Environmental Microbiology* 17:3857-3868.

65. Koch H*, et al.* (2019) Biphasic cellular adaptations and ecological implications of Alteromonas macleodii degrading a mixture of algal polysaccharides. *ISME Journal* 13:92-103.

66. Dupont CL*, et al.* (2012) Genomic insights to SAR86, an abundant and uncultivated marine bacterial lineage. *ISME Journal* 6:1186-1199.

67. Hoarfrost A*, et al.* (2020) Global ecotypes in the ubiquitous marine clade SAR86. *ISME Journal* 14:178-188.

68. Tang YZ, Koch F, & Gobler CJ (2010) Most harmful algal bloom species are vitamin B 1 and B 12 auxotrophs. 107:20756-20761.

69. Klippel B*, et al.* (2011) Complete genome sequence of the marine cellulose- and xylan-degrading bacterium Glaciecola sp. strain 4H-3-7+YE-5. *Journal of Bacteriology* 193:4547-4548.

70. Yin J*, et al.* (2013) Complete genome sequence of Glaciecola psychrophila strain 170T. *Genome Announcements* 1:5-6.

71. Xing P*, et al.* (2015) Niches of two polysaccharide-degrading Polaribacter isolates from the North Sea during a spring diatom bloom. *ISME Journal* 9:1410-1422.

72. Kappelmann L*, et al.* (2019) Polysaccharide utilization loci of North Sea Flavobacteriia as basis for using SusC/D-protein expression for predicting major phytoplankton glycans. *ISME Journal* 13:76-91.

73. Avcı B, Krüger K, Fuchs BM, Teeling H, & Amann RI (2020) Polysaccharide niche partitioning of distinct Polaribacter clades during North Sea spring algal blooms. *ISME Journal*.

74. Krüger K*, et al.* (2019) In marine Bacteroidetes the bulk of glycan degradation during algae blooms is mediated by few clades using a restricted set of genes. *ISME Journal* 13:2800-2816.

75. Glöckner FO*, et al.* (2003) Complete genome sequence of the marine planctomycete Pirellula sp. strain 1. *Proceedings of the National Academy of Sciences of the United States of America* 100:8298-8303.

76. Wecker P*, et al.* (2009) Transcriptional response of the model planctomycete Rhodopirellula baltica SH1Tto changing environmental conditions. *BMC Genomics* 10:410.

77. Orellana LH*, et al.* (2019) Niche differentiation among annually recurrent coastal Marine Group II Euryarchaeota. *ISME Journal* 13:3024-3036.

78. Gifford SM, Sharma S, Booth M, & Moran MA (2013) Expression patterns reveal niche diversification in a marine microbial assemblage. *ISME Journal* 7:281-298.

79. Yilmaz P, Yarza P, Rapp JZ, & Glöckner FO (2016) Expanding the world of marine bacterial and archaeal clades. *Frontiers in Microbiology* 6:1-29.

80. Sheik AR*, et al.* (2014) Responses of the coastal bacterial community to viral infection of the algae Phaeocystis globosa. *The ISME journal* 8:212-225.

81. Cao H*, et al.* (2016) Delta-proteobacterial SAR324 group in hydrothermal plumes on the South Mid-Atlantic Ridge. *Scientific Reports* 6:1-9.

82. Haroon MF, Thompson LR, & Stingl U (2016) Draft genome sequence of uncultured SAR324 bacterium lautmerah10, binned from a Red Sea metagenome. *Genome Announcements* 4:1-2.

83. Manivasagan P, Venkatesan J, & Kim SK (2013) Introduction to Marine Actinobacteria. *Marine Microbiology: Bioactive Compounds and Biotechnological Applications*:1-19.

84. Baines SB & Pace ML (1991) The production of dissolved organic matter by phytoplankton and its importance to bacteria: Patterns across marine and freshwater systems. *Limnology and Oceanography* 36(6):1078-1090.

85. Myklestad SM (1995) Release of extracellular products by phytoplankton with special emphasis on polysaccharides. *The Science of the total environment* 165:155-164.

86. Aluwihare LI & Repeta DJ (1999) A comparison of the chemical characteristics of oceanic DOM and extracellular DOM produced by marine algae. *Marine Ecology Progress Series* 186:105-117.

87. Shniukova EI & Zolotareva EK (2015) Diatom exopolysaccharides: A review. *International Journal on Algae* 17:50-67.

88. Paulsen BS & Vieira AAH (1994) STRUCTURE OF THE CAPSULAR AND EXTRACELLULAR POLYSACCHARIDES PRODUCED BY THE DESMID SPONDYLOSIUM PANDURIFORME (CHLOROPHYTA)1. *Journal of Phycology* 30:638-641.

89. Lombardi AT & Vieira AAH (1999) Lead- and copper-complexing extracellular ligands released by Kirchneriella aperta (Chloroccocales, Chlorophyta). *Phycologia* 38:283-288.

90. MacKenzie L, Sims I, Beuzenberg V, & Gillespie P (2002) Mass accumulation of mucilage caused by dinoflagellate polysaccharide exudates in Tasman Bay, New Zealand. *Harmful Algae* 1:69-83.

91. Pistocchi R*, et al.* (2005) Relevance of the dinoflagellate Gonyaulax fragilis in mucilage formations of the Adriatic Sea. *Science of the Total Environment* 353:307-316.

92. Walker CE*, et al.* (2018) An extracellular polysaccharide-rich organic layer contributes to organization of the coccosphere in coccolithophores. *Frontiers in Marine Science* 5:1-12.

93. Hellweger FL, Jabbur ML, Johnson CH, van Sebille E, & Sasaki H (2019) Circadian clock helps cyanobacteria manage energy in coastal and high latitude ocean. *The ISME Journal*.

94. Bucci V, Nunez-Milland D, Twining B, & Hellweger F (2012) Microscale patchiness leads to large and important intraspecific internal nutrient heterogeneity in phytoplankton. *Aquat Ecol* 46(1):101-118.

95. Buser H-R, Müller MD, & Theobald N (1998) Occurrence of the Pharmaceutical Drug Clofibric Acid and the Herbicide Mecoprop in Various Swiss Lakes and in the North Sea. *Environmental Science & Technology* 32(1):188-192.

96. Loebl M, van Beusekom JEE, & Philippart CJM (2013) No microzooplankton grazing during a Mediopyxis helysia dominated diatom bloom. *Journal of Sea Research* 82:80-85.

97. Menden-Deuer S & Lessard EJ (2000) Carbon to volume relationships for dinoflagellates, diatoms, and other protist plankton. *Limnology and Oceanography* 45(3):569-579.

98. Olenina I (2006) Biovolumes and size-classes of phytoplankton in the Baltic Sea.

99. Harrison PJ*, et al.* (2015) Cell volumes of marine phytoplankton from globally distributed coastal data sets. *Estuarine, Coastal and Shelf Science* 162:130-142.

100. Takahashi K (1989) Silicoflagellates as productivity indicators: evidence from long temporal and spatial flux variability responding to hydrography in the Northeast Pacifik. *Global Biogeochemical Cycles* 3:43-61.

101. Montagnes DJS, Berges JA, Harrison PJ, & Taylor FJR (1994) Estimating carbon, nitrogen, protein, and chlorophyll a from volume in marine phytoplankton. *Limnology and Oceanography* 39:1044-1060.

102. Cottrell MT & Kirchman DL (2016) Transcriptional Control in Marine Copiotrophic and Oligotrophic Bacteria with Streamlined Genomes. *Applied and Environmental Microbiology* 82(19):6010-6018.

103. Zhao X*, et al.* (2017) Three-Dimensional Structure of the Ultraoligotrophic Marine Bacterium "Candidatus Pelagibacter ubique". *Appl Environ Microbiol* 83(3).

104. Wagner-Dobler I, Rheims H, Felske A, Pukall R, & Tindall BJ (2003) Jannaschia helgolandensis gen. nov., sp. nov., a novel abundant member of the marine Roseobacter clade from the North Sea. *Int J Syst Evol Microbiol* 53(Pt 3):731-738.

105. Ho A, Di Lonardo DP, & Bodelier PL (2017) Revisiting life strategy concepts in environmental microbial ecology. *FEMS Microbiol Ecol* 93(3).

106. Yao F*, et al.* (2017) Microbial Taxa Distribution Is Associated with Ecological Trophic Cascades along an Elevation Gradient. *Front Microbiol* 8:2071.

107. Pelve EA, Fontanez KM, & DeLong EF (2017) Bacterial Succession on Sinking Particles in the Ocean's Interior. *Front Microbiol* 8:2269.

108. Satomi M & Fujii T (2014) The Family Oceanospirillaceae.491-527.

109. Cho JC & Giovannoni SJ (2004) Cultivation and growth characteristics of a diverse group of oligotrophic marine Gammaproteobacteria. *Appl Environ Microbiol* 70(1):432-440.

110. Yan S*, et al.* (2009) Biogeography and phylogeny of the NOR5/OM60 clade of Gammaproteobacteria. *Syst Appl Microbiol* 32(2):124-139.

111. Spring S*, et al.* (2013) Taxonomy and evolution of bacteriochlorophyll a-containing members of the OM60/NOR5 clade of marine gammaproteobacteria: description of Luminiphilus syltensis gen. nov., sp. nov., reclassification of Haliea rubra as Pseudohaliea rubra gen. nov., comb. nov., and emendation of Chromatocurvus halotolerans. *BMC Microbiology* 13:118-139.

112. Duhaime MB*, et al.* (2017) Comparative Omics and Trait Analyses of Marine Pseudoalteromonas Phages Advance the Phage OTU Concept. *Front Microbiol* 8:1241.

113. Beardsley C, Pernthaler J, Wosniok W, & Amann R (2003) Are readily culturable bacteria in coastal North Sea waters suppressed by selective grazing mortality? *Applied and environmental microbiology* 69(5):2624-2630.

114. Sheik AR (2012) Viral regulation of nutrient assimilation by algae and prokaryotes. PhD (University of Bremen, Bremen).

115. Lovell CR (2017) Ecological fitness and virulence features of Vibrio parahaemolyticus in estuarine environments. *Appl Microbiol Biotechnol* 101(5):1781-1794.

116. Bowman JP, McCammon SA, Brown JL, & McMeekin TA (1998) Glaciecola punicea gen. nov., sp. nov. and Glaciecola pallidula gen. nov., sp. nov. : psychrophilic bacteria from Antarctic sea-ice habitats. *International Journal of Systematic Bacteriology* 48:1213-1222.

117. Mann AJ*, et al.* (2013) The genome of the alga-associated marine Flavobacterium Formosa agaifila KMM 3901T reveals a broad potential for degradation of algal polysaccharides. *Appl Environ Microbiol* 79:6813-6822.

118. Buchan A, LeCleir GR, Gulvik CA, & Gonzalez JM (2014) Master recyclers: features and functions of bacteria associated with phytoplankton blooms. *Nat Rev Microbiol* 12(10):686-698.

119. Choi TH, Lee HK, Lee K, & Cho JC (2007) Ulvibacter antarcticus sp. nov., isolated from Antarctic coastal seawater. *Int J Syst Evol Microbiol* 57(Pt 12):2922-2925.

120. Seo HS*, et al.* (2009) Marinoscillum gen. nov., a member of the family 'Flexibacteraceae', with Marinoscillum pacificum sp. nov. from a marine sponge and Marinoscillum furvescens nom. rev., comb. nov. *Int J Syst Evol Microbiol* 59(Pt 5):1204-1208.

121. Lauro FM*, et al.* (2009) The genomic basis of trophic strategy in marine bacteria. *Proceedings of the National Academy of Sciences* 106(37):15527-15533.

122. Boedeker C*, et al.* (2017) Determining the bacterial cell biology of Planctomycetes. *Nat Commun* 8:14853.

123. Morris RM, Longnecker K, & Giovannoni SJ (2006) Pirellula and OM43 are among the dominant lineages identified in an Oregon coast diatom bloom. *Environ Microbiol* 8(8):1361-1370.

124. Clum A*, et al.* (2009) Complete genome sequence of Pirellula staleyi type strain (ATCC 27377). *Stand Genomic Sci* 1(3):308-316.

125. Sheik CS, Jain S, & Dick GJ (2014) Metabolic flexibility of enigmatic SAR324 revealed through metagenomics and metatranscriptomics. *Environ Microbiol* 16(1):304-317.

126. Voget S*, et al.* (2015) Adaptation of an abundant Roseobacter RCA organism to pelagic systems revealed by genomic and transcriptomic analyses. *ISME J* 9(2):371-384.

127. Mayali X, Franks PJS, & Azam F (2008) Cultivation and Ecosystem Role of a Marine <em>Roseobacter</em> Clade-Affiliated Cluster Bacterium. *Applied and Environmental Microbiology* 74(9):2595-2603.

128. Giebel H-A*, et al.* (2011) Distribution of Roseobacter RCA and SAR11 lineages in the North Sea and characteristics of an abundant RCA isolate. *The ISME Journal* 5(1):8-19.

129. Romanova ND & Sazhin AF (2010) Relationships between the cell volume and the carbon content of bacteria. *Oceanology* 50(4):522-530.

130. Minghou J, Yujun W, Zuhong X, & Yucai G (1984) *Studies on the M:G ratios in alginate*Dordrecht).

131. Mackie W & Sellen DB (1969) The degree of polymerization and polydispersity of mannan from the cell wall of the green seaweed codium fragile. *Polymer* 10:621-632.

132. dos Santos MA & Grenha A (2015) Polysaccharide Nanoparticles for Protein and Peptide Delivery: Exploring Less-Known Materials. *Advances in Protein Chemistry and Structural Biology* 98:231-269.

133. Bilan MI & Usov AI (2008) Structural analysis of fucoidans. *Natural Product Communications* 3:1639-1648.

# Figures and Tables

1. Total C, N and P fluxes in/out of the system and between major ecological compartments over the 2009 – 2012 model period. Units: µmolCNP/L/d (% of photosynthesis or external input).

1. Error distribution of replicate simulations. Values shown are the minimum error obtained by sub-sampling *n* runs from a population of 896 runs. Each point represents the average of the minimum error of 100 sub-sampling trials. The line represents the least-squares fit of the function *ERR* = *ERR_min_* + *ERR_base_* × *ERR_Km_* / (*ERR_Km_* + *n*), *ERR_min_* = 0.86, *ERR_base_* = 0.16, *ERR_Km_* = 4.8.

1. Model schematic. Components (types, state variables, model species) are shown with circles. “*” indicates multiple components are in this ecological position/compartment. Processes (functions) are shown with squares. See Table S1 for key. For clarity, only showing those processes “turned on” for the Helgoland application. The phytoplankton > DOM > bacteria link is highlighted bold.

1. Model – data comparison in two-dimensional space.

1. Illustration of optimization approach. SPO = Single-parameter optimization. MPO = Multi-parameter optimization (Nelder-Mead Simplex method). MTC = Monte Carlo/Latin hypercube sampling.

1. Time-variable input (forcing functions) for Helgoland application. (A) Temperature (*T*, °C) and salinity (*SAL*, PSU). (B) Light intensity (*IT*, μE/m2/s) and photoperiod (*f*). (C) Nutrient loadings (*W*, mol/d). See Table S18 and Section S1.6.

1. Model-data comparison for ecological compartments. (A) NOX, NH4 (mmolN/L). (B) PO4 (mmolP/L). (C) SIL (mmolSi/L). (D) POM and DOM (mmolC/L). (E) Phytoplankton (μgChla/L). (F) Bacteria (1e6/mL).
2. Model processes and expression

| **Process** | **Source/Sink** | **Expression (a)**  $\frac{d}{dt}C_{i}=$ … |
| --- | --- | --- |
| Loading [w] | *Source* | $+\frac{W_{i}}{V}$ |
| Flow [q] | *Source* | $+\frac{Q}{V}C_{in,i}$ |
|  | *Sink* | $-\frac{Q}{V}C_{i}$ |
| Settling [s] | Sink | $-\frac{{vs}_{i}}{H}C_{i}$ |
| First-order [f] | *Sink (i)* | $-\left( {kf}_{i} {FTf}_{i} C_{i} \right)$ |
|  | *Source (j)* | $+{Ff}_{i,j} \left( {kf}_{i} {FTf}_{i} C_{i} \right)$ |
| Respiration [r] | *Sink (i)* | $-\left( {kr}_{i} {FTr}_{i} C_{i} \right)$ |
|  | *Source (NH4,j)* | $+{SN}_{i} \left( {kr}_{i} {FTr}_{i} C_{i} \right)$ |
|  | *Source (PO4,j)* | $+{SP}_{i} \left( {kr}_{i} {FTr}_{i} C_{i} \right)$ |
|  | *Source (SIL,j)* | $+{SSi}_{i} \left( {kr}_{i} {FTr}_{i} C_{i} \right)$ |
| Photosynthesis [p] | *Source (i)* | $+\left( {kp}_{i} {LTp}_{i} {LEp}_{i} {LIp}_{i} {LNp}_{i} {LMp}_{i} C_{i} \right)$ |
|  | *Sink (NOX, j)* | $-{SN}_{i} \left( 1-{Pp}_{i} \right) \left( {kp}_{i} {LTp}_{i} {LEp}_{i} {LIp}_{i} {LNp}_{i} {LMp}_{i} C_{i} \right)$ |
|  | *Sink (NH4, j)* | $-{SN}_{i} {Pp}_{i} \left( {kp}_{i} {LTp}_{i} {LEp}_{i} {LIp}_{i} {LNp}_{i} {LMp}_{i} C_{i} \right)$ |
|  | *Sink (PO4, j)* | $-{SP}_{i} \left( {kp}_{i} {LTp}_{i} {LEp}_{i} {LIp}_{i}{LNp}_{i} {LMp}_{i} C_{i} \right)$ |
|  | *Sink (SIL, j)* | $-{SSi}_{i} \left( {kp}_{i} {LTp}_{i} {LEp}_{i} {LIp}_{i} {LNp}_{i} {LMp}_{i} C_{i} \right)$ |
|  | *Sink (DOM, j)* | $-Smbase {Fm}_{i} \left( {kp}_{i} {LTp}_{i} {LEp}_{i} {LIp}_{i} {LNp}_{i} {LMp}_{i} C_{i} \right)$ |
| Grazing [z] | *Source,*  *biomass accumulation (i)* | $+{ez}_{i} {LNzt}_{i}\left( {kz}_{i} {LTz}_{i} {LEz}_{i} C_{i} \right)$ |
|  | *Source,*  *sloppy feeding (POM, DOM, k)* | $+\left( 1-{ez}_{i} \right) {Fx}_{j,k} {LNz}_{i,j} \left( {kz}_{i} {LTz}_{i} {LEz}_{i} C_{i} \right)$ |
|  | *Source, Si (BSi, k)* | $+{SSi}_{j} {LNz}_{i,j} \left( {kz}_{i} {LTz}_{i} {LEz}_{i} C_{i} \right)$ |
|  | *Sink (j)* | $-{LNz}_{i,j} \left( {kz}_{i} {LTz}_{i}{LEz}_{i} C_{i} \right)$ |
| Heterotrophy [h] | *Source,*  *biomass accumulation (i)* | $+{Yh}_{i} {LNht}_{i}\left( {kh}_{i} {LTh}_{i} {LEh}_{i} {LIh}_{i} C_{i} \right)$ |
|  | *Source, NH4 (j)* | $+{SN}_{i} \left( 1-{Yh}_{i} \right) {LNht}_{i}\left( {kh}_{i} {LTh}_{i} {LEh}_{i} {LIh}_{i} C_{i} \right)$ |
|  | *Source, PO4 (j)* | $+{SP}_{i} \left( 1-{Yh}_{i} \right) {LNht}_{i}\left( {kh}_{i} {LTh}_{i} {LEh}_{i} {LIh}_{i} C_{i} \right)$ |
|  | *Sink (DOM, j)* | $-{LNh}_{i,j} \left( {kh}_{i} {LTh}_{i} {LEh}_{i} {LIh}_{i} C_{i} \right)$ |
| Viral [v] | *Source, virus (i)* | ${betav}_{i} \left( {kv}_{i,j}C_{i} C_{j} \right)$ |
|  | *Source, carcass (POM, DOM, k)* | $+\left( 1-{betav}_{i} \right) {Fx}_{j,k} \left( {kv}_{i,j}C_{i} C_{j} \right)$ |
|  | *Source, Si (BSi, k)* | $+ {SSi}_{j} \left( {kv}_{i,j}C_{i} C_{j} \right)$ |
|  | *Sink (j)* | $-\left( {kv}_{i,j}C_{i} C_{j} \right)$ |
| Exudation [e] (b) | *Sink (i)* | $-\left( \left[ {ke}_{i}+{ef}_{i} {kp}_{i} {LTp}_{i} {LIp}_{i} {LNp}_{i} {LMp}_{i} \right] {{LEe}_{i} C}_{i} \right)$  $-\left( \left[ {ke}_{i}+{ef}_{i} {Yh}_{i} {kh}_{i} {LTh}_{i} {LNht}_{i} \right] {{LEe}_{i} C}_{i} \right)$ |
|  | *Source (SIL,j)* | $+{SSi}_{i} \left( \left[ {ke}_{i}+{ef}_{i} {kp}_{i} {LTp}_{i} {LIp}_{i} {LNp}_{i} {LMp}_{i} \right] {LEe}_{i} C_{i} \right)$  $+{SSi}_{i} \left( \left[ {ke}_{i}+{ef}_{i} {Yh}_{i} {kh}_{i} {LTh}_{i} {LNht}_{i} \right] {LEe}_{i}C_{i} \right)$ |
|  | *Source (DOM, j)* | $+{Fe}_{i,j} \left( \left[ {ke}_{i}+{ef}_{i} {kp}_{i} {LTp}_{i} {LIp}_{i} {LNp}_{i} {LMp}_{i} \right] {LEe}_{i} C_{i} \right)$  $+{Fe}_{i,j} \left( \left[ {ke}_{i}+{ef}_{i} {Yh}_{i} {kh}_{i} {LTh}_{i} {LNht}_{i} \right] {LEe}_{i} C_{i} \right)$ |
| Death [y] | *Sink (i)* | $-\left( {ky}_{i} C_{i} \right)$ |
|  | *Source (j)* | $+{Fx}_{i,j} \left( {ky}_{i} C_{i} \right)$ |
| Loss [u] | *Sink (i)* | $-\left( {ku}_{i} {LTu}_{i} {LTYu}_{i} {LEu}_{i} {LIu}_{i} C_{i} \right)$ |
|  | *Source (j)* | $+{Fx}_{i,j} \left( {ku}_{i} {LTu}_{i} {LTYu}_{i} {LEu}_{i} {LIu}_{i} C_{i} \right)$ |
| Inhibition [i] | *Sink (i)* | $- \left( {LNit}_{i} {ki}_{i} C_{i} \right)$ |
|  | *Source (DOM, j)* | $+{Fx}_{i,j} \left( {LNit}_{i} {ki}_{i} C_{i} \right)$ |

(a) Limitation and other factors are defined in Table S2.

(b) Two formulations are for photosynthesis and heterotrophy, respectively.

1. Limitation and other factors

| **Process** | **Expression** |
| --- | --- |
| First-order [f] | *Temperature factor:*  $FTf={thetaf}^{\left( T-20 \right)}$ |
| Respiration [r] | *Temperature factor:* As first-order. |
| Photosynthesis (p) | *Temperature limitation:*  $LTp=\left\{ \begin{aligned} exp\left( -beta1p \left( T-Toptp \right)^{2} \right) if T\leq Toptp \\ exp\left( -beta2p \left( Toptp-T \right)^{2} \right) if T>Toptp \end{aligned} \right.$  *Environmental factor limitation:* As temperature limitation.  *Light limitation:*  $LI=\frac{2.718 f}{kext H}\left( exp\left[ -alpha1 \right]-exp\left[ -alpha0 \right] \right)$  $alpha0=\frac{Ia}{Is}exp\left[ -kext H1 \right]$  $alpha1=\frac{Ia}{Is}exp\left[ -kext H2 \right]$  $Ia=\frac{IT}{f}$  $kext=0.052 Nk+0.174 Dk+0.031 Pk$  $Pk=\sum_{i=1}^{n} {SChla}_{i} C_{i}$  $Dk=\sum_{i=1}^{n} {wDk}_{i} C_{i}$  *Nutrient limitation:*  ${LNp}_{j}=\frac{C_{j}}{{Ksp}_{j}+C_{j}};j=DIN, PO4, SIL$  $DIN=NOX+NH4$  $LNp=min\left( {LNp}_{j} \right)$  *Micronutrient limitation:*  ${LMp}_{j}=\frac{C_{j}}{{Ksp}_{j}+C_{j}};j=some DOM$  ${Ksp}_{j}={Fm}_{j} KspMbase$  $LMp=\min\left( {LMp}_{j} \right)$  *Ammonia preference:*  $Pp=\frac{NH4}{Kam+NH4}$ |
| Grazing [z] | *Temperature limitation:* As photosynthesis.  *Environmental factor limitation:* As temperature limitation.  *Substrate limitation:* As heterotrophy. |
| Heterotrophy [h] | *Temperature limitation:* As photosynthesis.  *Environmental factor limitation:* As temperature limitation.  *Light limitation:* As temperature limitation, based on Iavg.  $Iaout=Ia exp\left[ -kext H2 \right]$  $Iaavg=\frac{Ia-Iaout}{\ln\left( Ia \right)-\ln\left( Iaout \right)}$  *Substrate limitation:*  ${LNh}_{i,j}=\frac{{C_{j}}/{{Ksh}_{i,j}}}{1+\sum_{j=1}^{n} {C_{j}}/{{Ksh}_{i,j}}}$  ${LNht}_{i}=\sum_{j=1}^{n} {LNh}_{i,j}$ |
| Viral [v] | - |
| Exudation [e] | *Environmental factor limitation:* As temperature limitation. |
| Death [y] | $ky=kybase\left( \frac{C}{Cref} \right)^{xny}$ |
| Loss [u] | *Temperature limitation:* As photosynthesis.  *Time-of-year (TY) limitation:*  $LTYu=\left\{ \begin{aligned} exp\left( -beta1TYu \left( TY-TYopt1u \right)^{2} \right) if TY\leq TYopt1u \\ 1 if TYopt1u<TY\leq TYopt2u \\ exp\left( -beta2TYu \left( TYopt2u-TY \right)^{2} \right) if TY>TYopt2u \end{aligned} \right.$  *Environmental factor limitation:* As temperature limitation.  *Light limitation:* As temperature limitation. |
| Inhibition [i] | *Inhibitor limitation:*  ${LNi}_{i,j}=\frac{{C_{j}}/{{Ksi}_{i,j}}}{1+\sum_{j=1}^{n} {C_{j}}/{{Ksi}_{i,j}}}$  ${LNit}_{i}=\sum_{j=1}^{n} {LNi}_{i,j}$ |

1. Process parameters: General properties and flow [q]

| **Symbol** | **Units** | **Description & notes (a)** |
| --- | --- | --- |
| *Cic* | μmol*/L | Initial concentration (b) (c)  *Application:*  nox: 8.1, nh4: 1.3, po4: 0.47, sil: 5.8 (d)  bsi: 2.6 (e)  nkx: 4.0 (f)  ph1, eux: *Cflr*  p01: 6.4, d01: 93 (g)  m01: 1e-6, i01: 1e-8 (h) |
| *Cin* | μmol*/L | Inflow concentration (c)  Note: nox, po4 and sil added as time-variable mass loading, see Section S1.6.  *Application:*  nkx 4.0 (0, 4) (f)  d01, m01, i01: *Cic*/10 (i)  Others: 0 |
| *Cflr* | μmol*/L | Floor concentration (c)  *Literature:*  Phytoplankton: <0.42 (j)(k)  Bacteria: <1.1 (j)(k)  *Application:*  nox: 1e-4, nh4: 5e-5, po4: 5e-5, sil: 5e-4 (l)  ph1: 0.055 (l)  eux: 0.70 (l)  p01, d01: *Cic*/10; m01 = *Cic*/100 (i)  Others: 0 |
| *SChla* | μgChl*a*/mmolC | Chlorophyll *a* content  Note: Phytoplankton only  *Literature:*  Phytoplankton:  **120** – 340, range summarized by [Hellweger and Lall (10)](#_ENREF_10).  120 – **600**, range for models, [Chapra (11)](#_ENREF_11).  Coastal waters: 130 – 800, [Jakobsen and Markager (12)](#_ENREF_12)  *Application:*  Phytoplankton: (120, 600)  Load calculation (see Section S1.6): 310 |
| *SCopy* | copies/cell | Gene copy content  *Application:* Not used |
| *SN* | mmolN/mmolC | N content  Note: Organic material only  *Literature:* Redfield = 0.15  *Application:* 0.15 (m) |
| *SP* | mmolP/mmolC | P content  Note: Organic material only  *Literature:* Redfield = 0.0094  *Application:* 0.0094 (m) |
| *SSi* | mmolSi/mmolC | Si content  *Literature:* **0.21** – **0.54** (n)  *Application:*  Diatoms and silicioflagellates: (0.21, 0.54)  Others: 0  Load calculation (see Section S1.6): 0.15 (applied to Chl. *a*) |
| *MC* | molC/cell | Cell size  *Literature:*  Bacteria:  3.3e-15 – **2.7e-14**, range used in model, [Weitz et al. (8)](#_ENREF_8).  **5.4e-16** – 2.5e-14 (o)  Phytoplankton:  **3.3e-15** – 2.7e-14, range used in model, [Weitz et al. (8)](#_ENREF_8).  3.8e-15 – 1.8e-14, marine cyanobacteria, ([13](#_ENREF_13)).  9.7e-13 – 6.3e-10, various species, Table S22.  Zooplankton:  **3.3e-10** – **2.7e-9**, range used in model, [Weitz et al. (8)](#_ENREF_8).  Virus:  **3.3e-18** – **1.3e-16**, range used in model, [Weitz et al. (8)](#_ENREF_8).  *Application:*  Bacteria: See Table S23. (p)  Phytoplankton: See Table S22. (p) |
| *Cref* | mmol*/L | Reference concentration  *Application:* not used |
| *wDk* | mgS/mmolC | Solids:carbon conversion  *Application:* 12 mgC/mmolC / (0.4 mgC/mgS) for all POM species |
| *Fx_j_* | - | Sloppy feeding, viral carcass or death fraction going to component *j*  *Application:* (0, 1)**  See Table S4 for polysaccharide composition of phytoplankton |

(a) Value or range (min, max) if parameter subject to optimization. Values in **bold** indicate min/max of literature range. Values marked with “*” indicate range was extended from lumped model or observation (see Section S2.3). Values marked with “♥” indicate value was adjusted to allow for larger time step. Values marked with “♦” indicate value was adjusted to allow for optimization routine to “turn off” this process. Source fractions (e.g., *Fx*) marked with “**” are relative and thus normalized so that the sum is 1. Constraint on derived parameters marked with “***”.

(b) The simulation includes a 1-year “spin-up” period using forcing functions from the first year, and the initial concentrations do not affect the results substantially.

(c) Concentrations are specified for initial component at first de-lump level (corresponding to entire ecological compartment) and adjusted at subsequent de-lump levels.

(d) Initial value of Helgoland observations ([14](#_ENREF_14)).

(e) Set to POM * *SSi* from table above.

(f) *Cic* and max. *Cin* based on max. SD value of Helgoland observations ([14](#_ENREF_14)), converted using kext = 1.8 / SD ([15](#_ENREF_15)) and kext = 0.052 Nk (Table S2).

(g) Min. of Helgoland observations ([16](#_ENREF_16)).

(h) Assigned based on typical values in model (following spin-up period).

(i) Trace concentrations supplied in inflow and set as floor concentration, helps to stabilize the system.

(j) Based on min. 0.1 μgChl*a*/L and 2.1e5 no./mL total bacteria (from Helgoland data).

(k) Converted using 240 μgChl*a*/mmolC (see *SChla* parameter in Table above), 5e-15 molC/cell cell size (typical bacteria, see above).

(l) Found to produce good results in Helgoland application.

(m) The present version does not support variable stoichiometry. In other words, all organic material is at fixed Redfield composition. Variable stoichiometry is a potential future expansion, following [Hellweger and Lall (10)](#_ENREF_10).

(n) Based on 0.2-0.5 gSi/gDW from [Chapra (11)](#_ENREF_11) and 0.4 gC/gDW.

(o) Range based on SAR11 (6.5 fgC/cell, 5.4e-16 molC/cell, [White et al. (17)](#_ENREF_17)) and max. of literature values summarized (2.5e-14 molC/cell, [Hellweger et al. (18)](#_ENREF_18)).

(p) MH types optimization in range /2 to *2.

1. Polysaccharide composition of phytoplankton (a)

| **Polysaccharide/**  **Phytoplankton**  **(ID)** | **chitin**  **(chi)** | **chrysolaminarin**  **(chr)** | **alginate**  **(n/a)** | **glycogen**  **(gly)** | **xylan**  **(xyl)** | **cellulose**  **(cel)** | **mannan**  **(man)** | **carrageenan**  **(n/a)** | **chondroitin sulfate**  **(n/a)** | **starch**  **(sta)** | **pectin**  **(pec)** | **fucoidan**  **(n/a)** | **glucoro-mannan**  **(glo)** | **FCSP (fucose-containing PS)**  **(fcs)** | **rhamnan**  **(rhm)** | **Note** |
| --- | --- | --- | --- | --- | --- | --- | --- | --- | --- | --- | --- | --- | --- | --- | --- | --- |
| ***Diatoms***  **(dip, dix, mhe,**  **cde, cmi, rst, tno)** | +  (5) | +  (30) | - | - | +  (2.5) | +  (2.5) | +  (2.5) | - | - | - | - | - | +  (5) | +  (2.5) | - | ([19-31](#_ENREF_19)) |
| ***Green algae***  **(gre)** | +  (2.5) | +  (2.5) | - | - | +  (3.3) | +  (5.8) | +  (3.3) | - | - | +  (25) | +  (2.5) | - | - | - | +  (2.5) | ([19-21](#_ENREF_19), [23](#_ENREF_23), [24](#_ENREF_24), [26-29](#_ENREF_26), [31-35](#_ENREF_31)) |
| **Dinoflagellates**  **(dxf, din)** | - | - | - | - | - | +  (10) | - | - | - | +  (25) | - | - | - | - | - | ([21](#_ENREF_21), [29](#_ENREF_29), [36](#_ENREF_36)) |
| ***Silicoflagellates***  **(sif)** | +  (5) | +  (25) | - | - | - | +  (5) | - | - | - | - | - | - | - | - | - | ([19-21](#_ENREF_19), [23](#_ENREF_23), [24](#_ENREF_24), [26](#_ENREF_26), [27](#_ENREF_27), [29](#_ENREF_29), [34](#_ENREF_34), [37-39](#_ENREF_37)) |
| ***Coccolithophorids,***  ***Prymnesiophyceae***  **(coc, pha)** | +  (2.5) | +  (25) | - | - | - | +  (2.5) | - | - | - | - | +  (2.5) | - | - | - | - | ([19-21](#_ENREF_19), [23](#_ENREF_23), [24](#_ENREF_24), [26-31](#_ENREF_26), [34](#_ENREF_34), [40](#_ENREF_40), [41](#_ENREF_41)) |
| ***Ciliates***  **(n/a)** | +  (5) | - | - | +  (25) | - | - | - | - | - | - | - | - | - | - | - | ([19](#_ENREF_19), [42](#_ENREF_42), [43](#_ENREF_43)) |
| ***Chattonella***  **(cha)** | - | +  (25) | - | - | - | - | - | - | - | - | - | - | - | - | - | ([19-21](#_ENREF_19), [23](#_ENREF_23), [24](#_ENREF_24), [26-29](#_ENREF_26), [31](#_ENREF_31), [34](#_ENREF_34)) |

(a) Values are percentage of carbon pool. The composition was based on a literature survey on cell components, exopolymers and storage glucans. The cell wall was calculated as 10% of the cellular carbon content ([40](#_ENREF_40)). If a compound was produced, but no quantitative estimates provided, 2.5% was used.

1. Process parameters: Settling [s]

| **Symbol** | **Units** | **Description & notes (a)** |
| --- | --- | --- |
| *vs* | m/d | Settling velocity  Note: Phytoplankton and POM (POC and BSi) only  *Literature:*  Phytoplankton:  **0** – **2**, range summarized by [Hellweger and Lall (10)](#_ENREF_10).  0 – 2, model range, [Schnoor (44)](#_ENREF_44)  POM:  **0.01** – **2.3**, range summarized by [Hellweger and Lall (10)](#_ENREF_10).  0.01 – 0.3, model range, [Schnoor (44)](#_ENREF_44)  *Application:*  Phytoplankton: (0, 4*)  POM: (0.005*, 4.6*)  Load calculation (see Section S1.6): 1.5 |

(a) See footnote in Table S3.

1. Process parameters: First-order [f]

| **Symbol** | **Units** | **Description & notes (a)** |
| --- | --- | --- |
| *kf* | 1/d | Rate constant at T = 20°C  *Literature:*  Biogenic silicate dissolution: **0.01 – 0.25**,  range summarized by [Hellweger and Lall (10)](#_ENREF_10).  Viral decay: 0.05 – 5, range used in model, [Weitz et al. (8)](#_ENREF_8).  *Application:*  Biogenic silicate dissolution: (0.005*, 0.5*)  Inhibitors: (0.0, 0.5) |
| *thetaf* | - | Temperature exponent  *Application:*  Biogenic silicate dissolution: (1, 1.3)  Inhibitors: (1, 1.3) |
| *Ff_j_* | - | Fraction to component *j*  *Application:* (0, 1)** |

(a) See footnote in Table S3.

1. Process parameters: Respiration [r]

| **Symbol** | **Units** | **Description & notes (a)** |
| --- | --- | --- |
| *kr* | 1/d | Rate constant at T = 20°C  *Literature:*  Phytoplankton:  **0.001** – 0.1, range used in model, [Weitz et al. (8)](#_ENREF_8).  0.01 – **0.25**, range summarized by [Hellweger and Lall (10)](#_ENREF_10).  Bacteria:  0+ (b)  0.07 − 0.15, range used in model applications, [Connolly et al. (45)](#_ENREF_45). 0.001 – 0.1, range used in model, [Weitz et al. (8)](#_ENREF_8).  Zooplankton:  0.025 – 0.1, range used in model, [Weitz et al. (8)](#_ENREF_8) (c).  *Application:*  Phytoplankton: (0.0005*, 0.5*)  Bacteria: considered with heterotrophy. |
| *thetar* | - | Temperature exponent  *Literature:* 1.08, summary by [Hellweger and Lall (10)](#_ENREF_10).  *Application:* (1, 1.3) |

(a) See footnote in Table S3.

(b) As low as zero considering basal metabolism can be supported by rhodopsins ([46](#_ENREF_46)).

(c) This model also includes respired fraction on grazed mass.

1. Process parameters: Photosynthesis [p]

| **Symbol** | **Units** | **Description & notes (a)** |
| --- | --- | --- |
| *kp* | 1/d | Max. photosynthesis rate  *Literature:*  0.69 – 3.6, μmax, various species, [Di Toro (47)](#_ENREF_47).  1.0 – 2.0, μmax, model range, [Schnoor (44)](#_ENREF_44).  0.75 – 1.0, μmax, various species, [Morel (48)](#_ENREF_48) (b).  **0.2** – 2.0, range used in model, [Weitz et al. (8)](#_ENREF_8).  1.0 – 2.5, range summarized by [Hellweger and Lall (10)](#_ENREF_10).  < **5.0**, μmax, based on observed concentrations at Helgoland ([14](#_ENREF_14))  *Application:* (0.1*, 6*) |
| *Toptp* | °C | Optimum temperature  *Literature:*  6 – 18, range summarized by [Hellweger and Lall (10)](#_ENREF_10).  **1** – **19**, temperature range at Helgoland.  *Application:* (1, 19) |
| *beta1p* | - | Temperature limitation exponent 1  *Literature:* 0.003 – 0.005, range summarized by [Hellweger and Lall (10)](#_ENREF_10).  *Application:* (0, 0.2) |
| *beta2p* | - | Temperature limitation exponent 2  *Literature:* 0 – 0.006, range summarized by [Hellweger and Lall (10)](#_ENREF_10).  *Application:* (0, 0.2) |
| *LTp* | - | Temperature limitation factor  *Application:* (1e-3, 1)*** |
| *E1optp* | PSU | Optimum salinity  *Literature:*  28 – 35, salinity range at Helgoland.  *Application:* (28, 35) |
| *beta1E1p* | - | Salinity limitation exponent 1  *Application:* (0, 1) (F) |
| *beta2E1p* | - | Salinity limitation exponent 2  *Application:* (0, 1) (F) |
| *Is* | μE/m^2^/s | Saturating light intensity  *Literature:* **10** – **300**, range summarized by [Hellweger and Lall (10)](#_ENREF_10).  *Application:* (5*, 600*) |
| *KspDIN* | μmolN/L | DIN half-saturation constant  *Literature:*  **0.018** – 0.2, various species, [Di Toro (47)](#_ENREF_47).  0.36 – 1.4, model range, [Chapra (11)](#_ENREF_11).  0.071 – 2.1, model range, [Schnoor (44)](#_ENREF_44).  0.05 – 10, range used in model, [Weitz et al. (8)](#_ENREF_8).  0.036 – **11.6**, extreme range, [Sommer (49)](#_ENREF_49).  *Application:* (0.36♥, 12) |
| *KspPO4* | μmolP/L | PO4 half-saturation constant  *Literature:*  0.01 – 0.25, various species, [Di Toro (47)](#_ENREF_47).  0.032 – 0.16, model range, [Chapra (11)](#_ENREF_11).  0.19 – 0.81, model range, [Schnoor (44)](#_ENREF_44).  **0.003** – 1.83, extreme range, [Sommer (49)](#_ENREF_49).  0.5 – **4.0**, various species, [Morel (48)](#_ENREF_48).  *Application:* (0.032♥, 4.0) |
| *KspSIL* | μmolSi/L | SIL half-saturation constant  Note: Diatoms and silicioflagellates only  *Literature:*  **0.71** – 2.9, model range, [Chapra (11)](#_ENREF_11).  0.88 – **88.7**, extreme range, [Sommer (49)](#_ENREF_49).  1.8 – 3.6, model range, [Schnoor (44)](#_ENREF_44).  0.54 – **3.9**, various species, [Di Toro (47)](#_ENREF_47).  *Application:* (0.71♥, 89) |
| *KspMbase* | mmolC/L | Micronutrient half-saturation constant, base value  *Application:* 3.5e-11 (e) |
| *Kam* | μmolN/L | NH4 preference constant  *Literature:*  3.6 (d).  0.71 – 2.1 (c).  *Application:* (1.2, 40) |
| *Smbase* | molC/molC | Micronutrient quota, base value  *Application:* 7.5e-10 (e) |
| *Fm_j_* | - | Micronutrient fraction for component *j*  *Application:* (0, 1) |

(a) See footnote in Table S3.

(b) Calculated using Eq. 4 in Table 1 of reference.

(c) NH4 concentration corresponding to start of NO3 uptake, Table IV of ([50](#_ENREF_50)).

(d) *Kam* literature value based on Table 35.4 of [Chapra (11)](#_ENREF_11). *Kam* model range based on *KsN* model range scaled by *Kam*/*KsN* = 50/15 from Table 35.4 of [Chapra (11)](#_ENREF_11).

(e) Parameters for micronutrients are poorly constrained. For bacteria, the basal exudation rate (*ke*) was set to an arbitrary small value and the exudation fraction (*ef*) was adjusted so that basal and proportional terms are about equivalent (see Table S13). For phytoplankton, the quota (*Smbase*) and half-saturation constant (*KspMbase*) were adjusted to have phytoplankton uptake at 50% of the total micronutrient loss and a micronutrient limitation factor (*LMp*) of about 0.85.

(F) Max. beta values based on those for temperature, adjusted for different magnitude.

1. Process parameters: Grazing [z]

| **Symbol** | **Units** | **Description & notes (a)** |
| --- | --- | --- |
| *kz* | 1/d | Max. grazing rate  *Literature:*  8.5e-4 – 2.1, range summarized by [Hellweger and Lall (10)](#_ENREF_10) (c).  3.8e-2 – 3.8, range used in model, [Weitz et al. (8)](#_ENREF_8) (b) (c).  *Application:* not used |
| *Toptz* | °C | Optimum temperature  *Literature:* 1 – 19, range at Helgoland.  *Application:* not used |
| *beta1z* | - | Temperature limitation exponent 1  *Application:* not used |
| *beta2z* | - | Temperature limitation exponent 2  *Application:* not used |
| *LTz* | - | Temperature limitation factor  *Application:* not used |
| *ez_j_* | - | Assimilation fraction for component *j*  *Literature:*  0.4, value used in model, [Weitz et al. (8)](#_ENREF_8) (d).  0.4 – 0.8, range summarized by [Hellweger and Lall (10)](#_ENREF_10).  *Application:* not used |
| *Ksz_j_* | mmolC/L | Half-saturation constant for component *j*  *Literature:* 0.0083 – 0.10, range summarized by [Hellweger and Lall (10)](#_ENREF_10) (e).  *Application:* not used |

(a) See footnote in Table S3.

(b) Based on *Ψ_Z*_* = 1e-6 – 1e-4 L/zoopl/d, converted using 2e-4 μmolN/zoopl ([8](#_ENREF_8)) and 0.15 molN/molC (Redfield).

(c) Converted from grazing rate in L/mmolC/d and *Ksz* = 0.05 mmolC/L (see table entry).

(d) In this model the non-growth fraction is broken out into “egested” and “respired”.

(e) Converted using 240 μgChl*a*/mmolC, see Table S3.

1. Process parameters: Heterotrophy [h]

| **Symbol** | **Units** | **Description & notes (a)** |
| --- | --- | --- |
| *kh* | 1/d | Max. heterotrophy rate  *Literature:*  0.96 – 3.6, range summarized by [Mentges et al. (51)](#_ENREF_51).  1.4 – 5.6, range used in model, [Weitz et al. (8)](#_ENREF_8) (b).  1.8 – 13, range used in model applications, [Connolly et al. (45)](#_ENREF_45) (f).  5.0 – 11, range of literature values, [Bucci et al. (52)](#_ENREF_52) (d).  13, Delaware coastal waters, [Kirchman (53)](#_ENREF_53) (e).  1.1 – 46, predicted from codon usage bias, [Long et al. (54)](#_ENREF_54) (f).  **0.18** – 290, [Vieira-Silva and Rocha (55)](#_ENREF_55) (f).  < **3.1**, based on observed concentrations at Helgoland ([14](#_ENREF_14)).  *Application:* (0.18, 1.5♥) |
| *Topth* | °C | Optimum temperature  *Literature:* **1** – **19**, range at Helgoland.  *Application:* (1, 19) |
| *beta1h* | - | Temperature limitation exponent 1  *Application:* (0, 0.2) |
| *beta2h* | - | Temperature limitation exponent 2  *Application:* (0, 0.2) |
| *LTh* | - | Temperature limitation factor  *Application:* (1e-3, 1)*** |
| *E1opth* | PSU | Optimum salinity  *Literature:*  28 – 35, salinity range at Helgoland.  *Application:* (28, 35) |
| *beta1E1h* | - | Salinity limitation exponent 1  *Application:* (0, 1) (F) |
| *beta2E1h* | - | Salinity limitation exponent 2  *Application:* (0, 1) (F) |
| *Iopth* | μE/m^2^/s | Optimum light intensity  *Literature:*  30 – 350, light intensity range at Helgoland, est. from model.  *Application:* (30, 350) |
| *beta1Ih* | - | Light limitation exponent 1  *Application:* (0, 0.0005) (F) |
| *beta2Ih* | - | Light limitation exponent 2  *Application:* (0, 0.0005) (F) |
| *Ksh_j_* | μmolC/L | Half-saturation constant for component *j*  *Literature:*  0.043 – 0.38, range used in model applications, [Bucci et al. (52)](#_ENREF_52).  1.7 – 6.7, range used in model, [Weitz et al. (8)](#_ENREF_8) (g).  ~6.0 *E. coli* ABC transporters (h).  4.4 – 17 (i).  6 – 450, range summarized by [Mentges et al. (51)](#_ENREF_51).  **17** – 1,500, range used in model applications, [Connolly et al. (45)](#_ENREF_45).  *Application:* (17♥, 100,000♦)  See Table S11 for substrate utilization spectra of bacteria. |
| *Yh* | - | Yield coefficient  *Literature:*  0.05 – 0.2, range used in model, [Weitz et al. (8)](#_ENREF_8).  < 0.01 – 0.32, BGE for freshwater isolates, [Muscarella et al. (56)](#_ENREF_56).  < **0.05** – 0.6, BGE for natural planktonic bacteria, [Giorgio and Cole (57)](#_ENREF_57).  0.20 – 0.52, range used in model applications, [Connolly et al. (45)](#_ENREF_45).  0.09 – 0.33, range summarized by [Mentges et al. (51)](#_ENREF_51).  0.8 – **0.9**, high quality substrates, [Connolly et al. (45)](#_ENREF_45).  *Application:* (0.025*, 0.9) |

(a) See footnote in Table S3.

(b) Based on *k_G,max_* = 0.50 – 2.0 (f).

(c) Based on *k_G,max_* = 0.65 – 4.5 (f).

(d) Based on *k_G,max_* = 1.8 – 3.8 (f).

(e) Based on *k_G,max_* = 4.6 (f).

(f) Converted using *Yh = 0.36*.

(F) see footnote in Table S8.

(g) Converted using 0.15 molN/molC (Redfield).

(h) Based on “*K_m_* values of *E. coli* ABC transporters for sugars are in the range of 1 µM” [Ihssen and Egli (58)](#_ENREF_58), converted using 180 g/mol and 6 molC/mol.

(i) Based on “most high affinity uptake systems have *K_m_* values in the range of 100 – 500 µg L^-1^ for sugars” [Egli (59)](#_ENREF_59).

1. Polysaccharide utilization spectra of bacteria (a)

| **Polysaccharide/**  **Bacteria**  **(ID)** | **chitin**  **(chi)** | **chrysolaminarin**  **(chr)** | **alginate**  **(n/a)** | **glycogen**  **(gly)** | **xylan**  **(xyl)** | **cellulose**  **(cel)** | **mannan**  **(man)** | **carrageenan**  **(n/a)** | **chondroitin sulfate**  **(n/a)** | **starch**  **(sta)** | **pectin**  **(pec)** | **fucoidan**  **(n/a)** | **glucuromannan**  **(glo)** | **FCSP**  **(fcs)** | **rhamnan**  **(rhm)** | **Note** |
| --- | --- | --- | --- | --- | --- | --- | --- | --- | --- | --- | --- | --- | --- | --- | --- | --- |
| **SAR11**  **(s11)** | -  G:0/1 | -  G:0/1 | -  G:0/1 | -  G:0/1 | -  G:0/1 | -  G:0/1 | -  G:0/1 | -  G:0/1 | -  G:0/1 | -  G:0/1 | -  G:0/1 | -  G:0/1 | -  G:0/1 | -  G:0/1 | -  G:0/1 | (b1) |
| **Roseobacter, RCA**  **- and DC5-80-3**  **- clades of Roseobacter**  **(rox, nac, rca)** | -  G:0/1 | -  G:0/1 | -  G:0/1 | +  G:1/1 | -  G:0/1 | -  G:0/1 | -  G:0/1 | +  G:1/1 | -  G:0/1 | +  G:1/1 | -  G:0/1 | -  G:0/1 | -  G:0/1 | -  G:0/1 | -  G:0/1 | (b2) |
| **Reinekea**  **(rei)** | -  P:0/1  M:0/1  G:0/1 | -  P:0/1  M:0/1  G:0/1 | -  G:0/1  M:0/1 | +  P:1/1  G:1/1  M:1/1 | -  P:0/1  G:0/1  M:0/1 | -  P:0/1  G:0/1  M:0/1 | +  P:1/1  G:1/1  M:1/1 | -  P:0/1  G:0/1  M:0/1 | n/a | +  P:1/1  G:1/1  M:1/1 | -  P:0/1  G:0/1  M:0/1 | -  G:0/1  M:0/1 | -  G:0/1  M:0/1 | -  G:0/1  M:0/1 | -  G:0/1  M:0/1 | (b3) |
| **Pseudo-**  **alteromonas**  **(psa)** | -  G:0/1 | +  G:1/1 | +  G:1/1 | +  G:1/1 | -  G:0/1 | -  G:0/1 | -  G:1/1 | +  G:1/1 | -  G:0/1 | +  G:1/1 | -  G:0/1 | +  G:1/1 | -  G:0/1 | +  G:1/1 | -  G:0/1 | (b4) |
| **Alteromonas**  **(alt)** | -  G:0/1 | +  G:1/1  P:1/1 | +  G:1/1  P:1/1 | +  G:1/1 | +  G:1/1 | -  G:0/1 | -  G:0/1 | -  G:0/1 | -  G:0/1 | +  G:1/1 | +  G:1/1 | -  G:0/1  P:0/1 | -  G:0/1 | -  G:0/1 | -  G:0/1 | (b5) |
| **Vibrio**  **(gv8)** | +  G:1/1 | -  G:0/1 | +  G:1/1 | +  G:1/1 | -  G:0/1 | -  G:0/1 | -  G:0/1 | -  G:0/1 | n/a | +  G:1/1 | -  G:0/1 | -  G:0/1 | -  G:0/1 | -  G:0/1 | -  G:0/1 | (b6) |
| **SAR86 clade**  **(s86)** | n/a | +  M:1/1 | n/a | n/a | n/a | +  M:1/1 | n/a | n/a | n/a | n/a | n/a | n/a | n/a | n/a | n/a | (b7) |
| **Glaciecola**  **(gla)** | -  G:0/1 | +  G:1/1 | -  G:0/1 | +  G:1/1 | -  G:0/1 | -  G:0/1 | -  G:0/1 | -  G:0/1 | n/a | +  G:1/1 | -  G:0/1 | -  G:0/1 | -  G:0/1 | -  G:0/1 | -  G:0/1 | (b8) |
| **Polaribacter**  **(pol)** | -  M:0/6 | +  M:18/18  R:2/2  P:2/2 | +  M:8/18  R:4/4 | +  M:9/18  P:2/2 | +  M:8/18  P:1/2  R:5/5 | +  P:2/2 | +  M:9/18  R:4/4 | -  M:1/6  P:2/2 | +  P:2/2  R:2/2 | +  M:9/18  P:2/2 | -  M:0/5  G:0/5 | -  M:2/6 | -  M:5/18 | -  M:2/6 | n/a | (b9) |
| **Formosa**  **(fox, fob)** | -  G:0/3  M:0/3 | +  P:1/1  G:3/3  M:3/3  R:1/1 | -  G:0/3  M:0/3 | -  G:0/3  M:0/3 | -  G:1/3  M:1/3 | -  G:0/3  M:0/3 | +  G:2/3  M:2/3 | -  G:0/3  M:0/3 | n/a | -  G:0/3  M:0/3 | -  G:0/3  M:0/3 | -  G:0/3  M:0/3 | +  G:3/3  M:3/3 | -  G:0/3  M:0/3 | -  G:0/3  M:0/3 | (b10) |
| **Ulvibacter**  **(ulv)** | -  G:0/1 | -  G:0/1 | +  G:1/1  M:1/1 | -  G:0/1 | -  G:0/1 | -  G:0/1 | -  G:0/1 | -  G:0/1 | n/a | -  G:0/1 | -  G:0/1 | -  G:0/1 | -  G:0/1 | -  G:0/1 | -  G:0/1 | (b11) |
| **Cryomorphaceae**  **(vis)** | -  M:0/1 | +  M:1/1 | -  M:0/1 | +  M:1/1 | -  M:0/1 | -  M:0/1 | -  M:0/1 | -  M:0/1 | n/a | +  M:1/1 | -  M:0/1 | -  M:0/1 | -  M:0/1 | -  M:0/1 | -  M:0/1 | (b12) |
| **NS3a marine group**  **(ns3)** | -  M:0/2 | +  M:1/2 | -  M:0/2 | +  M:2/2 | -  M:0/2 | -  M:0/2 | +  M:1/2 | -  M:0/2 | n/a | +  M:2/2 | -  M:0/2 | -  M:0/2 | +  M:1/2 | -  M:0/2 | -  M:0/2 | (b12b) |
| **NS5 marine group,**  **clade DE2 and VIS1**  **(nde, nvi)** | -  M:0/2 | +  M:2/2 | +  M:2/2 | +  M:2/2 | +  M:1/2 | -  M:0/2 | +  M:2/2 | -  M:0/2 | n/a | +  M:2/2 | -  M:0/2 | +  M:1/2 | +  M:2/2 | +  M:1/2 | -  M:0/2 | (b12c) |
| **Marinoscillum**  **(cyt)** | -  G:0/1 | +  G:1/1 | -  G:0/1 | +  G:1/1 | -  G:0/1 | -  G:0/1 | -  G:0/1 | -  G:0/1 | n/a | +  G:1/1 | -  G:0/1 | -  G:0/1 | -  G:0/1 | -  G:0/1 | -  G:0/1 | (b13) |
| **Planctomycetes group A**  **(pa1)** | +  G:2/2 | -  G:0/2 | -  G:0/2 | +  G:2/2 | +  G:2/2 | +  G:2/2 | -  G:0/2 | +  G:2/3 | n/a | +  G:2/2 | +  G:1/2 | +  G:1/2 | -  G:0/2 | +  G:1/2 | +  G:1/2 | (b14) |
| **Pirellula**  **(pir)** | -  G:0/2 | -  G:0/2 | +  G:1/2 | +  G:2/2 | +  G:1/2 | +  G:1/2 | -  G:0/2 | +  G:1/2 | +  P:1/1 | +  G:2/2 | +  G:1/2 | +  G:1/2 | -  G:0/2 | +  G:1/2 | +  G:1/2 | (b16) |
| **Euryarchaeota marine group II**  **(eur)** | -  M:0/8 | -  M:0/8 | -  M:0/8 | -  M:0/8 | -  M:0/8 | -  M:0/8 | -  M:0/8 | -  M:0/8 | -  M:0/8 | -  M:0/8 | -  M:0/8 | -  M:0/8 | -  M:0/8 | -  M:0/8 | -  M:0/8 | (b17) |
| **Betaproteobacteria**  **(bet)** | -  M:0/3 | -  M:0/3 | -  M:0/3 | -  M:0/3 | -  M:0/3 | -  M:0/3 | -  M:0/3 | -  M:0/3 | -  M:0/3 | -  M:0/3 | -  M:0/3 | -  M:0/3 | -  M:0/3 | -  M:0/3 | -  M:0/3 | (b18) |
| **SAR 324 clade**  **(s32)** | -  M:0/6 | -  M:0/6 | -  M:0/6 | -  M:0/6 | -  M:0/6 | -  M:0/6 | -  M:0/6 | -  M:0/6 | -  M:0/6 | -  M:0/6 | -  M:0/6 | -  M:0/6 | -  M:0/6 | -  M:0/6 | -  M:0/6 | (b19) |
| **Actinobacteria**  **(hgc)** | +  G:2/2 | n/a | n/a | +  G:1/1 | +  G:1/1 | +  G:1/1 | n/a | n/a | n/a | +  G:1/1 | n/a | n/a | n/a | n/a | n/a | (b20) |

(a) For bacteria not listed, no information was found. n/a indicates no information available. +/- indicates bacteria can/cannot utilize this substrate. Also shown is the type of evidence, including G: genomic, M: meta-genomic, R: meta-proteomic, P: physiological, and the corresponding positive / total strains. The determination was based on a literature survey and the Carbohydrate Active Enzyme Database ([www.cazy.org](http://www.cazy.org)) ([60](#_ENREF_60)). For genomic studies and the cazy.org database, strains were rated as utilization positive if more than half of the required key enzymes for the degradation of a specific polysaccharide are present. If studies on different strains of the same bacterial species and/or different methods of proofs were available, we performed weight of evidence calculations, with the formula:

$ꭕ=\frac{\left( Fpos1\times Wst1\times Wp1 \right)+\left( Fpos2\times Wst2\times Wp2 \right)+\left( Fposi\times Wsti\times Wpi \right)+ \ldots}{\left( Wst1\times Wp1 \right)+\left( Wst2\times Wp2 \right)+\left( Wsti\times Wpi \right)+ \ldots}$

where $Fpos1$ is the fraction of positive tested strains with the method of proof 1, $Wst1$is the number of strains tested with method of proof 1 (i.e. the number of tested strains corresponds to the factor of weight), and $Wp1$is the weight factor for the method of proof 1. We weighted meta-proteomic studies with the factor 1, meta-genomic studies with the factor 1.5, genomic studies with the factor 2, and physiological studies (direct evidence) with the factor 2.5. If ꭕ was ≥ 0.5 we defined the utilization as positive, for ꭕ < 0.5 we defined it as negative.

Additional constraints apply to model hypothetical DOM species corresponding to observations by [Sperling et al. (16)](#_ENREF_16). Specifically, if a bacteria is negative for all polysaccharides with major fraction corresponding to a DOM species (e.g. glc for gly), then it is also negative for that DOM species. For example, rox is negative for chr and cel, but positive for gly and sta, so it is positive for glc. However, it is negative for xyl, man and glo, so it is negative for max.

(b1) [Lombard et al. (60)](#_ENREF_60), genome of *Candidatus Pelagibacter ubique* HTCC1062.

(b2) [Lombard et al. (60)](#_ENREF_60), genome of the type species *Roseobacter litoralis* Och 149.

(b3) [Lombard et al. (60)](#_ENREF_60), [Klindworth et al. (61)](#_ENREF_61), [Hahnke et al. (62)](#_ENREF_62), [Avcı et al. (63)](#_ENREF_63).

(b4) [Lombard et al. (60)](#_ENREF_60), genome of *Pseudoalteromonas* sp. 13-15, isolated at Kabeltonne Helgoland.

(b5) [Lombard et al. (60)](#_ENREF_60), [Neumann et al. (64)](#_ENREF_64), [Koch et al. (65)](#_ENREF_65), genome of the type species *Alteromonas macleodii* ATCC 27126.

(b6) ([60](#_ENREF_60)), genome of the type species *Vibrio alginolyticus* ATCC 17749.

(b7) [Dupont et al. (66)](#_ENREF_66), [Hoarfrost et al. (67)](#_ENREF_67).

(b8) [Lombard et al. (60)](#_ENREF_60), [Tang et al. (68)](#_ENREF_68), [Klippel et al. (69)](#_ENREF_69), [Yin et al. (70)](#_ENREF_70), strain *Glaciecola* *nitratireducens* FR1064T.

(b9) [Lombard et al. (60)](#_ENREF_60), [Xing et al. (71)](#_ENREF_71), [Kappelmann et al. (72)](#_ENREF_72), [Avcı et al. (73)](#_ENREF_73), all available genomes from Helgoland.

(b10) [Lombard et al. (60)](#_ENREF_60), all available genomes from Helgoland.

(b11) [Lombard et al. (60)](#_ENREF_60), genome of *Ulvibacter* sp. SCB49.

(b12) [Krüger et al. (74)](#_ENREF_74).

(b12b) [Teeling et al. (14)](#_ENREF_14), [Krüger et al. (74)](#_ENREF_74).

(b12c) [Teeling et al. (14)](#_ENREF_14), [Krüger et al. (74)](#_ENREF_74). nde and nvi lumped, since clade not specified in references.

(b13) [Lombard et al. (60)](#_ENREF_60), strain *Marivirga tractuosa* DSM 4126.

(b14) [Lombard et al. (60)](#_ENREF_60), [Glöckner et al. (75)](#_ENREF_75), [Wecker et al. (76)](#_ENREF_76), genomes from *Planctomyces* sp. SH-PL14 and *Planctomyces* sp. SH-PL62.

(b16) [Lombard et al. (60)](#_ENREF_60), [Glöckner et al. (75)](#_ENREF_75), genomes from *Pirellula staleyi* DSM 6068 and *Rhodopirellula baltica* SH 1.

(b17) [Orellana et al. (77)](#_ENREF_77).

(b18) [Gifford et al. (78)](#_ENREF_78), [Yilmaz et al. (79)](#_ENREF_79).

(b19) Probably mixotroph, only short chain hydrocarbon utilization [Sheik et al. (80)](#_ENREF_80), [Cao et al. (81)](#_ENREF_81), [Haroon et al. (82)](#_ENREF_82).

(b20) [Manivasagan et al. (83)](#_ENREF_83).

1. Process parameters: Viral [v]

| **Symbol** | **Units** | **Description & notes (a)** |
| --- | --- | --- |
| *kv_j_* | L/mmolC/d | Contact rate for component *j*  *Literature:* 1.5 – 1.5e3, range used in model, [Weitz et al. (8)](#_ENREF_8) (b).  *Application:* not used |
| *betav* | - | Burst size  *Literature:* 0.0016 – 1, range used in model, [Weitz et al. (8)](#_ENREF_8) (c)  *Application:* not used |

(a) See footnote in Table S3.

(b) Based on *φ_V*_* = 1e-13 – 1e-10 L/virus/d, converted using 1e-11 μmolN/virus from [Weitz et al. (8)](#_ENREF_8) and 0.15 molN/molC (Redfield).

(c) Lower bound based on *β_H_* = 12.5 viruses, small virus with 0.5e-12 μmolN/virus and large heterotrophic host with 4e-9 μmolN/cell from [Weitz et al. (8)](#_ENREF_8). Upper bound based on mass balance.

1. Process parameters: Exudation [e]

| **Symbol** | **Units** | **Description & notes (a)** |
| --- | --- | --- |
| *ke* | 1/d | Basal exudation rate  *Literature:*  Phytoplankton:  0.005 – 0.1, range used in model, [Weitz et al. (8)](#_ENREF_8) (d).  Bacteria:  0.005 – 0.1, range used in model, [Weitz et al. (8)](#_ENREF_8) (d).  *Application:*  Phytoplankton:  DOM: (0.005, 0.1).  Inhibitors: (5.0e-11, 5.0e-9)  Bacteria:  Micronutrients: 1.0e-9  Inhibitors: 5e-11 (b) |
| *ef* | - | Fraction of photosynthesis or heterotrophy exudated  *Literature:*  Phytoplankton:  **0** − **0.70**, range of literature values, [Connolly et al. (45)](#_ENREF_45) (d).  0.13, literature summary, [Baines and Pace (84)](#_ENREF_84) (d).  Bacteria:  0.65 – 0.75, range summarized by [Mentges et al. (51)](#_ENREF_51) (c) (d).  *Application:*  Phytoplankton:  DOM: (0, 0.4)  Inhibitors: (1.0e-9, 1.0e-7)  Bacteria:  Micronutrients: 5.0e-9  Inhibitors: 1.0e-9 (b) |
| *Fe_j_* | - | Fraction to component *j*  *Application:* (0, 1)** |

(a) See footnote in Table S3.

(b) See footnote (e) in Table S8.

(c) Converted using BGE = 0.20.

(d) Literature values are given as rates (1/d) or fraction of production. The model here uses a combination.

1. Exudation of carbohydrates by phytoplankton (a)

| **Polysaccharide/**  **Phytoplankton**  **(ID)** | **arabinose**  **(ar2)** | **glucose**  **(gl2)** | **galactose**  **(ga2)** | **fucose**  **(fu2)** | **rhamnose**  **(rh2)** | **mannose**  **(ma2)** | **xylose**  **(ma2)** | **galacturonic acid**  **(ga3)** | **gluconic acid**  **(gl3)** | **glucuronic acid**  **(gc2)** | **muramic acid**  **(mu2)** | **galactosamine**  **(ga4)** | **glucosamine**  **(gl4)** | **guluronic acid**  **(n/a)** | **mannuronic acid**  **(n/a)** | **Note** |
| --- | --- | --- | --- | --- | --- | --- | --- | --- | --- | --- | --- | --- | --- | --- | --- | --- |
| **Diatoms**  **(dip, dix, mhe,**  **cde, cmi, rst, tno)** | 1.7 | 3 | 11.8 | 21.4 | 14.9 | 9.2 | 3.1 | 0 | 0 | 6.3 | 0 | 0 | 0 | 0 | 0 | ([85-87](#_ENREF_85)) |
| **Green algae**  **(gre)** | 1.3 | 3.1 | 10.9 | 10.8 | 11.9 | 14.7 | 3.2 | 0 | 0 | 0 | 0 | 0 | 0 | 0 | 0 | ([88](#_ENREF_88), [89](#_ENREF_89)) |
| **Dinoflagellates**  **(dxf, din)** | 0.3 | 22.3 | 30.5 | 2.4 | 1.8 | 4.1 | 3.9 | 0 | 0 | 0 | 0 | 0 | 0 | 0 | 0 | ([90](#_ENREF_90), [91](#_ENREF_91)) |
| **Silicoflagellates**  **(crysophyta)**  **(sif)** | n/a | n/a | n/a | n/a | n/a | n/a | n/a | n/a | n/a | n/a | n/a | n/a | n/a | n/a | n/a |  |
| **Coccolithophorids**  **(coc)** | 9.8 | 13.7 | 14.6 | 4.2 | 4.3 | 11.8 | 8.6 | 0 | 0 | 1.8 | 0 | 0 | 0 | 0 | 0 | ([86](#_ENREF_86), [92](#_ENREF_92)) |
| **Ciliates**  **(n/a)** | n/a | n/a | n/a | n/a | n/a | n/a | n/a | n/a | n/a | n/a | n/a | n/a | n/a | n/a | n/a |  |
| **Phaeocystis**  **(pha)** | 11.9 | 2.0 | 11.2 | 1.4 | 14.7 | 21 | 4.2 | 0 | 0 | 0 | 0 | 0 | 0 | 0 | 0 | ([86](#_ENREF_86)) |
| **Chattonella**  **(cha)** | n/a | n/a | n/a | n/a | n/a | n/a | n/a | n/a | n/a | n/a | n/a | n/a | n/a | n/a | n/a |  |

(a) Values are percentages of total carbon exudation. Carbohydrate exudation was assumed as 70% of the overall carbon exudation ([85](#_ENREF_85), [89](#_ENREF_89)). n/a indicates no information available. If shares for poly- or mono-saccharides differed between species (in the same group) or studies, each species (study) was treated equal and a general mean was calculated.

1. Process parameters: Death [y]

| **Symbol** | **Units** | **Description & notes (a)** |
| --- | --- | --- |
| *kybase* | 1/d | Base rate constant  *Literature:*  Bacteria: 0.009 – 0.025, range summarized by [Mentges et al. (51)](#_ENREF_51).  *Application:* not used |
| *Cref* | - | See Table S3. |
| *xny* | - | Exponent on population term  *Literature*  2, model value ([93](#_ENREF_93))  *Application:* not used |

(a) See footnote in Table S3.

1. Process parameters: Loss [u]

| **Symbol** | **Units** | **Description & notes (a)** |
| --- | --- | --- |
| *ku* | 1/d | Max. rate constant  *Literature:*  Phytoplankton grazing:  0.06 - 0.4, model value and literature summary ([1](#_ENREF_1)).  0.2 - 0.5, model value and literature summary ([94](#_ENREF_94)).  *Application:*  Phytoplankton: 0.5  Bacteria: 0.5 |
| *Toptu* | °C | Optimum temperature  *Literature:* 1 – 19, range at Helgoland.  *Application:* not used |
| *beta1Tu* | - | Temperature limitation exponent 1  *Application:* not used |
| *beta2Tu* | - | Temperature limitation exponent 2  *Application:* not used |
| *TYopt1u* | d | Optimum TY 1  *Literature:* **-**  *Application:* 355 |
| *TYopt2u* | d | Optimum TY 2  *Literature:* **-**  *Application:* 5 |
| *beta1TYu* | - | TY limitation exponent 1  *Application:* (0.00015, 0.015) (b) |
| *beta2TYu* | - | TY limitation exponent 2  *Application:* (0.00015, 0.015) (b) |
| *E1optu* | PSU | Optimum salinity  *Literature:*  28 – 35, salinity range at Helgoland.  *Application:* (28, 35) |
| *beta1E1u* | - | Salinity limitation exponent 1  *Application:* (0, 1) (F) |
| *beta2E1u* | - | Salinity limitation exponent 2  *Application:* (0, 1) (F) |
| *Ioptu* | μE/m^2^/s | Optimum light intensity  *Literature:*  30 – 350, light intensity range at Helgoland, est. from model.  *Application:* (30, 350) |
| *beta1Iu* | - | Light limitation exponent 1  *Application:* (0, 0.0005) (F) |
| *beta2Iu* | - | Light limitation exponent 2  *Application:* (0, 0.0005) (F) |

(a) See footnote in Table S3.

(b) Corresponds to increase/decrease of grazer population over 7 days at a rate of 0.001 and 0.1 1/d.

(F) See footnote in Table S8.

1. Process parameters: Inhibition [i]

| **Symbol** | **Units** | **Description & notes (a)** |
| --- | --- | --- |
| *ki* | 1/d | Max. inhibition rate  *Application:* (0, 1) |
| *Ksi_j_* | μmolC/L | Half-saturation constant for component *j*  *Application:* (1.0e-14, 1.0e-9) |

(a) See footnote in Table S3.

1. Global parameters

| **Symbol** | **Units** | **Description & notes** |
| --- | --- | --- |
| *T* | °C | Temperature  Time-variable input  *Application:* Based on observations |
| *Q* | m^3^/d | Flow rate  Time-variable input  *Literature:* 0.53, corresponds to 2 month residence time on German Bight ([95](#_ENREF_95))  *Application:* 0.26 |
| *V* | m^3^ | Volume  Constant input  *Application:* 1 m^2^ × H |
| *IT* | μE/m^2^/s | Irradiance  Time-variable input  *Application:* MODIS PAR. |
| *f* | - | Photoperiod  Time-variable input  *Application:* From [www.timeanddate.com](http://www.timeanddate.com) |
| *H* | m | Depth  Constant input  *Application:* 8, average water column depth at Helgoland ([14](#_ENREF_14)) |

1. Observations: Nutrients, phytoplankton and zooplankton

| **Observed parameter** | **ID** | **Units** | **Weight(a)** |
| --- | --- | --- | --- |
| NOX | nox | μmolN/L | 3 |
| Ammonium | nh4 | μmolN/L | 3 |
| Phosphate | po4 | μmolP/L | 3 |
| Silicate | sil | μmolSi/L | 3 |
| Chlorophyll *a* (BBE) | chl | μgChl*a*/L | 10 |
| Light extinction coefficient (b) | kex | 1/m | 3 |
| Total | tot | 10^6^/L | 0 (c) |
| Diatoms | dia | 10^6^/L | 1 |
| Diatoms – Pennales | dip | 10^6^/L | 1 |
| Diatoms – Centrales | dic | 10^6^/L | 1 |
| Greenalgae | gre | 10^6^/L | 1 |
| Dinoflagellates | dif | 10^6^/L | 1 |
| Silicoflagellates | sif | 10^6^/L | 1 |
| Coccolithophorids | coc | 10^6^/L | 1 |
| Flagellates | fla | 10^6^/L | 0 (c) |
| Ciliates | cil | 10^6^/L | 0 (c) |
| *Mediopyxis helysia* | mhe | 10^6^/L | 1 |
| *Chaetoceros debilis* | cde | 10^6^/L | 1 |
| *Chaetoceros minimus* | cmi | 10^6^/L | 1 |
| *Rhizosolenia styliformis* | rst | 10^6^/L | 1 |
| *Thalassiosira nordenskioeldii* | tno | 10^6^/L | 1 |
| Dinophyceae | din | 10^6^/L | 1 |
| Phaeocystis | pha | 10^6^/L | 1 |
| Chattonella | cha | 10^6^/L | 1 |

(a) See Section S2.2.

(b) Observations converted from Secchi Disk (SD) using kext = 1.8 / SD ([15](#_ENREF_15)).

(c) Excluded, includes more than just phytoplankton.

1. Observations: Bacteria

| **Observed parameter** | **ID** | **Units** | **Weight(a)** |
| --- | --- | --- | --- |
| Total bacterioplankton by DAPI | dap | 1e6/mL | 10 |
| NON338 | non | 1e6/mL | 0 |
| EUB338-I-III (Eubacteria) | eub | 1e6/mL | 1 |
| ALF968 (Alphaproteobacteria) | alf | 1e6/mL | 1 |
| SAR11-486 (SAR11 clade Alphaproteobacteria) | s48 | 1e6/mL | 1/4 (b) |
| SAR11-441 (SAR11 clade Alphaproteobacteria) | s44 | 1e6/mL | 3/4 (b) |
| ROS537 (Roseobacter clade Alphaproteobacteria) | ros | 1e6/mL | 1 |
| NAC11-7-1030 (Nac 11 clade Roseobacter) | nac | 1e6/mL | 1 |
| RCA1000 (RCA and DC5 clade Roseobacter) | rca | 1e6/mL | 1 |
| GAM42a (Gammaproteobacteria) | gam | 1e6/mL | 1 |
| REI731 (Reinekea genus, Gammaproteobacteria) | rei | 1e6/mL | 1 |
| Bal731 (Balneatrix genus, Gammaproteobacteria) | bal | 1e6/mL | 1 |
| OM182-707 (OM182 clade of Gammaproteobacteria) | om1 | 1e6/mL | 1 |
| NOR5-730 (NOR5 clade of Gammaproteobacteria) | nor | 1e6/mL | 1 |
| PSA184 (Pseudoalteromonas Gammaproteobacteria) | psa | 1e6/mL | 1 |
| ALT1413 (Alteromonas Gammaproteobacteria) | alt | 1e6/mL | 1 |
| GV841 (Vibrio Gammaproteobacteria) | gv8 | 1e6/mL | 1 |
| SAR92-627 (SAR92 clade Gammaproteobacteria) | s92 | 1e6/mL | 1 |
| SAR86-1245 (SAR86 clade Gammaproteobacteria) | s86 | 1e6/mL | 1 |
| Glac227 (Glaciecola Gammaproteobacteria) | gla | 1e6/mL | 1 |
| CF319a (Bacteroidetes) | cf3 | 1e6/mL | 1 |
| POL740 (Polaribacter genus Bacteroidetes) | pol | 1e6/mL | 1 |
| FORM181A (Formosa genus Bacteroidetes) | foa | 1e6/mL | 1 |
| FORM181B (Formosa sp. Hel1_33_131 Bacteroidetes) | fob | 1e6/mL | 1 |
| ULV995 (Ulvibacter related clade I Bacteroidetes) | ulv | 1e6/mL | 1 |
| VIS6-814 (VIS6 clade Cryomorphaceae Bacteroidetes) | vis | 1e6/mL | 1 |
| NS3a-840 (NS3a marine group Bacteroidetes) | ns3 | 1e6/mL | 1 |
| NS5/DE2-471 (NS5/DE2 clade Bacteroidetes) | nde | 1e6/mL | 1 |
| NS5/VIS1-575 (VIS1 genus in NS5 marine group Bacteroidetes) | nvi | 1e6/mL | 1 |
| NS9-664 (NS9 marine group Bacteroidetes) | ns9 | 1e6/mL | 1 |
| CYT-734 (Marinoscillum Bacteroidetes) | cyt | 1e6/mL | 1 |
| PLA46 (Planctomycetes) | pa4 | 1e6/mL | 1 |
| PirD1039 (Pirellula genus Planctomycetes) | pir | 1e6/mL | 1 |
| uPlaB440 (Planctomycetes group B) | upl | 1e6/mL | 1 |
| PlaA1228 (Planctomycetes group A) | pa1 | 1e6/mL | 1 |
| ARCH915 (Archaea) | arc | 1e6/mL | 1 |
| EURY806 (Euryarcheota marine group II) | eur | 1e6/mL | 1 |
| CREN554 (Crenarcheota marine group I) | cre | 1e6/mL | 1 |
| BET42a (Betaproteobacteria) | bet | 1e6/mL | 1 |
| SAR324-1412 (SAR 324 clade Deltaproteobacteria) | s32 | 1e6/mL | 1 |
| HGC69a (Actinobacteria) | hgc | 1e6/mL | 1 |

(a) See footnote in Table S22.

(b) Based on use of probes for SAR11, SAR11-486 in year 1, SAR11-441 in years 2-4.

Note: For NON338+, observed concentrations calculated from DAPI (1e6/mL) and %.

1. Observations: POM and DOM

| **Observed parameter** | **ID** | **Units** | **Weight (a)** | **Source (b)** |
| --- | --- | --- | --- | --- |
| POC | poc | mmolC/L | 3 | S17 |
| DOC | doc | mmolC/L | 3 | S17 |
| Ara (arabinose) | ara | mmolC/L | 1 | S17 |
| Fuc (fucose) | fuc | mmolC/L | 1 | S17 |
| Gal (galactose) | gal | mmolC/L | 1 | S17 |
| Glc (glucose) | glc | mmolC/L | 1 | S17 |
| Man/Xyl (mannose/xylose) | max | mmolC/L | 1 | S17 |
| Rha (rhamnose) | rha | mmolC/L | 1 | S17 |
| GalUA (galacturonic acid) | gau | mmolC/L | 1 | S17 |
| GlcUA (gluconic acid) | glu | mmolC/L | 1 | S17 |
| GlcA (glucuronic acid) | gca | mmolC/L | 1 | S17 |
| Mur (muramic acid) | mur | mmolC/L | 1 | S17 |
| GalN (galactosamine) | gan | mmolC/L | 1 | S17 |
| GlcN (glucosamine) | gln | mmolC/L | 1 | S17 |
| POM chrysolaminarin | lam | mmolC/L | 3 | B17 |

(a) See footnote in Table S22.

(b) S17 = [Sperling et al. (16)](#_ENREF_16), B17 = [Becker et al. (23)](#_ENREF_23).

1. Model species: Nutrients and phytoplankton

| **Model**  **Species**  **(a)** | **Optimization target(s) (b)** | **MC**  **pmolC/cell** |
| --- | --- | --- |
| nox | **nox** | - |
| nh4 | **nh4** | - |
| po4 | **po4** | - |
| sil | **sil** | - |
| gre | **gre**, chl, kex, poc, lam (g) | 100 (c) |
| mhe | **mhe**, dic, dia, chl, kex, poc, lam (g) | 230 (d) (e) |
| cde | **cde**, dic, dia, chl, kex, poc, lam (g) | 18 (f) (e) |
| cmi | **cmi**, dic, dia, chl, kex, poc, lam (g) | 2.1 (f) (e) |
| rst | **rst**, dic, dia, chl, kex, poc, lam (g) | 630 (f) (e) |
| tno | **tno**, dic, dia, chl, kex, poc, lam (g) | 37 (f) (e) |
| dix(MH) | dic, dia, chl, kex, poc, lam (g) | 37 (h) (e) |
| dip | **dip**, dia, chl, kex, poc, lam (g) | 37 (h) (e) |
| din | **din**, dif, chl, kex, poc, lam (g) | 62 (j) |
| dxf(MH) | dif, chl, kex, poc, lam (g) | = din |
| cha | **cha**, chl, kex, poc, lam (g) | 21 (f) (e) |
| sif | **sif**, chl, kex, poc, lam (g) | 31 (i) |
| pha | **pha**, chl, kex, poc, lam (g) | 2.2 (j) |
| coc | **coc**, chl, kex, poc, lam (g) | 0.97 (k) |
| ph1(MH) | chl, kex, poc, lam (g) | 100 (c) |

(a) “(MH)” denotes model hypothetical species (i.e. no 1:1 observations).

(b) **Bold** denotes 1:1 model:observation.

(c) Reasonable value for phytoplankton, Table S3.

(d) From [Loebl et al. (96)](#_ENREF_96).

(e) Converted based on [Menden-Deuer and Lessard (97)](#_ENREF_97).

(f) From average cell volume in [Olenina (98)](#_ENREF_98).

(g) See footnote (g) in Table S24.

(h) From median cell volume in [Harrison et al. (99)](#_ENREF_99).

(i) From [Takahashi (100)](#_ENREF_100).

(j) From <http://nordicmicroalgae.org/>. Range: *Phaeocystis*: 17 – 41, *Dinophyceae*: 77 – 7,200 pgC/cell.

(k) From [Montagnes et al. (101)](#_ENREF_101).

1. Model species: Bacteria

| **Model**  **species**  **(a)** | **Optimization target(s) (b)** | **Oligo. (e)** | **MC (c) (d)**  **fmolC/cell** | **Notes** |
| --- | --- | --- | --- | --- |
| eux (MH) | eub, dap, poc | 0.29 (e1) | 8.4 | (d1) |
| alx (MH) | alf, eub, dap, poc | 0.63 (e1) | = eux | - |
| s11 | **s48**, **s44**, alf, eub, dap, poc | 1 ([102](#_ENREF_102)) (e2) | 2.6 | ([103](#_ENREF_103)) (d2) |
| rox (MH) | ros, alf, eub, dap, poc | 0.5 (e4) | = rca | - |
| nac | **nac**, ros, alf, eub, dap, poc | 0.5 (e4) | = rca | - |
| rca | **rca**, ros, alf, eub, dap, poc | 0.5 (e4) | 14 | ([104](#_ENREF_104)) (d2) |
| gax (MH) | gam, eub, dap, poc | 0.25 ([105](#_ENREF_105), [106](#_ENREF_106)) (e2) | = eux | - |
| rei | **rei**, gam, eub, dap, poc | 0 ([63](#_ENREF_63)) (e2) | 10 | ([62](#_ENREF_62)) (d2) |
| bal | **bal**, gam, eub, dap, poc | 0 ([107](#_ENREF_107)) (e2) | 13 | ([108](#_ENREF_108)) (d2) |
| om1 | **om1**, gam, eub, dap, poc | 1 ([109](#_ENREF_109)) (e2) | 4.1 | ([109](#_ENREF_109)) (d2) |
| nor | **nor**, gam, eub, dap, poc | 1 ([110](#_ENREF_110)) (e2) | 7.7 | ([111](#_ENREF_111)) (d2) |
| psa | **psa**, gam, eub, dap, poc | 0 ([112](#_ENREF_112)) (e2) | 6.3 | ([113](#_ENREF_113)) (d2) |
| alt | **alt**, gam, eub, dap, poc | 0 ([114](#_ENREF_114)) (e2) | 6.9 | ([113](#_ENREF_113)) (d2) |
| gv8 | **gv8**, gam, eub, dap, poc | 0 ([115](#_ENREF_115)) (e2) | 7.1 | ([113](#_ENREF_113)) (d2) |
| s92 | **s92**, gam, eub, dap, poc | 1 ([102](#_ENREF_102)) (e2) | 4.1 | ([109](#_ENREF_109)) (d2) |
| s86 | **s86**, gam, eub, dap, poc | 1 ([66](#_ENREF_66)) (e2) | = eux | - |
| gla | **gla**, gam, eub, dap, poc | 0 ([114](#_ENREF_114)) (e2) | 5.7 | ([116](#_ENREF_116)) (d2) |
| cfx (MH) | cf3, eub, dap, poc | 0 ([105](#_ENREF_105)) (e2) | = eux | - |
| pol | **pol**, cf3, eub, dap, poc | 0 ([102](#_ENREF_102)) (e2) | 9.5 | ([62](#_ENREF_62)) (d2) |
| fox (MH) | foa, cf3, eub, dap, poc | 0 ([117](#_ENREF_117)) (e2) | 7.9 | ([62](#_ENREF_62)) (d2) |
| fob | **fob**, foa, cf3, eub, dap, poc | 0 ([117](#_ENREF_117)) (e2) | = fox | - |
| ulv | **ulv**, cf3, eub, dap, poc | 0 ([118](#_ENREF_118)) (e2) | 7.4 | ([119](#_ENREF_119)) (d2) |
| vis | **vis**, cf3, eub, dap, poc | 0 ([74](#_ENREF_74)) (e2) | 6.5 | ([116](#_ENREF_116)) (d2) |
| ns3 | **ns3**, cf3, eub, dap, poc | 0 ([118](#_ENREF_118)) (e2) | = eux | - |
| nde | **nde**, cf3, eub, dap, poc | 0 ([118](#_ENREF_118)) (e2) | = eux | - |
| nvi | **nvi**, cf3, eub, dap, poc | 0 ([118](#_ENREF_118)) (e2) | = eux | - |
| ns9 | **ns9**, cf3, eub, dap, poc | 0 ([118](#_ENREF_118)) (e2) | = eux | - |
| cyt | **cyt**, cf3, eub, dap, poc | 0 ([120](#_ENREF_120)) (e2) | = eux | - |
| px4 (MH) | pa4, eub, dap, poc | 0.5 ([121](#_ENREF_121)) (e2) | 10 | ([122](#_ENREF_122)) (d2) |
| pir | **pir**, pa4, eub, dap, poc | 0 ([123](#_ENREF_123)) (e2) | 11 | ([124](#_ENREF_124)) (d2) |
| upl | **upl**, pa4, eub, dap, poc | 0.5 ([121](#_ENREF_121)) (e2) | 10 | ([122](#_ENREF_122)) (d2) |
| pa1 | **pa1**, pa4, eub, dap, poc | 0.5 ([121](#_ENREF_121)) (e2) | 10 | ([122](#_ENREF_122)) (d2) |
| arx (MH) | arc, dap, poc | 0.5 (e3) | = eux | - |
| eur | **eur**, arc, dap, poc | 0.5 (e3) | = eux | - |
| cre | **cre**, arc, dap, poc | 0.5 (e3) | = eux | - |
| bet | **bet**, eub, dap, poc | 0 ([105](#_ENREF_105)) (e2) | = eux | - |
| s32 | **s32**, eub, dap, poc | 0.5 ([125](#_ENREF_125)) (e2) | = eux | - |
| hgc | **hgc**, eub, dap, poc | 0.5 ([105](#_ENREF_105)) (e2) | = eux | - |

(a-c) See footnotes in Table S22.

(e) Fraction oligotrophic, used for Fig. 4C in main paper.

(e1) Average of constrained values.

(e2) Based on reference provided.

(e3) No information found.

(e4) Based on [Kirchman (53)](#_ENREF_53), [Ho et al. (105)](#_ENREF_105), [Voget et al. (126)](#_ENREF_126), [Mayali et al. (127)](#_ENREF_127), [Giebel et al. (128)](#_ENREF_128).

(d1) Average of constrained values.

(d2) Conversion based on [Romanova and Sazhin (129)](#_ENREF_129).

1. Model species: POM and DOM

| **Model species (a)** | **Optimization target(s) (b) (d)** |
| --- | --- |
| *POM* |  |
| p01+, pra, puc, pal, plc, pax,  xph, pau, plu, pca, pur, pan, pln, phi,  phr, ply, pyl, pel, xpa, pta, xpe, plo,  pcs, phm (f) (all MH) | kex, poc, lam (g) |
| bsi (biogenic silica) | - |
| *DOM* |  |
| d01+ (MH) | doc |
| *DOM/Polysaccharides* |  |
| ar2 (MH) | ara (1), doc |
| fu2 (MH) | fuc (1), doc |
| ga2 (MH) | gal (1), doc |
| gl2 (MH) | glc (1), doc |
| ma2 (MH) | max (1), doc |
| rh2 (MH) | rha (1), doc |
| ga3 (MH) | gau (1), doc |
| gl3 (MH) | glu (1), doc |
| gc2 (MH) | gca (1), doc |
| mu2 (MH) | mur (1), doc |
| ga4 (MH) | gan (1), doc |
| gl4 (MH) | gln (1), doc |
| chi (chitin) | gln (1), doc (e) |
| chr (chrysolaminarin) | glc (1), doc (e) |
| gly (glycogen) | glc (1), doc (e) |
| xyl (xylan) | max (1), doc (e) |
| cel (cellulose) | glc (1), doc (e) |
| man (mannan) | gal (0.05), max (0.95), doc (e) |
| sta (starch) | glc (1), doc (e) |
| pec (pectin) | gau (1), doc (e) |
| glo (glucoromannan) | glc (0.38), max (0.62), doc (e) |
| fcs (FCSP) | glc (0.05), fuc (0.80), rha (0.05), max (0.10), doc (e) |
| rhm (rhamnan) | rha (1), doc (e) |
| *DOM/micronutrients & inhibitors* |  |
| m01-m15 (MH) | doc |
| i01-i30 (MH) | doc |
| *Other solids* |  |
| nkx (inorganic suspended solids) | kex |

(a-c) See footnotes in Table S22.

(d) Value in parentheses indicates fractional contribution. e.g. 5% of man is gal.

(e) Polysaccharide monomer composition in Table S25.

(f) POM species correspond in order to DOM species (e.g. pra > ar2).

(g) lam = phr + mhe Fx(phr+chr) + other phytoplankton…

1. Monosaccharide shares of polysaccharides (a)

| **Monomer/**  **Polysaccharide**  **(ID)** | **arabinose**  **(ara)** | **glucose**  **(glc)** | **galactose**  **(gal)** | **fucose**  **(fuc)** | **rhamnose**  **(rha)** | **mannose/xylose**  **(max)** | **galacturonic acid**  **(gau)** | **gluconic acid**  **(glu)** | **glucuronic acid**  **(gca)** | **muramic acid**  **(mur)** | **galactosamine**  **(gan)** | **glucosamine**  **(gln)** | **guluronic acid**  **(n/a)** | **mannuronic acid**  **(n/a)** | **note** |
| --- | --- | --- | --- | --- | --- | --- | --- | --- | --- | --- | --- | --- | --- | --- | --- |
| **chitin**  **(chi)** |  |  |  |  |  |  |  |  |  |  |  | 1 |  |  |  |
| **chrysolaminarin**  **(chr)** |  | 1 |  |  |  |  |  |  |  |  |  |  |  |  |  |
| **alginate**  **(n/a)** |  |  |  |  |  |  |  |  |  |  |  |  | 0.45 | 0.55 | ([130](#_ENREF_130)) |
| **glycogen**  **(gly)** |  | 1 |  |  |  |  |  |  |  |  |  |  |  |  |  |
| **xylan**  **(xyl)** |  |  |  |  |  | 1 |  |  |  |  |  |  |  |  |  |
| **cellulose**  **(cell)** |  | 1 |  |  |  |  |  |  |  |  |  |  |  |  |  |
| **mannan**  **(man)** |  |  | 0.05 |  |  | 0.95 |  |  |  |  |  |  |  |  | ([131](#_ENREF_131)) |
| **carrageenan**  **(n/a)** |  |  | 1 |  |  |  |  |  |  |  |  |  |  |  |  |
| **chondroitin sulfate**  **(n/a)** |  |  |  |  |  |  |  |  | 0.5 |  | 0.5 |  |  |  |  |
| **starch**  **(sta)** |  | 1 |  |  |  |  |  |  |  |  |  |  |  |  |  |
| **pectin**  **(pec)** |  |  |  |  |  |  | 1 |  |  |  |  |  |  |  |  |
| **fucoidan**  **(n/a)** |  |  |  | 1 |  |  |  |  |  |  |  |  |  |  |  |
| **glucoromannan**  **(glo)** |  | 0.38 |  |  |  | 0.62 |  |  |  |  |  |  |  |  | ([132](#_ENREF_132)) |
| **FCSP**  **(fcs)** |  | 0.05 |  | 0.8 | 0.05 | 0.1 |  |  |  |  |  |  |  |  | ([133](#_ENREF_133)) |
| **rhamnan**  **(rhm)** |  |  |  |  | 1 |  |  |  |  |  |  |  |  |  |  |

(a) Values are fractions.

1. Uncertainty (CV, coefficient of variation) of selected model output and parameters (a)

| **Parameter** | **CV (b)** | | | |
| --- | --- | --- | --- | --- |
| Concentrations | Integrated |  | Instantaneous |  |
| All phytoplankton | 1.00 |  | 1.11 |  |
| *rst* in 2009 | 0.09 |  | 0.53 |  |
| All DOM | 0.38 |  | 0.40 |  |
| *d11* in 2009 | 0.17 |  | 0.09 |  |
| *gl2* in 2009 | 0.54 |  | 0.61 |  |
| *chr* in 2009 | 0.10 |  | 0.23 |  |
| All bacteria | 0.46 |  | 0.50 |  |
| *pol* in 2009 | 0.33 |  | 0.34 |  |
|  | Integrated | | Instantaneous | |
| Fluxes | Aggregated | Individual | Aggregated | Individual |
| All phytoplankton > DOM (Xa>d) | 0.99 | 1.45 | 1.16 | 1.55 |
| *rst* > *d11* |  | 2.04 |  | 2.60 |
| *rst* > *gl2* |  | 0.99 |  | 1.05 |
| *rst* > *chr* |  | 0.09 |  | 0.52 |
| All DOM > bacteria (Xd>b) | 0.52 | 0.96 | 0.61 | 1.01 |
| *d11* > *pol* |  | 0.75 |  | 0.65 |
| *gl2* > *pol* |  | 0.63 |  | 0.72 |
| *chr* > *pol* |  | 0.43 |  | 0.41 |
| All phytoplankton > bacteria (Xa>b) |  | 1.27 |  | 1.38 |
| *rst* > *pol* in 2009 |  | 0.42 |  | 0.76 |
| Parameters | Value |  |  |  |
| All *ke* | 0.43 |  |  |  |
| *ke_rst_* | 0.46 |  |  |  |
| All *ef* | 1.58 |  |  |  |
| *ef_rst_* | 2.15 |  |  |  |
| All *Fe* | 1.05 |  |  |  |
| *Fe_rst,d11_* | 1.09 |  |  |  |
| All *Fx* | 1.48 |  |  |  |
| *Fx_rst,d11_* | 2.78 |  |  |  |
| *Fx_rst,gl2_* | 1.00 |  |  |  |
| *Fx_rst,chr_* | 0 (specified) |  |  |  |
| All *kh* | 0.01 |  |  |  |
| *kh_pol_* | 0.01 |  |  |  |
| All *Ksh* | 1.01 |  |  |  |
| *Ksh_pol,d11_* | 0.05 |  |  |  |
| *Ksh_pol,gl2_* | 0.41 |  |  |  |
| *Ksh_pol,chr_* | 0.20 |  |  |  |

(a) Values describing interaction of phytoplankton and bacteria via DOM were selected. Generally higher values are evident for quantities not constrained (e.g. *d11*), quantities less important (e.g. *d11*) and summary quantities that include less important ones.

(b) Integrated or Instantaneous refers to model output averaged over the bloom periods or at specific times. Aggregated or Individual refers to model output summed for each species or each specific interaction. For example, for phytoplankton > DOM interaction, the integrated/aggregated CV is the average CV of Xa>d for the bloom periods, summed across all DOM for each phytoplankton species (Dataset_S1_Integrated_2009.xlsx/sum, Dataset_S1_Integrated_2010.xlsx/sum, …). The instantaneous/individual CV is the average CV of Xa>d for the instantaneous time points, for each phytoplankton > DOM interaction (Dataset_S1_Instantaneous_2009_0.xlsx/Xa>d, Dataset_S1_Instantaneous_2009_7.xlsx/Xa>d, …).

# Description of Dataset S1.

File naming convention

Integrated Networks: Dataset_S1_Integrated_(Y).xlsx

Instantaneous Networks: Dataset_S1_Instantaneous_(Y)_(T).xlsx

Y = year, T = bloom stage (days)

Bloom definitions (start)

| **Year** | **Date** |
| --- | --- |
| 2009 | 19.03.2009 |
| 2010 | 28.03.2010 |
| 2011 | 16.04.2011 |
| 2012 | 29.03.2012 |

Tab naming convention

| **Tab** | **Content** |
| --- | --- |
| sum | Summary |
| Xa>d | For each phytoplankton, the C flux to each DOM (μmolC/L/d) |
| Xa>dR | For each phytoplankton, the C flux to each DOM, rank for DOM |
| fa>din | For each phytoplankton, the C flux to each DOM, fraction for DOM |
| fa>dout | For each phytoplankton, the C flux to each DOM, fraction for phytoplankton |
| Xa>de | For each phytoplankton, the C flux to each DOM via exudation (μmolC/L/d) |
| Xa>p | For each phytoplankton, the C flux to each POM (μmolC/L/d) |
| fa>pin | For each phytoplankton, the C flux to each POM, fraction for POM |
| fa>pout | For each phytoplankton, the C flux to each POM, fraction for phytoplankton |
| Xp>d | For each POM, the C flux to each DOM (μmolC/L/d) |
| fp>din | For each POM, the C flux to each DOM, fraction for DOM |
| Xb>d | For each bacteria, the C flux to each DOM (μmolC/L/d) |
| Xb>p | For each bacteria, the C flux to each POM (μmolC/L/d) |
| Xd>b | For each bacteria, the C flux from each DOM (μmolC/L/d) |
| Xd>bR | For each bacteria, the C flux from each DOM, rank for bacteria |
| fd>bin | For each bacteria, the C flux from each DOM, fraction for bacteria |
| Xa>b | For each bacteria, the C flux from each phytoplankton (μmolC/L/d) |
| Xa>bR | For each bacteria, the C flux from each phytoplankton, global rank based on flux |
| fa>bin | For each bacteria, the C flux from each phytoplankton, fraction for bacteria |
| fa>binR | For each bacteria, the C flux from each phytoplankton, global rank based on fraction |

Values for Bray-Curtis similarity in Fig. 6A:

Recurrence, FluxNet, phytoplankton producers: absolute flux (Xa>d)

Recurrence, FluxNet, DOM exchanged: absolute flux (average of Xa+p+b>d and Xd>b)

Recurrence, FluxNet, bacteria consumers: absolute flux (Xd>b)

Recurrence, FluxNet, absolute phytoplankton > bacteria: absolute flux (Xa>b)

Recurrence, FluxNet, relative phytoplankton > bacteria: fractions (fa>bin)

Recurrence, LSA, local similarity scores

Methodology, absolute phytoplankton > bacteria: normalized FluxNet flux (Xa>b) and normalized LSA local similarity score

Methodology, relative phytoplankton > bacteria: normalized FluxNet fraction (fa>bin) and normalized LSA local similarity score

Flux calculations

Note: These calculations only consider interactions between phytoplankton, DOM, POM and bacteria. Fluxes and fractions do not include outflow, settling, photosynthesis and respiration.

Examples for Spring 2009 bloom, 28 days (Dataset_S1_Instantaneous_2009_28.xlsx) shown in red font.

*Flux (X) (μmolC/L/d) from i to j*

$$X_{i>j}$$

Example: Flux from *rst* to chr+phr

$$X_{rst>chr+phr}=0.0539+0.0539=0.108$$

*Fraction (f) of DOM d produced by phytoplankton a, directly, by DOM*

$$f_{a>d,in}=\frac{X_{a>d}}{X_{d,in}}$$

*Fraction (f) of DOM d produced by phytoplankton a, directly, by phytoplankton*

$$f_{a>d,out}=\frac{X_{a>d}}{X_{a,out}}$$

Example: Fraction of chr+phr produced by *rst*, by *rst*

$$f_{rst>chr+phr,out}=\frac{X_{rst>chr+phr}}{X_{rst,out}}=\frac{0.108}{0.364}=0.296$$

*Fraction (f) of POM p produced by phytoplankton a, by POM*

$$f_{a>p,in}=\frac{X_{a>p}}{X_{p,in}}$$

Example: Fraction of phr produced by *rst*, by phr

$$f_{rst>phr,in}=\frac{X_{rst>phr}}{X_{phr,in}}=\frac{0.0539}{0.0573}=0.939$$

*Flux (X) from phytoplankton a to DOM d, total (T)*

$$X_{a>d,T}=X_{a>d}+\sum_{p} X_{p>d} f_{a>p,in}$$

Example: Flux from *rst* to chr, total

$$X_{rst>chr,T}=X_{rst>chr}+X_{phr>chr} f_{rst>phr,in}=0.0539+0.0451 0.939=0.096$$

Note: phr is the only POM source to chr

*Fraction (f) of DOM d produced by phytoplankton a, total (T), by DOM*

$$f_{a>d,in,T}=\frac{X_{a>d,T}}{X_{d,in}}$$

Example: fraction of chr produced by *rst*, total, by chr

$$f_{rst>chr,in,T}=\frac{X_{rst>chr,T}}{X_{chr,in}}=\frac{0.096}{0.102}=0.939$$

*Fraction (f) of DOM d consumed by bacteria b, by bacteria*

$$f_{d>b,in}=\frac{X_{d>b}}{X_{b,in}}$$

Example: Fraction of chr consumed by *pol*, by *pol*

$$f_{chr>pol,in}=\frac{X_{chr>pol}}{X_{pol,in}}=\frac{0.159}{0.459}=0.346$$

*Flux (X) from phytoplankton a to bacteria b via DOM d*

$$X_{a>b,d}=X_{d>b} f_{a>d,in,T}$$

Example: Flux from *rst* to *pol* via chr

$$X_{rst>pol,chr}=X_{chr>pol} f_{rst>chr,in,T}=0.159 0.939=0.149$$

*Flux (X) from phytoplankton a to bacteria b via all DOM*

$$X_{a>b}=\sum_{d} X_{d>b,b} f_{a>d,in,T}$$

Note: Sum of DOM coming from all bacteria is less than sum of DOM because bacteria area also a source of DOM.

Example: Flux from *rst* to *pol* via all DOM

$$X_{rst>pol}=X_{chr>pol,chr}+other d=0.267$$

*Fraction (f) for bacteria b coming from phytoplankton a, by bacteria*

$$f_{a>b,in}=\frac{X_{a>b}}{X_{b,in}}$$

Example: fraction for *pol* coming from *rst*, by *pol*

$$f_{rst>pol,in}=\frac{0.267}{0.459}=0.579$$
